# Local Network Topology in Human Protein Interaction Data Predicts Functional Association

**DOI:** 10.1371/journal.pone.0006410

**Published:** 2009-07-29

**Authors:** Hua Li, Shoudan Liang

**Affiliations:** 1 Department of Bioinformatics & Computational Biology, The University of Texas M. D. Anderson Cancer Center, Houston, Texas, United States of America; 2 Biomathematics & Biostatistics, Graduate School of Biomedical Sciences, the University of Texas Health Science Center at Houston, Houston, Texas, United States of America; University of Pennsylvania School of Medicine, United States of America

## Abstract

The use of high-throughput techniques to generate large volumes of protein-protein interaction (PPI) data has increased the need for methods that systematically and automatically suggest functional relationships among proteins. In a yeast PPI network, previous work has shown that the local connection topology, particularly for two proteins sharing an unusually large number of neighbors, can predict functional association. In this study we improved the prediction scheme by developing a new algorithm and applied it on a human PPI network to make a genome-wide functional inference. We used the new algorithm to measure and reduce the influence of hub proteins on detecting function-associated protein pairs. We used the annotations of the Gene Ontology (GO) and the Kyoto Encyclopedia of Genes and Genomes (KEGG) as benchmarks to compare and evaluate the function relevance. The application of our algorithms to human PPI data yielded 4,233 significant functional associations among 1,754 proteins. Further functional comparisons between them allowed us to assign 466 KEGG pathway annotations to 274 proteins and 123 GO annotations to 114 proteins with estimated false discovery rates of <21% for KEGG and <30% for GO. We clustered 1,729 proteins by their functional associations and made functional inferences from detailed analysis on one subcluster highly enriched in the TGF-β signaling pathway (*P*<10^−50^). Analysis of another four subclusters also suggested potential new players in six signaling pathways worthy of further experimental investigations. Our study gives clear insight into the common neighbor-based prediction scheme and provides a reliable method for large-scale functional annotation in this post-genomic era.

## Introduction

Due to advance in DNA sequencing, genes are being discovered at unprecedented speed, creating a need for annotating their functions. High-throughput mapping of protein-protein interaction (PPI) data is an example of functional genomics that enables rapid assignment of functional annotations by links between proteins which imply functional associations. However, due to noises inherent in the process of data generation [1], for example, by a yeast two-hybrid method [2], it becomes important to develop algorithms that reduce the influence of such noises and improve the quality of declared functional associations. So far, partial PPI networks for several organisms have been mapped [3]–[11], and different methods have been formulated to investigate these networks, and hence protein functions [12]–[27]. One method to suggest biological function is to compare the PPI network with similar random networks to identify unusual topological connectivity between proteins, which we call common-neighbor statistics. Such statistics has been used to assess the functional relationship between proteins in a yeast PPI network, and functional inferences that are statistically significant have been made from those relationships [28]. In this study, we improved upon the common-neighbor statistics, thereby enhancing the quality of functional association predictions, and applied our methods to a comprehensive human PPI dataset [29] to suggest potential functions of human proteins.

PPIs can be visualized as a graph with proteins composing the nodes, and interactions composing the edges (the graphical interactions). Ample evidence exists that such a graph is nonrandom in the topologies of its connectivity [30]–[32]. We assumed that most of the nonrandomness is necessary for the protein-interaction network to perform proper biological function. We further hypothesize, that two proteins share a number of interacting neighbors which is significantly larger than that occurred on average in truncated power-law preserving random networks can significantly enhance the likelihood of the two proteins sharing a common or related biological function. In prior work on yeast PPI network, we developed a formula for ranking the degree of rareness of such occurrences [28]. In this study, we developed an additional formula to overcome a deficiency in the previous work and make the ranking more accurate. We found that the combination of these two formulas leads to better results. We applied the method of detecting nonrandomness to the publicly available PPI dataset for humans [29]. With our clustering method, we built a 1729-protein cluster where we found most function-related proteins were clustered together and many subclusters were highly enriched in different signaling pathways. In particular, we made an in-depth analysis of the transforming growth factor β (TGF-β) pathway which is important in cell proliferation and tumorigenesis, and suggested a list of proteins presumably involved in several signaling pathways.

## Results

### Algorithms

Suppose that in a PPI network of size *N*, the degree (i.e., the number of interactions) for each protein node is fixed, but the interacting partners are randomly selected. This specifies the random network which we compare the real PPI data with. We randomly pick proteins *X* and *Y* (*X* with 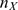 interactions and *Y* with 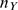 interactions) and find that *X* and *Y* share 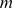 interacting partners (nodes) in this network. We denote the set of common partners as 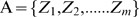, the set of all proteins as 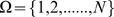, and the number of interacting partners for each protein in 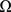 as 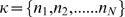.

The total number of graphs in which proteins *X* and *Y* have *m* common partners is a product of three factors: (*i*) *m* proteins can be chosen from any of the *N* proteins, and there are 
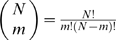
 ways to do that; (*ii*) the remaining 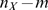 proteins that interact only with protein *X* can occupy 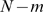 spaces still available, resulting in a count of 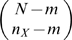; and (*iii*) 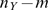 proteins that interact only with protein *Y* can be in any 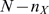 available spaces, contributing a factor of 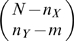. By multiplying these three factors and dividing by the total number of unrestricted ways for protein *X* to have 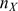 and protein *Y* to have 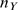 interacting partners—
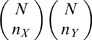
—we can arrive at the following formula (Algorithm I) by Samanta and Liang [28]:
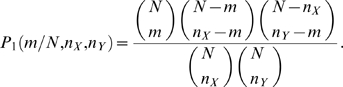
In this calculation, we have relaxed the constraint that the degree of each node remains the same. For such totally randomized networks for which only the average number of interactions per protein is fixed, our simulation showed that the probability computed by *P*_1_ is accurate.

However, a more realistic random control is to also keep the degree distribution the same as the real PPI network (i.e., to preserve the truncated power-law distribution [32]). This is much broader than a totally random network, for which the degree distribution, for a large number of interactions, decays exponentially. For such a truncated power-law random network, our simulations showed that *P*_1_ becomes inaccurate. To determine the reason behind this and to devise a compensation, we note that in any set A of *m* common partners, proteins with more interactions will appear at a higher frequency. An extreme case is that if one protein interacts with most proteins in the network (i.e., a hub protein), it is hardly a surprise to find any two proteins sharing it as a common partner. Because it is easier to observe hub proteins as common partners and because *P*_1_ only takes into account the degree of nodes on average, the significance of *P*_1_ should be down-weighted when hub proteins are involved as common partners. Therefore, we came up with another algorithm (Algorithm II) to reduce the influence of hub proteins: under the condition that all proteins are randomly connected, we used the degree 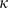
of 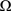 (except the degree of *X* and *Y*) to compute the probability that only 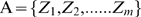 connects to *X* and *Y*, and we derived the probability as follows:
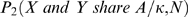


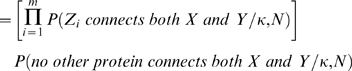


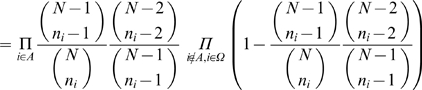


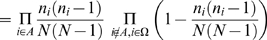
In supporting information (Text S1), we show that the second product is bounded from both above and below; and hence, we use the approximation 
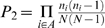
.

Therefore, each protein pair with common neighbor(s) was assigned with both *P*_1_ and *P*_2_. In previous work, Samanta and Liang [28] used only *P*_1_ to rank the relationship of protein pairs. For our method, we added *P*_2_ as a complementary algorithm to improve the biological inference. We showed that by reducing the influence of hub proteins in the network, the use of both *P*_1_ and *P*_2_ allowed us to identify a more reliable functional relationship than that identified by *P*_1_ alone.

### Comparing Network Topology between Real and Randomized PPI Networks

**Figure 1:**
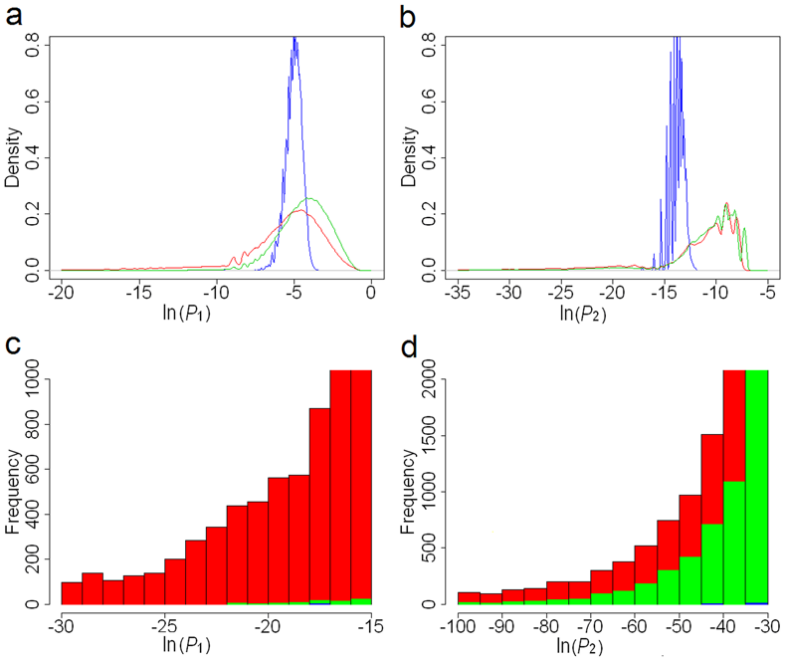
Density distributions and histograms of probabilities derived from our method. Red lines and bars: probabilities calculated from the human PPI network; green lines and bars: probabilities from truncated power law–preserving random networks; blue lines and bars: probabilities from simple random networks. (*a*) Density distributions of *P*_1_. (*b*) Density distributions of *P*_2_. (*c*) Histograms of *P*_1_. (*d*) Histograms of *P*_2_.

We computed the probabilities (*P*_1_ and *P*_2_) according to Algorithms I and II for 311,023 protein pairs that had at least one common neighbor, and plotted the distribution of the probabilities (Fig. 1*a and 1b*). In this paper, all the probabilities have been natural [base *e*] logarithm transformed. To assess the statistical significance of the topological connections in the human PPI network, we computed and compared the distributions of probabilities calculated from Algorithms I and II in suitably randomized networks. There are two ways to randomize the PPI network: (*i*) randomly connect nodes (proteins) but keep the total number of edges (interactions) the same (i.e., simple random network); and (*ii*) in addition to (*i*), keep the number of interacting partners of each protein the same as in our real PPI network (i.e., a truncated power law–preserving random network). Compared to simple randomization, for both Algorithms I and II, the truncated power law–preserving randomization produced a probability distribution more similar to that of the real PPI network (Fig. 1). As a biological network is a network with a truncated power-law distribution [32], it is more realistic to use a truncated power law–preserving random network as the background for comparisons. We use “random network” hereafter to refer to a truncated power law–preserving random network, unless otherwise specified. As expected, the human PPI network has much more highly improbable topological connections that happen by chance only with a very low probability (Fig. 1*c and 1d*).

### Ranking Protein Pairs and Suggesting Functionally Associated Protein Pairs

**Figure 2:**
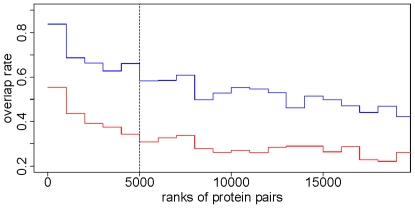
Annotation overlap rate with GO and KEGG as the benchmarks. Protein pairs are ranked by *P*_1_. The top-ranked 20,000 pairs are divided equally into 20 bins. In each bin (1,000 protein pairs), we calculated the GO overlap rate and the KEGG overlap rate. The red curve stands for the GO overlap rates and the blue one stands for the KEGG overlap rates. The dashed line is the cutoff at the 5,000th protein pair. The correlation coefficient between the two groups of overlap rates is 0.928 (P<0.0001).

Ideally, given that *P*_1_ assesses the degree of nonrandomness in the network, which indicates the functional association, we anticipated that *P*_1_ should rank our protein pairs in a way that reflected their functional relevance. Therefore, we hypothesized that a higher ranking (i.e., a better *P*_1_) corresponds to a closer biological relationship. With the Gene Ontology (GO) annotations [33] and Kyoto Encyclopedia of Genes and Genomes (KEGG) pathway annotations [34] as benchmarks, we used annotation overlap rates (see Methods and Materials) to validate the reliability of the protein pair ranking from *P*_1_, and preliminarily determined functionally associated protein pairs (i.e., significant protein pairs). We noted that in the top 5,000 protein pairs, each 1,000 pairs always had a higher overlap rate than those beyond the top 5,000 pairs, and that the region of high overlap will give us a high level of confidence in presenting reliable predictions (Fig. 2). Thus, we chose the 5,000th value of *P*_1_ (−17.11) as the cutoff from Algorithm I. It was interesting that the probability perfectly matched the Bonferroni correction 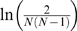 in which *N* = 7,362 is the size of the whole protein network. The false discovery rate (FDR) [35], which was used to assess the effectiveness of our method, is 0.40 for the top 5,000 functional associations selected by Algorithm I, with the cutoff at −17.11 (for our definition of FDR, see Methods and Materials).

**Figure 3:**
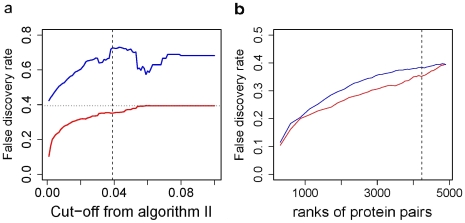
Algorithm II decreased the false discovery rate (FDR) of our predictions. (*a*) For the top 5,000 protein pairs ranked by *P*_1_, each cutoff value from *P*_2_ (on the *x* axis is the quantile of *P*_2_ we used as the cutoffs) divided them into two groups: Group I (red line), whose *P*_2_ was better than the cutoff, and Group II (blue line), whose *P*_2_ was worse than the cutoff. In this plot, the maximal difference between the two groups is at 0.039 (vertical dashed line), which corresponds to the cutoff of −30.03 from Algorithm II. The horizontal dotted line stands for the FDR (0.40) of the top 5,000 protein pairs ranked by *P*_1_. (*b*) The blue line (Group III) shows the FDR of protein pairs ranked by *P*_1_ only (*x* axis stands for the amount of selected top protein pairs), and the red line (Group I) shows the FDR of the significant protein pairs selected by *P*_1_ and *P*_2_ together.

In a real PPI network, it is common to have many hub proteins with large numbers of interacting neighbors. *P*_2_ is designed to reduce the influence of these hub proteins within the top 5,000 protein pairs selected by *P*_1_ as we believe that *P*_2_ can identify protein pairs whose lower *P*_1_ is caused by common neighbors that are hub proteins and remove them from the list of significant protein pairs. With GO and KEGG as the benchmarks, the utility of *P*_2_ is then confirmed by the following assertions: (*i*) the protein pairs with a good *P*_2_ (Group I) always have a lower FDR (here a lower FDR means a closer functional relationship) than those without a good *P*_2_ (Group II; Fig. 3*a*); and (*ii*) the protein pairs with a good *P*_2_ (Group I) always have a lower FDR than the same number of top protein pairs ranked by *P*_1_ only (Group III; Fig. 3*b*). We also noted that because *P*_1_ and *P*_2_ have a very low linear correlation (Pearson's correlation coefficient = −0.033, *P*<10^−16^; also see Fig. S1 *a* in supporting information) and rankings of functional association by *P*1 and *P*2 are significantly different (*P*<10^−16^), an additional cutoff from Algorithm II makes difference from merely tightening the cutoff from Algorithm I. As the cutoff for *P*2 changes, the difference in FDR between Groups I and II varies; the difference maximizes when the cutoff goes to −30.03, which is the value we used for the second cutoff from Algorithm II (Fig. 3*a*). Therefore, 4,233 significant protein pairs (*P*<0.001; see Table S1 in supporting information) were considered to have a close functional association in terms of the cutoffs from Algorithm I (−17.11) and Algorithm II (−30.03). In addition, the 4,233 significant pairs had a FDR of 0.35, compared with 0.39 for the top 4,233 pairs ranked by *P*_1_ only (Fig. 3*b*), 0.83 for the top 4233 pairs from the truncated power law–preserving random network [cutoffs: −8.90 for ln(*P*_1_) and −11.33 for ln(*P*_2_)] and 0.92 from the totally randomized network [cutoffs: −6.42 (*P*_1_) and −13.10 (*P*_2_)].

### Estimate the Lower Bound of FDR for the 4233 significant protein pairs

Because the functional annotations for human proteins are far from complete, the proportion of true positive functional associations must be higher and thus the FDR should be lower than 0.35. To estimate the lower bound of the FDR, we took into consideration the behavior of the random network by computing what percentage of the 4,233 protein pairs were generated by chance. As biological networks are networks with a truncated power-law distribution [32], we used only a truncated power law–preserving random network as the background. Cut by the same cutoff [−17.11 for ln(*P*_1_) and −30.03 for ln(*P*_2_)], the power law–preserving random networks have on average 86 protein pairs as significant associations (Fig. S1). The lower bound of FDR is the false discovery number generated in random network (86) divided by the number of predicted significant associations (4233), which is approximately 2%.

### Significant Protein Pairs Are Informative in Functional Inference

We observed strong functional relationships among the top 4,233 protein pairs. After manual inspection, we found that at least 96 of the top 100 annotated protein pairs (excluding pairs with unannotated proteins) have close functional relationships and we listed the top 10 pairs in Table 1.

**Table 1:** Top 10 protein pairs from our 4,233 significant protein pairs.

Protein_A	Protein_B	Ln(*P*1)	Functional Relationship
SMAD3	SMAD2	−157.6068	SMAD family member
TUBB	TUBB2	−136.0437	Cellular structural activity
PTPN11	PTPN6	−125.8552	Proliferation of cells
BMPR1B	TGFBR1	−124.9466	Differentiation of cells
CALM2	CALM3	−124.9368	Calcium-modulated proteins
MAPK1	MAPK3	−113.0905	MAP kinase family member
CALM1	CALM3	−112.6375	Calcium-modulated proteins
IXL	MED9	−107.7585	Mediator complex
PIK3R1	GRB2	−107.7070	Tyrosine phosphorylation
CALM1	CALM2	−106.1716	Calcium-modulated proteins

All of them share close functional relationships.

The GO and KEGG-based FDR for 23,782 direct interactions is 0.57, which is significantly higher than our FDR of 0.35 (*P*<10^−16^, two-sample proportion test). This comparison supports the notion that our method offers more reliable functional associations than the human PPI data itself does. Because only 21.6% of the 4,233 protein pairs interact directly in the PPI data, we believe that the rest of them provide additional functional information that is not revealed in the PPI data.

We used GO and KEGG annotations to compare functions and compute annotation overlaps. Among the 1,754 proteins in the top 4,233 protein pairs, 1,220 have qualified GO terms (i.e., GO terms at the highest level without direct or indirect GO “offspring” terms in each ontology), and 834 have KEGG pathway annotations. If a protein has at least one annotated significant partner (i.e., two proteins are significant partners to each other if they are a significant protein pair), a list of annotation(s) from its partner(s) can be sorted by frequency and annotations occurring at the highest frequency are assigned to this protein (frequency must be at least twice for KEGG and four times for GO; otherwise discarded. For more details, see Text S1 and Fig. S3 in supporting information). For an annotated protein (based on GO and KEGG annotations), if an assigned annotation occurs among its known functions, we consider this to be a correct prediction. By this method, we found that 79% (for KEGG) and 70% (for GO) of assigned annotations were correct predictions. (Randomly picking 4233 pairs from 1729 proteins will only yield a 7% correct prediction rate for KEGG and 12% for GO on average from 100 trials.) In the same way, we predicted 466 KEGG pathways for 274 proteins and 123 GO terms for 114 proteins. We estimated that the FDRs of our predictions are much less than 21% (for KEGG) and 30% (for GO) because of the percentage of correct predictions for annotated proteins and the incompleteness of GO and KEGG annotations. We arbitrarily selected 40 predicted annotations (20 for KEGG and 20 for GO) and listed them in Table 2. For complete predictions, see Table S2 in supporting information.

**Table 2:** Selected Predictions of KEGG and GO annotations for human proteins.

**Protein**	**KEGG**	**KEGG Pathway Name**	**Ratio**
**CDC5L**	hsa04110	Cell cycle	4/5
**DEDD**	hsa04210	Apoptosis	4/5
**KSR2**	hsa04010	MAPK signaling pathway	4/5
**GMFB**	hsa04010	MAPK signaling pathway	6/6
**ITGB1**	hsa04640	Hematopoietic cell lineage	4/6
**PTK2B**	hsa04630	Jak-STAT signaling pathway	21/68
**GDF9**	hsa04350	TGF-beta signaling pathway	5/5
**ZIC1**	hsa04340	Hedgehog signaling pathway	3/3
**GRAP2**	hsa04664	Fc epsilon RI signaling pathway	5/7
**ACTR2**	hsa04810	Regulation of actin cytoskeleton	5/6
**PLCG2**	hsa04660	T cell receptor signaling pathway	6/8
**CD2**	hsa04660	T cell receptor signaling pathway	5/6
**TRPV4**	hsa04670	Leukocyte transendothelial migration	3/10
**USP7**	hsa04060	Cytokine-cytokine receptor interaction	11/15
**CCBP2**	hsa04060	Cytokine-cytokine receptor interaction	4/6
**SLA**	hsa04650	Natural killer cell mediated cytotoxicity	4/5
**CSK**	hsa04650	Natural killer cell mediated cytotoxicity	10/15
**RGS16**	hsa04080	Neuroactive ligand-receptor interaction	5/14
**STX1A**	hsa04130	SNARE interactions in vesicular transport	5/6
**NAPA**	hsa04130	SNARE interactions in vesicular transport	4/6
**Protein**	**GO ID**	**GO Term**	**Ratio**
**KHDRBS1**	GO:0005524	ATP binding	5/18
**GNAI1**	GO:0003924	GTPase activity	4/4
**COL1A2**	GO:0005587	collagen type IV	6/9
**MCM10**	GO:0008270	zinc ion binding	10/26
**FN1**	GO:0005509	calcium ion binding	6/18
**SAA1**	GO:0005509	calcium ion binding	4/11
**ATP2B4**	GO:0030955	potassium ion binding	4/16
**ACTR2**	GO:0005885	Arp2/3 protein complex	6/6
**BLNK**	GO:0005070	SH3/SH2 adaptor activity	4/15
**CD28**	GO:0005070	SH3/SH2 adaptor activity	4/12
**DLG4**	GO:0004385	guanylate kinase activity	4/11
**TIF1**	GO:0003714	transcription corepressor activity	4/12
**GADD45G**	GO:0030521	androgen receptor signaling pathway	4/8
**TNFRSF17**	GO:0005031	tumor necrosis factor receptor activity	4/11
**TNFRSF8**	GO:0005031	tumor necrosis factor receptor activity	4/14
**SOCS3**	GO:0005159	insulin-like growth factor receptor binding	4/10
**PTPN1**	GO:0005159	insulin-like growth factor receptor binding	4/14
**FAS**	GO:0043123	positive regulation of I-kappaB kinase/NF-kappaB cascade	5/6
**CASP10**	GO:0043123	positive regulation of I-kappaB kinase/NF-kappaB cascade	4/6
**MAP3K14**	GO:0043123	positive regulation of I-kappaB kinase/NF-kappaB cascade	7/17

The 2^nd^ column is the predicted KEGG and GO IDs for proteins in the 1^st^ column. The 3^th^ column is the corresponding KEGG pathway name and GO term. Ratio is the number of significant partners with the assigned annotation(s) divided by the total number of significant partners.

### Clustering from the Significant Protein Pairs

Because clustering can significantly improve the quality of functional inference [28], we built a cluster consisting of 1,729 proteins (excluding 25 non-human proteins) based on the *P*_1_ of 4,233 significant protein pairs. We constructed the empirical cumulative distribution from these *P*_1_ values; thus, each significant protein pair had a score between 0 and 1 according to its ranking order in the distribution of *P*_1_. Then we built a 1729×1729 dissimilarity matrix in which each matrix element was assigned either a score (if applicable) or a “10” for pairs with no significant *P*_1_. The purpose of using such a large value was to minimize background noise. Then the dissimilarity matrix was subjected to agglomerative hierarchical clustering with an unweighted pair-group average. The whole cluster is given in Fig. S2 in supporting information.

### Analysis of Functional Modules with Significant *P* Values

**Figure 4:**
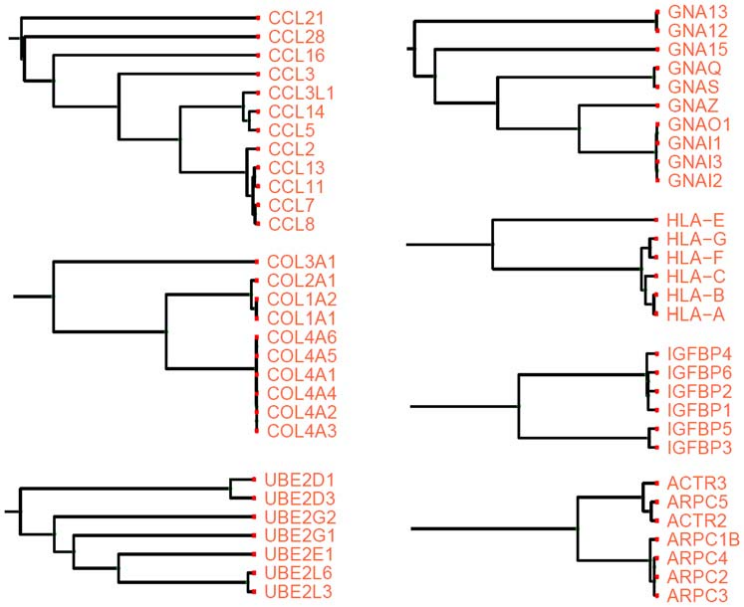
Examples of subclusters derived from the significant 4,233 protein associations. Apparently each of them belongs to the same functional module in which they perform similar or the same biological functions.

In the cluster of 1,729 proteins, most of the functionally related proteins were correctly clustered into their corresponding functional modules, in which they are characterized by similar functions or the same pathway (Fig. 4). The largest subcluster derives directly from the root of the whole cluster and consists of 959 proteins; the second-largest subcluster has only 51 members (Fig. S2). We cut the 959-member subcluster with different cutoff values and analyzed the corresponding subclusters by using both manual inspection and Ingenuity Pathway Analysis (IPA). We conducted a detailed analysis for one prominent subcluster (the subcluster related to the TGF-β signaling pathway) as a reference.

**Figure 5:**
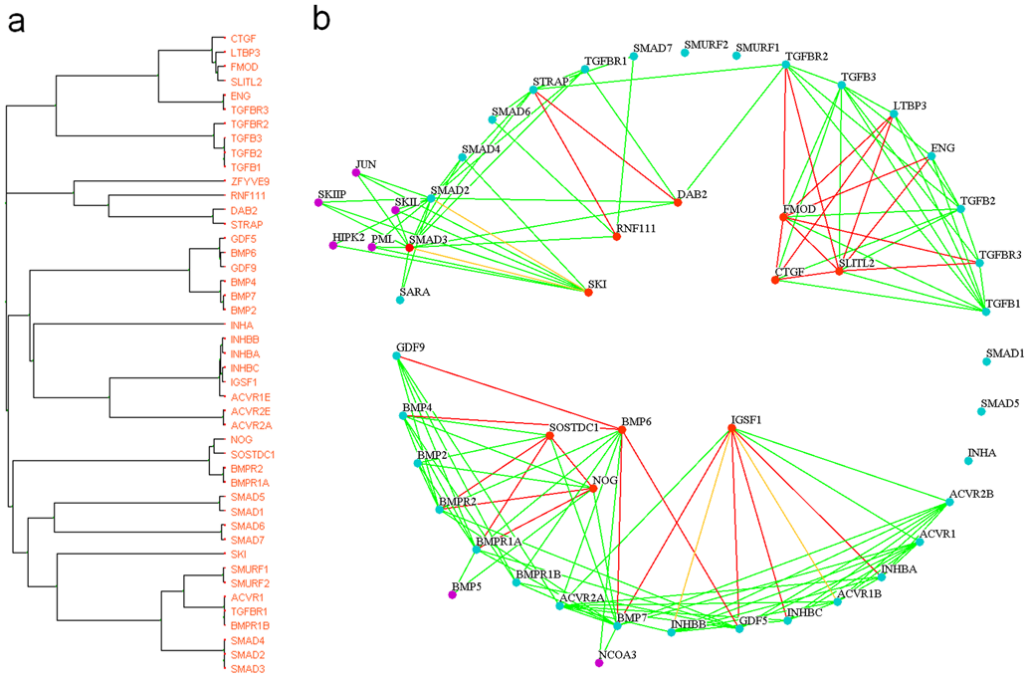
TGF-β signaling pathway–related subcluster. (*a*) One subcluster identified by our method consists of proteins presumably involved in the TGF-β signaling pathway. (*b*) Detailed interpretation of the relationships between each protein from the subcluster. On the basis of the Ingenuity Pathway Analysis 5.0, the 35 blue-green proteins on the circle participate in the TGF-β signaling pathway, and the 10 red proteins inside the circle are unrelated. The violet proteins outside the circle are common neighbors that do not belong to the subcluster in panel *a*. Red lines represent significant protein pairs, green lines represent direct protein–protein interactions, and yellow lines represent both.

The TGF-β signaling pathway–related subcluster (Fig. 5*a*) has a total of 45 protein members, 35 of which are known to participate in the TGF-β signaling pathway, according to the Ingenuity database. The probability of observing this by chance is <10^−54^, according to the calculation from Ingenuity software (right-tailed Fisher's exact test). With respect to this extreme *P* value, we reasoned that probably all the cluster members cooperate to mediate signal transduction. To investigate the role of the other 10 proteins in the TGF-β signaling pathway, we generated a functional relationship network using Osprey software (http://biodata.mshri.on.ca/osprey) [36] to explicitly elucidate the relationships between the 45 proteins (Fig. 5*b*): the 10 proteins not related to TGF-β according to the Ingenuity database are located inside a circle, whereas the other 35 TGF-β member proteins lie on the circle; common neighbors which do not belong to the 45-member subcluster stay outside the circle.

The cluster and the association network (Fig. 5*a and 5b*) intuitively suggest possible roles that the inner proteins play in the TGF-β signaling pathway, which have not yet been incorporated into the Ingenuity pathway. Take Fig. 5*b* for instance: SKI functions as both the significant partner and the direct interacting neighbor of SMAD2 and SMAD3, and the three proteins' common neighbors (five violet nodes) all share the function of transcriptional regulation. From this we infer that SKI may regulate the TGF-β signaling pathway on a transcription level, which is in accordance with findings in the literature (but has not been incorporated into the Ingenuity database) that SKI regulates downstream DNA transcription by forming a protein complex with SMAD2 and SMAD3 [37], [38]. With respect to IGSF1's significant partners, direct-interaction partners, and the previous work identifying IGSF1 as a potential receptor that could affect cellular response through its cytoplasmic region [39], we suspect that IGSF1 could function as a coreceptor for inhibin and/or activin. SOSTDC1 and NOG may regulate TGF-β by interacting with BMP receptors, which is in accordance with the findings that both of them function as BMP antagonists [40], [41]. In addition to positive regulatory functions [42], DAB2 may serve as an antagonist of STRAP, which has a negative regulation on TGF-β–mediated transcriptional activation [43], [44]. FMOD, CTGF, and SLITL2 may be involved in regulating receptor binding of TGF-βs, in accordance with published findings [45]–[47], and they may interact with each other. Thus, through integration with information from known networks, our method (probability, probability–derived clusters and networks) suggests new features which we can further investigate in experiments.

**Figure 6:**
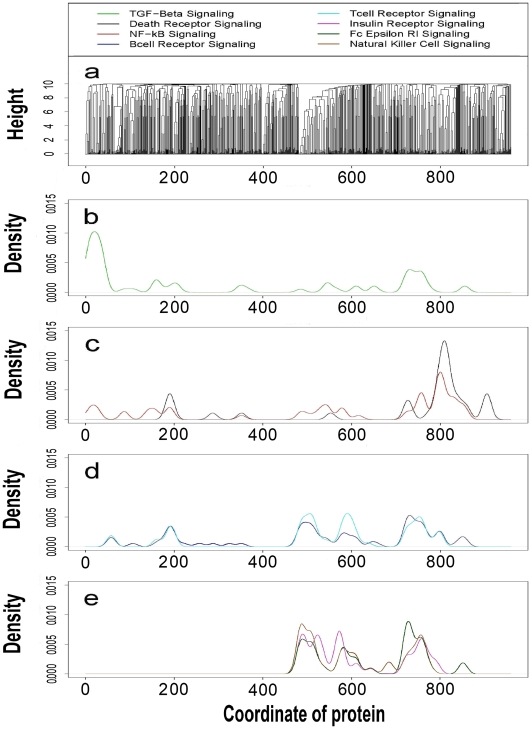
Distribution patterns of eight different signaling pathways. (*a*) The largest subcluster of 959 proteins is derived from the root of the whole 1729-member cluster. Each protein in this subcluster has a coordinate with respect to its order in the 959 members (from left to right); a pathway distribution is generated from the distribution of its members' coordinates under the bandwidth of 10 (R 2.25; IPA 5.5). (*b*) Distribution of the TGF-β signaling pathway. (*c*) Distributions of death-receptor and NF-κB signaling pathways. (*d*) Distributions of B- and T-cell receptor signaling pathways. (*e*) Distributions of insulin receptor, Fc epsilon RI and natural killer cell signaling pathways.

**Figure 7:**
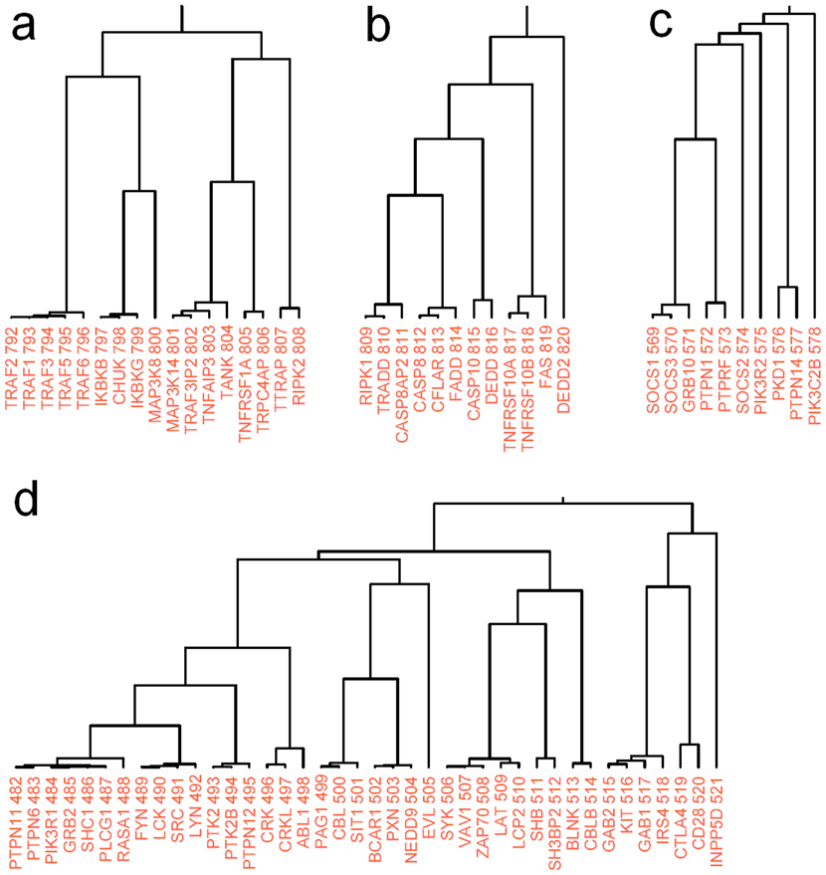
Four subclusters that are presumably involved in six signaling pathways (indices above protein names are their coordinates in Fig. 7 *a*). (*a*) Cluster for the NF-κB signaling pathway (*P*<3.2×10^−20^): TRAF1, TRAF3IP2, TANK, TRPC4AP and RIPK2 are potential pathway members. (*b*) Cluster for death receptor signaling pathway (*P*<2.4×10^−19^): CASP8AP2, DEDD and DEDD2 are potential members in this pathway. (*c*) Cluster for insulin receptor signaling pathway (*P*<2.4×10^−10^): SOCS2, PKD1, PTPN14 are potential pathway members. (*d*) Cluster for the immune response [T-cell receptor signaling (*P*<4.9×10^−22^), B-cell receptor signaling (*P*<1.1×10^−17^) and natural killer cell signaling (P<1.7×10^−21^)]: SRC, PTK2, PTK2B, PTPN12, CRK, CRKL, SIT1, PXN, NEDD9, EVL, KIT and IRS4 are potential members of the above three pathways.

To facilitate analysis of this type, we proposed eight signaling pathways with extreme *P* values (<10^−40^, from IPA 5.0) that are worthy of further investigations (Fig. 6). The proteins within the same signaling pathway tend to stay together in the same subclusters. This is shown for the largest 959-member subcluster (Fig. 6*a*; cluster members are indexed from 1 to 959). From IPA-based classification of the proteins into each of the eight pathways, we calculated a density distribution for all eight signaling pathways along the cluster (Fig. 6*b–e*). Each pathway is expected to have a distinct distribution (its own peaks). The peaks in Fig. 6*b–e* map to some areas (i.e., subclusters) that are probably highly related to their corresponding pathways. Functionally intercrossed pathways, like death-receptor/NF-κB signaling, may have close peaks. The distribution patterns are useful in identifying pathway-specific regions in the cluster. We selected another 4 subclusters that are presumably involved in six signaling pathways (excluding TGF-β) with respect to pathway member distributions, and listed the potential pathway members in Fig. 7. We expect that the clusters and distributions will help biologists to find their subcluster of interest and discover new pathway members.

## Discussion

An advantage of our prediction scheme, inherited from Samanta and Liang (2003), is the insensitivity to the high false positive rate of high-throughput PPI data. After adding 6086 randomly generated interactions (30.4% of the real data, assuming at least 50% false positive rate for high-throughput data), we were still able to recover on average 93.4% of significant protein pairs; furthermore, >90% of falsely generated “significant protein pairs” will become significant if we loosen the cutoffs of *P*1 and *P*2 a little to double the number of significant protein pairs. This will certainly offer more flexibility when selecting which PPI data to use.

We compared the performance of our prediction scheme with that of the direct prediction scheme used by Schwikowki *et al*. (2000) which infers the function of a protein from it direct interacting neighbors in the PPI network [48], [49]. Under the same criteria (i.e., the minimum frequency of shared functions required to assign annotations), the FDRs of our predictions (30% for GO and 21% for KEGG) have been significantly improved over the FDRs (60% for GO and 49% for KEGG) from the direct prediction scheme [48]. This result is reasonable because our algorithms identified significant protein pairs that are more functionally associated than the direct-interacting pairs in the human PPI data, and we made functional inferences from these significant pairs, not from direct protein interactions which may suffer large amounts of false positives generated in high throughput assays.

Human proteins may have multiple functions and belong to different functional modules, so different signaling pathways may also have some pathway members in common. It is thus reasonable to assume that the overlap of distribution (Fig. 7*b–e*), especially of peaks, may reveal the functional relevance of different pathways. For example, the death-receptor and NF-κB signaling pathways overlap in the peak area, and the T- and B-cell receptor signaling pathways have a similar distribution. Therefore, the cluster and its pathway distributions will be useful in multi-pathway analysis and accurate function prediction.

We also developed a new algorithm for computing the probabilities that three proteins share *m* interacting partners (see Text S1 in supporting information). However, we found that if three proteins have a very low probability of sharing *m* interacting partners, in most cases two of them will have a very low *P*_1_. Because this algorithm is highly dependent on Algorithm I (*P*_1_), we do not think it provides more information worthy of further investigation.

In conclusion, we proposed an improved method to predict protein functional association and make reliable functional annotations; we derived a cluster to investigate signaling pathways and suggest potential novel pathway members. We believe that with the explosion of available human PPI data, our method will contribute greatly to the functional research of human proteins.

## Materials and Methods

### Protein–Protein Interaction Data

From the BioGRID (www.thebiogrid.org), we downloaded the human PPI data (version 2.20), which derived from both conventional focused studies (∼69.6%) and high-throughput studies (yeast two-hybrid; ∼30.4%) [29]. There are 20,019 total non-redundant interactions (excluding self-interactions) and 7,362 protein entries in this dataset, including 42 nonhuman proteins that interact with human proteins.

### Benchmarks for evaluating the functional association

We used GO and KEGG as independent benchmarks to assess the functional association of each protein pair. GO and KEGG databases provide specific pathways, functions and cellular components for proteins in our PPI data: we classified the 7,362 proteins into 237 KEGG pathways and 1956 qualified GO terms (including biological process, molecular function and cellular component). These databases are good references for evaluating functional association because of its reasonable coverage of the genome and its large number of categories, which makes it improbable to have random matching of pathways.

### Annotation overlap rate

With GO annotation (R package: GO, 08-Aug-2006), we defined the GO overlap rate as follows: 
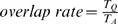
, where 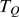 is the number of protein pairs of which both proteins share at least one qualified GO term; 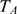 is the number of protein pairs of which both proteins are annotated with qualified GO terms. Here “qualified GO terms” means GO terms at the highest level without direct or indirect GO “offspring” terms in each ontology (the level is defined as the number of nestings from the root node (level 1) in the Gene Ontology DAG file [33]).

We defined the KEGG overlap rate in the same way as above (R package: KEGG, Release 41.1). We used the GO and KEGG overlap rates to assess the functional association of protein pairs: a higher overlap rate corresponds to a closer functional relationship.

### Definition of FDR for the declared significant functional associations

Suppose the GO and KEGG pathways are complete: if both proteins in each pair have KEGG pathway identifiers and qualified GO terms, we call them declared positive protein pairs. If they share at least one identifier (either GO or KEGG identifier), we consider this declared association true positive; otherwise we consider it false positive. Therefore, the FDR can be written as follows: 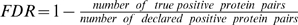


This false discovery rate is used to assess the performance of our algorithm as we expect an improved annotation scheme will lower the proportion of wrong predictions among declared significant functional associations.

### Pathway analysis tool

We used Ingenuity® Pathway Analysis (IPA) 5.0 software (Ingenuity Systems, Inc., Redwood City, CA) to identify existing pathway members and calculate *P* values for signaling pathways identified in our cluster.

## Supporting Information

Text S1 (0.12 MB DOC)

Figure S1 Density plot of the distributions of P_1_ and P_2_ (two dimensions) from the human protein-protein interaction (PPI) network (a) and the randomized but truncated power-law preserving PPI network (b). The vertical and horizontal lines stand for the thresholds from Algorithms I and II, respectively. In a random PPI network (with truncated power-law), the expectation of significant protein associations is 86 (lower left in b) compared with 4,233 significant associations in the real PPI network (lower left in a).
(0.15 MB TIF)

Figure S2 A cluster that consists of 1729 human proteins. Indices above protein names are their coordinates in this cluster.
(0.08 MB PDF)

Figure S3 Estimation of prediction precise rates and the number of predictions we can make given different *n* (*n* is the minimal frequency of annotation occurrence required for functional prediction). (a) Estimated precise rate of predicted KEGG pathways given *n*. (b) The number of predictions for KEGG pathway we can make given *n*. (c) Estimated precise rate of predicted GO terms given *n*. (d) The number of predictions for GO terms we can make given *n*.
(0.03 MB TIF)

Table S1 The 4233 significant protein pairs derived by our method. There are totally 1,729 human proteins and 25 nonhuman proteins. Protein pairs are ranked in terms of P_1_.
(5.43 MB DOC)

Table S2 Predictions of 466 KEGG pathways for 274 proteins and 123 GO annotations for 114 proteins. The 2nd column is the predicted KEGG and GO IDs for proteins in the 1st column, with 3rd column as corresponding KEGG pathway names and GO terms. Ratio is the number of significant partners with the assigned annotation(s) divided by the total number of significant partners.
(0.40 MB DOC)
