# Supplementary material for: Local Network Topology in Human Protein Interaction Data Predicts Functional Association
Source: PLoS One. 2009 Jul 29;4(7):e6410. doi: 10.1371/journal.pone.0006410 (PMC2713831; doi:10.1371/journal.pone.0006410)
Supplement: Text S1 — (0.12 MB DOC) [file pone.0006410.s001.doc]

**Supporting Information**

**Algorithm II:**

$$P_{2}(X and Y share A|\kappa,N)$$

$$=[\prod_{i=1}^{m} P(Z_{i} connects both X and Y|\kappa,N)] P(no other protein connects both X and Y |\kappa,N)$$

$$=\prod_{i\in A} \frac{\left(\begin{matrix} N-1 \\ n_{i}-1 \end{matrix}\right)}{\left(\begin{matrix} N \\ n_{i} \end{matrix}\right)}\frac{\left(\begin{matrix} N-2 \\ n_{i}-2 \end{matrix}\right)}{\left(\begin{matrix} N-1 \\ n_{i}-1 \end{matrix}\right)} \prod_{i\notin A,i\in\Omega} (1-\frac{\left(\begin{matrix} N-1 \\ n_{i}-1 \end{matrix}\right)}{\left(\begin{matrix} N \\ n_{i} \end{matrix}\right)}\frac{\left(\begin{matrix} N-2 \\ n_{i}-2 \end{matrix}\right)}{\left(\begin{matrix} N-1 \\ n_{i}-1 \end{matrix}\right)})$$

$$=\prod_{i\in A} \frac{n_{i}(n_{i}-1)}{N(N-1)} \prod_{i\notin A,i\in\Omega} (1-\frac{n_{i}(n_{i}-1)}{N(N-1)})$$

Let $M_{1}=\prod_{i_{1}\notin A_{1}, i_{1}\in\Omega} (1-\frac{n_{i}(n_{i}-1)}{N(N-1)})$ for any randomly picked protein $X_{1}$ and $Y_{1}$; let $M_{2}=\prod_{i_{2}\notin A_{2},i_{2}\in\Omega} (1-\frac{n_{i}(n_{i}-1)}{N(N-1)})$ for any randomly picked protein $X_{2}$ and $Y_{2}$. Also let $Q=\{i_{1}\}\cap\{i_{2}\}$. We can easily see that there are more then $N-2m$ common elements in *Q*. Therefore, we have the following inequality:

$$\frac{\min(M_{1})}{\max(M_{2})}\leq\frac{M_{1}}{M_{2}}\leq\frac{\max(M_{1})}{\min(M_{2})}$$

$$\Rightarrow\prod_{i=1}^{2m} (1-\frac{\max(n_{i})(\max(n_{i})-1)}{N(N-1)})\leq\frac{M_{1}}{M_{2}}\leq\frac{1}{\prod_{i=1}^{2m} (1-\frac{\max(n_{i})(\max(n_{i})-1)}{N(N-1)})}$$

For our human PPI network, *N* = 7,362, $1\leq m\leq45$, $1\leq n_{i}\leq157$. (Simulations 1,000 times for the simple random PPI network and the power law–preserving random PPI network also show the same result.). Thus we arrive at the following inequality:

$$\Rightarrow0.96\leq\frac{M_{1}}{M_{2}}\leq1.04$$

$\Rightarrow M_{1}\approx M_{2}$ and $\log(M_{1})\approx\log(M_{2})$

Thus, we consider $\prod_{i\notin A,i\in\Omega} (1-\frac{n_{i}(n_{i}-1)}{N(N-1)})$ a constant, and we derive $P_{2}\propto\prod_{i\in A} \frac{n_{i}(n_{i}-1)}{N(N-1)}$. For convenience, we use $P_{2}=\prod_{i\in A} \frac{n_{i}(n_{i}-1)}{N(N-1)}$ in our paper.

**Mathematical expression for the probability that three proteins share *m*** **interacting partners:** To compute this probability, we count the number of distinct ways in which three proteins with $n_{1}$, $n_{3}$ and $n_{3}$ interacting partners have *m* in common. We divide the whole set of partners of the three proteins into seven nonoverlapping groups: (*i*) *m* common protein partners that interact with proteins 1, 2 and 3; (*ii*) $n_{12}-m$ proteins that interact only with proteins 1 and 2; (*iii*) $n_{23}-m$ proteins that interact only with proteins 2 and 3; (*iv*) $n_{13}-m$ proteins that interact only with proteins 1 and 3; (*v*) $n_{1}-n_{12}-n_{13}+m$ partners that interact only with protein 1; (*vi*) $n_{2}-n_{12}-n_{23}+m$ partners that interact only with protein 2; and (*vii*) $n_{3}-n_{13}-n_{23}+m$ partners that interact only with protein 3. We count the total number of distinct ways of assigning these seven groups to *N* proteins. This is given by:

$\left(\begin{matrix} N \\ m \end{matrix}\right)\left(\begin{matrix} N-m \\ n_{12}-m \end{matrix}\right)\left(\begin{matrix} N-n_{12} \\ n_{23}-m \end{matrix}\right)\left(\begin{matrix} N-n_{12}-n_{23}+m \\ n_{13}-m \end{matrix}\right)\left(\begin{matrix} N-n_{12}-n_{23}-n_{13}+2m \\ n_{1}-n_{12}-n_{13}+m \end{matrix}\right)\left(\begin{matrix} N-n_{1}-n_{23}+m \\ n_{2}-n_{12}-n_{23}+m \end{matrix}\right)\left(\begin{matrix} N-n_{1}-n_{2}+n_{12} \\ n_{3}-n_{13}-n_{23}+m \end{matrix}\right)$ The total number of ways to randomly pick $n_{1}$, $n_{2}$ and $n_{3}$ proteins from *N* proteins is given by: $\left(\begin{matrix} N \\ n_{1} \end{matrix}\right)\left(\begin{matrix} N \\ n_{2} \end{matrix}\right)\left(\begin{matrix} N \\ n_{3} \end{matrix}\right)$.

Therefore, the probability that three proteins share *m* interacting partners is given as follows:

$$P_{1}(m|N,n_{1},n_{2},n_{3})=\frac{\left(\begin{matrix} N \\ m \end{matrix}\right)\left(\begin{matrix} N-m \\ n_{12}-m \end{matrix}\right)\left(\begin{matrix} N-n_{12} \\ n_{23}-m \end{matrix}\right)\left(\begin{matrix} N-n_{12}-n_{23}+m \\ n_{13}-m \end{matrix}\right)\left(\begin{matrix} N-n_{12}-n_{23}-n_{13}+2m \\ n_{1}-n_{12}-n_{13}+m \end{matrix}\right)\left(\begin{matrix} N-n_{1}-n_{23}+m \\ n_{2}-n_{12}-n_{23}+m \end{matrix}\right)\left(\begin{matrix} N-n_{1}-n_{2}+n_{12} \\ n_{3}-n_{13}-n_{23}+m \end{matrix}\right)}{\left(\begin{matrix} N \\ n_{1} \end{matrix}\right)\left(\begin{matrix} N \\ n_{2} \end{matrix}\right)\left(\begin{matrix} N \\ n_{3} \end{matrix}\right)}$$

**Assessing the reliability of functional predictions for GO and KEGG annotations**. If a protein has at least one annotated significant partner, a list of annotation(s) from its partner(s) can be sorted by frequency. Suppose that annotations occurring *n* times or more will be assigned to this protein. For an annotated protein (based on GO and KEGG annotations), if an assigned annotation occurs among its known functions, we consider this a correct prediction. Assuming that GO and KEGG annotations are complete for those annotated proteins, we define a prediction precise rate as $\frac{total number of correct predictions}{total number of predictions}$ and, as *n* varies, we have different precise rates (Fig. S3) which we used to estimate the FDRs of our functional predictions ($FDR=1-precise rate$), hence to assess the reliability of our functional predictions. From Fig. S3, we decided to use n=2 (for KEGG) and n=4 (for GO) as the thresholds of minimum frequency of functions shared by significant partners, which gave us relatively low FDRs (21% for KEGG and 30% for GO) without sacrificing too many predictions (466 predictions for KEGG and 123 predictions for GO were made).
