# Supplementary figures and images for: Local Network Topology in Human Protein Interaction Data Predicts Functional Association
Source: PLoS One. 2009 Jul 29;4(7):e6410. doi: 10.1371/journal.pone.0006410 (PMC2713831; doi:10.1371/journal.pone.0006410)

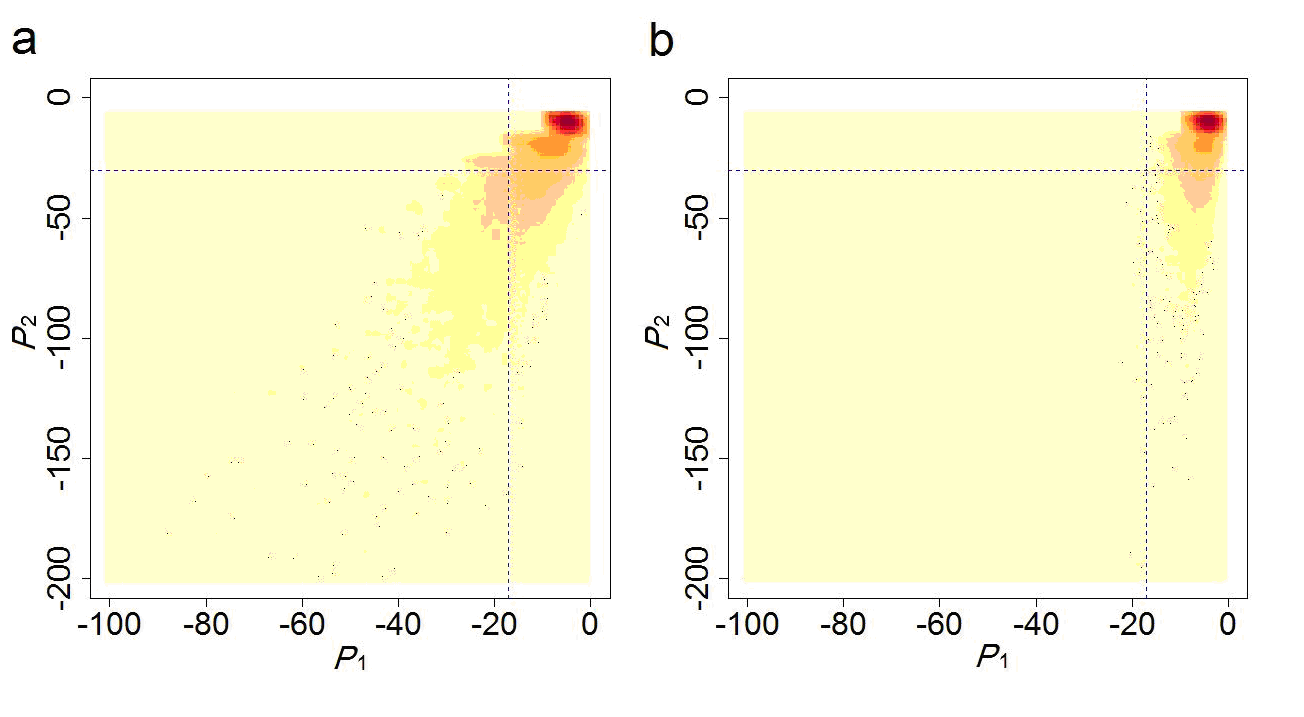

Supplement: Figure S1 — Density plot of the distributions of P1 and P2 (two dimensions) from the human protein-protein interaction (PPI) network (a) and the randomized but truncated power-law preserving PPI network (b). The vertical and horizontal lines stand for the thresholds from Algorithms I and II, respectively. In a random PPI network (with truncated power-law), the expectation of significant protein associations is 86 (lower left in b) compared with 4,233 significant associations in the real PPI network (lower left in a). (0.15 MB TIF) [file pone.0006410.s002.tif]
