# Supplementary material for: Local Network Topology in Human Protein Interaction Data Predicts Functional Association
Source: PLoS One. 2009 Jul 29;4(7):e6410. doi: 10.1371/journal.pone.0006410 (PMC2713831; doi:10.1371/journal.pone.0006410)
Supplement: Table S1 — The 4233 significant protein pairs derived by our method. There are totally 1,729 human proteins and 25 nonhuman proteins. Protein pairs are ranked in terms of P1. (5.43 MB DOC) [file pone.0006410.s005.doc]

|  | **Protein A** | **Protein B** | **Ln(*P*1)** | **Ln(*P*2)** | **# of Common Neighbors** |
| --- | --- | --- | --- | --- | --- |
| 1 | SMAD3 | SMAD2 | -157.6068404 | -597.2243748 | 45 |
| 2 | TUBB | TUBB2 | -136.0437454 | -289.8610141 | 21 |
| 3 | PTPN11 | PTPN6 | -125.8551562 | -424.3147331 | 33 |
| 4 | BMPR1B | TGFBR1 | -124.9465733 | -599.6449534 | 38 |
| 5 | CALM2 | CALM3 | -124.9367504 | -291.8124921 | 21 |
| 6 | MAPK1 | MAPK3 | -113.0905345 | -384.8694846 | 27 |
| 7 | CALM1 | CALM3 | -112.6375413 | -302.5388215 | 22 |
| 8 | IXL | MED9 | -107.7585446 | -279.4824444 | 18 |
| 9 | PIK3R1 | GRB2 | -107.7069756 | -489.2596327 | 42 |
| 10 | CALM1 | CALM2 | -106.1716277 | -306.6247978 | 22 |
| 11 | RAC1 | CDC42 | -102.793931 | -373.9517939 | 27 |
| 12 | FYN | LCK | -102.6697375 | -358.873044 | 30 |
| 13 | HDAC1 | HDAC2 | -102.2579826 | -358.5313968 | 28 |
| 14 | CREBBP | EP300 | -102.0552572 | -420.1182758 | 32 |
| 15 | GRB2 | SHC1 | -99.65932503 | -469.6442329 | 39 |
| 16 | SMAD2 | SMAD4 | -99.09164657 | -460.3519272 | 34 |
| 17 | DLG4 | DLG1 | -96.76638457 | -356.084827 | 26 |
| 18 | TRAF2 | TRAF1 | -95.58565584 | -338.2088375 | 25 |
| 19 | BCL2L1 | BCL2 | -94.54649001 | -324.8992611 | 23 |
| 20 | ACVR1 | TGFBR1 | -89.65577292 | -467.2366865 | 30 |
| 21 | CPNE1 | CPNE4 | -87.91061912 | -181.4118777 | 13 |
| 22 | MDFI | KRTAP4-12 | -87.88978999 | -438.6963486 | 30 |
| 23 | SMAD3 | SMAD4 | -85.77343234 | -435.3492838 | 33 |
| 24 | ACVR1 | BMPR1B | -84.52904218 | -343.6074392 | 23 |
| 25 | COL4A3 | COL4A2 | -82.20398173 | -167.9671211 | 13 |
| 26 | PTPN11 | GRB2 | -81.63255068 | -383.1932153 | 32 |
| 27 | COL4A2 | COL4A4 | -80.7276668 | -157.9221044 | 12 |
| 28 | PTK2 | PTK2B | -80.57310834 | -240.1404574 | 22 |
| 29 | SRC | FYN | -79.46202476 | -355.9045821 | 30 |
| 30 | COL4A3 | COL4A4 | -79.33974045 | -157.9221044 | 12 |
| 31 | FYN | LYN | -79.00597291 | -320.8620938 | 26 |
| 32 | RELA | NFKB1 | -78.83597444 | -279.429444 | 23 |
| 33 | TRAF3 | TRAF1 | -78.157227 | -213.0497868 | 16 |
| 34 | CSNK2A1 | CSNK2A2 | -77.73600946 | -181.4968663 | 15 |
| 35 | JAK1 | JAK2 | -77.22522398 | -233.6962148 | 20 |
| 36 | PTPN11 | PIK3R1 | -74.97662001 | -296.7887929 | 26 |
| 37 | COL4A2 | COL4A1 | -74.60859428 | -151.9508426 | 12 |
| 38 | YWHAZ | YWHAE | -74.52029133 | -245.9556029 | 19 |
| 39 | JUN | FOS | -74.00815036 | -250.4496582 | 21 |
| 40 | RBL2 | RBL1 | -74.0078444 | -175.0053511 | 14 |
| 41 | COL4A5 | COL4A6 | -73.93034147 | -123.3558506 | 10 |
| 42 | NR3C1 | AR | -73.72738905 | -289.2367706 | 25 |
| 43 | NR3C1 | ESR1 | -73.72738905 | -292.7791161 | 25 |
| 44 | SUMO1 | UBE2I | -73.5062065 | -258.2503183 | 20 |
| 45 | COL4A3 | COL4A1 | -73.22107635 | -151.9508426 | 12 |
| 46 | DLG4 | DLG3 | -73.17886306 | -232.2327749 | 17 |
| 47 | HDAC1 | SIN3A | -72.47932222 | -266.6637009 | 21 |
| 48 | IRS2 | IRS1 | -71.88001114 | -151.8096496 | 14 |
| 49 | COL4A1 | COL4A4 | -70.72591947 | -141.9058259 | 11 |
| 50 | IKBKB | CHUK | -69.96305423 | -162.9136822 | 14 |
| 51 | GADD45B | GADD45G | -69.56739182 | -130.2946982 | 11 |
| 52 | GRB2 | PLCG1 | -69.39340099 | -337.6520463 | 28 |
| 53 | JUP | CTNNB1 | -69.25868254 | -254.9584259 | 19 |
| 54 | PLSCR1 | KRTAP4-12 | -68.91001314 | -329.6901377 | 23 |
| 55 | PTPN11 | SHC1 | -68.90609068 | -272.90666 | 24 |
| 56 | PIK3R1 | SHC1 | -68.82244231 | -294.9077988 | 26 |
| 57 | ESR1 | ESR2 | -68.16817601 | -221.2853544 | 17 |
| 58 | DLG2 | DLG3 | -67.29158995 | -151.719371 | 12 |
| 59 | TBP | GTF2B | -66.86509738 | -191.3382891 | 16 |
| 60 | COL4A2 | COL4A5 | -65.9161763 | -123.3558506 | 10 |
| 61 | COL4A2 | COL4A6 | -65.9161763 | -123.3558506 | 10 |
| 62 | COL4A1 | COL4A5 | -65.9161763 | -123.3558506 | 10 |
| 63 | COL4A1 | COL4A6 | -65.9161763 | -123.3558506 | 10 |
| 64 | SRC | GRB2 | -65.68304701 | -369.6892177 | 31 |
| 65 | AR | ESR1 | -65.36120402 | -294.273143 | 24 |
| 66 | COL4A3 | COL4A5 | -64.93398687 | -123.3558506 | 10 |
| 67 | COL4A3 | COL4A6 | -64.93398687 | -123.3558506 | 10 |
| 68 | RARA | RXRA | -64.11053408 | -215.3482183 | 17 |
| 69 | TRAF3 | TRAF2 | -64.01676383 | -260.0936955 | 19 |
| 70 | DLG4 | DLG2 | -63.98188409 | -202.8927639 | 15 |
| 71 | COL1A1 | COL1A2 | -63.24777014 | -151.5867474 | 12 |
| 72 | TGFBR1 | SMURF1 | -62.87519103 | -423.9212755 | 27 |
| 73 | ORC2L | MCM3 | -62.62470809 | -143.1193491 | 11 |
| 74 | GRB2 | FYN | -62.50403476 | -353.978848 | 30 |
| 75 | FLJ22494 | LNX | -61.76207239 | -191.962379 | 15 |
| 76 | TNFSF13 | TWE-PRIL | -60.6242878 | -105.0219606 | 8 |
| 77 | PSEN1 | PSEN2 | -59.82978047 | -172.4340654 | 12 |
| 78 | SHANK2 | SHANK1 | -59.63303509 | -125.6225476 | 9 |
| 79 | COL4A4 | COL4A5 | -59.6305881 | -113.3108339 | 9 |
| 80 | COL4A4 | COL4A6 | -59.6305881 | -113.3108339 | 9 |
| 81 | GRB2 | CRKL | -59.62441316 | -240.3660842 | 21 |
| 82 | NIF3L1 | LNX | -59.17599865 | -179.0708543 | 15 |
| 83 | KRT15 | DIPA | -59.0531779 | -283.1457656 | 22 |
| 84 | HDAC4 | HDAC5 | -58.97406646 | -131.1471165 | 11 |
| 85 | NCOR1 | NCOR2 | -58.96941997 | -160.9803545 | 14 |
| 86 | GRB2 | EGFR | -58.92264739 | -339.0230987 | 29 |
| 87 | DDIT3 | CEBPG | -57.53762273 | -144.3332532 | 11 |
| 88 | NCOA1 | NCOA2 | -57.49927497 | -148.7111631 | 13 |
| 89 | TGFB1 | TGFB2 | -57.37723082 | -148.0591844 | 11 |
| 90 | TRAF2 | TRAF5 | -57.35284724 | -187.591848 | 14 |
| 91 | PIK3R1 | PLCG1 | -57.07816398 | -234.8728064 | 21 |
| 92 | PTPN6 | VAV1 | -56.80604805 | -182.1499538 | 17 |
| 93 | PTK2 | PXN | -56.6283112 | -199.3110295 | 17 |
| 94 | YWHAE | YWHAB | -56.36045703 | -168.5263529 | 14 |
| 95 | NCOA1 | PPARBP | -56.35164632 | -147.0223305 | 13 |
| 96 | TCEB2 | TCEB1 | -56.22888739 | -116.397614 | 8 |
| 97 | RAC1 | RHOA | -55.93112463 | -243.8942209 | 17 |
| 98 | STX1A | STX4A | -55.66368681 | -170.1849433 | 12 |
| 99 | IGF2 | IGF1 | -55.36089149 | -129.1709752 | 9 |
| 100 | PAG1 | CBL | -55.22086551 | -119.852138 | 12 |
| 101 | YWHAZ | YWHAB | -54.86337875 | -193.8031874 | 15 |
| 102 | SMN2 | DDX20 | -54.80372312 | -123.8406098 | 9 |
| 103 | GNAI2 | GNAI3 | -54.55428285 | -171.4575127 | 12 |
| 104 | MCM7 | MCM2 | -54.4577214 | -155.7905121 | 12 |
| 105 | ESR1 | RXRA | -54.42433382 | -233.5142756 | 19 |
| 106 | SYK | VAV1 | -54.32372654 | -168.6185713 | 15 |
| 107 | SRC | PIK3R1 | -54.26967037 | -271.9053986 | 23 |
| 108 | GNAI1 | GNAO1 | -54.13038371 | -171.8454402 | 12 |
| 109 | RBBP4 | HDAC1 | -53.99133316 | -149.8446323 | 13 |
| 110 | RIBC2 | TU3A | -53.78938962 | -105.218086 | 10 |
| 111 | JAK2 | PTPN11 | -53.6548795 | -198.1082041 | 18 |
| 112 | JUN | JUND | -53.56563793 | -156.5032226 | 13 |
| 113 | ERBB2 | EGFR | -53.37266001 | -194.4930757 | 17 |
| 114 | IL8RB | IL8RA | -53.31826358 | -125.3057676 | 8 |
| 115 | SMN2 | GEMIN5 | -53.31826358 | -107.4878591 | 8 |
| 116 | DIPA | KIAA0980 | -53.21491811 | -248.0511319 | 18 |
| 117 | GRB2 | LCK | -53.08345707 | -258.9204744 | 22 |
| 118 | K-ALPHA-1 | TUBA2 | -53.0093322 | -94.17201147 | 8 |
| 119 | PTPN6 | SHC1 | -52.93501896 | -209.5488701 | 19 |
| 120 | CPNE2 | CPNE1 | -52.61692073 | -105.5499929 | 8 |
| 121 | CPNE2 | CPNE4 | -52.61692073 | -105.5499929 | 8 |
| 122 | INSR | IGF1R | -52.60883246 | -152.7198638 | 13 |
| 123 | CCR5 | CCBP2 | -52.53526685 | -146.9777368 | 10 |
| 124 | CDC27 | CDC16 | -52.21869964 | -96.02430266 | 8 |
| 125 | EGFR | INSR | -52.00018901 | -199.9203353 | 17 |
| 126 | NCOA1 | RELA | -51.4879004 | -181.7950369 | 17 |
| 127 | TGFB3 | TGFB2 | -51.2665869 | -107.9942436 | 8 |
| 128 | ERBB2 | ERBB3 | -51.14620615 | -124.7513678 | 11 |
| 129 | KIAA0408 | RIBC2 | -51.11713523 | -107.8027107 | 10 |
| 130 | PLCG1 | SHC1 | -51.09921926 | -217.1652406 | 19 |
| 131 | NCOR1 | SIN3A | -51.06007248 | -162.6756772 | 13 |
| 132 | TRAF3 | TRAF5 | -51.05966346 | -135.8949254 | 10 |
| 133 | PSMA2 | PLK1 | -50.92646954 | -157.7437897 | 10 |
| 134 | SYK | ZAP70 | -50.85333953 | -147.3483587 | 13 |
| 135 | NCOA1 | NCOA3 | -50.47714922 | -134.0379987 | 12 |
| 136 | JUND | JUNB | -50.379487 | -106.6247075 | 9 |
| 137 | LRP2 | LRP1 | -50.17507279 | -184.9652614 | 14 |
| 138 | TGFB1 | TGFB3 | -50.15242977 | -120.7524255 | 9 |
| 139 | PLSCR1 | MDFI | -50.09653138 | -284.4524951 | 20 |
| 140 | NOTCH1 | NOTCH2 | -50.05417387 | -132.2017988 | 9 |
| 141 | ACTA1 | ACTA2 | -49.92697889 | -121.5887173 | 8 |
| 142 | GEMIN5 | DDX20 | -49.65742117 | -106.2810332 | 8 |
| 143 | PPFIA1 | PPFIA2 | -49.65089455 | -111.7036483 | 8 |
| 144 | NIF3L1 | FLJ22494 | -49.41538383 | -153.1918347 | 12 |
| 145 | RB1 | RBL2 | -49.35799053 | -159.7627593 | 13 |
| 146 | TGFBR1 | SMAD2 | -49.22991515 | -313.6395562 | 23 |
| 147 | PAG1 | PTK2B | -49.13577967 | -106.1032354 | 11 |
| 148 | DLG1 | DLG2 | -49.11758986 | -149.7428191 | 11 |
| 149 | SYK | GRB2 | -48.96440679 | -216.4612199 | 19 |
| 150 | GRB2 | VAV1 | -48.95379374 | -221.5131424 | 20 |
| 151 | GNAI1 | GNAI3 | -48.92562467 | -156.5021062 | 11 |
| 152 | STAT3 | STAT5A | -48.87833789 | -143.7916207 | 13 |
| 153 | GRIN2B | GRIN2A | -48.70997026 | -103.2496301 | 9 |
| 154 | BCAR1 | PXN | -48.65494906 | -144.6837174 | 13 |
| 155 | SNAP23 | SNAP25 | -48.54485032 | -136.1281066 | 10 |
| 156 | DCN | MMP9 | -48.4240181 | -121.169453 | 10 |
| 157 | LSM2 | LSM6 | -48.35487606 | -97.64839637 | 8 |
| 158 | RB1 | RBL1 | -48.21283721 | -171.297408 | 14 |
| 159 | MRAS | RAP2A | -48.13695391 | -92.11190456 | 7 |
| 160 | SYK | CBL | -48.09739775 | -146.6243912 | 14 |
| 161 | RXRA | PPARG | -48.03681755 | -154.551442 | 12 |
| 162 | DCN | TGFBI | -47.93369554 | -95.13917142 | 8 |
| 163 | GNAI2 | GNAO1 | -47.80939626 | -154.1143633 | 11 |
| 164 | MCM3 | MCM7 | -47.73212946 | -132.8259441 | 10 |
| 165 | DCN | C1QR1 | -47.63175485 | -113.3746534 | 9 |
| 166 | CDK4 | CDK6 | -47.39346181 | -117.4577271 | 9 |
| 167 | GADD45A | GADD45G | -47.35497338 | -93.59561967 | 8 |
| 168 | PPARA | PPARG | -47.31448046 | -127.2745442 | 10 |
| 169 | LSM5 | LSM2 | -47.25803203 | -97.64839637 | 8 |
| 170 | NUP214 | NUP153 | -47.25694418 | -100.9688921 | 8 |
| 171 | RIPK1 | TRADD | -47.2113429 | -117.5311184 | 10 |
| 172 | BIRC3 | BIRC2 | -47.12603232 | -83.77493325 | 7 |
| 173 | BATF | FOSL2 | -47.12127462 | -83.25986031 | 7 |
| 174 | GNA12 | GNA13 | -47.01864688 | -138.531382 | 10 |
| 175 | SYK | PIK3R1 | -47.0063129 | -166.6326524 | 16 |
| 176 | PLAT | PLG | -46.96960868 | -133.6613215 | 10 |
| 177 | CDH1 | CTNNB1 | -46.90858967 | -179.2859447 | 14 |
| 178 | GRB2 | NCK1 | -46.90800812 | -231.1999571 | 19 |
| 179 | GRB2 | CBL | -46.9007666 | -229.5719977 | 20 |
| 180 | ORC2L | MCM7 | -46.84619219 | -132.9907331 | 10 |
| 181 | Rps27a | Uhmk1 | -46.84323227 | -54.41756914 | 6 |
| 182 | GRB2 | ZAP70 | -46.84008345 | -188.8756016 | 17 |
| 183 | FLJ32855 | LNX | -46.75233618 | -123.3224073 | 11 |
| 184 | ABLIM1 | TU3A | -46.74843035 | -85.3823523 | 8 |
| 185 | PPFIA2 | PPFIA3 | -46.6321971 | -98.54875399 | 7 |
| 186 | TGFBR1 | SMAD4 | -46.56941628 | -366.3543753 | 25 |
| 187 | RXRA | THRB | -46.41308504 | -165.5102055 | 12 |
| 188 | ACVR2A | ACVR2B | -46.41127861 | -110.1503806 | 8 |
| 189 | LSM3 | LSM5 | -46.40910277 | -97.80254705 | 8 |
| 190 | PDGFRB | EGFR | -46.40310111 | -162.0161989 | 15 |
| 191 | BCAR1 | NEDD9 | -46.24876461 | -116.599624 | 11 |
| 192 | FXR2 | LNX | -46.24552497 | -166.307615 | 14 |
| 193 | GNAI2 | GNAI1 | -46.21961531 | -166.406671 | 12 |
| 194 | TNPO1 | RANBP5 | -46.05025162 | -108.2216242 | 8 |
| 195 | JUN | CEBPB | -46.03232224 | -163.504687 | 15 |
| 196 | LSM4 | LSM5 | -45.64641909 | -97.9456479 | 8 |
| 197 | LSM3 | LSM2 | -45.45440799 | -97.96960114 | 8 |
| 198 | TGFBR3 | ENG | -45.42849633 | -82.7769275 | 7 |
| 199 | PEPP-2 | RBPMS | -45.36250418 | -157.0131503 | 12 |
| 200 | NCOA1 | NRIP1 | -45.25772539 | -131.2515547 | 12 |
| 201 | NDP52 | DIPA | -45.24092977 | -236.7683123 | 18 |
| 202 | NCOA2 | PPARBP | -45.10586824 | -101.2283063 | 9 |
| 203 | SIT1 | PAG1 | -45.03108659 | -68.7234239 | 7 |
| 204 | ORC1L | MCM3 | -44.95708047 | -105.1465529 | 8 |
| 205 | HABP2 | SERPINE2 | -44.89732213 | -76.79095267 | 6 |
| 206 | PTPN11 | CRKL | -44.78490422 | -156.8651716 | 14 |
| 207 | LSM4 | LSM2 | -44.69186048 | -98.11270198 | 8 |
| 208 | HRAS | RAP1A | -44.65606489 | -128.1956568 | 10 |
| 209 | JUN | JUNB | -44.63141161 | -129.3848887 | 11 |
| 210 | TGFBR1 | TGFBR2 | -44.56273839 | -174.3312409 | 14 |
| 211 | PIK3R1 | PIK3R2 | -44.49201549 | -114.1243101 | 11 |
| 212 | PIK3R1 | FYN | -44.2701179 | -232.9912143 | 20 |
| 213 | CCR2 | CCR5 | -44.14390473 | -110.3012698 | 8 |
| 214 | AP2M1 | AP1M1 | -43.94719372 | -117.6375598 | 9 |
| 215 | CDC42 | RHOA | -43.85316884 | -200.1685715 | 14 |
| 216 | LSM3 | LSM4 | -43.84660373 | -98.26685266 | 8 |
| 217 | LSM3 | LSM7 | -43.84660373 | -99.12430289 | 8 |
| 218 | MMP9 | MATN2 | -43.76638644 | -97.93373905 | 8 |
| 219 | EFEMP2 | KRTAP4-12 | -43.76009548 | -178.2902063 | 13 |
| 220 | VAV1 | ZAP70 | -43.73581462 | -136.522431 | 12 |
| 221 | STAT5B | STAT5A | -43.61607016 | -100.7888124 | 9 |
| 222 | Rasd2 | Rps27a | -43.509397 | -54.41756914 | 6 |
| 223 | Rasd2 | Fbxo3 | -43.509397 | -54.65430411 | 6 |
| 224 | Rasd2 | Sqstm1 | -43.509397 | -55.14657866 | 6 |
| 225 | Rasd2 | Map2k3 | -43.509397 | -53.1824134 | 6 |
| 226 | Rasd2 | Uhmk1 | -43.509397 | -54.41756914 | 6 |
| 227 | Rasd2 | Rhod | -43.509397 | -55.7396668 | 6 |
| 228 | DCN | FBLN2 | -43.42110213 | -112.3362812 | 9 |
| 229 | PIK3R1 | LYN | -43.25809006 | -199.2928279 | 17 |
| 230 | PTPN11 | SYK | -43.00462897 | -149.9651422 | 14 |
| 231 | APEX2 | TU3A | -42.95178206 | -71.96030807 | 7 |
| 232 | VLDLR | LRP8 | -42.9507326 | -84.28501423 | 6 |
| 233 | RPP25 | POP5 | -42.9507326 | -87.80362146 | 6 |
| 234 | RPP25 | RPP30 | -42.9507326 | -87.80362146 | 6 |
| 235 | POP5 | RPP30 | -42.9507326 | -87.80362146 | 6 |
| 236 | DCN | MATN2 | -42.83346747 | -97.93373905 | 8 |
| 237 | ACVR1 | SMAD4 | -42.72020889 | -240.1710342 | 17 |
| 238 | GAB2 | SHC1 | -42.6974178 | -112.0751914 | 11 |
| 239 | RXRA | RXRB | -42.68817061 | -130.8295077 | 10 |
| 240 | NCOA1 | RXRA | -42.63524817 | -170.8421429 | 14 |
| 241 | HDAC3 | HDAC2 | -42.61819069 | -158.2345068 | 13 |
| 242 | TBP | TAF1 | -42.59805866 | -137.9181026 | 12 |
| 243 | OSM | HABP2 | -42.40996916 | -76.79095267 | 6 |
| 244 | MAPK8IP1 | MAPK8IP2 | -42.33772675 | -83.34262178 | 7 |
| 245 | BMP2 | BMP7 | -42.33582328 | -89.46033915 | 7 |
| 246 | GADD45B | GADD45A | -42.33582328 | -83.22242956 | 7 |
| 247 | GNAI1 | GNAQ | -42.31927231 | -161.3585908 | 11 |
| 248 | SNX1 | SNX2 | -42.20077893 | -81.12426297 | 7 |
| 249 | ERCC3 | GTF2H5 | -42.07141913 | -100.6980051 | 7 |
| 250 | MAP3K14 | TRAF3IP2 | -42.07141913 | -75.87139392 | 7 |
| 251 | MYOD1 | TCF3 | -41.94784723 | -134.3091363 | 11 |
| 252 | CASP8 | CFLAR | -41.93916853 | -113.6687053 | 9 |
| 253 | PIK3R1 | LCK | -41.91302113 | -184.8567086 | 16 |
| 254 | JAK1 | JAK3 | -41.82955924 | -117.902316 | 10 |
| 255 | DDIT3 | BATF | -41.7367615 | -99.22834816 | 8 |
| 256 | C1QR1 | MMP9 | -41.72314951 | -100.4494174 | 8 |
| 257 | KRT15 | USHBP1 | -41.69024654 | -158.4353022 | 12 |
| 258 | DLG1 | DLG3 | -41.65494862 | -135.4901292 | 10 |
| 259 | E2F2 | E2F3 | -41.56375877 | -78.20255709 | 6 |
| 260 | BMP7 | GDF5 | -41.49286277 | -71.03511552 | 6 |
| 261 | SIP1 | SFRS2IP | -41.49286277 | -77.52434331 | 6 |
| 262 | FN1 | DCN | -41.46084085 | -135.7774582 | 11 |
| 263 | PPFIA1 | PPFIA3 | -41.44213051 | -99.6191954 | 7 |
| 264 | DIPA | HOOK2 | -41.41509206 | -153.9566021 | 12 |
| 265 | SMURF2 | SMURF1 | -41.39619496 | -220.2378639 | 14 |
| 266 | SFRS1 | SFRS2 | -41.38096387 | -112.9666444 | 8 |
| 267 | VAV1 | CBL | -41.35790865 | -138.5413097 | 13 |
| 268 | DNM1 | SYNJ1 | -41.34420166 | -86.97828944 | 7 |
| 269 | SNRPE | SNRPD2 | -41.32490269 | -96.06384734 | 7 |
| 270 | LSM5 | LSM6 | -41.32490269 | -84.37378467 | 7 |
| 271 | ARF1 | ARF6 | -41.30877327 | -111.7981418 | 8 |
| 272 | CASP8 | FADD | -41.29842529 | -131.406081 | 10 |
| 273 | SRC | PLCG1 | -41.21726699 | -189.9434317 | 17 |
| 274 | LYN | LCK | -41.18164688 | -162.2855551 | 14 |
| 275 | SRC | LCK | -41.1732206 | -183.5990962 | 16 |
| 276 | PTK2B | PXN | -41.01939063 | -145.9634317 | 13 |
| 277 | RAP1A | RAP2A | -40.8660863 | -92.39958664 | 7 |
| 278 | DIPA | USHBP1 | -40.78388281 | -195.9356015 | 15 |
| 279 | HABP2 | MATN2 | -40.70766738 | -76.79095267 | 6 |
| 280 | MYOD1 | MYOG | -40.62940142 | -91.27315733 | 8 |
| 281 | PIK3R1 | CRKL | -40.57375767 | -156.0399911 | 14 |
| 282 | SNTA1 | SNTB2 | -40.55130524 | -103.4834277 | 7 |
| 283 | C1QR1 | MATN2 | -40.55130524 | -88.12201829 | 7 |
| 284 | GHR | EPOR | -40.54843776 | -92.54024858 | 9 |
| 285 | RPP25 | RPP38 | -40.46609791 | -88.34261796 | 6 |
| 286 | POP5 | RPP38 | -40.46609791 | -88.34261796 | 6 |
| 287 | RPP30 | RPP38 | -40.46609791 | -90.28852811 | 6 |
| 288 | OSM | SERPINE2 | -40.46446692 | -76.79095267 | 6 |
| 289 | TGFBI | MMP9 | -40.46367871 | -83.64468148 | 7 |
| 290 | PTPN6 | GRB2 | -40.37656558 | -215.1490247 | 19 |
| 291 | HDAC1 | HDAC3 | -40.29652281 | -184.5879805 | 15 |
| 292 | BMPR1A | BMPR2 | -40.23924629 | -93.96468397 | 7 |
| 293 | PXN | NEDD9 | -40.2361581 | -113.3121523 | 11 |
| 294 | NCK2 | NCK1 | -40.20086252 | -134.6177707 | 11 |
| 295 | RGS16 | RIC8 | -40.17773637 | -67.38652981 | 6 |
| 296 | GRB2 | LCP2 | -40.17518002 | -143.5999216 | 13 |
| 297 | ESR1 | PPARA | -40.15293346 | -139.6695433 | 12 |
| 298 | SYK | SHC1 | -40.09195087 | -153.7464221 | 14 |
| 299 | PIK3R1 | SOCS1 | -40.06668637 | -129.5450323 | 12 |
| 300 | SMAD4 | SMURF2 | -40.04433175 | -210.6740511 | 15 |
| 301 | PIK3R1 | PTPN6 | -39.93165164 | -180.291024 | 16 |
| 302 | VAV1 | FYN | -39.88833982 | -164.0926823 | 15 |
| 303 | LSM3 | LSM6 | -39.85775044 | -84.69498943 | 7 |
| 304 | SNX6 | SNX2 | -39.85775044 | -83.82843161 | 7 |
| 305 | CCND3 | CCND2 | -39.84162117 | -90.72353943 | 7 |
| 306 | SRC | PTK2 | -39.82567764 | -172.8450731 | 16 |
| 307 | GRB2 | LYN | -39.74804755 | -222.7113672 | 19 |
| 308 | NRBF2 | CGI-63 | -39.73158422 | -55.92972196 | 5 |
| 309 | MNAT1 | CCNH | -39.67665305 | -85.02417441 | 7 |
| 310 | TRAF5 | TRAF6 | -39.59479632 | -117.835573 | 9 |
| 311 | SHB | SHC1 | -39.57382389 | -121.6037075 | 11 |
| 312 | RASIP1 | RALGDS | -39.39548099 | -76.14064973 | 6 |
| 313 | C1QR1 | HABP2 | -39.38977137 | -76.79095267 | 6 |
| 314 | PDGFRB | PDGFRA | -39.38885079 | -106.2921215 | 9 |
| 315 | CRK | CRKL | -39.36474033 | -131.4257445 | 11 |
| 316 | LSM4 | LSM6 | -39.22873411 | -84.83809028 | 7 |
| 317 | LSM7 | LSM6 | -39.22873411 | -84.83809028 | 7 |
| 318 | TU3A | ZNF638 | -39.22873411 | -73.58197839 | 7 |
| 319 | NCOA1 | TRIP4 | -39.08813372 | -88.31539245 | 9 |
| 320 | VAV1 | LYN | -38.96454176 | -145.5439083 | 13 |
| 321 | VAV1 | ITK | -38.88788498 | -95.53901474 | 9 |
| 322 | PIK3R1 | CBL | -38.87133268 | -159.2201208 | 15 |
| 323 | CHUK | IKBKG | -38.86245192 | -109.922838 | 9 |
| 324 | MPP6 | MPHOSPH6 | -38.82933948 | -89.39471023 | 6 |
| 325 | IL2RB | IL2RG | -38.78349477 | -100.1553455 | 8 |
| 326 | SERPINE2 | MATN2 | -38.76162185 | -76.79095267 | 6 |
| 327 | LSM1 | LSM7 | -38.74995345 | -102.4421187 | 8 |
| 328 | MYOD1 | MYF5 | -38.74757995 | -86.57241694 | 7 |
| 329 | NCOR1 | HDAC1 | -38.6291783 | -153.4759068 | 13 |
| 330 | JUN | ATF2 | -38.55288725 | -122.141834 | 11 |
| 331 | SOCS1 | SOCS3 | -38.48629283 | -92.27214114 | 8 |
| 332 | PTPN6 | CRKL | -38.46424811 | -135.5828818 | 12 |
| 333 | RBBP4 | HDAC2 | -38.45389791 | -103.3187214 | 9 |
| 334 | PTPN11 | SOCS3 | -38.35768563 | -111.7661621 | 10 |
| 335 | LSM5 | LSM7 | -38.35721042 | -85.02041183 | 7 |
| 336 | JAK2 | EPOR | -38.35610561 | -111.6535173 | 11 |
| 337 | BCAR1 | CBL | -38.34818087 | -113.6672268 | 11 |
| 338 | ACVR1B | ACVR2B | -38.21194941 | -95.01677625 | 7 |
| 339 | JAK2 | GHR | -38.19468926 | -101.7545324 | 10 |
| 340 | RB1 | HDAC1 | -38.17580263 | -202.9810522 | 17 |
| 341 | FN1 | C1QR1 | -38.15340304 | -113.3746534 | 9 |
| 342 | KRT19 | KRT15 | -38.10828079 | -120.1304474 | 9 |
| 343 | MCM3 | MCM2 | -38.06647217 | -104.5689186 | 8 |
| 344 | IGF1R | IRS1 | -38.05870836 | -108.2068021 | 10 |
| 345 | PTPN12 | PTK2 | -38.03029447 | -97.40838181 | 9 |
| 346 | ARCN1 | COPE | -37.93982475 | -67.78837801 | 5 |
| 347 | AFTIPHILIN | AP1GBP1 | -37.93982475 | -68.10316201 | 5 |
| 348 | Rab38 | Map2k3 | -37.93982475 | -44.04891418 | 5 |
| 349 | Rhobtb1 | Rps27a | -37.93982475 | -45.96449121 | 5 |
| 350 | Rhobtb1 | Fbxo3 | -37.93982475 | -45.96449121 | 5 |
| 351 | Rhobtb1 | Uhmk1 | -37.93982475 | -45.96449121 | 5 |
| 352 | Rhoj | Rhod | -37.93982475 | -45.28406991 | 5 |
| 353 | Rps27a | Rhoj | -37.93914536 | -45.28406991 | 5 |
| 354 | Uhmk1 | Rhoj | -37.93914536 | -45.28406991 | 5 |
| 355 | ZAP70 | CBL | -37.91376014 | -119.9245798 | 11 |
| 356 | JAK2 | GRB2 | -37.89879538 | -199.208469 | 18 |
| 357 | SMAD2 | SMURF1 | -37.84663713 | -245.4812714 | 16 |
| 358 | VAV1 | SHC1 | -37.80246512 | -146.7413383 | 14 |
| 359 | YWHAH | YWHAB | -37.67115933 | -124.2123049 | 10 |
| 360 | TRAF1 | TRAF5 | -37.66196924 | -104.3042011 | 8 |
| 361 | GHR | IL2RB | -37.6535237 | -81.44727819 | 8 |
| 362 | FBLN2 | MATN2 | -37.62904187 | -86.60267342 | 7 |
| 363 | CCR3 | CCBP2 | -37.58415763 | -102.8772919 | 7 |
| 364 | LSM7 | LSM2 | -37.58225339 | -85.18746592 | 7 |
| 365 | CBX5 | CBX3 | -37.45101917 | -96.06384734 | 7 |
| 366 | MYF5 | TCF4 | -37.44970757 | -71.24928561 | 6 |
| 367 | SNTA1 | SNTB1 | -37.44481328 | -84.86445954 | 6 |
| 368 | C1QR1 | SERPINE2 | -37.44481328 | -76.79095267 | 6 |
| 369 | ORC2L | MCM2 | -37.43143898 | -102.846638 | 8 |
| 370 | TGFBI | MATN2 | -37.37519209 | -71.31728241 | 6 |
| 371 | MEP50 | SMN2 | -37.37519209 | -81.31323519 | 6 |
| 372 | COPG | COPG2 | -37.37274511 | -81.61758432 | 6 |
| 373 | SNX1 | SNX4 | -37.37274511 | -68.86960731 | 6 |
| 374 | SNX2 | SNX4 | -37.37274511 | -67.84650257 | 6 |
| 375 | CDC2 | CCNB1 | -37.36662133 | -104.0034713 | 9 |
| 376 | GRB2 | SOCS1 | -37.32588335 | -151.7299828 | 13 |
| 377 | GAB1 | SHC1 | -37.32014814 | -103.9326095 | 10 |
| 378 | KIT | GAB2 | -37.23916225 | -78.5627139 | 8 |
| 379 | ORC4L | MCM7 | -37.19693349 | -89.75000862 | 7 |
| 380 | DNMT3B | DNMT3A | -37.06367795 | -75.51782783 | 6 |
| 381 | YWHAH | YWHAE | -37.01500475 | -124.0001303 | 10 |
| 382 | NCOA1 | ESR1 | -37.0092193 | -161.0801637 | 14 |
| 383 | FBLN2 | HABP2 | -37.00446152 | -76.79095267 | 6 |
| 384 | WAS | CBL | -36.98082325 | -103.8072983 | 10 |
| 385 | CCR5 | CCR1 | -36.95819592 | -101.3368469 | 7 |
| 386 | SYK | LYN | -36.79947833 | -144.6458661 | 12 |
| 387 | PTPN11 | LYN | -36.79710806 | -165.9931712 | 14 |
| 388 | PTPN11 | VAV1 | -36.71246718 | -137.8453417 | 13 |
| 389 | CORT | SST | -36.68570291 | -77.67344698 | 5 |
| 390 | GAB3 | CSF1R | -36.68231702 | -62.32889307 | 6 |
| 391 | HLA-A | HLA-B | -36.67905416 | -81.31974988 | 6 |
| 392 | BATF | ATF3 | -36.67905416 | -69.65580396 | 6 |
| 393 | GNAI3 | GNAO1 | -36.66530744 | -113.2407623 | 8 |
| 394 | THRAP4 | PPARBP | -36.63476315 | -65.60952835 | 6 |
| 395 | KIT | EPOR | -36.63165599 | -95.0663538 | 9 |
| 396 | XRCC5 | XRCC6 | -36.59489555 | -94.9311125 | 8 |
| 397 | IL6ST | LIFR | -36.59474601 | -95.92593859 | 7 |
| 398 | BCL3 | TRIP4 | -36.47599033 | -71.03108651 | 7 |
| 399 | JAK1 | IL2RB | -36.39720697 | -94.26662965 | 9 |
| 400 | JAK2 | PTPN6 | -36.33127453 | -145.5425149 | 13 |
| 401 | PDGFRB | INSR | -36.33042752 | -118.0134758 | 10 |
| 402 | HIST3H3 | HIST2H2BE | -36.31673665 | -97.46379232 | 7 |
| 403 | JAK1 | VAV1 | -36.30137608 | -119.24112 | 11 |
| 404 | OSM | MATN2 | -36.27644439 | -76.79095267 | 6 |
| 405 | MMP9 | HABP2 | -36.26666352 | -76.79095267 | 6 |
| 406 | GAB1 | GAB2 | -36.26267605 | -72.22069688 | 7 |
| 407 | LSM4 | LSM7 | -36.26267605 | -85.48471744 | 7 |
| 408 | TRAF2 | TRAF6 | -36.20107542 | -197.1283011 | 15 |
| 409 | PTK2B | CRKL | -36.19897285 | -110.8158048 | 11 |
| 410 | PTPN11 | SRC | -36.196943 | -173.2372823 | 16 |
| 411 | IGSF1 | INHBC | -36.14752183 | -61.74378984 | 5 |
| 412 | ARPC3 | ARPC2 | -36.14752183 | -71.70218514 | 5 |
| 413 | ARPC3 | ARPC4 | -36.14752183 | -71.70218514 | 5 |
| 414 | ARPC2 | ARPC4 | -36.14752183 | -71.70218514 | 5 |
| 415 | Rps27a | Fbxo3 | -36.14752183 | -45.96449121 | 5 |
| 416 | Rps27a | Sqstm1 | -36.14752183 | -46.45676576 | 5 |
| 417 | Rps27a | Map2k3 | -36.14752183 | -44.4926005 | 5 |
| 418 | Rps27a | Rhod | -36.14752183 | -45.28406991 | 5 |
| 419 | Fbxo3 | Sqstm1 | -36.14752183 | -46.69350074 | 5 |
| 420 | Fbxo3 | Map2k3 | -36.14752183 | -44.72933548 | 5 |
| 421 | Fbxo3 | Uhmk1 | -36.14752183 | -45.96449121 | 5 |
| 422 | Sqstm1 | Map2k3 | -36.14752183 | -45.22161003 | 5 |
| 423 | Sqstm1 | Uhmk1 | -36.14752183 | -46.45676576 | 5 |
| 424 | Map2k3 | Uhmk1 | -36.14752183 | -44.4926005 | 5 |
| 425 | Uhmk1 | Rhod | -36.14752183 | -45.28406991 | 5 |
| 426 | FYN | CRKL | -36.09941856 | -144.5021595 | 13 |
| 427 | PTK2 | PDGFRB | -36.09474132 | -104.3050692 | 11 |
| 428 | TGFBI | C1QR1 | -36.06354996 | -73.83296072 | 6 |
| 429 | SYK | PLCG1 | -35.94622955 | -125.7579613 | 12 |
| 430 | TRAF2 | DIPA | -35.90885608 | -283.1926128 | 22 |
| 431 | GTF2E2 | GTF2E1 | -35.7842048 | -85.02246909 | 7 |
| 432 | ESR1 | HNF4A | -35.76439277 | -119.2556816 | 10 |
| 433 | IKBKB | RIPK1 | -35.73511568 | -90.29451606 | 8 |
| 434 | CDC2 | CDK2 | -35.72524914 | -125.8585826 | 10 |
| 435 | PARD6G | PARD6A | -35.70623253 | -57.17807582 | 5 |
| 436 | TNFRSF19 | TNFRSF11A | -35.70623253 | -53.47486974 | 5 |
| 437 | BAK1 | PMAIP1 | -35.70419407 | -60.61054889 | 5 |
| 438 | TFDP1 | TFDP2 | -35.70419407 | -66.5474495 | 5 |
| 439 | Rasd2 | Rab38 | -35.70419407 | -44.04891418 | 5 |
| 440 | Rasd2 | Rhobtb1 | -35.70419407 | -45.96449121 | 5 |
| 441 | Rasd2 | Rhoj | -35.70419407 | -45.28406991 | 5 |
| 442 | Rasd2 | Smad3 | -35.70419407 | -45.86548616 | 5 |
| 443 | Rasd2 | Rras2 | -35.70419407 | -47.3351771 | 5 |
| 444 | Rasd2 | Stat1 | -35.70419407 | -45.92794604 | 5 |
| 445 | COL4A2 | COL1A2 | -35.68840138 | -82.69612751 | 7 |
| 446 | VIL2 | MSN | -35.68291003 | -106.6973835 | 8 |
| 447 | PTPRC | PAG1 | -35.68291003 | -79.63800386 | 8 |
| 448 | DCN | HABP2 | -35.61010368 | -76.79095267 | 6 |
| 449 | PTPN6 | LYN | -35.58471818 | -149.3415021 | 13 |
| 450 | RELA | JUN | -35.582208 | -156.1129099 | 15 |
| 451 | INHBA | INHBB | -35.58125809 | -78.6355371 | 6 |
| 452 | GRB2 | INSR | -35.57112982 | -171.0766973 | 15 |
| 453 | PIK3R1 | PTK2B | -35.53017551 | -135.8205498 | 14 |
| 454 | RAD9A | HUS1 | -35.49931139 | -75.89204162 | 6 |
| 455 | JAK1 | PTPN11 | -35.48636942 | -131.213017 | 12 |
| 456 | CD22 | CBL | -35.43098247 | -77.42968386 | 8 |
| 457 | CCND1 | CCND2 | -35.41841277 | -90.72353943 | 7 |
| 458 | YWHAH | SMARCE1 | -35.41841277 | -72.80221266 | 7 |
| 459 | NFKB1 | NFKB2 | -35.38845238 | -101.7668362 | 8 |
| 460 | DNM1 | WASL | -35.29172923 | -85.57242498 | 7 |
| 461 | IKBKB | IKBKG | -35.28503947 | -97.95715952 | 8 |
| 462 | SMAD4 | SMURF1 | -35.19121876 | -263.1069878 | 17 |
| 463 | RXRA | PPARA | -35.1706672 | -124.9050345 | 10 |
| 464 | LYN | HCK | -35.13675288 | -127.3651279 | 10 |
| 465 | CASP3 | CASP7 | -35.07561256 | -107.0499034 | 8 |
| 466 | FBLN2 | SERPINE2 | -35.06018402 | -76.79095267 | 6 |
| 467 | GRB2 | GRAP2 | -35.03829526 | -159.0576748 | 13 |
| 468 | RARA | ESR1 | -35.0124807 | -144.9254486 | 12 |
| 469 | MEP50 | DDX20 | -34.99365295 | -81.31323519 | 6 |
| 470 | GAB2 | GAB3 | -34.98794177 | -55.64576093 | 6 |
| 471 | ACVR1B | INHBB | -34.96181113 | -77.19315327 | 6 |
| 472 | C1QR1 | OSM | -34.96181113 | -76.79095267 | 6 |
| 473 | DAZAP2 | RNF11 | -34.9335871 | -126.1961635 | 9 |
| 474 | CCL8 | CCL7 | -34.89489481 | -63.50804216 | 5 |
| 475 | ARHGEF12 | ARHGEF11 | -34.89489481 | -63.18994706 | 5 |
| 476 | LILRB2 | LILRB1 | -34.89421532 | -68.16645686 | 5 |
| 477 | GGA3 | GGA1 | -34.89421532 | -68.53818941 | 5 |
| 478 | TOB1 | ZNF8 | -34.89258419 | -48.97444407 | 5 |
| 479 | USP7 | TNFRSF19 | -34.89258419 | -53.47486974 | 5 |
| 480 | PIK3R1 | ZAP70 | -34.77856073 | -128.0593263 | 12 |
| 481 | RAF1 | BRAF | -34.72406764 | -92.8809925 | 8 |
| 482 | KIAA0980 | HOOK2 | -34.71540429 | -102.1030953 | 8 |
| 483 | JAK2 | SHC1 | -34.6932362 | -154.7227013 | 14 |
| 484 | PECAM1 | TRPV4 | -34.68001391 | -60.94693657 | 6 |
| 485 | BLNK | CBLB | -34.66747603 | -73.09234363 | 7 |
| 486 | TF | IGF1 | -34.66741533 | -88.94045496 | 6 |
| 487 | KIAA0408 | TU3A | -34.66461869 | -72.34650036 | 7 |
| 488 | PTPN6 | PLCG1 | -34.65915131 | -139.2295527 | 13 |
| 489 | CSK | FYN | -34.57068818 | -126.9290328 | 11 |
| 490 | PLCG1 | FYN | -34.56321283 | -168.0280133 | 15 |
| 491 | ZBTB16 | HDAC3 | -34.52712025 | -127.3560678 | 11 |
| 492 | RAP1A | MRAS | -34.51739407 | -78.32921827 | 6 |
| 493 | SMAD2 | SMAD1 | -34.39386332 | -145.4455218 | 12 |
| 494 | FN1 | COL1A1 | -34.38279739 | -131.4316458 | 10 |
| 495 | SIN3A | HDAC9 | -34.38188955 | -90.63924088 | 8 |
| 496 | RB1 | SP1 | -34.38086977 | -160.3971522 | 14 |
| 497 | SLC9A3R1 | SLC9A3R2 | -34.3603185 | -111.7973943 | 8 |
| 498 | SRC | RASA1 | -34.34859508 | -143.5616914 | 13 |
| 499 | MMP9 | SERPINE2 | -34.32265839 | -76.79095267 | 6 |
| 500 | BGN | DCN | -34.31263744 | -85.13127648 | 7 |
| 501 | CCR5 | CCR3 | -34.30406426 | -102.8772919 | 7 |
| 502 | PECAM1 | CBL | -34.29840109 | -88.24072021 | 9 |
| 503 | POU2F1 | POU2F2 | -34.22793856 | -69.96311341 | 6 |
| 504 | BLNK | LCP2 | -34.20903362 | -78.37354623 | 7 |
| 505 | TBL1X | TBL1XR1 | -34.19875724 | -62.58438979 | 5 |
| 506 | CDC5L | CCDC5 | -34.19875724 | -60.05789863 | 5 |
| 507 | RAC1 | RAC2 | -34.11034585 | -112.3971561 | 8 |
| 508 | S100A1 | S100B | -34.0926579 | -102.3084425 | 7 |
| 509 | ORC4L | ORC2L | -34.0888758 | -76.25991841 | 6 |
| 510 | EGFR | ERBB3 | -34.07603135 | -121.7110921 | 10 |
| 511 | CCND1 | CCND3 | -34.06321245 | -100.0779632 | 8 |
| 512 | FOS | CEBPB | -34.05116493 | -114.8295819 | 10 |
| 513 | CCR3 | CCR1 | -34.04647284 | -89.34592007 | 6 |
| 514 | PTPN11 | SOCS1 | -34.01472826 | -103.2900617 | 10 |
| 515 | MLLT4 | RASIP1 | -33.98595884 | -76.14064973 | 6 |
| 516 | NEDD4 | NEDD4L | -33.98550548 | -83.6034998 | 6 |
| 517 | ACVR1 | ACVR2A | -33.95679771 | -109.0517684 | 8 |
| 518 | LYN | CRKL | -33.9558387 | -125.7169321 | 11 |
| 519 | MAPK1 | MAPK14 | -33.92044979 | -160.6026348 | 11 |
| 520 | ORC4L | ORC5L | -33.91284246 | -61.96298679 | 5 |
| 521 | HUS1 | RAD1 | -33.91284246 | -71.1701573 | 5 |
| 522 | SCNN1B | SCNN1G | -33.91284246 | -66.6742012 | 5 |
| 523 | MAP2K3 | MAP2K6 | -33.91284246 | -67.35821776 | 5 |
| 524 | ZNFN1A1 | ZNFN1A4 | -33.89055406 | -75.36823352 | 6 |
| 525 | MBD2 | MBD3 | -33.87814276 | -85.45156742 | 6 |
| 526 | NUP153 | RANBP2 | -33.87814276 | -73.80201592 | 6 |
| 527 | FXR2 | FLJ32855 | -33.87642856 | -98.99838339 | 9 |
| 528 | GRB2 | PTK2B | -33.83177756 | -160.7430491 | 16 |
| 529 | GHR | IGF1R | -33.82987341 | -88.56633783 | 8 |
| 530 | HDAC4 | HDAC9 | -33.77457789 | -81.91910026 | 7 |
| 531 | YWHAZ | YWHAQ | -33.69046478 | -129.652023 | 9 |
| 532 | DCN | SERPINE2 | -33.66637099 | -76.79095267 | 6 |
| 533 | NCOA1 | JUN | -33.65774353 | -140.5353335 | 13 |
| 534 | CREBBP | JUN | -33.63178649 | -165.8707259 | 15 |
| 535 | CBX1 | CBX5 | -33.60652711 | -93.69535104 | 7 |
| 536 | PTPN12 | PTK2B | -33.5598132 | -85.8268805 | 8 |
| 537 | ESR1 | PPARG | -33.54224535 | -124.295377 | 10 |
| 538 | APP | C1QR1 | -33.52055199 | -102.9786486 | 8 |
| 539 | ZAP70 | LCP2 | -33.51980547 | -83.83132016 | 8 |
| 540 | PIK3R1 | PTK2 | -33.45295382 | -143.4695103 | 14 |
| 541 | PTPN11 | CSK | -33.45225403 | -107.972887 | 10 |
| 542 | NCOR2 | HDAC3 | -33.43088621 | -123.7613897 | 10 |
| 543 | DIPA | MDFI | -33.41648469 | -275.3632229 | 19 |
| 544 | LAT | PLCG1 | -33.39324909 | -104.2851291 | 9 |
| 545 | SMARCA4 | SMARCA2 | -33.36340926 | -73.89034773 | 6 |
| 546 | BCL6 | ZBTB16 | -33.29316751 | -92.97564178 | 8 |
| 547 | NCK1 | CRKL | -33.27477166 | -115.4856006 | 10 |
| 548 | JAK2 | SYK | -33.25824935 | -111.7408158 | 11 |
| 549 | RARA | THRA | -33.23045779 | -98.03845869 | 8 |
| 550 | CCNA2 | CCNE1 | -33.20098475 | -86.19745052 | 7 |
| 551 | PTK2B | PDGFRB | -33.18243816 | -94.18335239 | 10 |
| 552 | JAK2 | TYK2 | -33.15466294 | -97.43908829 | 9 |
| 553 | GRB2 | PLCG2 | -33.15358051 | -133.4569799 | 11 |
| 554 | LAT | LCP2 | -33.104406 | -78.46792829 | 7 |
| 555 | INHBC | INHBB | -33.1034073 | -61.13765404 | 5 |
| 556 | TNFRSF10A | TNFRSF10B | -33.10136856 | -60.52826152 | 5 |
| 557 | REM1 | CDC25B | -33.05702283 | -54.28692389 | 5 |
| 558 | RABEP1 | AFTIPHILIN | -33.05226513 | -68.10316201 | 5 |
| 559 | DIPA | KRTAP4-12 | -33.02838293 | -233.4692543 | 17 |
| 560 | GRB2 | ERBB2 | -33.02799709 | -150.9252139 | 14 |
| 561 | PIK3R1 | TYK2 | -32.99424352 | -107.4069855 | 10 |
| 562 | APEX2 | RIBC2 | -32.94806323 | -61.83105909 | 6 |
| 563 | LCK | ZAP70 | -32.90135464 | -116.0301879 | 10 |
| 564 | COL2A1 | COL1A1 | -32.87620443 | -100.3510895 | 8 |
| 565 | SYK | PTPN6 | -32.86554283 | -118.7756582 | 11 |
| 566 | RIPK1 | RIPK2 | -32.79579325 | -77.69960059 | 7 |
| 567 | SHC1 | CBL | -32.75931493 | -144.5900294 | 13 |
| 568 | FN1 | MMP9 | -32.73817565 | -111.3577322 | 9 |
| 569 | GNAS | GNAQ | -32.73806062 | -113.6733988 | 8 |
| 570 | SIN3A | HDAC2 | -32.71826037 | -120.9104291 | 10 |
| 571 | YWHAZ | YWHAH | -32.6937727 | -130.1203729 | 10 |
| 572 | ATF3 | FOSL2 | -32.66048741 | -57.88802068 | 5 |
| 573 | SMN1 | LSM2 | -32.65337462 | -87.60111767 | 7 |
| 574 | FYN | LCP2 | -32.6525298 | -113.9976768 | 10 |
| 575 | KRT15 | NDP52 | -32.6442493 | -143.3894736 | 11 |
| 576 | BMPR1B | SMURF1 | -32.63634858 | -194.6474246 | 13 |
| 577 | NCOA3 | NCOA2 | -32.61165933 | -81.32546031 | 7 |
| 578 | NCOA1 | TIF1 | -32.59360525 | -97.73605227 | 9 |
| 579 | SHC1 | STAT5A | -32.59325557 | -115.735683 | 11 |
| 580 | OSM | FBLN2 | -32.58588828 | -76.79095267 | 6 |
| 581 | NUP98 | NUP153 | -32.56677358 | -76.40998266 | 6 |
| 582 | CD40 | TNFRSF19 | -32.56607728 | -53.47486974 | 5 |
| 583 | CD4 | LCK | -32.55970867 | -122.5366485 | 10 |
| 584 | FN1 | TGFBI | -32.55353135 | -85.32745066 | 7 |
| 585 | NOTCH1 | NOTCH3 | -32.53688296 | -85.4482858 | 6 |
| 586 | CD9 | CD81 | -32.5052127 | -82.62493258 | 6 |
| 587 | NCOA1 | PPARGC1A | -32.50422281 | -94.50659117 | 8 |
| 588 | ZBTB16 | NRIP1 | -32.49577075 | -87.90110711 | 9 |
| 589 | EMP3 | KCNQ2 | -32.4374787 | -47.02795198 | 4 |
| 590 | CCL11 | CCL13 | -32.4374787 | -50.19981384 | 4 |
| 591 | RPP14 | RPP21 | -32.4374787 | -55.74247697 | 4 |
| 592 | Arhgef7 | Trim35 | -32.4374787 | -35.40988928 | 4 |
| 593 | Pftk1 | Nat5 | -32.4374787 | -36.83099199 | 4 |
| 594 | MAFF | MAFG | -32.41039613 | -72.91127304 | 5 |
| 595 | BMP7 | BMP6 | -32.40767763 | -60.47934284 | 5 |
| 596 | TNFRSF14 | TNFRSF17 | -32.40767763 | -58.12704277 | 5 |
| 597 | SYK | PLCG2 | -32.37197104 | -95.18028599 | 8 |
| 598 | MLLT4 | RALGDS | -32.365557 | -92.15692824 | 7 |
| 599 | RASA1 | SHC1 | -32.28121773 | -125.3254865 | 12 |
| 600 | PTPN11 | PLCG1 | -32.20979568 | -141.9619983 | 13 |
| 601 | MAP2K4 | MAP2K7 | -32.18825561 | -79.73741389 | 6 |
| 602 | TU3A | KIAA1267 | -32.18825561 | -58.04879702 | 6 |
| 603 | MMP9 | FBLN2 | -32.18142273 | -86.60267342 | 7 |
| 604 | RARA | PPARG | -32.18018211 | -95.65850212 | 8 |
| 605 | STAT3 | STAT1 | -32.15265248 | -113.9395696 | 10 |
| 606 | PTK2B | CBL | -32.14192738 | -103.8014498 | 11 |
| 607 | BCL6 | HDAC3 | -32.13667985 | -92.38746555 | 8 |
| 608 | HSPA5 | CANX | -32.12555011 | -112.4662423 | 8 |
| 609 | PLCG1 | LCP2 | -32.01650369 | -100.9770405 | 9 |
| 610 | BCAR1 | CRK | -32.00183808 | -104.8603711 | 9 |
| 611 | ORC4L | MCM2 | -31.99167383 | -74.39574636 | 6 |
| 612 | CHUK | MAP3K14 | -31.96295261 | -83.05833272 | 7 |
| 613 | CSF3R | PTK2B | -31.95792521 | -66.47528197 | 7 |
| 614 | HNF4A | THRB | -31.88925458 | -87.63468639 | 7 |
| 615 | OSM | MMP9 | -31.85054094 | -76.79095267 | 6 |
| 616 | TLR2 | TLR4 | -31.85050877 | -74.22758315 | 5 |
| 617 | SMAD7 | SMAD6 | -31.84156256 | -79.39663943 | 6 |
| 618 | ADAM15 | CBL | -31.80737585 | -70.1869827 | 7 |
| 619 | MAP1A | CRIPT | -31.800998 | -54.98866917 | 5 |
| 620 | BLNK | CBL | -31.78599342 | -84.86274172 | 8 |
| 621 | PTPN11 | GRB10 | -31.77781584 | -95.46808621 | 9 |
| 622 | CANX | CALR | -31.77660889 | -103.5384027 | 8 |
| 623 | RBBP7 | RBBP4 | -31.72421716 | -57.07161006 | 5 |
| 624 | KCNJ12 | ATP2B4 | -31.71741906 | -54.58546523 | 5 |
| 625 | MCM10 | MCM3 | -31.70587608 | -91.39203747 | 7 |
| 626 | CASP8 | RIPK1 | -31.70415659 | -103.5002758 | 8 |
| 627 | AFAP | HABP4 | -31.68006612 | -57.53713921 | 5 |
| 628 | SNRPD3 | SNRPD1 | -31.68006612 | -66.19077455 | 5 |
| 629 | PTK2B | LCK | -31.67173124 | -115.2096598 | 11 |
| 630 | FBLN2 | FBLN1 | -31.66388593 | -73.54489411 | 6 |
| 631 | GRB2 | CRK | -31.6597022 | -167.808353 | 14 |
| 632 | COL4A2 | COL1A1 | -31.64751933 | -85.75866846 | 7 |
| 633 | COL4A1 | COL1A1 | -31.64751933 | -86.78317277 | 7 |
| 634 | VIM | KRT15 | -31.63073262 | -126.2457415 | 10 |
| 635 | TRAF2 | IKBKG | -31.63064588 | -138.4271118 | 11 |
| 636 | BRCA1 | RB1 | -31.61476877 | -156.3847421 | 14 |
| 637 | PTK2B | FYN | -31.59120717 | -140.520247 | 13 |
| 638 | JAK2 | VAV1 | -31.54245229 | -116.4323945 | 11 |
| 639 | TRAF1 | TRAF6 | -31.49650211 | -111.9558318 | 9 |
| 640 | PTPN11 | EPOR | -31.48659412 | -105.5320496 | 10 |
| 641 | PIK3R1 | EGFR | -31.47494251 | -162.1650224 | 16 |
| 642 | GRB2 | PTK2 | -31.44723677 | -166.9434391 | 16 |
| 643 | PTPN11 | CRK | -31.42206663 | -117.0050637 | 11 |
| 644 | PRKAR2A | PRKAR2B | -31.41622061 | -88.78857443 | 6 |
| 645 | TNFAIP3 | MAP3K14 | -31.39002439 | -64.21578463 | 6 |
| 646 | SOS1 | PDGFRB | -31.38561486 | -78.17246333 | 8 |
| 647 | REM1 | BAD | -31.34952371 | -54.28692389 | 5 |
| 648 | EPOR | PAG1 | -31.33706949 | -66.01767275 | 7 |
| 649 | PTPRC | CD5 | -31.32026466 | -75.49957905 | 7 |
| 650 | JAK3 | IL2RB | -31.29465117 | -77.59721877 | 7 |
| 651 | CD82 | CD81 | -31.28412446 | -80.29480992 | 6 |
| 652 | IGSF1 | INHBA | -31.2655359 | -61.74378984 | 5 |
| 653 | INHBC | INHBA | -31.2655359 | -61.74378984 | 5 |
| 654 | CCL5 | CCL8 | -31.26145759 | -62.40204976 | 5 |
| 655 | RABEP1 | AP1GBP1 | -31.26145759 | -68.10316201 | 5 |
| 656 | JAK1 | STAT3 | -31.23779968 | -114.540012 | 10 |
| 657 | FYN | CBL | -31.22107532 | -147.1857299 | 13 |
| 658 | PIK3R1 | RASA1 | -31.19756881 | -138.9056496 | 12 |
| 659 | PTK2 | SHC1 | -31.19518894 | -134.7707299 | 13 |
| 660 | DCN | OSM | -31.18582016 | -76.79095267 | 6 |
| 661 | HOXA1 | CATSPER1 | -31.15559461 | -54.05257076 | 5 |
| 662 | NOTCH2 | NOTCH3 | -31.15559461 | -73.86678449 | 5 |
| 663 | PTPN11 | TYK2 | -31.14416544 | -97.9043831 | 9 |
| 664 | PRKDC | XRCC6 | -31.12227761 | -95.81309889 | 8 |
| 665 | SOS1 | CBLB | -31.03169926 | -70.98504121 | 7 |
| 666 | RBMX | KHDRBS3 | -31.02959518 | -75.51382838 | 6 |
| 667 | BIRC2 | BIRC4 | -31.02959518 | -79.79049898 | 6 |
| 668 | FGF1 | FGF2 | -31.02959518 | -79.49214238 | 6 |
| 669 | BAD | BAX | -31.02633054 | -75.6748186 | 6 |
| 670 | LMO2 | LMO1 | -30.99305818 | -74.08992804 | 5 |
| 671 | SIN3A | SIN3B | -30.97066433 | -84.69341608 | 7 |
| 672 | SFRS1 | U2AF2 | -30.93942936 | -100.3646354 | 7 |
| 673 | TIF1 | PPARBP | -30.93657046 | -75.10238772 | 7 |
| 674 | DLG4 | GRIN2B | -30.93432139 | -114.6437909 | 9 |
| 675 | DVL2 | FXR2 | -30.8953341 | -116.6308025 | 10 |
| 676 | COPG | COPB | -30.88277562 | -80.97005152 | 6 |
| 677 | RBBP4 | DNMT1 | -30.87851954 | -66.54218575 | 6 |
| 678 | RASA1 | FYN | -30.87845917 | -134.937216 | 12 |
| 679 | IL16 | INADL | -30.87706211 | -88.44059837 | 6 |
| 680 | BCL2A1 | MCL1 | -30.86954393 | -70.96251794 | 5 |
| 681 | HLA-B | HLA-G | -30.86954393 | -68.39451381 | 5 |
| 682 | MEP50 | GEMIN5 | -30.86954393 | -66.90639459 | 5 |
| 683 | RGS16 | RGS19 | -30.86954393 | -61.16342782 | 5 |
| 684 | LIN7B | LIN7A | -30.86954393 | -57.12851243 | 5 |
| 685 | PER3 | PER1 | -30.86886426 | -70.13323858 | 5 |
| 686 | USP7 | TNFRSF11A | -30.86886426 | -53.47486974 | 5 |
| 687 | CASP8 | CASP10 | -30.85105869 | -94.78382841 | 7 |
| 688 | GLI1 | GLI2 | -30.82804079 | -58.93718969 | 4 |
| 689 | Rhebl1 | Rhobtb1 | -30.82804079 | -36.83099199 | 4 |
| 690 | Rhebl1 | Rhoj | -30.82804079 | -36.83099199 | 4 |
| 691 | Arhgef7 | Smad3 | -30.82804079 | -35.40988928 | 4 |
| 692 | 1200013B22Rik | Rhoj | -30.82804079 | -35.47234916 | 4 |
| 693 | Trim35 | Smad3 | -30.82804079 | -35.40988928 | 4 |
| 694 | Smad2 | Smad3 | -30.82804079 | -36.73198694 | 4 |
| 695 | KCNQ5 | EMP3 | -30.82749731 | -47.02795198 | 4 |
| 696 | KCNQ5 | KCNQ2 | -30.82749731 | -47.02795198 | 4 |
| 697 | ZNF8 | ZFHX1B | -30.82749731 | -40.52136615 | 4 |
| 698 | PTCH | PTCH2 | -30.82749731 | -67.36095093 | 4 |
| 699 | POP1 | RPP14 | -30.82749731 | -55.74247697 | 4 |
| 700 | POP1 | RPP21 | -30.82749731 | -55.74247697 | 4 |
| 701 | Rab38 | Arhgef6 | -30.82749731 | -35.59583626 | 4 |
| 702 | Rhobtb1 | Pftk1 | -30.82749731 | -36.83099199 | 4 |
| 703 | Rhobtb1 | Nat5 | -30.82749731 | -36.83099199 | 4 |
| 704 | Rhoj | Ilkap | -30.82749731 | -36.15057069 | 4 |
| 705 | JAK2 | PTK2B | -30.77981862 | -108.2485729 | 11 |
| 706 | ACVR1B | INHBC | -30.7754058 | -61.91084393 | 5 |
| 707 | GGA2 | GGA1 | -30.7754058 | -72.97296661 | 5 |
| 708 | SYK | PTK2B | -30.66180183 | -95.26766814 | 10 |
| 709 | E2F1 | E2F2 | -30.65974521 | -78.48187091 | 6 |
| 710 | GRB2 | BLNK | -30.65670975 | -120.3327043 | 10 |
| 711 | TCF4 | TCF3 | -30.64875469 | -83.94407337 | 7 |
| 712 | EPOR | PTK2B | -30.60277689 | -84.59039467 | 9 |
| 713 | RPA2 | RPA1 | -30.59509422 | -79.54375183 | 6 |
| 714 | ORC1L | ORC2L | -30.59509422 | -77.83249473 | 6 |
| 715 | PTPN11 | PTPN12 | -30.59238456 | -78.21043474 | 8 |
| 716 | IRS2 | VAV3 | -30.59101314 | -56.64860035 | 6 |
| 717 | CEBPG | BATF | -30.59101314 | -77.28930497 | 6 |
| 718 | PML | JUN | -30.59048666 | -127.5630773 | 12 |
| 719 | EP300 | JUN | -30.58288398 | -154.1086094 | 14 |
| 720 | ITK | PLCG1 | -30.57818452 | -86.38077167 | 8 |
| 721 | TNFRSF1A | TRPC4AP | -30.57769724 | -66.07590436 | 6 |
| 722 | IPO7 | RANBP5 | -30.55176989 | -71.48477226 | 5 |
| 723 | STAM | STAM2 | -30.55176989 | -60.90738284 | 5 |
| 724 | SNTB2 | SNTB1 | -30.54905102 | -71.55623122 | 5 |
| 725 | SP1 | SP3 | -30.53379597 | -82.46918274 | 7 |
| 726 | ZAP70 | CD3Z | -30.5130943 | -96.66278047 | 8 |
| 727 | PPARG | THRB | -30.50973846 | -87.58117972 | 7 |
| 728 | CD22 | LCP2 | -30.50396706 | -59.23466246 | 6 |
| 729 | PPARBP | NR0B2 | -30.49743689 | -62.16398723 | 6 |
| 730 | FLJ32855 | KIAA1267 | -30.49743689 | -60.12905523 | 6 |
| 731 | FLJ32855 | C16orf48 | -30.49743689 | -57.60455668 | 6 |
| 732 | HSPCA | TEBP | -30.47408992 | -98.7493154 | 8 |
| 733 | EPOR | IGF1R | -30.46509596 | -87.499932 | 8 |
| 734 | RARA | PPARA | -30.42990791 | -99.84815686 | 8 |
| 735 | LCK | CD3Z | -30.39640064 | -112.7030258 | 9 |
| 736 | PTK2 | CRKL | -30.38170107 | -100.8116102 | 10 |
| 737 | NRBF2 | PPARBP | -30.37041296 | -55.92972196 | 5 |
| 738 | CGI-63 | PPARBP | -30.37041296 | -55.92972196 | 5 |
| 739 | PTPN6 | PTK2B | -30.36253445 | -107.3659262 | 11 |
| 740 | VAMP1 | VAMP2 | -30.33846747 | -69.95625593 | 5 |
| 741 | ZNF250 | TU3A | -30.32094537 | -63.78927639 | 6 |
| 742 | FYN | YES1 | -30.26928892 | -111.3274872 | 9 |
| 743 | PAG1 | ZAP70 | -30.26729977 | -67.9525623 | 7 |
| 744 | PTPN11 | PTK2 | -30.24728526 | -121.3937455 | 12 |
| 745 | THBS1 | COL1A1 | -30.22677991 | -97.73549068 | 8 |
| 746 | CD22 | VAV1 | -30.22331259 | -69.70956063 | 7 |
| 747 | BMP7 | BMP4 | -30.1754454 | -66.2271422 | 5 |
| 748 | SNRPB | SNRPD1 | -30.1754454 | -67.28938684 | 5 |
| 749 | SRC | YES1 | -30.16948538 | -117.6526729 | 9 |
| 750 | ABLIM1 | RIBC2 | -30.14797327 | -63.84199498 | 6 |
| 751 | PTPN6 | CBL | -30.12052074 | -114.4451687 | 11 |
| 752 | JAK2 | PIK3R1 | -30.1170636 | -132.7598553 | 13 |
| 753 | DDIT3 | ATF4 | -30.10441242 | -83.51794802 | 7 |
| 754 | STX1A | SNAP23 | -30.0802505 | -113.8608355 | 8 |
| 755 | JAK2 | INSR | -30.07291882 | -113.1983839 | 10 |
| 756 | RELA | RXRA | -30.06364042 | -143.0928839 | 12 |
| 757 | NFKB1 | JUN | -30.05027524 | -131.8268917 | 12 |
| 758 | VAV1 | LCP2 | -30.02714709 | -87.35803829 | 8 |
| 759 | NCOR1 | ZBTB16 | -30.0252852 | -99.81703578 | 9 |
| 760 | PTK2B | SHB | -30.02217216 | -79.34269335 | 8 |
| 761 | LRPAP1 | DAB1 | -30.0130455 | -62.39035372 | 5 |
| 762 | GRB10 | SHC1 | -29.98785821 | -99.95373595 | 9 |
| 763 | USP2 | TU3A | -29.93313847 | -50.42322479 | 5 |
| 764 | AP1G1 | AP1G2 | -29.92701976 | -74.17693941 | 5 |
| 765 | BMPR1B | BMPR2 | -29.85857895 | -96.61389367 | 7 |
| 766 | ACVR1 | SMURF1 | -29.85557579 | -180.8283706 | 12 |
| 767 | PAG1 | PLCG1 | -29.82246951 | -79.60351768 | 8 |
| 768 | USP7 | TRIM37 | -29.8203985 | -66.23305171 | 6 |
| 769 | FYN | NCK1 | -29.81354004 | -139.4346562 | 12 |
| 770 | PAG1 | BCR | -29.81052911 | -55.89621302 | 6 |
| 771 | EPOR | CSF3R | -29.80569961 | -55.91574806 | 6 |
| 772 | PLSCR1 | RBPMS | -29.78827891 | -148.1500656 | 11 |
| 773 | GTF2A1 | TBP | -29.76841192 | -86.51154003 | 7 |
| 774 | SRC | SHC1 | -29.76590365 | -168.7906788 | 15 |
| 775 | EGFR | SHC1 | -29.76590365 | -162.1615187 | 15 |
| 776 | TSC22D4 | EFCBP2 | -29.74677946 | -81.79213535 | 7 |
| 777 | GDF9 | BMP6 | -29.7294285 | -48.50611559 | 4 |
| 778 | Rhebl1 | Rps27a | -29.7294285 | -36.83099199 | 4 |
| 779 | Rhebl1 | Fbxo3 | -29.7294285 | -36.83099199 | 4 |
| 780 | Rhebl1 | Uhmk1 | -29.7294285 | -36.83099199 | 4 |
| 781 | Rhebl1 | Rhod | -29.7294285 | -36.83099199 | 4 |
| 782 | Arhgef7 | Sqstm1 | -29.7294285 | -35.40988928 | 4 |
| 783 | Arhgef7 | Map2k3 | -29.7294285 | -35.40988928 | 4 |
| 784 | 1200013B22Rik | Uhmk1 | -29.7294285 | -35.47234916 | 4 |
| 785 | 1200013B22Rik | Rhod | -29.7294285 | -35.47234916 | 4 |
| 786 | GDF5 | GDF9 | -29.72834147 | -48.50611559 | 4 |
| 787 | CCL8 | CCL11 | -29.72834147 | -50.19981384 | 4 |
| 788 | CCL8 | CCL13 | -29.72834147 | -50.19981384 | 4 |
| 789 | POP7 | RPP14 | -29.72834147 | -55.74247697 | 4 |
| 790 | POP7 | RPP21 | -29.72834147 | -55.74247697 | 4 |
| 791 | POP4 | RPP14 | -29.72834147 | -55.74247697 | 4 |
| 792 | POP4 | RPP21 | -29.72834147 | -55.74247697 | 4 |
| 793 | Rps27a | 1200013B22Rik | -29.72834147 | -35.47234916 | 4 |
| 794 | Rps27a | Ilkap | -29.72834147 | -36.15057069 | 4 |
| 795 | Rps27a | Pftk1 | -29.72834147 | -36.83099199 | 4 |
| 796 | Rps27a | Nat5 | -29.72834147 | -36.83099199 | 4 |
| 797 | Fbxo3 | Arhgef6 | -29.72834147 | -35.59583626 | 4 |
| 798 | Fbxo3 | Pftk1 | -29.72834147 | -36.83099199 | 4 |
| 799 | Fbxo3 | Nat5 | -29.72834147 | -36.83099199 | 4 |
| 800 | Sqstm1 | Trim35 | -29.72834147 | -35.40988928 | 4 |
| 801 | Map2k3 | Arhgef6 | -29.72834147 | -35.59583626 | 4 |
| 802 | Map2k3 | Trim35 | -29.72834147 | -35.40988928 | 4 |
| 803 | Uhmk1 | Ilkap | -29.72834147 | -36.15057069 | 4 |
| 804 | Uhmk1 | Pftk1 | -29.72834147 | -36.83099199 | 4 |
| 805 | Uhmk1 | Nat5 | -29.72834147 | -36.83099199 | 4 |
| 806 | Rhod | Ilkap | -29.72834147 | -36.15057069 | 4 |
| 807 | Rhod | Nkiras1 | -29.72834147 | -38.15308965 | 4 |
| 808 | GTF2A1 | GTF2B | -29.69263171 | -76.96296024 | 6 |
| 809 | YWHAH | YWHAG | -29.67995499 | -85.85211212 | 7 |
| 810 | DIPA | LDOC1 | -29.6567833 | -138.1661495 | 11 |
| 811 | FN1 | HABP2 | -29.62070003 | -76.79095267 | 6 |
| 812 | HDAC1 | SIN3B | -29.58543353 | -97.96864765 | 8 |
| 813 | C1QR1 | FBLN2 | -29.57222511 | -76.79095267 | 6 |
| 814 | CASP7 | CASP9 | -29.57222511 | -81.62883586 | 6 |
| 815 | PRKCABP | SDCBP | -29.57222511 | -85.31041601 | 6 |
| 816 | CSNK1D | CSNK1E | -29.57107716 | -60.77524715 | 5 |
| 817 | SNRPD2 | SNRPD1 | -29.56903787 | -67.28938684 | 5 |
| 818 | GTF2H1 | MNAT1 | -29.56814348 | -66.48111952 | 6 |
| 819 | NCOA1 | NR0B2 | -29.56441359 | -74.76801853 | 7 |
| 820 | SERPINA5 | SERPINB6 | -29.55178211 | -65.91715059 | 5 |
| 821 | PTK2B | SHC1 | -29.55122404 | -118.3624281 | 12 |
| 822 | JAK2 | EGFR | -29.5418775 | -135.6048075 | 13 |
| 823 | POLR2A | BAZ1B | -29.53666482 | -96.3703725 | 7 |
| 824 | CDH15 | CDH2 | -29.52780983 | -62.62390552 | 5 |
| 825 | CD40 | TNFRSF8 | -29.52373097 | -56.69707335 | 5 |
| 826 | PTK2B | PECAM1 | -29.48122689 | -77.0966084 | 8 |
| 827 | PTK2B | BCAR1 | -29.4761638 | -94.4072495 | 9 |
| 828 | RXRA | HNF4A | -29.45798692 | -104.4827955 | 8 |
| 829 | ITK | PLCG2 | -29.41780277 | -66.48780753 | 6 |
| 830 | PTK2B | PLCG1 | -29.40724611 | -105.7825471 | 11 |
| 831 | RELA | SP1 | -29.39197455 | -130.3367837 | 12 |
| 832 | COL4A1 | COL1A2 | -29.34175122 | -69.93794553 | 6 |
| 833 | RELA | MYOD1 | -29.34017324 | -101.8729666 | 10 |
| 834 | PIK3R1 | VAV1 | -29.32182843 | -124.2346563 | 12 |
| 835 | GHR | KIT | -29.29760047 | -69.09055156 | 7 |
| 836 | TYK2 | EPOR | -29.29760047 | -71.47875929 | 7 |
| 837 | JAK2 | KIT | -29.27943552 | -93.34691818 | 9 |
| 838 | DEF6 | ARHGDIA | -29.21819531 | -42.74755273 | 4 |
| 839 | DSC1 | DSC2 | -29.21819531 | -54.41651876 | 4 |
| 840 | ATP2B4 | GUCY1A2 | -29.21819531 | -43.61261183 | 4 |
| 841 | Rab38 | Rhoj | -29.21819531 | -35.35910128 | 4 |
| 842 | Rhobtb1 | Rhoj | -29.21819531 | -36.83099199 | 4 |
| 843 | B2M | LILRB1 | -29.20293115 | -68.16645686 | 5 |
| 844 | TCF3 | ID2 | -29.19535356 | -78.21011612 | 6 |
| 845 | LSM1 | LSM6 | -29.18301252 | -74.46341483 | 6 |
| 846 | PLCG1 | CBL | -29.1448595 | -112.4999166 | 11 |
| 847 | CDC7 | MCM7 | -29.12551869 | -80.01032244 | 6 |
| 848 | BMPR1B | SMAD2 | -29.12328583 | -175.3069097 | 12 |
| 849 | LRPAP1 | APOE | -29.08624926 | -62.39035372 | 5 |
| 850 | MAP3K14 | TRPC4AP | -29.08149027 | -54.49440304 | 5 |
| 851 | SRC | GRAP2 | -29.04477411 | -114.6672196 | 10 |
| 852 | RFC4 | RFC1 | -29.03248885 | -62.09133682 | 5 |
| 853 | BMP2 | BMP4 | -29.02976961 | -66.2271422 | 5 |
| 854 | SNRPE | SNRPD3 | -29.02976961 | -65.48657754 | 5 |
| 855 | PPARGC1A | NCOA2 | -29.01342743 | -61.67688606 | 6 |
| 856 | GTF2H1 | ERCC3 | -29.0101619 | -72.97748307 | 6 |
| 857 | KHDRBS1 | WAS | -29.00092927 | -69.56914187 | 7 |
| 858 | YWHAB | YWHAG | -28.96498241 | -85.85211212 | 7 |
| 859 | PLCG1 | GRAP2 | -28.96453203 | -108.8299725 | 9 |
| 860 | PTPN6 | EPOR | -28.94143404 | -92.89211295 | 9 |
| 861 | SYK | ITK | -28.91871394 | -72.61243706 | 7 |
| 862 | NR3C1 | JUN | -28.9077122 | -140.5444232 | 13 |
| 863 | HDAC9 | HDAC5 | -28.90765905 | -67.84873189 | 6 |
| 864 | MCM10 | MYST2 | -28.89519476 | -69.54711026 | 6 |
| 865 | CCL7 | CCL11 | -28.88049998 | -50.19981384 | 4 |
| 866 | CCL7 | CCL13 | -28.88049998 | -50.19981384 | 4 |
| 867 | STRAP | Wwp2 | -28.88049998 | -37.65198224 | 4 |
| 868 | COPB | COPB2 | -28.8769644 | -67.34822734 | 5 |
| 869 | MAP2K1 | MAP2K2 | -28.8769644 | -57.13037623 | 5 |
| 870 | KIAA0408 | USP2 | -28.8769644 | -50.42322479 | 5 |
| 871 | COL4A3 | COL1A2 | -28.87229329 | -69.93794553 | 6 |
| 872 | FN1 | MATN2 | -28.85352829 | -88.12201829 | 7 |
| 873 | EPOR | CRKL | -28.83777334 | -80.09127891 | 8 |
| 874 | PTPN6 | RASA1 | -28.82583931 | -106.2772981 | 10 |
| 875 | TRAF6 | MAP3K7 | -28.76267001 | -108.2190772 | 8 |
| 876 | WWP2 | WWP1 | -28.75892387 | -62.78592424 | 5 |
| 877 | SRC | ERBB2 | -28.74717966 | -109.6039678 | 11 |
| 878 | EGFR | PDGFRA | -28.74612024 | -103.6483368 | 9 |
| 879 | SRC | LYN | -28.74469116 | -155.2766279 | 13 |
| 880 | ERBB2 | MUC1 | -28.742113 | -68.91182163 | 7 |
| 881 | NCOR1 | HDAC3 | -28.72024739 | -110.4278692 | 9 |
| 882 | BRCA1 | JUN | -28.71278637 | -141.3348215 | 13 |
| 883 | GGA3 | AP1G1 | -28.68105637 | -69.74216221 | 5 |
| 884 | KCNJ12 | KCNJ4 | -28.67561715 | -56.39492428 | 5 |
| 885 | BCL2L1 | BCL2A1 | -28.66261416 | -84.67621136 | 6 |
| 886 | FOS | ATF3 | -28.66261416 | -69.65580396 | 6 |
| 887 | HDAC1 | DNMT1 | -28.64072856 | -93.84842714 | 8 |
| 888 | PRKCB1 | PRKCD | -28.58268394 | -93.97435546 | 7 |
| 889 | NSD1 | PPARBP | -28.57987886 | -50.70810029 | 5 |
| 890 | RELA | RB1 | -28.57115228 | -132.4988695 | 13 |
| 891 | PIK3R1 | EPOR | -28.56861525 | -102.2538134 | 10 |
| 892 | STAT3 | PIK3R1 | -28.56007532 | -132.981611 | 12 |
| 893 | CD40 | TNFRSF11A | -28.54399 | -53.47486974 | 5 |
| 894 | SMARCB1 | SMARCC1 | -28.54399 | -60.1994413 | 5 |
| 895 | JUN | TBP | -28.53192292 | -136.9601126 | 12 |
| 896 | MAPK8 | MAPK9 | -28.52616406 | -86.06256784 | 7 |
| 897 | PLCG1 | CRK | -28.51318208 | -108.3651143 | 10 |
| 898 | GAB2 | SHB | -28.50342002 | -59.79636895 | 6 |
| 899 | ORC1L | MCM2 | -28.50116267 | -77.44608277 | 6 |
| 900 | LSM1 | LSM5 | -28.49218063 | -74.64573639 | 6 |
| 901 | HRAS | RRAS2 | -28.48684069 | -81.74668972 | 7 |
| 902 | VAV1 | LCK | -28.47502339 | -102.7353504 | 10 |
| 903 | CHUK | RIPK1 | -28.4591813 | -77.69048476 | 7 |
| 904 | PLCG1 | PLCG2 | -28.45595749 | -99.3640846 | 8 |
| 905 | SIT1 | PTK2B | -28.4489583 | -57.14192258 | 6 |
| 906 | PML | NR3C1 | -28.43470265 | -108.7244988 | 11 |
| 907 | TNFRSF19 | TRIM37 | -28.43271869 | -53.47486974 | 5 |
| 908 | STAT5A | STAT1 | -28.43244139 | -86.62968762 | 8 |
| 909 | ESR1 | VDR | -28.40291637 | -90.68348975 | 8 |
| 910 | PSMA1 | LNX | -28.39916854 | -77.56049878 | 7 |
| 911 | SRC | CRKL | -28.39406721 | -116.7709607 | 11 |
| 912 | NRIP1 | NR0B2 | -28.35812221 | -68.87628895 | 6 |
| 913 | HSPA1A | HSPA8 | -28.35095766 | -111.364164 | 8 |
| 914 | KHDRBS1 | FASLG | -28.33195004 | -68.99242733 | 7 |
| 915 | SIT1 | CBL | -28.32126168 | -59.36900012 | 6 |
| 916 | BIRC3 | BIRC4 | -28.30663518 | -64.46736764 | 5 |
| 917 | TNPO1 | IPO7 | -28.30051564 | -71.48477226 | 5 |
| 918 | SYK | GAB2 | -28.29460641 | -69.91291521 | 7 |
| 919 | SYK | PAG1 | -28.29460641 | -67.87961111 | 7 |
| 920 | PRKCZ | PRKCI | -28.2891537 | -81.46463484 | 6 |
| 921 | NCOA1 | PELP1 | -28.28609734 | -57.25043877 | 6 |
| 922 | RIBC2 | USP2 | -28.28281176 | -50.42322479 | 5 |
| 923 | VAV1 | CRKL | -28.24067621 | -99.54411743 | 9 |
| 924 | GNB1 | GNB5 | -28.21979171 | -81.58546998 | 5 |
| 925 | ORC1L | MCM10 | -28.21429848 | -76.72650233 | 6 |
| 926 | GHR | EGFR | -28.20961701 | -95.51617822 | 9 |
| 927 | RGS18 | RIC8 | -28.18898346 | -44.28768091 | 4 |
| 928 | RGS16 | RGS18 | -28.1868091 | -44.28768091 | 4 |
| 929 | RGS16 | RGS5 | -28.1868091 | -45.27029429 | 4 |
| 930 | RIC8 | RGS5 | -28.1868091 | -45.27029429 | 4 |
| 931 | RAB11FIP2 | RAB11FIP5 | -28.1868091 | -59.25927319 | 4 |
| 932 | ACTR2 | ARPC5 | -28.1868091 | -57.62736242 | 4 |
| 933 | NRP1 | NRP2 | -28.1868091 | -59.95971967 | 4 |
| 934 | Rasd2 | Rhebl1 | -28.1868091 | -36.83099199 | 4 |
| 935 | Rasd2 | Arhgef7 | -28.1868091 | -35.40988928 | 4 |
| 936 | Rasd2 | 1200013B22Rik | -28.1868091 | -35.47234916 | 4 |
| 937 | Rasd2 | Arhgef6 | -28.1868091 | -35.59583626 | 4 |
| 938 | Rasd2 | Trim35 | -28.1868091 | -35.40988928 | 4 |
| 939 | Rasd2 | Smad2 | -28.1868091 | -36.73198694 | 4 |
| 940 | Rasd2 | Ilkap | -28.1868091 | -36.15057069 | 4 |
| 941 | Rasd2 | Nkiras1 | -28.1868091 | -38.15308965 | 4 |
| 942 | Rasd2 | Pftk1 | -28.1868091 | -36.83099199 | 4 |
| 943 | Rasd2 | Nat5 | -28.1868091 | -36.83099199 | 4 |
| 944 | Rasd2 | Smad7 | -28.1868091 | -37.36162024 | 4 |
| 945 | GRB2 | PIK3R2 | -28.18542496 | -102.9243264 | 9 |
| 946 | PTK2 | BCAR1 | -28.18271888 | -102.4010602 | 9 |
| 947 | VAV1 | WAS | -28.17807724 | -81.67199171 | 8 |
| 948 | PAG1 | PECAM1 | -28.12515664 | -58.96763868 | 6 |
| 949 | GHR | BCR | -28.12189067 | -57.57909023 | 6 |
| 950 | TYK2 | IL4R | -28.12189067 | -68.83726301 | 6 |
| 951 | FLJ32855 | TU3A | -28.12189067 | -60.3720014 | 6 |
| 952 | CD22 | EPOR | -28.11973949 | -55.28788369 | 6 |
| 953 | SAA1 | HABP2 | -28.11971896 | -50.17449604 | 4 |
| 954 | ASIP | POMC | -28.11971896 | -63.55428844 | 4 |
| 955 | POP1 | POP7 | -28.11971896 | -55.74247697 | 4 |
| 956 | POP1 | POP4 | -28.11971896 | -55.74247697 | 4 |
| 957 | Rab38 | Rps27a | -28.11971896 | -35.35910128 | 4 |
| 958 | Rab38 | Fbxo3 | -28.11971896 | -35.59583626 | 4 |
| 959 | Rab38 | Sqstm1 | -28.11971896 | -36.08811081 | 4 |
| 960 | Rab38 | Uhmk1 | -28.11971896 | -35.35910128 | 4 |
| 961 | Rab38 | Rhod | -28.11971896 | -35.35910128 | 4 |
| 962 | Rhobtb1 | Sqstm1 | -28.11971896 | -38.00368784 | 4 |
| 963 | Rhobtb1 | Map2k3 | -28.11971896 | -36.03952258 | 4 |
| 964 | Rhobtb1 | Rhod | -28.11971896 | -36.83099199 | 4 |
| 965 | CCL8 | CCL14 | -28.11917541 | -50.19981384 | 4 |
| 966 | MAP2K2 | KSR2 | -28.11917541 | -44.02281861 | 4 |
| 967 | SCNN1G | SCNN1A | -28.11917541 | -52.72839556 | 4 |
| 968 | TAF1A | TAF1B | -28.11917541 | -46.92642106 | 4 |
| 969 | TAF1C | TAF1B | -28.11917541 | -46.92642106 | 4 |
| 970 | CRIPT | ATP2B4 | -28.11917541 | -43.61261183 | 4 |
| 971 | CRIPT | GUCY1A2 | -28.11917541 | -43.61261183 | 4 |
| 972 | CDC45L | CCDC5 | -28.11917541 | -47.13266257 | 4 |
| 973 | SFRS2IP | SFRS11 | -28.11917541 | -48.7106621 | 4 |
| 974 | AQP1 | FLJ22746 | -28.11917541 | -39.12516735 | 4 |
| 975 | Rps27a | Stat1 | -28.11917541 | -35.47234916 | 4 |
| 976 | Fbxo3 | Rhoj | -28.11917541 | -36.83099199 | 4 |
| 977 | Sqstm1 | Rhoj | -28.11917541 | -37.32326654 | 4 |
| 978 | Sqstm1 | Smad3 | -28.11917541 | -35.40988928 | 4 |
| 979 | Sqstm1 | Rras2 | -28.11917541 | -36.87958022 | 4 |
| 980 | Map2k3 | Rhoj | -28.11917541 | -35.35910128 | 4 |
| 981 | Map2k3 | Smad3 | -28.11917541 | -35.40988928 | 4 |
| 982 | Uhmk1 | Stat1 | -28.11917541 | -35.47234916 | 4 |
| 983 | Rhod | Rras2 | -28.11917541 | -38.6453642 | 4 |
| 984 | Rhod | Stat1 | -28.11917541 | -36.79444682 | 4 |
| 985 | EPOR | PRLR | -28.10667203 | -71.25452147 | 6 |
| 986 | SMN1 | LSM6 | -28.10667203 | -74.79081157 | 6 |
| 987 | VAMP8 | VAMP2 | -28.10596483 | -61.57591392 | 5 |
| 988 | SNX6 | SNX4 | -28.10188542 | -57.44171401 | 5 |
| 989 | MAPK8IP3 | DUSP16 | -28.10188542 | -63.3004028 | 5 |
| 990 | MAP3K14 | TNFRSF11A | -28.10188542 | -52.45233975 | 5 |
| 991 | CFLAR | DEDD | -28.10188542 | -60.34274537 | 5 |
| 992 | PROC | F10 | -28.06727287 | -68.03132419 | 5 |
| 993 | SNRPB | SNRPD2 | -28.06727287 | -67.28938684 | 5 |
| 994 | GRB2 | SHB | -28.03828855 | -107.7701219 | 10 |
| 995 | SPTAN1 | SPTA1 | -28.01033347 | -68.63134539 | 5 |
| 996 | EXOC8 | RALA | -28.00370146 | -91.69729533 | 6 |
| 997 | B2M | LILRB2 | -27.95180083 | -68.16645686 | 5 |
| 998 | NCOA1 | NR3C1 | -27.94520126 | -120.6194393 | 11 |
| 999 | RAPGEF1 | CRKL | -27.94002377 | -61.59795291 | 6 |
| 1000 | RIPK1 | MAP3K14 | -27.93834498 | -61.73017949 | 6 |
| 1001 | LSM1 | LSM2 | -27.87545703 | -74.81279048 | 6 |
| 1002 | KPNB1 | RANBP5 | -27.86537421 | -76.57968575 | 6 |
| 1003 | JAK2 | CSF1R | -27.85080627 | -71.22877744 | 7 |
| 1004 | EGFR | PLCG1 | -27.83957197 | -150.562416 | 13 |
| 1005 | EP300 | PCAF | -27.83447845 | -111.4298623 | 9 |
| 1006 | JAK2 | JAK3 | -27.83250313 | -97.42756677 | 8 |
| 1007 | HDAC1 | CTBP1 | -27.80774211 | -106.6428601 | 9 |
| 1008 | RB1 | HDAC2 | -27.75973352 | -130.922496 | 11 |
| 1009 | CD40 | USP7 | -27.73414822 | -53.47486974 | 5 |
| 1010 | TNFAIP3 | TRAF3IP2 | -27.73414822 | -53.03578802 | 5 |
| 1011 | LSM1 | LSM8 | -27.7225544 | -89.24210394 | 7 |
| 1012 | AFAP | TIAM1 | -27.70090808 | -55.26744198 | 5 |
| 1013 | RRAS2 | MRAS | -27.69614845 | -61.97646751 | 5 |
| 1014 | UBE2L3 | UBE2L6 | -27.69614845 | -68.45513843 | 5 |
| 1015 | TU3A | ZNF426 | -27.69614845 | -49.49214786 | 5 |
| 1016 | PTPN11 | IRS2 | -27.68715789 | -81.38540042 | 8 |
| 1017 | FN1 | SERPINE2 | -27.6814734 | -76.79095267 | 6 |
| 1018 | COL4A4 | COL1A1 | -27.67913348 | -73.00048648 | 6 |
| 1019 | CD82 | CD9 | -27.65365804 | -83.60291688 | 6 |
| 1020 | DLG4 | GRIN2A | -27.65076067 | -100.5826562 | 8 |
| 1021 | FXR2 | C6orf55 | -27.63500003 | -90.24214927 | 7 |
| 1022 | RAN | RANBP2 | -27.63343681 | -76.34098979 | 6 |
| 1023 | KIAA0408 | ZNF417 | -27.62597026 | -53.66983174 | 5 |
| 1024 | DDIT3 | JUNB | -27.62016462 | -78.71642132 | 6 |
| 1025 | PTPN11 | LCK | -27.6175111 | -125.8525664 | 11 |
| 1026 | HLA-F | HLA-G | -27.60119679 | -54.74981326 | 4 |
| 1027 | SH2D1A | SH2D1B | -27.59847866 | -63.95975355 | 4 |
| 1028 | TOB1 | ZFHX1B | -27.59847866 | -40.52136615 | 4 |
| 1029 | JAK1 | IRS2 | -27.5869937 | -73.19529797 | 7 |
| 1030 | JAK1 | IL2RG | -27.5869937 | -85.43911519 | 7 |
| 1031 | RELA | BCL3 | -27.56593552 | -82.88024499 | 8 |
| 1032 | CCNA2 | CDK2 | -27.54503195 | -82.66833488 | 7 |
| 1033 | SOS2 | SOS1 | -27.51785639 | -49.3826976 | 5 |
| 1034 | MET | PIK3R1 | -27.51275867 | -79.44140428 | 8 |
| 1035 | PTPN6 | ZAP70 | -27.50930161 | -104.0584032 | 9 |
| 1036 | GHR | IRS1 | -27.50769973 | -70.78520874 | 7 |
| 1037 | TYK2 | GRB2 | -27.5008727 | -106.8414065 | 10 |
| 1038 | YWHAB | YWHAQ | -27.49339357 | -97.66598595 | 7 |
| 1039 | PPP5C | SKIL | -27.46127365 | -65.44735682 | 5 |
| 1040 | RUNX1T1 | HDAC9 | -27.42956282 | -64.09889222 | 6 |
| 1041 | SNRPE | SMN1 | -27.42863727 | -76.74068212 | 6 |
| 1042 | LSM5 | SMN1 | -27.42863727 | -74.97313313 | 6 |
| 1043 | KIT | CSF1R | -27.4163857 | -62.01112592 | 6 |
| 1044 | MAPK14 | MAPK3 | -27.41552412 | -131.2781279 | 9 |
| 1045 | SMAD2 | SKI | -27.37415474 | -74.26165399 | 7 |
| 1046 | AFAP | YWHAG | -27.32662305 | -55.46283562 | 5 |
| 1047 | MCM5 | MCM3 | -27.32662305 | -60.18697567 | 5 |
| 1048 | YWHAG | HABP4 | -27.32118309 | -60.53465561 | 5 |
| 1049 | LSM1 | LSM3 | -27.31815716 | -74.96694115 | 6 |
| 1050 | TRADD | CFLAR | -27.31815716 | -67.8488607 | 6 |
| 1051 | SMAD1 | SMAD5 | -27.301798 | -94.07741251 | 7 |
| 1052 | KIAA1446 | HOOK2 | -27.29752909 | -76.5316796 | 6 |
| 1053 | RIBC2 | 76P | -27.29752909 | -63.49006452 | 6 |
| 1054 | HLA-C | HLA-G | -27.29217978 | -69.31080454 | 5 |
| 1055 | TRAF3 | TRAF6 | -27.28900439 | -108.2903321 | 8 |
| 1056 | SAA1 | SERPINE2 | -27.27255705 | -50.17449604 | 4 |
| 1057 | CASP14 | CARD4 | -27.27255705 | -52.42602596 | 4 |
| 1058 | RCV1 | RHO | -27.27255705 | -54.06631633 | 4 |
| 1059 | TNFRSF19 | TNFRSF8 | -27.27255705 | -43.16570148 | 4 |
| 1060 | MCM10 | ORC5L | -27.2721329 | -60.93336737 | 5 |
| 1061 | COL2A1 | COL3A1 | -27.2721329 | -65.52092702 | 5 |
| 1062 | LRPAP1 | SNX17 | -27.27146987 | -51.93475684 | 4 |
| 1063 | ARHGDIB | DEF6 | -27.27146987 | -42.74755273 | 4 |
| 1064 | ARHGDIB | ARHGDIA | -27.27146987 | -42.74755273 | 4 |
| 1065 | CCL7 | CCL14 | -27.27146987 | -50.19981384 | 4 |
| 1066 | KCNJ4 | ATP2B4 | -27.27146987 | -42.61223798 | 4 |
| 1067 | BRCA1 | FOS | -27.24849888 | -114.7270232 | 10 |
| 1068 | BRCA1 | CEBPB | -27.24849888 | -102.0027975 | 10 |
| 1069 | GHR | IRS2 | -27.2201112 | -57.3448705 | 6 |
| 1070 | SYK | LAT | -27.19761812 | -80.54694938 | 7 |
| 1071 | VAV1 | RASA1 | -27.19467333 | -90.71777802 | 9 |
| 1072 | PCNA | RAD17 | -27.17693894 | -103.9468359 | 7 |
| 1073 | PLCG1 | NCK1 | -27.14934698 | -119.71482 | 10 |
| 1074 | RELA | POU2F1 | -27.14682847 | -94.87029664 | 9 |
| 1075 | GNAO1 | GNAQ | -27.14202476 | -101.1365068 | 7 |
| 1076 | NCOR2 | HDAC1 | -27.13248825 | -111.3268798 | 10 |
| 1077 | TGFB1 | TGFBR2 | -27.13153358 | -90.07271494 | 7 |
| 1078 | MYF6 | MYOG | -27.09037117 | -47.36442422 | 4 |
| 1079 | GLI1 | GLI3 | -27.09037117 | -58.93718969 | 4 |
| 1080 | BMP7 | GDF9 | -27.08710919 | -48.50611559 | 4 |
| 1081 | NDP52 | Cep70 | -27.07965998 | -90.31143801 | 7 |
| 1082 | GAB3 | PTK2B | -27.06771202 | -52.96679631 | 6 |
| 1083 | NCOR2 | SIN3A | -27.05553352 | -95.39919376 | 8 |
| 1084 | BRCA1 | RELA | -27.04929809 | -118.9285713 | 12 |
| 1085 | TF | IGF2 | -27.04412947 | -76.47952451 | 5 |
| 1086 | ELA2 | CTSG | -27.04208963 | -62.57648804 | 5 |
| 1087 | TGFB2 | ENG | -27.04208963 | -57.09350946 | 5 |
| 1088 | RET | MST1R | -27.03209003 | -43.70632075 | 5 |
| 1089 | GDF5 | BMP6 | -27.020835 | -48.50611559 | 4 |
| 1090 | IRF2 | IRF1 | -27.020835 | -46.23426357 | 4 |
| 1091 | CCL8 | CCL2 | -27.020835 | -49.64386779 | 4 |
| 1092 | EDNRB | EDNRA | -27.020835 | -59.05447877 | 4 |
| 1093 | TAF1A | TAF1C | -27.020835 | -47.33188617 | 4 |
| 1094 | EIF2B2 | EIF2S2 | -27.020835 | -54.85144591 | 4 |
| 1095 | ARPC3 | ARPC1B | -27.020835 | -57.91949884 | 4 |
| 1096 | POP7 | POP4 | -27.020835 | -55.74247697 | 4 |
| 1097 | ARPC1B | ARPC2 | -27.020835 | -57.91949884 | 4 |
| 1098 | ARPC1B | ARPC4 | -27.020835 | -57.91949884 | 4 |
| 1099 | Fbxo3 | Rhod | -27.020835 | -36.83099199 | 4 |
| 1100 | Sqstm1 | Rhod | -27.020835 | -37.32326654 | 4 |
| 1101 | Map2k3 | Rhod | -27.020835 | -35.35910128 | 4 |
| 1102 | ITGA2B | THBS1 | -27.00429193 | -71.89454504 | 6 |
| 1103 | FYN | SHC1 | -26.97993617 | -152.1690297 | 14 |
| 1104 | LAT | SH3BP2 | -26.97260445 | -50.07027195 | 5 |
| 1105 | JUND | ATF4 | -26.96568623 | -76.10495195 | 6 |
| 1106 | PAG1 | PXN | -26.95366385 | -70.50842589 | 7 |
| 1107 | ACVR1 | ACVR2B | -26.94799495 | -95.74354004 | 7 |
| 1108 | RAN | NUP153 | -26.94342336 | -76.00451755 | 6 |
| 1109 | SH3BP2 | CBL | -26.94015217 | -62.3722613 | 6 |
| 1110 | PML | RBBP4 | -26.9221688 | -67.63422235 | 7 |
| 1111 | PML | HDAC9 | -26.9221688 | -75.64620217 | 7 |
| 1112 | AR | JUN | -26.92025265 | -128.7676866 | 13 |
| 1113 | JAK2 | IRS1 | -26.90932285 | -94.63093022 | 9 |
| 1114 | SMARCD1 | TU3A | -26.89065893 | -50.48654184 | 5 |
| 1115 | PAG1 | CSF3R | -26.88657896 | -46.14039495 | 5 |
| 1116 | RRAS2 | RAP2A | -26.88657896 | -61.97646751 | 5 |
| 1117 | STX1A | SNAP25 | -26.846359 | -96.19168071 | 7 |
| 1118 | TNFRSF1A | FAS | -26.84397166 | -83.64449384 | 7 |
| 1119 | LSM1 | LSM4 | -26.80964777 | -75.110042 | 6 |
| 1120 | PARP1 | XRCC5 | -26.80964777 | -72.00453482 | 6 |
| 1121 | GAB2 | PTK2B | -26.80060756 | -64.91204971 | 7 |
| 1122 | KIT | TEK | -26.80020772 | -60.55872811 | 6 |
| 1123 | PTPN11 | FYN | -26.78698842 | -150.8492762 | 13 |
| 1124 | LNX | C1orf65 | -26.77408631 | -58.36081198 | 6 |
| 1125 | CREBBP | STAT3 | -26.75949241 | -111.6956076 | 11 |
| 1126 | ORC1L | MCM7 | -26.73498537 | -78.18871001 | 6 |
| 1127 | SHC1 | IRS1 | -26.73411898 | -107.4640231 | 10 |
| 1128 | SYK | SHB | -26.70964461 | -70.85830254 | 7 |
| 1129 | KIT | VAV1 | -26.70933302 | -81.55110597 | 8 |
| 1130 | PIK3R1 | PAG1 | -26.67775566 | -75.00472757 | 8 |
| 1131 | PIK3R1 | IL4R | -26.67775566 | -89.90796538 | 8 |
| 1132 | CDC42 | RHOQ | -26.66750983 | -102.6940729 | 7 |
| 1133 | HDAC1 | SP1 | -26.66033654 | -134.8172308 | 12 |
| 1134 | TGFBI | FBLN2 | -26.65371095 | -59.98621679 | 5 |
| 1135 | GAB2 | CBL | -26.65087921 | -66.22679692 | 7 |
| 1136 | CBLB | SH3BP2 | -26.64691007 | -51.50535648 | 5 |
| 1137 | SHB | SH3BP2 | -26.64691007 | -50.58752847 | 5 |
| 1138 | COPB | COPG2 | -26.64691007 | -69.41761855 | 5 |
| 1139 | CSK | ZAP70 | -26.64571902 | -76.0126803 | 7 |
| 1140 | ERBB2 | PTK2 | -26.64432014 | -87.50233371 | 9 |
| 1141 | JAK1 | GHR | -26.64344444 | -70.65298343 | 7 |
| 1142 | JAK1 | TYK2 | -26.64344444 | -81.23276747 | 7 |
| 1143 | NSD1 | POU2F1 | -26.64327466 | -50.70810029 | 5 |
| 1144 | HUS1B | RAD17 | -26.63838605 | -56.11668953 | 4 |
| 1145 | ZNF205 | C16orf48 | -26.63838605 | -35.02199211 | 4 |
| 1146 | RAB27A | RAB27B | -26.63458015 | -60.44522748 | 4 |
| 1147 | SKIL | Smad2 | -26.63458015 | -36.73198694 | 4 |
| 1148 | FYN | ZAP70 | -26.61667077 | -112.1963479 | 10 |
| 1149 | MEF2A | MEF2C | -26.60025734 | -58.54787745 | 5 |
| 1150 | MAP3K14 | TANK | -26.60025734 | -53.90661637 | 5 |
| 1151 | PIK3R1 | PTPRC | -26.58983941 | -104.0969969 | 10 |
| 1152 | RELA | TBP | -26.58086707 | -122.1953077 | 11 |
| 1153 | SAA1 | TGFBI | -26.57954584 | -50.17449604 | 4 |
| 1154 | ORC4L | CCDC5 | -26.57791496 | -48.94936099 | 4 |
| 1155 | DUSP16 | DUSP10 | -26.57791496 | -51.21141476 | 4 |
| 1156 | PER1 | PER2 | -26.57791496 | -57.97833934 | 4 |
| 1157 | PACSIN1 | PACSIN3 | -26.57791496 | -54.48744899 | 4 |
| 1158 | CDH1 | CDH2 | -26.55918279 | -70.71629269 | 6 |
| 1159 | MCM10 | MCM7 | -26.55681282 | -92.08472499 | 7 |
| 1160 | PCNA | CDKN1A | -26.54031121 | -111.1035904 | 9 |
| 1161 | CEBPG | ATF4 | -26.53159573 | -73.90630389 | 6 |
| 1162 | FN1 | SPARC | -26.53137857 | -85.55369115 | 7 |
| 1163 | MCM10 | TU3A | -26.52237556 | -62.65693715 | 6 |
| 1164 | TTRAP | RIPK2 | -26.51651006 | -52.9854353 | 5 |
| 1165 | ERBB2IP | TOB1 | -26.51174978 | -47.61632059 | 5 |
| 1166 | NDP52 | HOOK2 | -26.50975511 | -89.0340068 | 7 |
| 1167 | DDIT3 | FOSL2 | -26.50151047 | -58.98335592 | 5 |
| 1168 | PTK2 | SOS1 | -26.49318118 | -79.21035908 | 8 |
| 1169 | ERBB2 | SHC1 | -26.4748464 | -101.7854067 | 10 |
| 1170 | SUV39H1 | HDAC1 | -26.44822107 | -91.43567698 | 8 |
| 1171 | TNFRSF1A | RIPK1 | -26.44232752 | -81.65958343 | 7 |
| 1172 | VAV1 | PLCG1 | -26.44013358 | -106.6201935 | 10 |
| 1173 | NCOA3 | PPARBP | -26.43869967 | -63.79203231 | 6 |
| 1174 | RASA1 | CBL | -26.39744241 | -95.07446205 | 9 |
| 1175 | GRB2 | EPOR | -26.39462541 | -113.8048429 | 11 |
| 1176 | ACVR2A | INHBA | -26.38436996 | -67.53599463 | 5 |
| 1177 | SYK | PTPRC | -26.36754293 | -84.07529317 | 8 |
| 1178 | JAK2 | LCK | -26.35162342 | -107.8996353 | 10 |
| 1179 | GAB3 | PECAM1 | -26.34873788 | -45.26486014 | 5 |
| 1180 | APEX2 | FLJ32855 | -26.34873788 | -49.52620688 | 5 |
| 1181 | VAV1 | VAV2 | -26.34339147 | -64.47618786 | 6 |
| 1182 | AR | HNF4A | -26.34159753 | -96.55514649 | 8 |
| 1183 | LCP2 | SH3BP2 | -26.34125641 | -54.5705717 | 5 |
| 1184 | BCL2A1 | BCL2 | -26.34031465 | -84.67621136 | 6 |
| 1185 | PML | ZBTB16 | -26.30219048 | -98.11560236 | 9 |
| 1186 | PTK2 | EGFR | -26.29902089 | -123.5332216 | 12 |
| 1187 | RARA | THRB | -26.28520605 | -92.70389291 | 7 |
| 1188 | JAK1 | IRS1 | -26.27818665 | -84.86777107 | 8 |
| 1189 | SYK | TYK2 | -26.25461587 | -69.51237863 | 7 |
| 1190 | SYK | LCP2 | -26.25461587 | -73.75246527 | 7 |
| 1191 | IFNAR1 | EPOR | -26.25407303 | -58.67465787 | 6 |
| 1192 | LSM3 | SMN1 | -26.25407303 | -75.29433789 | 6 |
| 1193 | EPOR | CSF2RB | -26.24345355 | -57.72540694 | 6 |
| 1194 | ARF5 | ARF6 | -26.23292094 | -55.90459582 | 4 |
| 1195 | LYN | CBL | -26.21533389 | -110.1502558 | 10 |
| 1196 | RELA | REL | -26.20430614 | -83.44515259 | 7 |
| 1197 | CCR2 | CCBP2 | -26.1981929 | -73.53031225 | 5 |
| 1198 | GAB1 | IRS4 | -26.19479269 | -47.08243366 | 5 |
| 1199 | GAB2 | IRS4 | -26.19479269 | -46.22744345 | 5 |
| 1200 | CCBP2 | CCR1 | -26.19479269 | -69.99743704 | 5 |
| 1201 | NGB | RGS14 | -26.17380907 | -45.14714931 | 4 |
| 1202 | CCL2 | CCL7 | -26.17380907 | -52.25393758 | 4 |
| 1203 | AQP1 | CATSPER1 | -26.17380907 | -40.26988446 | 4 |
| 1204 | ASK | ORC5L | -26.17326544 | -47.15068107 | 4 |
| 1205 | GGA3 | AP1G2 | -26.17326544 | -58.1606609 | 4 |
| 1206 | CDK8 | MED6 | -26.17326544 | -52.83375607 | 4 |
| 1207 | TNFRSF13B | TNFRSF17 | -26.17326544 | -50.5052473 | 4 |
| 1208 | KIT | PTK2B | -26.16997962 | -78.71133804 | 8 |
| 1209 | RYR1 | ITPR1 | -26.16874803 | -68.77226203 | 5 |
| 1210 | CDC7 | ORC2L | -26.16874803 | -65.63292904 | 5 |
| 1211 | PTK2B | NEDD9 | -26.14596377 | -82.12554369 | 8 |
| 1212 | VAV1 | LAT | -26.14544358 | -79.60603673 | 7 |
| 1213 | VAV1 | PLCG2 | -26.14544358 | -80.77344539 | 7 |
| 1214 | CREBBP | FOS | -26.1304523 | -112.2594596 | 10 |
| 1215 | RXRA | GTF2B | -26.09427796 | -92.04895001 | 8 |
| 1216 | SHC1 | CRKL | -26.09252491 | -111.9426427 | 10 |
| 1217 | RIBC2 | FLJ32855 | -26.08297992 | -62.70368287 | 6 |
| 1218 | FYN | HCK | -26.08203547 | -111.8083817 | 9 |
| 1219 | ORC5L | MCM7 | -26.07540989 | -64.70612831 | 5 |
| 1220 | PTPN11 | JAK3 | -26.06702426 | -87.13140982 | 8 |
| 1221 | COL2A1 | COL1A2 | -26.05482492 | -73.73665104 | 6 |
| 1222 | KRT15 | HOOK2 | -26.04797659 | -88.52969586 | 7 |
| 1223 | GTF2B | GTF2A2 | -26.0219562 | -63.37636434 | 5 |
| 1224 | SYK | LCK | -26.02174061 | -98.71271079 | 9 |
| 1225 | RASA1 | LCK | -26.02174061 | -101.6812253 | 9 |
| 1226 | ACVR1 | SMURF2 | -26.00698853 | -133.9969309 | 9 |
| 1227 | JUN | POU2F1 | -26.00591918 | -93.51266838 | 9 |
| 1228 | DLG4 | DLGAP1 | -26.00171362 | -88.69931076 | 7 |
| 1229 | DLG4 | INADL | -26.00171362 | -101.4123544 | 7 |
| 1230 | KIT | CBL | -25.99822773 | -78.7639967 | 8 |
| 1231 | EPOR | CBL | -25.99822773 | -76.12978877 | 8 |
| 1232 | SNX6 | SNX1 | -25.99534644 | -57.96129243 | 5 |
| 1233 | MAPK8IP3 | MAP2K7 | -25.99534644 | -61.98821641 | 5 |
| 1234 | SAA1 | OSM | -25.99189517 | -50.17449604 | 4 |
| 1235 | POP1 | RPP38 | -25.99189517 | -56.61794571 | 4 |
| 1236 | ATP2B4 | LIN7A | -25.99189517 | -44.17987877 | 4 |
| 1237 | EIF3S4 | EIF3S1 | -25.98972051 | -56.95887229 | 4 |
| 1238 | SP3 | ARID4A | -25.98972051 | -39.36198961 | 4 |
| 1239 | TAF5 | TAF13 | -25.98972051 | -50.73596539 | 4 |
| 1240 | SRC | CSK | -25.98319645 | -95.09759376 | 9 |
| 1241 | PTPN6 | LCK | -25.97827333 | -109.6297896 | 10 |
| 1242 | NCOA1 | POU2F1 | -25.97311854 | -82.52715501 | 8 |
| 1243 | CCND1 | CDK4 | -25.97129557 | -86.8846738 | 7 |
| 1244 | PML | HDAC1 | -25.9607699 | -114.767883 | 11 |
| 1245 | NCOR2 | ZBTB16 | -25.95446679 | -91.1493448 | 8 |
| 1246 | BMPR1B | BMPR1A | -25.90932373 | -97.46119153 | 7 |
| 1247 | FN1 | COL4A1 | -25.90932373 | -88.75865338 | 7 |
| 1248 | FN1 | NID | -25.90932373 | -95.08507842 | 7 |
| 1249 | ACVR1B | INHBA | -25.89913493 | -63.39244847 | 5 |
| 1250 | CD2AP | CSF1R | -25.89913493 | -47.56304244 | 5 |
| 1251 | NR3C1 | EP300 | -25.88535953 | -135.2363115 | 12 |
| 1252 | RABAC1 | RPIA | -25.8826407 | -56.04034931 | 5 |
| 1253 | RAPGEF5 | RALGDS | -25.86519616 | -54.96458856 | 4 |
| 1254 | EPHA2 | EPHA5 | -25.86030219 | -60.55855617 | 4 |
| 1255 | PTPN11 | ZAP70 | -25.85712434 | -105.0834278 | 9 |
| 1256 | SUV39H1 | DNMT3B | -25.82009971 | -59.43000834 | 5 |
| 1257 | COL1A2 | COL4A5 | -25.82009971 | -56.15525924 | 5 |
| 1258 | COL1A2 | COL4A6 | -25.82009971 | -56.15525924 | 5 |
| 1259 | SOCS1 | GRB10 | -25.80727332 | -61.48523974 | 6 |
| 1260 | TIF1 | NCOA2 | -25.80727332 | -61.97018096 | 6 |
| 1261 | GRB7 | SHC1 | -25.80595305 | -71.59818878 | 7 |
| 1262 | FRS2 | SHC1 | -25.80595305 | -76.37057285 | 7 |
| 1263 | JAK2 | PAG1 | -25.77593025 | -64.29421958 | 7 |
| 1264 | DLG4 | IL16 | -25.76379932 | -114.27875 | 8 |
| 1265 | ESR1 | THRA | -25.76379932 | -99.52703704 | 8 |
| 1266 | CTBP1 | CTBP2 | -25.74746301 | -86.47081578 | 6 |
| 1267 | LSM4 | SMN1 | -25.7452932 | -75.43743873 | 6 |
| 1268 | LSM7 | LSM8 | -25.7452932 | -76.78117348 | 6 |
| 1269 | RAD54B | TSC22D4 | -25.7452932 | -80.08263092 | 6 |
| 1270 | KIT | GAB1 | -25.73548994 | -57.47177695 | 6 |
| 1271 | EPOR | GAB2 | -25.73548994 | -55.89750098 | 6 |
| 1272 | EPOR | BCR | -25.73548994 | -55.66737145 | 6 |
| 1273 | SMN1 | LSM7 | -25.73548994 | -75.43743873 | 6 |
| 1274 | CDK4 | CDK2 | -25.73261747 | -78.61502781 | 7 |
| 1275 | KRT18 | ZFP36 | -25.71219627 | -54.3838536 | 5 |
| 1276 | MDFI | RBPMS | -25.70367233 | -153.2803103 | 11 |
| 1277 | ELA2 | PLG | -25.69756055 | -76.61138623 | 6 |
| 1278 | MYOD1 | TCF4 | -25.68203555 | -71.24928561 | 6 |
| 1279 | MCM10 | ORC2L | -25.62196016 | -78.93110702 | 6 |
| 1280 | YWHAZ | YWHAG | -25.61493317 | -86.23784047 | 7 |
| 1281 | CD22 | PAG1 | -25.59273834 | -46.49051488 | 5 |
| 1282 | SNRPD2 | DDX20 | -25.59273834 | -67.58663837 | 5 |
| 1283 | IGFBP3 | IGFBP5 | -25.59001799 | -67.50010787 | 5 |
| 1284 | JAK1 | SHC1 | -25.55532621 | -108.9739224 | 10 |
| 1285 | CRK | SHC1 | -25.55532621 | -104.6239571 | 10 |
| 1286 | LAT | CBL | -25.5547227 | -76.51566295 | 7 |
| 1287 | NCOR2 | HDAC2 | -25.55451264 | -100.5371045 | 8 |
| 1288 | GHR | CSF3R | -25.53223173 | -46.34962755 | 5 |
| 1289 | FLJ32855 | MGC3162 | -25.53223173 | -56.49342571 | 5 |
| 1290 | COL4A3 | COL1A1 | -25.527286 | -73.00048648 | 6 |
| 1291 | RAD9A | RAD9B | -25.52328581 | -56.62021586 | 4 |
| 1292 | NCOA1 | BCL3 | -25.52321906 | -69.77268737 | 7 |
| 1293 | PTPN6 | PAG1 | -25.51656049 | -70.87137397 | 7 |
| 1294 | RICS | WAS | -25.50989597 | -56.75648694 | 6 |
| 1295 | KIT | SHC1 | -25.50078877 | -91.39170528 | 9 |
| 1296 | EPOR | SHC1 | -25.50078877 | -90.66107996 | 9 |
| 1297 | PIK3R1 | GRAP2 | -25.49152148 | -107.9418609 | 9 |
| 1298 | JAK1 | PTPN6 | -25.49074168 | -95.71513422 | 9 |
| 1299 | UTRN | DMD | -25.48120555 | -56.93581652 | 4 |
| 1300 | GLI2 | GLI3 | -25.48120555 | -58.93718969 | 4 |
| 1301 | SALL1 | CHD3 | -25.48120555 | -39.12208549 | 4 |
| 1302 | SNRPF | SNRPD1 | -25.48093385 | -52.45873199 | 4 |
| 1303 | NGB | RIC8 | -25.48093385 | -45.14714931 | 4 |
| 1304 | RASIP1 | RASSF5 | -25.48093385 | -53.30636048 | 4 |
| 1305 | AQP1 | ZNF426 | -25.48093385 | -37.18608317 | 4 |
| 1306 | ORC4L | CDC45L | -25.47984652 | -47.0636697 | 4 |
| 1307 | TGFBI | HABP2 | -25.47984652 | -50.17449604 | 4 |
| 1308 | RGS16 | NGB | -25.47984652 | -45.14714931 | 4 |
| 1309 | VAMP8 | SYBL1 | -25.47984652 | -47.47655802 | 4 |
| 1310 | ATF3 | NFE2L1 | -25.47984652 | -45.78109494 | 4 |
| 1311 | GLI3 | ZNF8 | -25.47848705 | -36.88999119 | 4 |
| 1312 | SIP1 | SFRS11 | -25.47848705 | -48.7106621 | 4 |
| 1313 | B2M | CD8A | -25.47179348 | -67.65563124 | 5 |
| 1314 | APOE | DAB1 | -25.45757501 | -62.39035372 | 5 |
| 1315 | HLA-C | HLA-A | -25.45757501 | -67.7703595 | 5 |
| 1316 | DDIT3 | FOS | -25.45216501 | -82.98850513 | 7 |
| 1317 | PLCG1 | SH3BP2 | -25.44571851 | -63.7075924 | 6 |
| 1318 | JUN | AHR | -25.43576828 | -80.14245883 | 8 |
| 1319 | INSR | LEPR | -25.35540322 | -74.28365567 | 6 |
| 1320 | JAK1 | GRB2 | -25.35333762 | -132.714558 | 12 |
| 1321 | GRAP2 | PLCG2 | -25.34820616 | -69.63720598 | 6 |
| 1322 | SYK | CRKL | -25.32832589 | -88.00907411 | 8 |
| 1323 | SCAP1 | TRPV4 | -25.3263755 | -37.03803619 | 4 |
| 1324 | FOSL1 | FOSL2 | -25.3263755 | -45.56062162 | 4 |
| 1325 | KCNJ2 | KCNJ4 | -25.3263755 | -42.49445495 | 4 |
| 1326 | CDK8 | SURB7 | -25.3263755 | -48.63226687 | 4 |
| 1327 | WASF1 | WAS | -25.2878054 | -50.05775399 | 5 |
| 1328 | SMARCA4 | SMARCC1 | -25.27760062 | -59.24059095 | 5 |
| 1329 | SIAH1 | SIAH2 | -25.27760062 | -77.15465318 | 5 |
| 1330 | PRKCB1 | PRKCG | -25.24441386 | -67.63031343 | 5 |
| 1331 | VAV1 | PTK2B | -25.22360492 | -84.85485679 | 9 |
| 1332 | PIAS3 | LEF1 | -25.21886188 | -48.49147141 | 5 |
| 1333 | PLCG1 | CRKL | -25.21543578 | -99.20867118 | 9 |
| 1334 | GRIN2A | GRIN2C | -25.21258667 | -46.19679995 | 4 |
| 1335 | GRIN2A | KCNJ10 | -25.21258667 | -44.62915917 | 4 |
| 1336 | BMPR1A | NOG | -25.21258667 | -56.03242919 | 4 |
| 1337 | RAB3A | RAB3D | -25.21258667 | -56.97503723 | 4 |
| 1338 | FN1 | OSM | -25.20993651 | -76.79095267 | 6 |
| 1339 | CBFA2T2 | HDAC3 | -25.19298036 | -71.00326487 | 6 |
| 1340 | APP | FBLN2 | -25.18688843 | -88.78757752 | 7 |
| 1341 | PTK2B | RICS | -25.18688843 | -69.33195017 | 7 |
| 1342 | FOS | BATF | -25.18355364 | -69.65580396 | 6 |
| 1343 | PIK3R1 | GRB7 | -25.1527072 | -76.2860079 | 7 |
| 1344 | MET | PDGFRB | -25.14067283 | -58.50934408 | 6 |
| 1345 | NCOA1 | RXRG | -25.13457816 | -58.54205292 | 5 |
| 1346 | NCOA1 | NRBF2 | -25.13457816 | -55.92972196 | 5 |
| 1347 | NCOA1 | CGI-63 | -25.13457816 | -55.92972196 | 5 |
| 1348 | IL6ST | GHR | -25.12863944 | -63.96414439 | 6 |
| 1349 | TRAF2 | TRIM37 | -25.12105597 | -121.8424436 | 10 |
| 1350 | TRIM37 | DIPA | -25.10089362 | -127.7944276 | 10 |
| 1351 | CBLB | CBL | -25.06716516 | -70.51217344 | 7 |
| 1352 | SHB | CBL | -25.06716516 | -67.86675492 | 7 |
| 1353 | SNRPE | DDX20 | -25.05442317 | -68.21079267 | 5 |
| 1354 | GAB1 | VAV3 | -25.05238277 | -45.3615413 | 5 |
| 1355 | GAB1 | MAP4K1 | -25.05238277 | -49.05261572 | 5 |
| 1356 | RBBP4 | MBD2 | -25.05238277 | -51.95347087 | 5 |
| 1357 | MSN | RDX | -25.05238277 | -62.86239985 | 5 |
| 1358 | MCM10 | APEX2 | -25.04371357 | -52.65274247 | 5 |
| 1359 | IGF1R | IL4R | -25.04061315 | -62.12239233 | 6 |
| 1360 | NR3C1 | ESR2 | -25.03161763 | -99.01032946 | 8 |
| 1361 | SAA1 | MATN2 | -25.02935645 | -50.17449604 | 4 |
| 1362 | FRS3 | FRS2 | -25.02935645 | -42.27547238 | 4 |
| 1363 | REM1 | BRAF | -25.02935645 | -43.6177529 | 4 |
| 1364 | GRM5 | GRM7 | -25.02935645 | -43.57118475 | 4 |
| 1365 | XPOT | RANBP5 | -25.02935645 | -49.89951331 | 4 |
| 1366 | RCOR3 | KIAA1267 | -25.02935645 | -45.39537254 | 4 |
| 1367 | C10orf10 | ZNF638 | -25.02935645 | -40.08825896 | 4 |
| 1368 | SKIL | Smad3 | -25.02609403 | -36.73198694 | 4 |
| 1369 | PTK2 | NEDD9 | -25.00918384 | -89.72679265 | 8 |
| 1370 | PTPN1 | SHC1 | -24.99548411 | -87.73228904 | 8 |
| 1371 | FXR2 | FLJ22494 | -24.99124445 | -98.83498322 | 8 |
| 1372 | DIPA | MGC2749 | -24.96096403 | -126.9415622 | 10 |
| 1373 | PIK3R1 | INSR | -24.95911887 | -107.5261416 | 10 |
| 1374 | GAB2 | PLCG1 | -24.95328806 | -73.54463814 | 7 |
| 1375 | PTPN6 | SOCS3 | -24.94816216 | -77.97902207 | 7 |
| 1376 | JUN | TP53 | -24.94802184 | -160.6246065 | 15 |
| 1377 | KRT19 | USHBP1 | -24.94109414 | -73.45706607 | 6 |
| 1378 | PTPN12 | CRKL | -24.93618258 | -61.64468678 | 6 |
| 1379 | MCM3 | MCM4 | -24.9243603 | -48.52633704 | 4 |
| 1380 | ASCL2 | PPEF2 | -24.92009373 | -32.95758362 | 3 |
| 1381 | ASCL2 | PPEF1 | -24.92009373 | -32.95758362 | 3 |
| 1382 | PPEF2 | PPEF1 | -24.92009373 | -32.95758362 | 3 |
| 1383 | IGFBP1 | IGFBP2 | -24.92009373 | -38.99164636 | 3 |
| 1384 | IGFBP1 | IGFBP6 | -24.92009373 | -38.99164636 | 3 |
| 1385 | IGFBP2 | IGFBP6 | -24.92009373 | -38.99164636 | 3 |
| 1386 | PTPN7 | DUSP4 | -24.92009373 | -30.5592851 | 3 |
| 1387 | PTPN7 | GMFB | -24.92009373 | -30.5592851 | 3 |
| 1388 | DUSP4 | GMFB | -24.92009373 | -30.5592851 | 3 |
| 1389 | SLITL2 | FMOD | -24.92009373 | -37.19786872 | 3 |
| 1390 | SCN4A | SCN5A | -24.92009373 | -39.93610797 | 3 |
| 1391 | ASF1A | ASF1B | -24.92009373 | -43.15598329 | 3 |
| 1392 | RPL23A | RPS7 | -24.92009373 | -39.50532505 | 3 |
| 1393 | RRAGD | RRAGC | -24.92009373 | -48.45430066 | 3 |
| 1394 | EIF4G2 | EIF4G3 | -24.92009373 | -41.29708452 | 3 |
| 1395 | UCP2 | UCP3 | -24.92009373 | -32.6448995 | 3 |
| 1396 | BIRC1 | COL11A2 | -24.92009373 | -40.78084501 | 3 |
| 1397 | TACR2 | TACR3 | -24.92009373 | -46.15171557 | 3 |
| 1398 | ZIC1 | ZIC2 | -24.92009373 | -43.44366536 | 3 |
| 1399 | CRSP7 | MED19 | -24.92009373 | -37.22294266 | 3 |
| 1400 | MUC2 | MUC5AC | -24.92009373 | -47.76115348 | 3 |
| 1401 | AR | RXRA | -24.90909932 | -130.2125495 | 11 |
| 1402 | PTK2B | ZAP70 | -24.9004961 | -78.51209621 | 8 |
| 1403 | IGSF1 | INHBB | -24.89341918 | -48.21241798 | 4 |
| 1404 | SF3B1 | SF3B4 | -24.89341918 | -56.76681933 | 4 |
| 1405 | POP7 | RPP38 | -24.89341918 | -58.22738362 | 4 |
| 1406 | POP4 | RPP38 | -24.89341918 | -57.02341082 | 4 |
| 1407 | CACYBP | AGER | -24.89178808 | -56.33904846 | 4 |
| 1408 | HIP1 | HIP1R | -24.89178808 | -53.59631271 | 4 |
| 1409 | GEMIN5 | LSM11 | -24.89178808 | -51.45257681 | 4 |
| 1410 | RGS19 | NGB | -24.89178808 | -45.14714931 | 4 |
| 1411 | PML | RELA | -24.88297117 | -99.49212067 | 10 |
| 1412 | RAC1 | RHOG | -24.8749523 | -81.69474706 | 6 |
| 1413 | RASA1 | CRK | -24.85707077 | -85.75775348 | 8 |
| 1414 | PPARA | AHR | -24.84191308 | -68.96023226 | 6 |
| 1415 | GAB3 | FASLG | -24.83186477 | -45.35342334 | 5 |
| 1416 | LYN | YES1 | -24.82214348 | -84.16246458 | 7 |
| 1417 | PML | MYOD1 | -24.79447707 | -77.61481135 | 8 |
| 1418 | PTPN11 | PTK2B | -24.7830068 | -94.5013452 | 10 |
| 1419 | PRKCA | PRKCB1 | -24.77862567 | -88.47333715 | 7 |
| 1420 | FADD | FAS | -24.77428231 | -79.33065861 | 6 |
| 1421 | GHR | PTK2B | -24.76173309 | -64.85154007 | 7 |
| 1422 | TYK2 | PTK2B | -24.76173309 | -67.64057645 | 7 |
| 1423 | NR3C1 | VDR | -24.755187 | -79.80887479 | 7 |
| 1424 | GRB2 | ERBB3 | -24.7404586 | -97.51612808 | 9 |
| 1425 | FOS | GTF2B | -24.73761718 | -73.54232512 | 7 |
| 1426 | RARA | VDR | -24.73168483 | -65.98428036 | 6 |
| 1427 | ITGA4 | ITGB1 | -24.71946761 | -77.3003528 | 6 |
| 1428 | SYK | CSK | -24.69049061 | -73.84054601 | 7 |
| 1429 | SYK | WAS | -24.69049061 | -70.43267093 | 7 |
| 1430 | COL4A4 | COL1A2 | -24.68068295 | -59.89292886 | 5 |
| 1431 | BAD | CDC25B | -24.67796223 | -54.28692389 | 5 |
| 1432 | ZBTB16 | SIN3A | -24.66646123 | -93.84198732 | 8 |
| 1433 | PIK3R1 | CRK | -24.66485684 | -111.9630924 | 10 |
| 1434 | SRC | PTK2B | -24.66265413 | -112.1405759 | 11 |
| 1435 | GRIN2B | GRIN2C | -24.65555195 | -46.19679995 | 4 |
| 1436 | PTPRC | PTK2B | -24.63698645 | -79.53264334 | 8 |
| 1437 | PTPN11 | ERBB2 | -24.63657185 | -85.60842432 | 9 |
| 1438 | FOSL1 | ATF3 | -24.63363628 | -45.56062162 | 4 |
| 1439 | TRPV4 | NPHS1 | -24.63363628 | -39.44046662 | 4 |
| 1440 | CRHR1 | RIC8 | -24.63363628 | -45.32913479 | 4 |
| 1441 | RGS14 | RIC8 | -24.63363628 | -45.14714931 | 4 |
| 1442 | SLA | SH3BP2 | -24.63363628 | -43.80342031 | 4 |
| 1443 | TNFRSF8 | TNFRSF11A | -24.63363628 | -43.16570148 | 4 |
| 1444 | GAB3 | SCAP1 | -24.63309258 | -34.81500373 | 4 |
| 1445 | ORC4L | ASK | -24.63309258 | -47.15068107 | 4 |
| 1446 | MCM5 | ASK | -24.63309258 | -48.47981702 | 4 |
| 1447 | TGFBI | SERPINE2 | -24.63309258 | -50.17449604 | 4 |
| 1448 | ICAM1 | ICAM3 | -24.63309258 | -50.79663218 | 4 |
| 1449 | RGS16 | RGS14 | -24.63309258 | -45.14714931 | 4 |
| 1450 | GAB1 | PDGFRB | -24.63257564 | -54.66189786 | 6 |
| 1451 | PAG1 | PDGFRB | -24.63257564 | -56.110796 | 6 |
| 1452 | SNX17 | DAB1 | -24.62402739 | -51.93475684 | 4 |
| 1453 | ELK1 | RPS6KA4 | -24.62022097 | -41.11881902 | 4 |
| 1454 | CCL5 | CCL14 | -24.62022097 | -50.19981384 | 4 |
| 1455 | GRIK2 | ATP2B4 | -24.62022097 | -42.38322931 | 4 |
| 1456 | BAX | PMAIP1 | -24.62022097 | -47.68531283 | 4 |
| 1457 | LCP2 | CBL | -24.61255246 | -71.10311298 | 7 |
| 1458 | EPOR | LYN | -24.60330649 | -85.43012648 | 8 |
| 1459 | JUN | SP1 | -24.59061044 | -116.7071903 | 11 |
| 1460 | NCOA1 | GTF2F2 | -24.5829541 | -74.67422969 | 7 |
| 1461 | ABLIM1 | KIAA0408 | -24.54676711 | -52.99620046 | 5 |
| 1462 | GNAI2 | GNA15 | -24.54390645 | -83.28764685 | 6 |
| 1463 | TNFRSF1A | MAP3K14 | -24.54390645 | -65.67439965 | 6 |
| 1464 | TNFRSF1A | CFLAR | -24.54390645 | -67.83340301 | 6 |
| 1465 | RXRA | NR1H3 | -24.54110407 | -59.47646506 | 5 |
| 1466 | MAP3K14 | CFLAR | -24.53268509 | -53.53299187 | 5 |
| 1467 | JAK3 | SHC1 | -24.49397353 | -94.67720752 | 8 |
| 1468 | BRCA1 | STAT3 | -24.48503407 | -100.5578952 | 10 |
| 1469 | MST1R | PDGFRB | -24.48012777 | -43.70632075 | 5 |
| 1470 | CDC7 | MCM2 | -24.47864616 | -64.14523456 | 5 |
| 1471 | GRB2 | RASA1 | -24.47253264 | -129.3263365 | 12 |
| 1472 | RAF1 | MAP2K1 | -24.46610491 | -88.14472927 | 7 |
| 1473 | NCOR2 | NCOA1 | -24.46592028 | -86.30423326 | 8 |
| 1474 | RIPK1 | FADD | -24.4417623 | -74.77678172 | 6 |
| 1475 | RICS | NEDD9 | -24.44064545 | -62.48955825 | 6 |
| 1476 | KIT | ERBB2 | -24.43989445 | -75.46085286 | 7 |
| 1477 | EGFR | RICS | -24.43819241 | -86.19666722 | 8 |
| 1478 | HSPCA | BAG1 | -24.42450068 | -78.03907762 | 7 |
| 1479 | NCOR1 | HDAC9 | -24.41842264 | -68.69411231 | 6 |
| 1480 | KIAA0980 | Cep70 | -24.41842264 | -81.1169029 | 6 |
| 1481 | BCAR1 | SHC1 | -24.41194249 | -96.73114343 | 9 |
| 1482 | SMAD3 | SMAD1 | -24.40831894 | -127.1157461 | 10 |
| 1483 | TUBA1 | TUBA8 | -24.40369308 | -49.71855051 | 4 |
| 1484 | KIT | GAB3 | -24.40138468 | -46.93489462 | 5 |
| 1485 | SMN1 | SNRPD3 | -24.40138468 | -64.36549239 | 5 |
| 1486 | TRIM37 | TNFRSF11A | -24.40138468 | -53.47486974 | 5 |
| 1487 | CDC45L | CDC5L | -24.38286559 | -47.13266257 | 4 |
| 1488 | RAD23B | RAD23A | -24.38286559 | -60.37623461 | 4 |
| 1489 | PCBD1 | RPIA | -24.38069064 | -43.28216733 | 4 |
| 1490 | CD8A | LILRB1 | -24.38069064 | -52.84332552 | 4 |
| 1491 | HOXA1 | AQP1 | -24.38069064 | -40.26988446 | 4 |
| 1492 | CTNNB1 | CDH2 | -24.37329171 | -93.51018594 | 7 |
| 1493 | AR | YWHAH | -24.35178582 | -96.2479799 | 9 |
| 1494 | FN1 | FBLN2 | -24.33052168 | -91.19779327 | 7 |
| 1495 | EXOC8 | EXOC7 | -24.32992848 | -56.62570246 | 5 |
| 1496 | NRIP1 | PPARBP | -24.30702379 | -63.92729528 | 6 |
| 1497 | JAK1 | SRC | -24.30033484 | -101.0756654 | 10 |
| 1498 | CD22 | PTK2B | -24.27950001 | -55.28788369 | 6 |
| 1499 | SAP30 | HDAC2 | -24.27089128 | -71.78430882 | 6 |
| 1500 | PIK3R1 | PTPN1 | -24.25974438 | -83.80726863 | 8 |
| 1501 | SAA1 | C1QR1 | -24.25643867 | -50.17449604 | 4 |
| 1502 | PCTK1 | TNFAIP3 | -24.25643867 | -43.8559258 | 4 |
| 1503 | REM1 | TNFAIP3 | -24.25643867 | -42.31369664 | 4 |
| 1504 | TNFAIP3 | KIF1C | -24.25208819 | -43.6682423 | 4 |
| 1505 | PTK2B | CTLA4 | -24.24841822 | -56.25431557 | 6 |
| 1506 | HDAC2 | SIN3B | -24.24472429 | -72.48929046 | 6 |
| 1507 | CTLA4 | PECAM1 | -24.24220279 | -46.47896246 | 5 |
| 1508 | ABLIM1 | FLJ32855 | -24.24220279 | -52.68411615 | 5 |
| 1509 | GHR | PRLR | -24.23676061 | -63.38437247 | 5 |
| 1510 | NCOA3 | NR0B2 | -24.23676061 | -52.1149819 | 5 |
| 1511 | PAK1 | PAK2 | -24.23537032 | -56.15202783 | 5 |
| 1512 | SRC | CRK | -24.22226293 | -109.1346241 | 10 |
| 1513 | EGFR | CRK | -24.22226293 | -111.1759334 | 10 |
| 1514 | NR3C1 | PPARA | -24.21567516 | -88.50606725 | 8 |
| 1515 | RXRA | THRA | -24.1975393 | -90.33951222 | 7 |
| 1516 | MAP3K1 | TNFAIP3 | -24.19395213 | -51.60982001 | 5 |
| 1517 | SUV39H1 | DNMT1 | -24.19395213 | -54.47154685 | 5 |
| 1518 | PLCG1 | RASA1 | -24.17877485 | -94.21317424 | 9 |
| 1519 | IRS2 | IGF1R | -24.15245968 | -58.22538042 | 6 |
| 1520 | PTPRC | LCK | -24.13481648 | -96.1129006 | 8 |
| 1521 | BCR | CSF2RB | -24.12776533 | -47.67920353 | 5 |
| 1522 | RELA | TP53 | -24.10406032 | -159.9835658 | 14 |
| 1523 | GRB2 | RICS | -24.06766696 | -96.69704504 | 9 |
| 1524 | GRB2 | GRB10 | -24.06766696 | -102.4799307 | 9 |
| 1525 | JAK2 | BCAR1 | -24.0594824 | -79.45328524 | 8 |
| 1526 | KIT | PECAM1 | -24.05666546 | -56.98913133 | 6 |
| 1527 | POU2F1 | NCOA3 | -24.05666546 | -60.01663543 | 6 |
| 1528 | PTPRC | ZAP70 | -24.04709153 | -74.10373896 | 7 |
| 1529 | VLDLR | LDLR | -24.04625764 | -54.41194144 | 4 |
| 1530 | LRP8 | LDLR | -24.04625764 | -54.41194144 | 4 |
| 1531 | SMARCA2 | SMARCE1 | -24.04625764 | -41.60309879 | 4 |
| 1532 | TRPC4AP | TRAF3IP2 | -24.04625764 | -42.72661976 | 4 |
| 1533 | THAP7 | C1orf65 | -24.04625764 | -39.31431488 | 4 |
| 1534 | ADAM15 | TUB | -24.04517017 | -36.3419658 | 4 |
| 1535 | ADAM15 | SCAP1 | -24.04517017 | -35.06991483 | 4 |
| 1536 | ADAM15 | TRPV4 | -24.04517017 | -38.94947997 | 4 |
| 1537 | BIRC5 | BIRC3 | -24.04517017 | -49.65506193 | 4 |
| 1538 | RGS19 | RGS14 | -24.04517017 | -45.14714931 | 4 |
| 1539 | USP7 | TNFRSF8 | -24.04517017 | -43.16570148 | 4 |
| 1540 | TRIM28 | TIF1 | -24.03171122 | -64.22437083 | 5 |
| 1541 | JUN | SPIB | -24.02816652 | -62.91090459 | 6 |
| 1542 | EPOR | INSR | -24.01941791 | -72.93690301 | 7 |
| 1543 | CBLB | MAP4K1 | -24.00477906 | -48.50128451 | 5 |
| 1544 | GTF2H1 | CCNH | -24.00477906 | -60.67640132 | 5 |
| 1545 | ERBB2 | MST1R | -23.98436492 | -43.70632075 | 5 |
| 1546 | CDKN1B | CDKN1A | -23.97095667 | -71.36903182 | 6 |
| 1547 | VAV1 | VAV3 | -23.96186285 | -58.89291317 | 6 |
| 1548 | RIBC2 | KIAA1267 | -23.94921529 | -47.91954804 | 5 |
| 1549 | ORC4L | MCM5 | -23.9404894 | -47.87368122 | 4 |
| 1550 | ACTR3 | ACTR2 | -23.9404894 | -56.52875013 | 4 |
| 1551 | APEX2 | ZNF426 | -23.9404894 | -40.25123544 | 4 |
| 1552 | ID2 | ID3 | -23.9404894 | -48.46319463 | 4 |
| 1553 | LAT | ZAP70 | -23.9388497 | -72.10894098 | 6 |
| 1554 | DLG2 | CASK | -23.9388497 | -74.31212506 | 6 |
| 1555 | LYN | SHC1 | -23.93337796 | -133.6102907 | 11 |
| 1556 | NSD1 | NR0B2 | -23.93115254 | -38.6236474 | 4 |
| 1557 | ABLIM1 | USP2 | -23.92843367 | -42.32702947 | 4 |
| 1558 | ZAP70 | PLCG2 | -23.92740789 | -65.5563628 | 6 |
| 1559 | CREBBP | ESR1 | -23.92112602 | -136.7133675 | 12 |
| 1560 | FLT4 | KDR | -23.92010252 | -50.89009836 | 4 |
| 1561 | KTN1 | PLD1 | -23.92010252 | -43.17136697 | 4 |
| 1562 | APOE | SNX17 | -23.91520789 | -51.93475684 | 4 |
| 1563 | MAP3K14 | TNFRSF19 | -23.91520789 | -40.87083844 | 4 |
| 1564 | ZNF250 | UTP14A | -23.91520789 | -42.68049723 | 4 |
| 1565 | ITGB2 | ITGB1 | -23.8557277 | -84.31872962 | 7 |
| 1566 | MAPK10 | MAPK8 | -23.85416939 | -64.45407627 | 5 |
| 1567 | ABL1 | LCK | -23.85324646 | -106.9514294 | 9 |
| 1568 | ERBB2 | PTK2B | -23.84513642 | -75.46901979 | 8 |
| 1569 | PPARA | THRB | -23.83767614 | -75.11437953 | 6 |
| 1570 | FADD | TRADD | -23.82403081 | -77.17133809 | 6 |
| 1571 | TAF1 | GTF2F2 | -23.81645964 | -67.20081212 | 6 |
| 1572 | ESR1 | THRB | -23.81483927 | -92.67116521 | 8 |
| 1573 | JAK1 | EPOR | -23.8080533 | -69.88274745 | 7 |
| 1574 | C6orf55 | LNX | -23.80186616 | -73.80822523 | 6 |
| 1575 | RASA1 | NCK1 | -23.79202697 | -95.30489805 | 8 |
| 1576 | WASF2 | WAS | -23.7872702 | -52.32643753 | 5 |
| 1577 | CTSG | F2 | -23.7872702 | -64.08056543 | 5 |
| 1578 | CDC5L | MCM2 | -23.7872702 | -62.73204728 | 5 |
| 1579 | PTPN11 | INPP5D | -23.78465897 | -67.77992396 | 6 |
| 1580 | TRAF2 | IKBKB | -23.77200994 | -113.1840462 | 9 |
| 1581 | MAP3K14 | RIPK2 | -23.75497785 | -52.07437685 | 5 |
| 1582 | PTK2 | PECAM1 | -23.74470144 | -68.91630872 | 7 |
| 1583 | TBP | GTF2F2 | -23.74470144 | -81.44677149 | 7 |
| 1584 | RIPK1 | CASP8AP2 | -23.73121789 | -42.36210694 | 4 |
| 1585 | GAB2 | PAG1 | -23.72366207 | -47.40381459 | 5 |
| 1586 | HSPG2 | NID | -23.72366207 | -64.25137909 | 5 |
| 1587 | BRCA1 | PTMA | -23.72019014 | -83.21015398 | 8 |
| 1588 | IL2RB | EPOR | -23.708575 | -61.64117716 | 6 |
| 1589 | RET | KIT | -23.708575 | -64.53731268 | 6 |
| 1590 | GAB3 | SHC1 | -23.70766979 | -55.64576093 | 6 |
| 1591 | TP53 | E2F1 | -23.70063331 | -131.2662393 | 10 |
| 1592 | PAG1 | INSR | -23.69658904 | -60.22718037 | 6 |
| 1593 | HDAC3 | SAP30 | -23.69175727 | -65.80087645 | 6 |
| 1594 | SYK | CD19 | -23.68460077 | -64.44486962 | 6 |
| 1595 | SIN3A | ZNFN1A1 | -23.67860415 | -71.15750831 | 6 |
| 1596 | CCR10 | CCBP2 | -23.61008369 | -61.60837829 | 4 |
| 1597 | BRCA1 | SP3 | -23.60593228 | -61.4929776 | 6 |
| 1598 | CREBBP | PCAF | -23.6048129 | -103.5661858 | 8 |
| 1599 | GRIN2A | KCNA4 | -23.60464484 | -44.7469422 | 4 |
| 1600 | GRIN2A | ATP2B4 | -23.60464484 | -43.61261183 | 4 |
| 1601 | GRIN2A | GUCY1A2 | -23.60464484 | -43.61261183 | 4 |
| 1602 | RBBP4 | ARID4A | -23.60464484 | -38.26868238 | 4 |
| 1603 | KCNJ12 | GUCY1A2 | -23.60464484 | -43.61261183 | 4 |
| 1604 | TU3A | C10orf10 | -23.60464484 | -41.55021582 | 4 |
| 1605 | TU3A | UTP14A | -23.60464484 | -42.68049723 | 4 |
| 1606 | TU3A | RHPN1 | -23.60464484 | -42.56754486 | 4 |
| 1607 | PIK3R1 | BCAR1 | -23.60138032 | -95.22259602 | 9 |
| 1608 | EPOR | INPP5D | -23.59345051 | -46.35396905 | 5 |
| 1609 | CSF1R | PTK2B | -23.59140367 | -55.36129779 | 6 |
| 1610 | SOCS1 | SHC1 | -23.58104925 | -86.66626996 | 8 |
| 1611 | FOS | FOSL2 | -23.54734488 | -57.88802068 | 5 |
| 1612 | DDX54 | TIF1 | -23.53979639 | -43.33769985 | 4 |
| 1613 | MCM10 | MCM2 | -23.5378506 | -74.73803257 | 6 |
| 1614 | MYF5 | MYOG | -23.5358401 | -43.68391302 | 4 |
| 1615 | TRPV4 | UNC119 | -23.5358401 | -39.90867119 | 4 |
| 1616 | NRAS | KRAS | -23.5358401 | -55.78111476 | 4 |
| 1617 | TMPO | EMD | -23.5358401 | -60.8687111 | 4 |
| 1618 | SDC1 | SDC4 | -23.53420878 | -48.90777841 | 4 |
| 1619 | CD8A | LILRB2 | -23.53420878 | -52.84332552 | 4 |
| 1620 | TNFRSF1B | TNFRSF8 | -23.53420878 | -47.65124318 | 4 |
| 1621 | FLJ20626 | SCAND1 | -23.53420878 | -56.59204498 | 4 |
| 1622 | DNASE1 | TMSB4X | -23.53379937 | -38.47117048 | 3 |
| 1623 | APH1B | APH1A | -23.53379937 | -37.02446699 | 3 |
| 1624 | APH1B | PSENEN | -23.53379937 | -37.02446699 | 3 |
| 1625 | ASCL2 | EMP3 | -23.53379937 | -32.95758362 | 3 |
| 1626 | ASCL2 | KCNQ2 | -23.53379937 | -32.95758362 | 3 |
| 1627 | PPEF2 | EMP3 | -23.53379937 | -32.95758362 | 3 |
| 1628 | PPEF2 | KCNQ2 | -23.53379937 | -32.95758362 | 3 |
| 1629 | PPEF1 | EMP3 | -23.53379937 | -32.95758362 | 3 |
| 1630 | PPEF1 | KCNQ2 | -23.53379937 | -32.95758362 | 3 |
| 1631 | CSTA | CSTB | -23.53379937 | -43.95449099 | 3 |
| 1632 | EDG5 | EDG3 | -23.53379937 | -33.0245504 | 3 |
| 1633 | TACR2 | TACR1 | -23.53379937 | -46.15171557 | 3 |
| 1634 | ADRA2A | ADRA2C | -23.53339179 | -36.06112623 | 3 |
| 1635 | RAMP3 | RAMP2 | -23.53339179 | -45.40977822 | 3 |
| 1636 | MYF6 | ASCL2 | -23.53339179 | -32.95758362 | 3 |
| 1637 | MYF6 | PPEF2 | -23.53339179 | -32.95758362 | 3 |
| 1638 | MYF6 | PPEF1 | -23.53339179 | -32.95758362 | 3 |
| 1639 | CLTA | CLTB | -23.53339179 | -39.3493208 | 3 |
| 1640 | GRIN2C | GRIN2D | -23.53339179 | -34.2235727 | 3 |
| 1641 | IGFBP4 | IGFBP1 | -23.53339179 | -38.99164636 | 3 |
| 1642 | IGFBP4 | IGFBP2 | -23.53339179 | -38.99164636 | 3 |
| 1643 | IGFBP4 | IGFBP6 | -23.53339179 | -38.99164636 | 3 |
| 1644 | LTBP3 | SLITL2 | -23.53339179 | -37.19786872 | 3 |
| 1645 | LTBP3 | FMOD | -23.53339179 | -37.19786872 | 3 |
| 1646 | TAC3 | TAC4 | -23.53339179 | -47.35568837 | 3 |
| 1647 | WNT1 | WNT4 | -23.53339179 | -47.25032785 | 3 |
| 1648 | DLL1 | JAG2 | -23.53339179 | -38.70966231 | 3 |
| 1649 | STK36 | ZIC1 | -23.53339179 | -43.44366536 | 3 |
| 1650 | STK36 | ZIC2 | -23.53339179 | -43.44366536 | 3 |
| 1651 | RFC3 | RFC5 | -23.53339179 | -36.24466769 | 3 |
| 1652 | FANCE | FANCF | -23.53339179 | -39.72401425 | 3 |
| 1653 | KCNJ10 | GRIN2D | -23.53339179 | -34.2235727 | 3 |
| 1654 | PGF | SEMA3F | -23.53339179 | -41.70984893 | 3 |
| 1655 | PGF | VEGFB | -23.53339179 | -40.45708596 | 3 |
| 1656 | MGC2560 | NEU4 | -23.53339179 | -30.75502354 | 3 |
| 1657 | TACR1 | TACR3 | -23.53339179 | -46.15171557 | 3 |
| 1658 | APP | DCN | -23.53106201 | -87.10143055 | 7 |
| 1659 | PTK2B | SOCS1 | -23.53106201 | -67.52043405 | 7 |
| 1660 | MLLT7 | FOXO1A | -23.52595954 | -35.25633433 | 4 |
| 1661 | BMP2 | GDF5 | -23.52269668 | -45.50584816 | 4 |
| 1662 | CCL5 | CCL2 | -23.52269668 | -51.65877081 | 4 |
| 1663 | ORC1L | ORC5L | -23.52269668 | -48.60596831 | 4 |
| 1664 | C1QA | C1QB | -23.52269668 | -61.76252897 | 4 |
| 1665 | SNRPE | SNRPF | -23.52269668 | -52.27641043 | 4 |
| 1666 | BAX | BCL2L11 | -23.52269668 | -47.01018416 | 4 |
| 1667 | BATF | HLF | -23.52269668 | -50.454798 | 4 |
| 1668 | BATF | NFE2L1 | -23.52269668 | -45.78109494 | 4 |
| 1669 | NCOR2 | RXRA | -23.51266515 | -86.22487875 | 8 |
| 1670 | GAB3 | PDGFRB | -23.50229666 | -43.29673878 | 5 |
| 1671 | IL2RB | SRC | -23.48675028 | -79.68467041 | 8 |
| 1672 | RET | EGFR | -23.48675028 | -86.63226127 | 8 |
| 1673 | JAK2 | PDGFRB | -23.48644457 | -75.25282107 | 8 |
| 1674 | PTPN6 | TYK2 | -23.48642825 | -74.80311499 | 7 |
| 1675 | KPNA2 | KPNB1 | -23.46932342 | -75.79619173 | 6 |
| 1676 | APP | HABP2 | -23.45486134 | -63.8657166 | 5 |
| 1677 | PTPN11 | SOCS2 | -23.45305872 | -57.14978734 | 5 |
| 1678 | SYK | KIT | -23.42141426 | -68.46255049 | 7 |
| 1679 | SYK | NEDD9 | -23.42141426 | -68.90340002 | 7 |
| 1680 | TIF1 | NR0B2 | -23.41972602 | -50.2961205 | 5 |
| 1681 | HMGB2 | HMGB1 | -23.41770433 | -62.15660628 | 5 |
| 1682 | CD28 | LAT | -23.40789706 | -52.16315956 | 5 |
| 1683 | CEBPG | JUNB | -23.40585611 | -65.40819301 | 5 |
| 1684 | PRKCD | PRKCG | -23.39660427 | -68.36998063 | 5 |
| 1685 | YWHAE | YWHAG | -23.3892123 | -71.85073662 | 6 |
| 1686 | TYK2 | IGF1R | -23.37241794 | -64.58018403 | 6 |
| 1687 | PTPN12 | FYN | -23.3693886 | -73.55756294 | 7 |
| 1688 | LYN | LCP2 | -23.36082089 | -75.9337209 | 7 |
| 1689 | TGFBI | OSM | -23.3532468 | -50.17449604 | 4 |
| 1690 | RGS16 | RGS7 | -23.3532468 | -45.32913479 | 4 |
| 1691 | RGS16 | RGS4 | -23.3532468 | -44.18759745 | 4 |
| 1692 | LTBR | USP7 | -23.3532468 | -44.74388685 | 4 |
| 1693 | SYTL4 | RPH3AL | -23.3532468 | -54.96198778 | 4 |
| 1694 | ZNF426 | C1orf65 | -23.3532468 | -36.13533555 | 4 |
| 1695 | CDC7 | ORC4L | -23.35270303 | -47.87368122 | 4 |
| 1696 | CDC7 | MCM5 | -23.35270303 | -47.87368122 | 4 |
| 1697 | GRB14 | SNX4 | -23.35270303 | -42.31723521 | 4 |
| 1698 | RGS7 | RIC8 | -23.35270303 | -45.32913479 | 4 |
| 1699 | RGS19 | RIC8 | -23.35270303 | -45.14714931 | 4 |
| 1700 | RGS4 | RIC8 | -23.35270303 | -44.18759745 | 4 |
| 1701 | RRAS | MRAS | -23.35270303 | -52.12770549 | 4 |
| 1702 | TRAF3IP2 | TNFRSF11A | -23.35270303 | -41.27234314 | 4 |
| 1703 | RRAS2 | RAP1A | -23.35033064 | -59.16663462 | 5 |
| 1704 | SNIP1 | LEF1 | -23.35033064 | -51.71454262 | 5 |
| 1705 | ERBB4 | KCNJ12 | -23.34965032 | -56.72016945 | 5 |
| 1706 | NCOA1 | NCOA4 | -23.3489541 | -53.7507733 | 5 |
| 1707 | CASP8AP2 | TRADD | -23.34874115 | -42.36210694 | 4 |
| 1708 | MCM4 | MCM2 | -23.34874115 | -48.52633704 | 4 |
| 1709 | ARF1 | ARF5 | -23.33840241 | -55.90459582 | 4 |
| 1710 | EPS15 | EPS15L1 | -23.33840241 | -61.65716846 | 4 |
| 1711 | PLCG1 | SHB | -23.33191843 | -73.81830604 | 7 |
| 1712 | PTPN11 | RASA1 | -23.32375382 | -90.36426859 | 9 |
| 1713 | XPOT | TNPO1 | -23.32253774 | -49.89951331 | 4 |
| 1714 | SOCS2 | SOCS3 | -23.32253774 | -47.37443423 | 4 |
| 1715 | ZNF8 | LEF1 | -23.32253774 | -36.88999119 | 4 |
| 1716 | TNFRSF19 | RIPK2 | -23.32253774 | -42.22076515 | 4 |
| 1717 | PDGFRB | CBLB | -23.31853301 | -58.30139741 | 6 |
| 1718 | PDGFRB | SHB | -23.31853301 | -57.39192697 | 6 |
| 1719 | PDGFRB | RICS | -23.31853301 | -56.64684987 | 6 |
| 1720 | ERBB2IP | ZNF8 | -23.3165546 | -36.88999119 | 4 |
| 1721 | BAD | PMAIP1 | -23.3165546 | -47.68531283 | 4 |
| 1722 | SUV39H1 | ARID4A | -23.3165546 | -38.26868238 | 4 |
| 1723 | ERBB4 | ATP2B4 | -23.3165546 | -43.61261183 | 4 |
| 1724 | ERBB4 | GUCY1A2 | -23.3165546 | -43.61261183 | 4 |
| 1725 | SYK | SCAP1 | -23.27579363 | -44.73997236 | 5 |
| 1726 | LRP2 | VLDLR | -23.27579363 | -67.17012342 | 5 |
| 1727 | LRP2 | LRP8 | -23.27579363 | -67.68094905 | 5 |
| 1728 | NUP62 | RAN | -23.23943255 | -74.6362417 | 6 |
| 1729 | TRAF2 | HOOK2 | -23.23782754 | -102.4417438 | 8 |
| 1730 | NR3C1 | RXRA | -23.22430205 | -108.4121025 | 10 |
| 1731 | LNX | ZNF408 | -23.22276624 | -67.51314065 | 6 |
| 1732 | CEBPA | SMAD3 | -23.21878857 | -73.32100815 | 7 |
| 1733 | FLJ32855 | PSMF1 | -23.21606919 | -53.8037985 | 5 |
| 1734 | SMAD4 | SMAD1 | -23.2031064 | -129.1303955 | 10 |
| 1735 | EGFR | IGF1R | -23.20309925 | -100.477519 | 9 |
| 1736 | CSK | PTK2B | -23.19872451 | -69.70605352 | 7 |
| 1737 | GRM7 | SIAH1 | -23.18290585 | -67.33226917 | 5 |
| 1738 | SYK | BLNK | -23.17841299 | -63.09101983 | 6 |
| 1739 | CSK | SHC1 | -23.16292411 | -86.69373807 | 8 |
| 1740 | DLG3 | CASK | -23.15908119 | -73.16699276 | 6 |
| 1741 | SNRPF | LSM2 | -23.15850691 | -51.4189602 | 4 |
| 1742 | NFE2 | NFE2L2 | -23.15850691 | -50.86731859 | 4 |
| 1743 | ACVR1B | IGSF1 | -23.15469997 | -48.98560786 | 4 |
| 1744 | CD2AP | TYRO3 | -23.15469997 | -38.56777789 | 4 |
| 1745 | CASP7 | DIABLO | -23.15469997 | -52.85437536 | 4 |
| 1746 | NFE2L2 | NFE2L1 | -23.15469997 | -51.81025126 | 4 |
| 1747 | NCOA1 | TBP | -23.15342955 | -96.20190185 | 9 |
| 1748 | NFKB1 | TBP | -23.15342955 | -98.74347488 | 9 |
| 1749 | ZAP70 | CRKL | -23.15286562 | -75.65318851 | 7 |
| 1750 | YWHAH | YWHAQ | -23.14927259 | -77.58435041 | 6 |
| 1751 | PTK2 | CBL | -23.14604645 | -87.58312028 | 9 |
| 1752 | CTBP1 | SIN3B | -23.14060078 | -57.95461648 | 5 |
| 1753 | ATF4 | BATF | -23.14060078 | -57.92296484 | 5 |
| 1754 | CTNND1 | CTNNB1 | -23.13491057 | -95.33295983 | 7 |
| 1755 | ERBB2 | PDGFRB | -23.11895607 | -62.19254017 | 7 |
| 1756 | COL1A1 | COL4A5 | -23.10809374 | -59.21780018 | 5 |
| 1757 | COL1A1 | COL4A6 | -23.10809374 | -59.21780018 | 5 |
| 1758 | CPSF1 | TAF15 | -23.08426311 | -56.93866959 | 4 |
| 1759 | DNAJB1 | HSPA4 | -23.08426311 | -52.47708113 | 4 |
| 1760 | BAHD1 | C16orf48 | -23.08426311 | -39.27801568 | 4 |
| 1761 | EPHB1 | EPHB2 | -23.08426311 | -51.55560904 | 4 |
| 1762 | CD22 | MST1R | -23.08208787 | -34.99545444 | 4 |
| 1763 | WWP2 | NEDD4L | -23.08208787 | -54.33289824 | 4 |
| 1764 | LCK | CRKL | -23.08033884 | -82.24602747 | 8 |
| 1765 | POU2F1 | TIF1 | -23.05365761 | -61.25731196 | 6 |
| 1766 | ACVR1 | SMAD2 | -23.05054554 | -146.0877634 | 10 |
| 1767 | KHDRBS1 | CBL | -23.05009191 | -69.18494225 | 7 |
| 1768 | SOS1 | CBL | -23.05009191 | -72.30293208 | 7 |
| 1769 | CEBPG | TEF | -23.04788238 | -53.07491813 | 4 |
| 1770 | ORC2L | ORC3L | -23.04788238 | -55.24535019 | 4 |
| 1771 | EXOC8 | C10orf10 | -23.04788238 | -41.55021582 | 4 |
| 1772 | ADAM15 | BCAR1 | -23.03906323 | -47.94474452 | 5 |
| 1773 | BLNK | LAT | -23.00338616 | -53.13653438 | 5 |
| 1774 | GAB1 | IRS2 | -23.00338616 | -46.00509877 | 5 |
| 1775 | GRIN2B | KCNJ12 | -23.00202543 | -60.52108353 | 5 |
| 1776 | EXOC8 | TU3A | -23.00202543 | -56.36252153 | 5 |
| 1777 | AR | GTF2B | -23.00192209 | -90.77709389 | 8 |
| 1778 | CD2AP | PTK2B | -22.97741599 | -54.98605446 | 6 |
| 1779 | IRS2 | CRKL | -22.9735199 | -58.03944117 | 6 |
| 1780 | GRB2 | KIT | -22.95874031 | -106.6309465 | 10 |
| 1781 | PTK2B | CD5 | -22.94796607 | -59.85952612 | 6 |
| 1782 | RAF1 | RASIP1 | -22.94623143 | -62.60927786 | 5 |
| 1783 | ABL1 | CRK | -22.94402064 | -94.31074144 | 8 |
| 1784 | PDGFRB | PECAM1 | -22.94258998 | -57.3348074 | 6 |
| 1785 | MCM10 | KIAA1267 | -22.94126234 | -51.53636396 | 5 |
| 1786 | PPARA | PPARD | -22.94126234 | -60.08077945 | 5 |
| 1787 | GRB2 | ITK | -22.91201558 | -91.64463696 | 8 |
| 1788 | KIT | IRS4 | -22.90329979 | -46.22744345 | 5 |
| 1789 | EPOR | IRS4 | -22.90329979 | -46.22744345 | 5 |
| 1790 | TGFBR1 | SMURF2 | -22.90028618 | -151.3881097 | 11 |
| 1791 | PTPN6 | FYN | -22.88305471 | -117.5388175 | 11 |
| 1792 | PLCG1 | BTK | -22.8800406 | -77.89971858 | 7 |
| 1793 | EGFR | ZAP70 | -22.87195324 | -93.09952894 | 9 |
| 1794 | ZBTB16 | HDAC9 | -22.86685999 | -67.60121148 | 6 |
| 1795 | MAPK8 | ATF3 | -22.85781848 | -50.6350462 | 5 |
| 1796 | ORC4L | CDC6 | -22.84296532 | -48.49441582 | 4 |
| 1797 | ORC4L | CDC5L | -22.84296532 | -48.94936099 | 4 |
| 1798 | OPRD1 | OPRM1 | -22.8418777 | -51.11442249 | 4 |
| 1799 | WASF2 | WASF1 | -22.8418777 | -40.35321028 | 4 |
| 1800 | ARNTL | PER1 | -22.8418777 | -51.61435384 | 4 |
| 1801 | HOXA1 | ZNF426 | -22.8418777 | -42.87257415 | 4 |
| 1802 | TNFRSF14 | LTBR | -22.8418777 | -49.8047927 | 4 |
| 1803 | SNRPB | SNRPD3 | -22.8418777 | -52.88254624 | 4 |
| 1804 | JUN | CSNK2A1 | -22.82309359 | -99.80328364 | 9 |
| 1805 | NR2F6 | PPARGC1A | -22.82230685 | -40.03366621 | 4 |
| 1806 | FGF7 | SPARC | -22.82230685 | -49.69332662 | 4 |
| 1807 | NXF1 | NXF2 | -22.81795578 | -56.51200227 | 4 |
| 1808 | PPARGC1A | THRAP4 | -22.81795578 | -42.16927198 | 4 |
| 1809 | RAD9A | RAD1 | -22.81795578 | -57.02568096 | 4 |
| 1810 | TRAF1 | IKBKB | -22.8138616 | -70.12837128 | 6 |
| 1811 | TRAF1 | IKBKG | -22.8138616 | -73.49195333 | 6 |
| 1812 | SAA1 | FBLN2 | -22.80323162 | -50.17449604 | 4 |
| 1813 | GIT2 | GIT1 | -22.80323162 | -50.35230091 | 4 |
| 1814 | MAP2K1 | KSR2 | -22.79615967 | -44.02281861 | 4 |
| 1815 | KIAA0408 | MGC10854 | -22.79615967 | -40.3510623 | 4 |
| 1816 | CCND1 | CCNA2 | -22.79600805 | -71.81954062 | 6 |
| 1817 | IFNAR1 | GHR | -22.77954533 | -49.87458022 | 5 |
| 1818 | CD19 | TYK2 | -22.77954533 | -47.37649883 | 5 |
| 1819 | PPARGC1A | PPARBP | -22.77954533 | -53.84174507 | 5 |
| 1820 | PSMA1 | FLJ32855 | -22.77954533 | -54.02493223 | 5 |
| 1821 | ZNF250 | FLJ32855 | -22.77954533 | -54.08522596 | 5 |
| 1822 | PTPN1 | PTPRF | -22.77614327 | -51.73252624 | 5 |
| 1823 | PECAM1 | CD36 | -22.77614327 | -51.64270254 | 5 |
| 1824 | PTK2 | LCK | -22.77474479 | -93.10628505 | 9 |
| 1825 | LRP1 | VLDLR | -22.7731087 | -69.22424715 | 5 |
| 1826 | CSF3R | INPP5D | -22.7655965 | -35.68479806 | 4 |
| 1827 | RGS7 | RGS19 | -22.7655965 | -47.72904822 | 4 |
| 1828 | RRAS | RAP2A | -22.7655965 | -52.12770549 | 4 |
| 1829 | DGKZ | DTNA | -22.7655965 | -53.24433628 | 4 |
| 1830 | CSK | LCK | -22.76225614 | -81.64327347 | 7 |
| 1831 | KHDRBS1 | HNRPK | -22.75751141 | -65.65341545 | 6 |
| 1832 | LSM1 | SMN1 | -22.75751141 | -76.31686787 | 6 |
| 1833 | TU3A | LNX | -22.73988795 | -57.00922874 | 6 |
| 1834 | LCK | HCK | -22.73455603 | -83.46470413 | 7 |
| 1835 | PTK2 | ILK | -22.71877472 | -66.81763796 | 6 |
| 1836 | GRB7 | PLCG1 | -22.70827825 | -60.03645631 | 6 |
| 1837 | FRS2 | PLCG1 | -22.70827825 | -67.68679773 | 6 |
| 1838 | BRCA1 | MTA1 | -22.69852078 | -63.3871508 | 6 |
| 1839 | GAB1 | SHB | -22.67932616 | -48.0077161 | 5 |
| 1840 | PAG1 | CBLB | -22.67932616 | -48.26150114 | 5 |
| 1841 | MST1R | VAV3 | -22.67920619 | -34.99545444 | 4 |
| 1842 | AKAP8 | CDK6 | -22.67920619 | -48.17017947 | 4 |
| 1843 | FOSL1 | BATF | -22.67920619 | -45.56062162 | 4 |
| 1844 | HIPK2 | SNIP1 | -22.67728493 | -52.42230705 | 5 |
| 1845 | MDK | PTN | -22.67648696 | -55.08796758 | 4 |
| 1846 | CCL5 | CCL7 | -22.67648696 | -50.19981384 | 4 |
| 1847 | SYN1 | SLC9A2 | -22.67648696 | -39.73161528 | 4 |
| 1848 | TRA@ | CD3D | -22.67648696 | -51.43659174 | 4 |
| 1849 | ORC1L | ASK | -22.67648696 | -48.35465388 | 4 |
| 1850 | TGFB2 | TGFBR2 | -22.66271587 | -59.2400903 | 5 |
| 1851 | RIPK1 | CASP10 | -22.65727221 | -57.56489865 | 5 |
| 1852 | DPPA2 | ZBTB8 | -22.62964705 | -56.40487428 | 5 |
| 1853 | CEBPG | JUND | -22.62896664 | -63.01029773 | 5 |
| 1854 | JAK1 | LCK | -22.62767445 | -82.28191832 | 8 |
| 1855 | ASCL2 | KCNQ5 | -22.61750864 | -32.95758362 | 3 |
| 1856 | ASCL2 | GRM5 | -22.61750864 | -32.95758362 | 3 |
| 1857 | PPEF2 | KCNQ5 | -22.61750864 | -32.95758362 | 3 |
| 1858 | PPEF2 | GRM5 | -22.61750864 | -32.95758362 | 3 |
| 1859 | IGSF8 | PTGFRN | -22.61750864 | -37.60885463 | 3 |
| 1860 | BIK | PMAIP1 | -22.61750864 | -34.15394096 | 3 |
| 1861 | BIK | BID | -22.61750864 | -34.15394096 | 3 |
| 1862 | PTPN7 | DUSP1 | -22.61750864 | -30.5592851 | 3 |
| 1863 | JAG2 | JAG1 | -22.61750864 | -38.70966231 | 3 |
| 1864 | GDA | ATP2B4 | -22.61750864 | -31.41037591 | 3 |
| 1865 | GDA | GUCY1A2 | -22.61750864 | -31.41037591 | 3 |
| 1866 | AP1S2 | AFTIPHILIN | -22.61750864 | -40.93813943 | 3 |
| 1867 | ATP2B2 | GUCY1A2 | -22.61750864 | -31.63938458 | 3 |
| 1868 | CCL3L1 | CCL14 | -22.61750864 | -37.73888339 | 3 |
| 1869 | SAA1 | ANTXR2 | -22.61669342 | -37.71356558 | 3 |
| 1870 | CALD1 | ASCL2 | -22.61669342 | -32.95758362 | 3 |
| 1871 | CALD1 | PPEF2 | -22.61669342 | -32.95758362 | 3 |
| 1872 | CALD1 | PPEF1 | -22.61669342 | -32.95758362 | 3 |
| 1873 | REM1 | UCP2 | -22.61669342 | -32.6448995 | 3 |
| 1874 | REM1 | UCP3 | -22.61669342 | -32.6448995 | 3 |
| 1875 | KCNQ5 | PPEF1 | -22.61669342 | -32.95758362 | 3 |
| 1876 | GRM5 | PPEF1 | -22.61669342 | -32.95758362 | 3 |
| 1877 | DEF6 | ICMT | -22.61669342 | -32.8987907 | 3 |
| 1878 | CREG1 | RBBP9 | -22.61669342 | -32.33585507 | 3 |
| 1879 | MASP1 | MASP2 | -22.61669342 | -43.95449099 | 3 |
| 1880 | RPS6KA4 | PTPN7 | -22.61669342 | -30.5592851 | 3 |
| 1881 | RPS6KA4 | DUSP4 | -22.61669342 | -30.5592851 | 3 |
| 1882 | RPS6KA4 | GMFB | -22.61669342 | -30.5592851 | 3 |
| 1883 | TAC1 | TAC4 | -22.61669342 | -47.35568837 | 3 |
| 1884 | TRIP | HIVEP3 | -22.61669342 | -32.61579392 | 3 |
| 1885 | ARHGDIA | ICMT | -22.61669342 | -32.8987907 | 3 |
| 1886 | NRBF2 | TRIP3 | -22.61669342 | -33.65006212 | 3 |
| 1887 | CGI-63 | TRIP3 | -22.61669342 | -33.65006212 | 3 |
| 1888 | KCNA4 | ATP2B2 | -22.61669342 | -31.63938458 | 3 |
| 1889 | DUSP1 | DUSP4 | -22.61669342 | -30.5592851 | 3 |
| 1890 | DUSP1 | GMFB | -22.61669342 | -30.5592851 | 3 |
| 1891 | KSR2 | RPS6KA2 | -22.61669342 | -32.1881411 | 3 |
| 1892 | SEMA4C | GDA | -22.61669342 | -31.41037591 | 3 |
| 1893 | GOSR1 | GOSR2 | -22.61669342 | -39.03532142 | 3 |
| 1894 | RAB11B | RAB25 | -22.61669342 | -44.42894897 | 3 |
| 1895 | SF3B2 | SF3B14 | -22.61669342 | -42.00858084 | 3 |
| 1896 | NXT1 | NXT2 | -22.61669342 | -43.02715042 | 3 |
| 1897 | ATP2B4 | ATP2B2 | -22.61669342 | -31.63938458 | 3 |
| 1898 | SYK | BCAR1 | -22.60530488 | -66.51864682 | 7 |
| 1899 | RET | PDGFRB | -22.59927204 | -55.79077363 | 6 |
| 1900 | SOCS1 | STAT5A | -22.58914829 | -64.75471405 | 6 |
| 1901 | LCK | CD5 | -22.5727799 | -62.5895114 | 6 |
| 1902 | STATIP1 | GHR | -22.56697901 | -42.983935 | 4 |
| 1903 | RXRG | PPARBP | -22.56697901 | -47.63373804 | 4 |
| 1904 | KNG1 | F12 | -22.55936256 | -49.47759832 | 4 |
| 1905 | PTPRC | PLCG1 | -22.53270982 | -85.18945219 | 8 |
| 1906 | PXN | TGFB1I1 | -22.5245644 | -69.402544 | 6 |
| 1907 | GGA1 | AP1G1 | -22.51242414 | -53.7258837 | 4 |
| 1908 | GRIN2A | CRIPT | -22.50752885 | -43.61261183 | 4 |
| 1909 | GAB1 | SOS2 | -22.50752885 | -37.36167969 | 4 |
| 1910 | KCNJ12 | CRIPT | -22.50752885 | -43.61261183 | 4 |
| 1911 | HDAC9 | HDAC7A | -22.50752885 | -43.51801726 | 4 |
| 1912 | DDX20 | LSM11 | -22.50752885 | -51.45257681 | 4 |
| 1913 | ABL1 | RASA1 | -22.50280559 | -90.29100496 | 8 |
| 1914 | FOS | SRF | -22.49680336 | -83.47231966 | 7 |
| 1915 | MET | RET | -22.49308967 | -47.8771302 | 5 |
| 1916 | SYNCRIP | NRXN1 | -22.49308967 | -73.09852983 | 5 |
| 1917 | PLD1 | PLD2 | -22.49308967 | -60.50329623 | 5 |
| 1918 | RIBC2 | ZNF250 | -22.48900692 | -56.0624917 | 5 |
| 1919 | PIK3R1 | CSK | -22.43539648 | -79.6493073 | 8 |
| 1920 | HRAS | MRAS | -22.43353859 | -61.30133884 | 5 |
| 1921 | CD19 | PTK2B | -22.42285242 | -57.83209572 | 6 |
| 1922 | SOCS1 | PIK3R2 | -22.39985259 | -51.39859043 | 5 |
| 1923 | STAT3 | STAT5B | -22.3942186 | -74.02243155 | 6 |
| 1924 | SRC | EGFR | -22.39266553 | -135.1376422 | 13 |
| 1925 | GAB3 | CTLA4 | -22.39152442 | -36.55399383 | 4 |
| 1926 | APEX2 | ZNF638 | -22.39152442 | -41.73283998 | 4 |
| 1927 | APEX2 | KIAA1267 | -22.39152442 | -37.19880782 | 4 |
| 1928 | PER1 | CRY1 | -22.39152442 | -57.46751372 | 4 |
| 1929 | ZNF426 | ZNF638 | -22.39152442 | -38.64635334 | 4 |
| 1930 | ZNF426 | KIAA1267 | -22.39152442 | -41.53216928 | 4 |
| 1931 | ZNF426 | C16orf48 | -22.39152442 | -39.35535358 | 4 |
| 1932 | F8 | F5 | -22.38989287 | -52.07673112 | 4 |
| 1933 | ITSN1 | PACSIN1 | -22.38989287 | -50.05267179 | 4 |
| 1934 | SNRPD2 | SNRPD3 | -22.38989287 | -52.88254624 | 4 |
| 1935 | SKIL | Rasd2 | -22.38989287 | -36.73198694 | 4 |
| 1936 | INSR | PDGFRA | -22.38050408 | -72.64271909 | 6 |
| 1937 | GAB2 | PECAM1 | -22.37530709 | -46.23726014 | 5 |
| 1938 | GAB2 | LCP2 | -22.37530709 | -55.19018141 | 5 |
| 1939 | PAG1 | LCP2 | -22.37530709 | -48.9613128 | 5 |
| 1940 | CDKN1A | GADD45GIP1 | -22.34848963 | -49.97197177 | 4 |
| 1941 | SIT1 | PXN | -22.34407716 | -50.04863432 | 5 |
| 1942 | STATIP1 | IL2RB | -22.34397166 | -43.93491129 | 4 |
| 1943 | SAA1 | MMP9 | -22.34397166 | -50.17449604 | 4 |
| 1944 | RIBC2 | MGC10854 | -22.33581061 | -40.3510623 | 4 |
| 1945 | RAF1 | RALGDS | -22.33512776 | -75.91750618 | 6 |
| 1946 | MCM7 | CDC5L | -22.33502734 | -62.73204728 | 5 |
| 1947 | GAB2 | VAV1 | -22.31411349 | -60.63304259 | 6 |
| 1948 | PTPN11 | BCAR1 | -22.31185332 | -76.90006268 | 8 |
| 1949 | CD22 | KIT | -22.31105115 | -45.4116751 | 5 |
| 1950 | NR2F1 | POU2F1 | -22.31105115 | -52.05591566 | 5 |
| 1951 | CD2AP | ADAM12 | -22.30862635 | -37.53757036 | 4 |
| 1952 | PHB | HBP1 | -22.30862635 | -40.50617315 | 4 |
| 1953 | TRIP4 | STAT6 | -22.30862635 | -38.19779441 | 4 |
| 1954 | CASP10 | CARD4 | -22.30862635 | -50.44011048 | 4 |
| 1955 | GGA2 | GGA3 | -22.30862635 | -56.95668809 | 4 |
| 1956 | TNFAIP3 | TRPC4AP | -22.30862635 | -42.72661976 | 4 |
| 1957 | RBBP7 | HDAC1 | -22.30651189 | -57.07161006 | 5 |
| 1958 | GRB10 | SOCS3 | -22.30436247 | -52.11259258 | 5 |
| 1959 | KIAA0408 | 76P | -22.30436247 | -52.64427 | 5 |
| 1960 | KIT | CTLA4 | -22.30016086 | -47.52217186 | 5 |
| 1961 | SMN1 | SNRPD2 | -22.30016086 | -66.26488253 | 5 |
| 1962 | LSM8 | LSM6 | -22.30016086 | -63.20931025 | 5 |
| 1963 | CD19 | CBL | -22.29611359 | -61.3592729 | 6 |
| 1964 | VAV1 | PAG1 | -22.28875558 | -59.54030113 | 6 |
| 1965 | TOB1 | GLI3 | -22.2554511 | -36.88999119 | 4 |
| 1966 | EFNB2 | EFNB1 | -22.2554511 | -50.30192365 | 4 |
| 1967 | CTSB | CTSL | -22.25490725 | -62.23698695 | 4 |
| 1968 | RIMS2 | RPH3AL | -22.25490725 | -55.92479853 | 4 |
| 1969 | PCBD1 | TIFA | -22.25490725 | -42.87710155 | 4 |
| 1970 | ISL1 | RNF12 | -22.25490725 | -57.43766075 | 4 |
| 1971 | TANK | TRAF3IP2 | -22.25490725 | -42.72661976 | 4 |
| 1972 | SHC1 | SOCS3 | -22.24301527 | -74.15186706 | 7 |
| 1973 | INSR | IRS1 | -22.2372069 | -80.18014487 | 7 |
| 1974 | TRIM37 | NDP52 | -22.23706902 | -83.71050765 | 7 |
| 1975 | GTF2B | TAF1 | -22.2331726 | -65.92155405 | 6 |
| 1976 | PIK3R1 | GAB2 | -22.2282734 | -69.47949842 | 7 |
| 1977 | PIK3R1 | BCR | -22.2282734 | -69.19874331 | 7 |
| 1978 | CDKN3 | CCNE1 | -22.22501433 | -47.68780935 | 4 |
| 1979 | SFRS10 | KHDRBS3 | -22.22501433 | -56.93037078 | 4 |
| 1980 | MAP3K1 | MAP3K2 | -22.21957474 | -46.12416142 | 4 |
| 1981 | MUC1 | NRG1 | -22.21957474 | -43.96683069 | 4 |
| 1982 | BAD | BCL2L11 | -22.21957474 | -49.91890505 | 4 |
| 1983 | GADD45G | THRAP4 | -22.21957474 | -42.16927198 | 4 |
| 1984 | ERBB4 | CRIPT | -22.21957474 | -43.61261183 | 4 |
| 1985 | RIPK1 | CFLAR | -22.217619 | -56.08107742 | 5 |
| 1986 | PTPN11 | GHR | -22.21631565 | -66.94612691 | 7 |
| 1987 | PTPN11 | PTPN1 | -22.21631565 | -68.88911964 | 7 |
| 1988 | APP | SERPINE2 | -22.20809785 | -63.8657166 | 5 |
| 1989 | PTK2B | SCAP1 | -22.20809785 | -44.73997236 | 5 |
| 1990 | ERBB2 | EGF | -22.2007804 | -55.78006099 | 5 |
| 1991 | GHR | CRKL | -22.19511807 | -57.89387423 | 6 |
| 1992 | JAK2 | IL6ST | -22.1893427 | -77.65357806 | 7 |
| 1993 | PCBD1 | RABAC1 | -22.17343356 | -54.39070497 | 5 |
| 1994 | GRB2 | GAB1 | -22.17036157 | -82.1010307 | 8 |
| 1995 | LCK | SHC1 | -22.16800681 | -114.1138912 | 10 |
| 1996 | JUN | CEBPG | -22.16715808 | -88.84844938 | 7 |
| 1997 | CD2AP | SH3KBP1 | -22.16194518 | -54.02886692 | 5 |
| 1998 | SRC | VAV1 | -22.16127125 | -98.44006791 | 10 |
| 1999 | SOS1 | EGFR | -22.16024663 | -84.40391637 | 8 |
| 2000 | JUP | PKP2 | -22.15913714 | -72.35678047 | 5 |
| 2001 | PIK3R1 | PXN | -22.14886747 | -103.7339504 | 10 |
| 2002 | APH1A | PSENEN | -22.14723332 | -37.02446699 | 3 |
| 2003 | SOSTDC1 | NOG | -22.14723332 | -40.01615067 | 3 |
| 2004 | MYF6 | EMP3 | -22.14723332 | -32.95758362 | 3 |
| 2005 | MYF6 | KCNQ2 | -22.14723332 | -32.95758362 | 3 |
| 2006 | GRIN2C | KCNJ10 | -22.14723332 | -34.2235727 | 3 |
| 2007 | RGS18 | RGS5 | -22.14723332 | -33.50251101 | 3 |
| 2008 | GALNT10 | GALNT14 | -22.14723332 | -47.35568837 | 3 |
| 2009 | TAL2 | LYL1 | -22.14723332 | -37.74087103 | 3 |
| 2010 | PTPRS | PTPRD | -22.14723332 | -39.52367419 | 3 |
| 2011 | PTPRS | PPFIBP1 | -22.14723332 | -39.52367419 | 3 |
| 2012 | PTPRD | PPFIBP1 | -22.14723332 | -39.52367419 | 3 |
| 2013 | TNFRSF18 | TNFRSF12A | -22.14723332 | -30.56167018 | 3 |
| 2014 | VPS16 | VPS18 | -22.14723332 | -43.44366536 | 3 |
| 2015 | RAB11FIP5 | MYO5B | -22.14723332 | -43.93614185 | 3 |
| 2016 | DCTD | PAICS | -22.14723332 | -35.48807427 | 3 |
| 2017 | TEAD4 | TEAD3 | -22.14723332 | -43.80034031 | 3 |
| 2018 | GRB2 | STAT5A | -22.13454238 | -103.3310099 | 10 |
| 2019 | TUB | CBL | -22.13163299 | -45.87973468 | 5 |
| 2020 | AXL | CBL | -22.13163299 | -48.05433368 | 5 |
| 2021 | MST1R | CBL | -22.13163299 | -43.70632075 | 5 |
| 2022 | SCAP1 | CBL | -22.13163299 | -44.73997236 | 5 |
| 2023 | TRPV4 | CBL | -22.13163299 | -48.61953751 | 5 |
| 2024 | RIPK1 | TNFRSF19 | -22.12409307 | -43.16570148 | 4 |
| 2025 | ATF4 | TEF | -22.12409307 | -51.20678568 | 4 |
| 2026 | E2F4 | E2F1 | -22.11515918 | -60.23164539 | 5 |
| 2027 | RCC1 | KPNB1 | -22.11515918 | -62.84831565 | 5 |
| 2028 | NR3C1 | AHR | -22.11164254 | -76.38421011 | 7 |
| 2029 | GRB2 | PXN | -22.1092938 | -129.4958576 | 12 |
| 2030 | PTPN11 | GRB7 | -22.09712174 | -60.81404329 | 6 |
| 2031 | PRKCA | PRKCD | -22.09510292 | -90.63735478 | 7 |
| 2032 | BCL6 | HDAC4 | -22.08898779 | -58.10320802 | 5 |
| 2033 | IL2RB | IL4R | -22.08558527 | -53.85066538 | 5 |
| 2034 | CCNA2 | CCNA1 | -22.08558527 | -57.43475486 | 5 |
| 2035 | CHML | RABAC1 | -22.0808476 | -52.66670199 | 4 |
| 2036 | CD3Z | CBL | -22.07786078 | -83.08970787 | 7 |
| 2037 | JUP | PKP3 | -22.06614771 | -59.76004847 | 4 |
| 2038 | RET | ERBB2 | -22.02212924 | -56.81387837 | 6 |
| 2039 | PML | BCL6 | -22.01947102 | -61.47644567 | 6 |
| 2040 | GADD45G | PPARBP | -22.00184068 | -52.95444188 | 5 |
| 2041 | PTPN1 | SOCS3 | -21.99979917 | -50.92453523 | 5 |
| 2042 | CDKN1A | MCM10 | -21.99974057 | -75.6332589 | 6 |
| 2043 | ERCC2 | CCNH | -21.9866036 | -46.15177768 | 4 |
| 2044 | BAK1 | BAX | -21.9866036 | -47.68531283 | 4 |
| 2045 | VAMP8 | NAPA | -21.9866036 | -47.58778365 | 4 |
| 2046 | CCL5 | CCL3 | -21.98442806 | -51.84847247 | 4 |
| 2047 | ORC1L | MCM5 | -21.98442806 | -49.46064627 | 4 |
| 2048 | SNRPE | SNRPD1 | -21.98442806 | -52.88254624 | 4 |
| 2049 | NAPA | NSF | -21.98442806 | -51.29304603 | 4 |
| 2050 | BAHD1 | ZNF408 | -21.97582573 | -42.43952622 | 4 |
| 2051 | BMPR1B | SMAD4 | -21.97299347 | -161.5932163 | 11 |
| 2052 | APOB | TG | -21.97201827 | -46.22528575 | 4 |
| 2053 | CD36 | TRPV4 | -21.97201827 | -39.44046662 | 4 |
| 2054 | SFRS2 | U2AF2 | -21.96578016 | -71.38187784 | 5 |
| 2055 | IKBKB | MAP3K14 | -21.96033576 | -56.65162228 | 5 |
| 2056 | DCN | SPARC | -21.96033576 | -56.96958438 | 5 |
| 2057 | IRS2 | SHB | -21.95850752 | -48.4644745 | 5 |
| 2058 | LAT | SHB | -21.95850752 | -50.79677473 | 5 |
| 2059 | CEBPG | HLF | -21.95103868 | -51.17779815 | 4 |
| 2060 | ORC2L | ORC5L | -21.95103868 | -51.2160381 | 4 |
| 2061 | CSPG2 | AGC1 | -21.95103868 | -57.31987772 | 4 |
| 2062 | HDAC5 | HDAC7A | -21.95103868 | -43.81273101 | 4 |
| 2063 | TP53 | MDM2 | -21.94421745 | -103.0400445 | 9 |
| 2064 | SAA1 | DCN | -21.93226431 | -50.17449604 | 4 |
| 2065 | MYOD1 | MYF6 | -21.93207084 | -47.36442422 | 4 |
| 2066 | ANTXR2 | HABP2 | -21.92436145 | -37.71356558 | 3 |
| 2067 | MS4A2 | FCER1G | -21.92436145 | -37.24054522 | 3 |
| 2068 | JAG2 | MAML1 | -21.92436145 | -38.70966231 | 3 |
| 2069 | ALS2CR2 | MAP3K7IP2 | -21.92436145 | -34.21806863 | 3 |
| 2070 | AP1S2 | AP1GBP1 | -21.92436145 | -40.93813943 | 3 |
| 2071 | IL8 | CXCL1 | -21.92313855 | -41.96206082 | 3 |
| 2072 | CCL8 | CCL3L1 | -21.92313855 | -37.73888339 | 3 |
| 2073 | TIMP3 | SPOCK | -21.92313855 | -40.42813046 | 3 |
| 2074 | BNIP3 | BNIP1 | -21.92313855 | -34.44162304 | 3 |
| 2075 | MC4R | MC5R | -21.92313855 | -44.54227765 | 3 |
| 2076 | EFNA1 | EFNA3 | -21.92313855 | -41.86399961 | 3 |
| 2077 | CRIPT | GDA | -21.92313855 | -31.41037591 | 3 |
| 2078 | CRIPT | ATP2B2 | -21.92313855 | -31.63938458 | 3 |
| 2079 | DDIT3 | TEF | -21.92301386 | -53.60468096 | 4 |
| 2080 | GTF2B | TRIP4 | -21.92193443 | -51.49509365 | 5 |
| 2081 | GTF2B | GTF2F1 | -21.92193443 | -62.44637439 | 5 |
| 2082 | SRC | GAB1 | -21.91751166 | -71.30429109 | 7 |
| 2083 | EGFR | BCR | -21.91751166 | -69.01601599 | 7 |
| 2084 | TP53 | RB1 | -21.91662909 | -169.9583386 | 14 |
| 2085 | TRAF2 | NDP52 | -21.91539974 | -138.9625247 | 11 |
| 2086 | SIN3A | ZNFN1A4 | -21.90579444 | -61.19928433 | 5 |
| 2087 | PTK2B | BCR | -21.8892621 | -55.66737145 | 6 |
| 2088 | PML | MYC | -21.84558001 | -84.64134376 | 8 |
| 2089 | IRAK1 | TRAF6 | -21.84303176 | -81.60743195 | 6 |
| 2090 | VIM | DIPA | -21.83038841 | -126.7718346 | 10 |
| 2091 | VAV1 | SOCS3 | -21.82421122 | -65.04298408 | 6 |
| 2092 | RBBP4 | CTBP1 | -21.81841703 | -48.09245701 | 5 |
| 2093 | JAK1 | STAT1 | -21.81744136 | -79.93626569 | 7 |
| 2094 | CTBP1 | ZNFN1A1 | -21.81433373 | -55.49763973 | 5 |
| 2095 | GRB14 | SNX2 | -21.80414629 | -42.31723521 | 4 |
| 2096 | SMARCD1 | ABLIM1 | -21.80414629 | -42.39034652 | 4 |
| 2097 | SMARCD1 | KIAA1267 | -21.80414629 | -39.07543351 | 4 |
| 2098 | PER3 | CRY1 | -21.80414629 | -55.01077794 | 4 |
| 2099 | C1orf65 | C16orf48 | -21.80414629 | -38.97887601 | 4 |
| 2100 | CD22 | CSF3R | -21.80305853 | -36.78597117 | 4 |
| 2101 | GRB7 | EGF | -21.80305853 | -39.34136781 | 4 |
| 2102 | NR0B2 | ERBP | -21.80305853 | -42.34725513 | 4 |
| 2103 | ABLIM1 | MGC3162 | -21.80305853 | -42.77326477 | 4 |
| 2104 | ZNF638 | C1orf65 | -21.80305853 | -37.47055141 | 4 |
| 2105 | PTPN11 | IL2RB | -21.794012 | -67.09574243 | 7 |
| 2106 | BCR | CBL | -21.79047777 | -59.85001756 | 6 |
| 2107 | IRS2 | SHC1 | -21.78703514 | -71.22190971 | 7 |
| 2108 | LSM5 | LSM8 | -21.77437197 | -63.39163181 | 5 |
| 2109 | BCL2L1 | MCL1 | -21.76457971 | -68.25446774 | 5 |
| 2110 | MGC2749 | LOC138046 | -21.76416164 | -56.08215945 | 5 |
| 2111 | GRB2 | IGF1R | -21.74932729 | -107.6836462 | 10 |
| 2112 | GRK6 | GNA11 | -21.74489796 | -55.82455311 | 4 |
| 2113 | SIP1 | SNRPB | -21.74489796 | -50.35396452 | 4 |
| 2114 | ORC3L | MCM2 | -21.74134532 | -51.43130759 | 4 |
| 2115 | PARP1 | PARP2 | -21.73155007 | -59.77009881 | 4 |
| 2116 | FADD | DEDD2 | -21.73155007 | -49.93006947 | 4 |
| 2117 | MCM2 | CCDC5 | -21.73155007 | -48.94936099 | 4 |
| 2118 | CASP3 | CASP9 | -21.72414064 | -82.40202574 | 6 |
| 2119 | KHDRBS1 | CD28 | -21.71575925 | -51.17981331 | 5 |
| 2120 | FADD | CFLAR | -21.71575925 | -63.50066205 | 5 |
| 2121 | TRADD | MAP3K14 | -21.71575925 | -53.11462336 | 5 |
| 2122 | SNAP23 | VAMP2 | -21.71575925 | -66.90385944 | 5 |
| 2123 | ITGAV | ITGA5 | -21.71293556 | -70.34395962 | 5 |
| 2124 | SRC | PXN | -21.7118011 | -117.441514 | 10 |
| 2125 | NR2F6 | NCOA2 | -21.70598054 | -40.03366621 | 4 |
| 2126 | GTF2H1 | ERCC6 | -21.69945215 | -45.73163316 | 4 |
| 2127 | GIT1 | PAK3 | -21.69945215 | -54.44664547 | 4 |
| 2128 | CASP9 | DIABLO | -21.69945215 | -52.85437536 | 4 |
| 2129 | EFCBP2 | RPIA | -21.69945215 | -45.19455479 | 4 |
| 2130 | PTMA | NUP98 | -21.69920054 | -54.34482601 | 5 |
| 2131 | HSPCA | HSPA1A | -21.69553565 | -97.35621998 | 8 |
| 2132 | SMN1 | LSM8 | -21.68990872 | -76.31686787 | 6 |
| 2133 | GNB2L1 | IRS2 | -21.67584644 | -66.82510985 | 6 |
| 2134 | PLCG1 | LCK | -21.66833164 | -104.7837445 | 9 |
| 2135 | RET | INSR | -21.66670237 | -72.32291046 | 6 |
| 2136 | RAB8A | RAB3A | -21.66607916 | -55.43459219 | 4 |
| 2137 | NCOR2 | RELA | -21.66478315 | -83.71945298 | 8 |
| 2138 | PIK3R1 | YES1 | -21.66440836 | -86.36480426 | 7 |
| 2139 | PIK3R1 | SOCS3 | -21.66440836 | -78.20823256 | 7 |
| 2140 | GRIN2A | KCNJ4 | -21.66172749 | -43.72383747 | 4 |
| 2141 | PAG1 | SCAP1 | -21.66172749 | -36.02910605 | 4 |
| 2142 | RBBP4 | HBP1 | -21.66172749 | -41.45818197 | 4 |
| 2143 | UBE2L3 | UBE2E1 | -21.66172749 | -52.57239131 | 4 |
| 2144 | UBE2L3 | UBE2D3 | -21.66172749 | -46.70027352 | 4 |
| 2145 | ZNFN1A1 | SETDB1 | -21.66172749 | -42.68926801 | 4 |
| 2146 | TU3A | ZNF417 | -21.66172749 | -40.45907906 | 4 |
| 2147 | GTF2F2 | GTF2E2 | -21.65340009 | -61.03889693 | 5 |
| 2148 | RASA1 | IRS1 | -21.65162436 | -75.17085113 | 7 |
| 2149 | KHDRBS1 | PDGFRB | -21.65025834 | -55.44748021 | 6 |
| 2150 | PDGFRB | WAS | -21.64289938 | -53.78925213 | 6 |
| 2151 | PLG | F2 | -21.64289938 | -79.72044718 | 6 |
| 2152 | HRAS | RRAS | -21.62724243 | -59.73824126 | 5 |
| 2153 | DRD3 | DRD2 | -21.61942319 | -53.16117847 | 4 |
| 2154 | RASSF5 | RALGDS | -21.61942319 | -53.30636048 | 4 |
| 2155 | ELA2 | PRTN3 | -21.61670358 | -57.91211873 | 4 |
| 2156 | BGN | TGFBI | -21.61670358 | -47.61388508 | 4 |
| 2157 | CD2AP | GAB3 | -21.61670358 | -33.94231501 | 4 |
| 2158 | CD2AP | NPHS1 | -21.61670358 | -45.09354042 | 4 |
| 2159 | TRIP4 | NPAS2 | -21.61670358 | -38.74992805 | 4 |
| 2160 | FBF1 | LZTS2 | -21.61670358 | -39.65970102 | 4 |
| 2161 | CASP10 | DEDD | -21.61670358 | -49.78300823 | 4 |
| 2162 | RTN4 | RTN3 | -21.61670358 | -52.11587103 | 4 |
| 2163 | TRAF2 | CHUK | -21.60999089 | -115.2045533 | 9 |
| 2164 | JAK1 | CRKL | -21.60545815 | -69.39528522 | 7 |
| 2165 | VAV1 | SH3BP2 | -21.56419771 | -50.87039125 | 5 |
| 2166 | JAK2 | CSF2RB | -21.55377258 | -63.34835078 | 6 |
| 2167 | RXRA | VDR | -21.55377258 | -69.39937253 | 6 |
| 2168 | MCM10 | UTP14A | -21.54881989 | -43.73031936 | 4 |
| 2169 | SIT1 | PTK2 | -21.54390367 | -47.82560186 | 5 |
| 2170 | CSK | GRB2 | -21.51338234 | -99.52127143 | 9 |
| 2171 | GNAI2 | GNAZ | -21.51172154 | -67.78730906 | 5 |
| 2172 | TNFRSF1A | TNFRSF14 | -21.51172154 | -61.2797788 | 5 |
| 2173 | PTK2 | TUB | -21.51049521 | -46.51109437 | 5 |
| 2174 | PTK2 | MST1R | -21.51049521 | -43.70632075 | 5 |
| 2175 | SYK | PECAM1 | -21.50669428 | -56.28034877 | 6 |
| 2176 | TP53 | SUMO1 | -21.50617788 | -121.2645992 | 10 |
| 2177 | BRCA1 | MYOD1 | -21.50588252 | -76.31681366 | 8 |
| 2178 | LRP2 | LDLR | -21.49357486 | -67.01597274 | 5 |
| 2179 | AKT1 | AKT2 | -21.49357486 | -74.52071095 | 5 |
| 2180 | MCM10 | ZNF250 | -21.48269041 | -55.79902388 | 5 |
| 2181 | HDAC1 | RBBP8 | -21.46944187 | -81.87771018 | 7 |
| 2182 | PECAM1 | TYRO3 | -21.46279122 | -38.56777789 | 4 |
| 2183 | ABL1 | FYN | -21.45117286 | -119.5199905 | 10 |
| 2184 | CREBBP | SP1 | -21.43987663 | -114.4199556 | 10 |
| 2185 | IRS2 | VAV1 | -21.41807832 | -59.87800682 | 6 |
| 2186 | SMAD2 | SKIL | -21.41404326 | -67.36332396 | 6 |
| 2187 | PTPN11 | VAV3 | -21.41258176 | -55.82169778 | 6 |
| 2188 | SERPINE1 | THBS1 | -21.40108036 | -58.61820403 | 5 |
| 2189 | RAPGEF1 | MAP4K1 | -21.3993616 | -37.88902165 | 4 |
| 2190 | BIRC5 | BIRC2 | -21.3993616 | -49.65506193 | 4 |
| 2191 | ACVR2A | INHBB | -21.39772983 | -54.49709925 | 4 |
| 2192 | VAV3 | VAV2 | -21.39772983 | -40.03086373 | 4 |
| 2193 | HLA-A | HLA-G | -21.39772983 | -53.98767321 | 4 |
| 2194 | EGFR | CRKL | -21.38985751 | -96.73493281 | 9 |
| 2195 | MAPK10 | MAPK9 | -21.3788055 | -49.64177056 | 4 |
| 2196 | CDKN1A | CDK6 | -21.37875609 | -57.43759187 | 5 |
| 2197 | DAB2 | STRAP | -21.37390953 | -37.55189878 | 4 |
| 2198 | CD19 | PTPN6 | -21.369916 | -62.20750963 | 6 |
| 2199 | IL2RB | IRS2 | -21.36667288 | -47.88508621 | 5 |
| 2200 | SLC4A3 | SLC4A1 | -21.36474567 | -44.17763454 | 3 |
| 2201 | ASCL2 | MYF5 | -21.36474567 | -32.95758362 | 3 |
| 2202 | ASCL2 | KCNQ3 | -21.36474567 | -32.95758362 | 3 |
| 2203 | ASCL2 | RALB | -21.36474567 | -32.95758362 | 3 |
| 2204 | PPEF2 | MYF5 | -21.36474567 | -32.95758362 | 3 |
| 2205 | PPEF2 | KCNQ3 | -21.36474567 | -32.95758362 | 3 |
| 2206 | PPEF2 | RALB | -21.36474567 | -32.95758362 | 3 |
| 2207 | PPEF1 | KCNQ3 | -21.36474567 | -32.95758362 | 3 |
| 2208 | PPEF1 | RALB | -21.36474567 | -32.95758362 | 3 |
| 2209 | ANTXR2 | SERPINE2 | -21.36474567 | -37.71356558 | 3 |
| 2210 | SLA2 | SLA | -21.36474567 | -32.30811371 | 3 |
| 2211 | SLITL2 | CTGF | -21.36474567 | -37.19786872 | 3 |
| 2212 | FMOD | CTGF | -21.36474567 | -37.19786872 | 3 |
| 2213 | CALCRL | ADMR | -21.36311501 | -46.15171557 | 3 |
| 2214 | RAB3B | ASCL2 | -21.36311501 | -32.95758362 | 3 |
| 2215 | RAB3B | PPEF2 | -21.36311501 | -32.95758362 | 3 |
| 2216 | RAB3B | PPEF1 | -21.36311501 | -32.95758362 | 3 |
| 2217 | MYF5 | PPEF1 | -21.36311501 | -32.95758362 | 3 |
| 2218 | AKAP8 | DMTF1 | -21.36311501 | -36.58867815 | 3 |
| 2219 | ARHGDIB | ICMT | -21.36311501 | -32.8987907 | 3 |
| 2220 | BIRC3 | BIRC7 | -21.36311501 | -35.24822133 | 3 |
| 2221 | CRHR1 | CRHR2 | -21.36311501 | -50.24606013 | 3 |
| 2222 | CCL7 | CCL28 | -21.36311501 | -40.03141815 | 3 |
| 2223 | CCL7 | CCL3L1 | -21.36311501 | -37.73888339 | 3 |
| 2224 | MAP2K1IP1 | RPS6KA2 | -21.36311501 | -32.1881411 | 3 |
| 2225 | STRAP | RNF111 | -21.36311501 | -34.0890515 | 3 |
| 2226 | TXNL2 | EGFL9 | -21.36311501 | -31.73230771 | 3 |
| 2227 | KCNJ4 | ATP2B2 | -21.36311501 | -31.63938458 | 3 |
| 2228 | CLDN1 | CLDN5 | -21.36311501 | -40.90600815 | 3 |
| 2229 | BTG2 | BTG1 | -21.36311501 | -39.46960697 | 3 |
| 2230 | TNFRSF1A | IKBKG | -21.36285428 | -70.79667209 | 6 |
| 2231 | PTK2B | STAT5A | -21.35730037 | -69.43450704 | 7 |
| 2232 | EGFR | ERBB4 | -21.35419751 | -94.99216793 | 7 |
| 2233 | GRB2 | SOS1 | -21.34765 | -97.72701767 | 9 |
| 2234 | PML | BRCA1 | -21.33928222 | -83.3181877 | 9 |
| 2235 | CBLB | LCP2 | -21.33056617 | -52.09811574 | 5 |
| 2236 | RICS | PECAM1 | -21.33056617 | -44.8097099 | 5 |
| 2237 | PTPN1 | GRB10 | -21.32988557 | -50.59496222 | 5 |
| 2238 | HSPCA | TRPC4AP | -21.32771581 | -57.09316345 | 5 |
| 2239 | JAK1 | PIK3R1 | -21.32365663 | -98.24193144 | 9 |
| 2240 | STX1A | STX1B1 | -21.32141321 | -54.86392657 | 4 |
| 2241 | BLNK | WAS | -21.31819193 | -53.25753396 | 5 |
| 2242 | GAB1 | SOS1 | -21.31819193 | -47.17927626 | 5 |
| 2243 | KHDRBS1 | BLNK | -21.31274679 | -54.79059889 | 5 |
| 2244 | PML | HDAC3 | -21.30206467 | -82.00260116 | 8 |
| 2245 | NCOA1 | TCF1 | -21.29784327 | -62.57769287 | 6 |
| 2246 | GRK6 | GRK5 | -21.29372927 | -55.37396757 | 4 |
| 2247 | HOXA1 | ILF3 | -21.29372927 | -38.47372551 | 4 |
| 2248 | SKB1 | STAM2 | -21.29372927 | -44.17598101 | 4 |
| 2249 | SERPINE1 | SERPING1 | -21.29318535 | -47.57539724 | 4 |
| 2250 | TOP2A | CHD3 | -21.29318535 | -37.86752234 | 4 |
| 2251 | CD40 | TRIM37 | -21.2911818 | -53.47486974 | 5 |
| 2252 | TRIP4 | POU2F1 | -21.2911818 | -48.69287456 | 5 |
| 2253 | SRC | ABL1 | -21.28377971 | -124.0237217 | 10 |
| 2254 | EGFR | CBL | -21.28377971 | -103.0536195 | 10 |
| 2255 | ERCC2 | ERCC3 | -21.28349539 | -49.5640249 | 4 |
| 2256 | ZNF426 | ZNF408 | -21.28349539 | -40.50007068 | 4 |
| 2257 | LSM8 | LSM2 | -21.28165151 | -63.55868589 | 5 |
| 2258 | WASL | SYNJ1 | -21.28023164 | -49.4044858 | 4 |
| 2259 | WASL | WASF1 | -21.28023164 | -43.68610137 | 4 |
| 2260 | HLA-C | HLA-B | -21.28023164 | -53.98767321 | 4 |
| 2261 | CD36 | NPHS1 | -21.28023164 | -39.44046662 | 4 |
| 2262 | ITK | GRAP2 | -21.26758053 | -59.61510037 | 5 |
| 2263 | PRKAR1A | PRKAR2A | -21.26758053 | -77.16262135 | 5 |
| 2264 | ITGB2 | ITGAM | -21.26009295 | -71.59682462 | 5 |
| 2265 | BCOR | ZBTB16 | -21.25897876 | -59.20150197 | 5 |
| 2266 | NDP52 | ZBTB8 | -21.25846254 | -69.41344299 | 6 |
| 2267 | FAS | TNFRSF10B | -21.23937549 | -45.71595581 | 4 |
| 2268 | HDAC4 | HDAC7A | -21.23937549 | -43.81273101 | 4 |
| 2269 | CALM1 | ID2 | -21.23326888 | -62.53356299 | 5 |
| 2270 | SCYE1 | EPRS | -21.23107851 | -41.55659572 | 3 |
| 2271 | CDH7 | CDH9 | -21.23107851 | -37.91423628 | 3 |
| 2272 | MYF6 | KCNQ5 | -21.23107851 | -32.95758362 | 3 |
| 2273 | MYF6 | GRM5 | -21.23107851 | -32.95758362 | 3 |
| 2274 | DLL1 | JAG1 | -21.23107851 | -38.70966231 | 3 |
| 2275 | RAPGEF5 | RAPGEF6 | -21.23107851 | -39.64145722 | 3 |
| 2276 | U2AF1L2 | SFRS11 | -21.23107851 | -36.24973164 | 3 |
| 2277 | PEX13 | PEX14 | -21.23107851 | -41.03472026 | 3 |
| 2278 | TNFRSF4 | TNFRSF19 | -21.23107851 | -31.98570487 | 3 |
| 2279 | KIF1B | SEMA4C | -21.23107851 | -31.10962176 | 3 |
| 2280 | CCL11 | CCL14 | -21.23107851 | -36.89158553 | 3 |
| 2281 | TNFRSF18 | TNFRSF9 | -21.23107851 | -30.56167018 | 3 |
| 2282 | TNFRSF18 | TNFRSF19 | -21.23107851 | -30.56167018 | 3 |
| 2283 | TNFRSF12A | TNFRSF9 | -21.23107851 | -30.56167018 | 3 |
| 2284 | TNFRSF12A | TNFRSF19 | -21.23107851 | -30.56167018 | 3 |
| 2285 | VPS16 | VPS11 | -21.23107851 | -43.95449099 | 3 |
| 2286 | EIF2B5 | EIF2B4 | -21.23107851 | -44.82995973 | 3 |
| 2287 | EPRS | DARS | -21.23067087 | -35.93800809 | 3 |
| 2288 | CALD1 | MYF6 | -21.23067087 | -32.95758362 | 3 |
| 2289 | CALD1 | EMP3 | -21.23067087 | -32.95758362 | 3 |
| 2290 | CALD1 | KCNQ2 | -21.23067087 | -32.95758362 | 3 |
| 2291 | RAMP1 | RAMP3 | -21.23067087 | -45.40977822 | 3 |
| 2292 | GRM5 | EMP3 | -21.23067087 | -32.95758362 | 3 |
| 2293 | GRM5 | KCNQ2 | -21.23067087 | -32.95758362 | 3 |
| 2294 | CNR1 | RGS18 | -21.23067087 | -33.37936603 | 3 |
| 2295 | TAC1 | TAC3 | -21.23067087 | -47.35568837 | 3 |
| 2296 | GPSM2 | RGS5 | -21.23067087 | -34.36197941 | 3 |
| 2297 | ASIP | AGRP | -21.23067087 | -47.53800993 | 3 |
| 2298 | DNAJC7 | HUS1B | -21.23067087 | -40.7935582 | 3 |
| 2299 | NFKBIE | NKRF | -21.23067087 | -36.03698837 | 3 |
| 2300 | KCNA4 | KCNA5 | -21.23067087 | -32.96852053 | 3 |
| 2301 | EFNA5 | EFNA2 | -21.23067087 | -42.48815392 | 3 |
| 2302 | TNFRSF7 | TNFRSF4 | -21.23067087 | -31.98570487 | 3 |
| 2303 | VPS11 | VPS18 | -21.23067087 | -43.95449099 | 3 |
| 2304 | CCL14 | CCL13 | -21.23067087 | -36.89158553 | 3 |
| 2305 | Rras2 | Nkiras1 | -21.23067087 | -30.19228627 | 3 |
| 2306 | MAPK14 | MAPK8 | -21.22128521 | -97.46606685 | 7 |
| 2307 | STX1A | STX7 | -21.21700872 | -67.82268532 | 5 |
| 2308 | GRB2 | GRB7 | -21.20686542 | -82.20759292 | 7 |
| 2309 | PTPN11 | CBL | -21.19094805 | -90.54057449 | 9 |
| 2310 | IKBKB | RIPK2 | -21.18480999 | -53.91557269 | 5 |
| 2311 | FN1 | COL4A2 | -21.16269181 | -74.97596708 | 6 |
| 2312 | GAB3 | CBL | -21.15626123 | -43.61237254 | 5 |
| 2313 | PIK3R1 | IRS2 | -21.14528078 | -70.84115105 | 7 |
| 2314 | RARA | NR3C1 | -21.13369555 | -91.75230056 | 8 |
| 2315 | NCOA1 | NPAS2 | -21.1276352 | -50.33142937 | 5 |
| 2316 | NCOA1 | DAP3 | -21.1276352 | -53.69258236 | 5 |
| 2317 | HDAC3 | HDAC5 | -21.12561614 | -77.8128559 | 6 |
| 2318 | PELP1 | BAG1 | -21.11094998 | -36.42012283 | 4 |
| 2319 | TP53 | PRKDC | -21.10907673 | -107.3439105 | 9 |
| 2320 | GRIN2B | KCNJ4 | -21.10550965 | -47.1009373 | 4 |
| 2321 | IRS2 | MST1R | -21.10550965 | -34.99545444 | 4 |
| 2322 | BCL3 | TSC22D3 | -21.10550965 | -42.75316752 | 4 |
| 2323 | CEBPG | NFIL3 | -21.10550965 | -52.71824319 | 4 |
| 2324 | RB1 | TP73 | -21.09954267 | -63.93307483 | 6 |
| 2325 | DIPA | EFEMP2 | -21.09598897 | -120.1591088 | 9 |
| 2326 | CDH1 | CTNNA1 | -21.08535104 | -56.77267817 | 5 |
| 2327 | GAB1 | PTK2 | -21.08333559 | -54.66189786 | 6 |
| 2328 | PAG1 | PTK2 | -21.08333559 | -56.78398244 | 6 |
| 2329 | TNFRSF1A | TRADD | -21.06453219 | -70.68435236 | 6 |
| 2330 | PPARGC1A | NRIP1 | -21.05523384 | -51.49470822 | 5 |
| 2331 | AR | AHR | -21.05427805 | -72.85321257 | 7 |
| 2332 | ESR1 | NCOA3 | -21.05427805 | -76.86478195 | 7 |
| 2333 | ESR1 | AHR | -21.05427805 | -77.96514652 | 7 |
| 2334 | FXR2 | LMO3 | -21.05117451 | -69.2840391 | 6 |
| 2335 | JAK2 | GAB2 | -21.04977479 | -56.48243458 | 6 |
| 2336 | JAK2 | BCR | -21.04977479 | -57.97647122 | 6 |
| 2337 | IL2RB | SHB | -21.04329477 | -51.12894155 | 5 |
| 2338 | CCNA2 | CCNB1 | -21.04329477 | -59.14202401 | 5 |
| 2339 | CCNA2 | SKP2 | -21.04329477 | -58.54457895 | 5 |
| 2340 | MMP9 | MMP2 | -21.04329477 | -69.77739043 | 5 |
| 2341 | TGFBR3 | TGFB2 | -21.03231732 | -44.1682734 | 4 |
| 2342 | GEMIN5 | LSM2 | -21.03231732 | -49.74782872 | 4 |
| 2343 | INS | IGF2 | -21.03231732 | -48.49274609 | 4 |
| 2344 | JAK1 | FYN | -21.03027336 | -95.94088862 | 9 |
| 2345 | CRK | FYN | -21.03027336 | -101.1151268 | 9 |
| 2346 | PTPN12 | RAPGEF1 | -21.03014149 | -38.95396611 | 4 |
| 2347 | DNMT1 | DNMT3A | -21.03014149 | -47.28159193 | 4 |
| 2348 | CBX1 | CBX3 | -21.03014149 | -50.00656322 | 4 |
| 2349 | TRIP4 | TADA3L | -21.03014149 | -38.69960894 | 4 |
| 2350 | SMARCB1 | ACTL6A | -21.03014149 | -45.04478753 | 4 |
| 2351 | SMARCB1 | SMARCE1 | -21.03014149 | -41.28240291 | 4 |
| 2352 | ARGBP2 | SORBS1 | -21.03014149 | -45.46570321 | 4 |
| 2353 | RUNX1T1 | CBFA2T3 | -21.02779417 | -43.69380859 | 4 |
| 2354 | RUNX1T1 | HDAC7A | -21.02779417 | -41.96540169 | 4 |
| 2355 | RIPK1 | TNFRSF10B | -21.02779417 | -46.55220383 | 4 |
| 2356 | GRB2 | PDGFRB | -21.02567749 | -96.44165989 | 10 |
| 2357 | TRAF2 | KIAA1446 | -21.02202397 | -104.6369462 | 8 |
| 2358 | IRS2 | PTK2B | -21.02186588 | -57.23950999 | 6 |
| 2359 | KIAA1446 | DIPA | -21.01415873 | -104.1334198 | 8 |
| 2360 | SMAD2 | SMAD7 | -20.99908248 | -82.55305108 | 7 |
| 2361 | RASA1 | RASA3 | -20.99497547 | -47.70292048 | 4 |
| 2362 | SMURF2 | Wwp2 | -20.99497547 | -37.65198224 | 4 |
| 2363 | VAV1 | SHB | -20.99434883 | -61.18539043 | 6 |
| 2364 | VAV1 | GRB10 | -20.99434883 | -60.14165675 | 6 |
| 2365 | PTK2 | PLCG1 | -20.98429756 | -90.95574262 | 9 |
| 2366 | ORC4L | MCM6 | -20.97388498 | -46.44293509 | 4 |
| 2367 | ZNFN1A3 | ZNFN1A1 | -20.97388498 | -45.5336937 | 4 |
| 2368 | BLNK | SH3BP2 | -20.97007701 | -40.53250308 | 4 |
| 2369 | GAB1 | GAB3 | -20.97007701 | -37.26483709 | 4 |
| 2370 | GAB2 | SH3BP2 | -20.97007701 | -43.07926141 | 4 |
| 2371 | PAG1 | GAB3 | -20.97007701 | -35.45856119 | 4 |
| 2372 | PAG1 | SH3BP2 | -20.97007701 | -39.30312056 | 4 |
| 2373 | PML | NCOA1 | -20.96043175 | -86.52820828 | 8 |
| 2374 | PXN | CBL | -20.96043175 | -80.12345921 | 8 |
| 2375 | CCND1 | JUN | -20.95699814 | -77.7074468 | 8 |
| 2376 | KIT | PTK2 | -20.95675293 | -69.00073776 | 7 |
| 2377 | EPOR | PTK2 | -20.95675293 | -69.85536505 | 7 |
| 2378 | TRADD | RIPK2 | -20.94050632 | -53.74430097 | 5 |
| 2379 | HRAS | KRAS | -20.93872994 | -69.31248663 | 5 |
| 2380 | SIT1 | PLCG1 | -20.93600052 | -47.7874988 | 5 |
| 2381 | SCAP1 | PLCG1 | -20.93600052 | -44.73997236 | 5 |
| 2382 | BRCA1 | PCNA | -20.93004309 | -120.2475045 | 10 |
| 2383 | NR3C1 | RELA | -20.92920598 | -102.8494003 | 10 |
| 2384 | NUP214 | RAN | -20.90741217 | -64.82452094 | 5 |
| 2385 | CREG1 | TAF1 | -20.90380449 | -42.11120818 | 4 |
| 2386 | PLCG1 | TRPV4 | -20.89780116 | -48.61953751 | 5 |
| 2387 | PLCG1 | SLA | -20.89780116 | -53.80761499 | 5 |
| 2388 | EDG5 | RIC8 | -20.89474204 | -33.0245504 | 3 |
| 2389 | ALS2CR2 | MAP3K7IP1 | -20.89474204 | -34.21806863 | 3 |
| 2390 | SH3KBP1 | CRKL | -20.89434746 | -62.63038432 | 6 |
| 2391 | OPRM1 | HRH4 | -20.89270358 | -35.15698447 | 3 |
| 2392 | BAK1 | BIK | -20.89270358 | -34.15394096 | 3 |
| 2393 | TGFBI | ANTXR2 | -20.89270358 | -37.71356558 | 3 |
| 2394 | RGS16 | EDG5 | -20.89270358 | -33.0245504 | 3 |
| 2395 | VAMP8 | STXBP2 | -20.89270358 | -34.79913375 | 3 |
| 2396 | VAMP8 | STXBP6 | -20.89270358 | -34.67128127 | 3 |
| 2397 | ZNFN1A3 | ZNFN1A2 | -20.89270358 | -40.80460803 | 3 |
| 2398 | PLXNB1 | PLXNB2 | -20.89270358 | -43.28951468 | 3 |
| 2399 | BMP7 | INHBA | -20.88908071 | -48.76246431 | 4 |
| 2400 | MARS | JTV1 | -20.88908071 | -62.57345919 | 4 |
| 2401 | SRC | BMX | -20.88884646 | -67.24101641 | 6 |
| 2402 | ACVR2A | BMPR2 | -20.88799279 | -57.44504086 | 4 |
| 2403 | SNRPE | SNRPB | -20.88799279 | -52.88254624 | 4 |
| 2404 | MST1R | SHB | -20.86004407 | -34.74794017 | 4 |
| 2405 | CD19 | PLCG1 | -20.85738408 | -62.14228453 | 6 |
| 2406 | LDOC1 | Cep70 | -20.85735338 | -57.20039425 | 5 |
| 2407 | TYK2 | SHC1 | -20.8541998 | -74.10142508 | 7 |
| 2408 | LSM3 | LSM8 | -20.85166701 | -63.71283657 | 5 |
| 2409 | PDGFRB | VAV3 | -20.85146522 | -43.70632075 | 5 |
| 2410 | PDGFRB | SYN1 | -20.85146522 | -46.22530209 | 5 |
| 2411 | SLC9A3R1 | PDZK1 | -20.85146522 | -73.56805258 | 5 |
| 2412 | KIT | CD28 | -20.84281685 | -47.37315082 | 5 |
| 2413 | KIT | CSF2RB | -20.84281685 | -49.75848271 | 5 |
| 2414 | ACTN2 | ACTN1 | -20.8422236 | -72.68301439 | 5 |
| 2415 | PLAU | F10 | -20.8421528 | -49.96995361 | 4 |
| 2416 | ABLIM1 | KIAA1267 | -20.8421528 | -39.10680718 | 4 |
| 2417 | ABLIM1 | C16orf48 | -20.8421528 | -41.3787893 | 4 |
| 2418 | TCEB3B | C16orf48 | -20.8421528 | -41.33134957 | 4 |
| 2419 | KIAA1267 | C16orf48 | -20.8421528 | -38.81394375 | 4 |
| 2420 | ACTN1 | ACTN4 | -20.83882016 | -71.40208054 | 5 |
| 2421 | ARRB1 | ARRB2 | -20.83882016 | -67.87432044 | 5 |
| 2422 | TIF1 | BAG1 | -20.83882016 | -50.77776183 | 5 |
| 2423 | JAK1 | SOCS1 | -20.83585494 | -67.55799928 | 6 |
| 2424 | NSD1 | TIF1 | -20.83555812 | -38.6236474 | 4 |
| 2425 | GNA13 | GNAQ | -20.83397165 | -85.91565435 | 6 |
| 2426 | CD4 | CD3Z | -20.82906468 | -75.56367869 | 6 |
| 2427 | DDIT3 | HLF | -20.82685122 | -51.70756097 | 4 |
| 2428 | DDIT3 | NFE2L1 | -20.82685122 | -46.48529196 | 4 |
| 2429 | IKBKB | MAP3K8 | -20.82685122 | -41.98989956 | 4 |
| 2430 | C3 | C4B | -20.82685122 | -58.43032446 | 4 |
| 2431 | IXL | MED6 | -20.82685122 | -57.53423644 | 4 |
| 2432 | SMAD3 | SKI | -20.82403321 | -62.62088743 | 6 |
| 2433 | PTPN11 | PIK3R2 | -20.80215093 | -58.10090593 | 6 |
| 2434 | ABL1 | PTK2B | -20.78575594 | -84.37703888 | 8 |
| 2435 | TUBA1 | TUBB | -20.77449455 | -68.55011798 | 5 |
| 2436 | PDGFRB | PXN | -20.77300946 | -67.89708596 | 7 |
| 2437 | MAPK8 | MAP2K7 | -20.76027992 | -68.56746762 | 5 |
| 2438 | PRKCA | PRKCG | -20.75885829 | -65.27893817 | 5 |
| 2439 | ESR1 | RB1 | -20.75051509 | -128.6430993 | 11 |
| 2440 | GHR | RET | -20.74063838 | -47.58715512 | 5 |
| 2441 | JAK3 | TYK2 | -20.7399577 | -59.96272567 | 5 |
| 2442 | HIF1A | AHR | -20.7399577 | -59.55783498 | 5 |
| 2443 | RAF1 | RIN1 | -20.72600715 | -54.30866388 | 5 |
| 2444 | PML | HIPK2 | -20.72588562 | -67.2842031 | 6 |
| 2445 | E2F4 | HDAC1 | -20.71893513 | -63.69145322 | 6 |
| 2446 | NR3C1 | TRIP4 | -20.71267668 | -57.27294133 | 6 |
| 2447 | RELA | TRIP4 | -20.71267668 | -56.82618725 | 6 |
| 2448 | CSF3R | CSF2RB | -20.69652568 | -37.758215 | 4 |
| 2449 | ACVR2B | INHBB | -20.6938057 | -54.49709925 | 4 |
| 2450 | ETS1 | ETS2 | -20.6938057 | -42.327147 | 4 |
| 2451 | SNX6 | TGFBRAP1 | -20.6938057 | -47.65574928 | 4 |
| 2452 | UPF2 | RENT1 | -20.6938057 | -55.8580758 | 4 |
| 2453 | MAP3K14 | USP7 | -20.6938057 | -40.87083844 | 4 |
| 2454 | PSMA1 | MGC3162 | -20.6938057 | -44.01192313 | 4 |
| 2455 | KIT | EGFR | -20.69334861 | -83.88948553 | 8 |
| 2456 | ORC4L | MCM3 | -20.68674749 | -48.52237664 | 4 |
| 2457 | BAD | BAK1 | -20.68239523 | -47.68531283 | 4 |
| 2458 | RIPK2 | TNFRSF11A | -20.68239523 | -42.22076515 | 4 |
| 2459 | NDP52 | KIAA0980 | -20.67994432 | -89.70913547 | 7 |
| 2460 | NCOR2 | JUN | -20.67795751 | -81.78459267 | 8 |
| 2461 | PTPN6 | KIT | -20.67295943 | -70.43201716 | 7 |
| 2462 | PIK3R1 | RICS | -20.66430654 | -73.10514889 | 7 |
| 2463 | PIK3R1 | GRB10 | -20.66430654 | -75.55055504 | 7 |
| 2464 | ORC5L | MCM2 | -20.6447754 | -49.35186605 | 4 |
| 2465 | CDC45L | MCM2 | -20.6447754 | -47.0636697 | 4 |
| 2466 | COL1A1 | HSPG2 | -20.64446192 | -67.18687128 | 5 |
| 2467 | PRKDC | XRCC5 | -20.64446192 | -57.7710619 | 5 |
| 2468 | GHR | VAV1 | -20.64241266 | -59.62490214 | 6 |
| 2469 | FADD | TNFRSF10B | -20.63552369 | -46.73848579 | 4 |
| 2470 | BRCA1 | TRIP4 | -20.62452201 | -55.63247947 | 6 |
| 2471 | SIT1 | PECAM1 | -20.62406388 | -39.59303744 | 4 |
| 2472 | SCAP1 | PECAM1 | -20.62406388 | -37.03803619 | 4 |
| 2473 | MUC1 | PTK2 | -20.61934439 | -59.72470721 | 6 |
| 2474 | LCP2 | SLA | -20.6175346 | -42.83476159 | 4 |
| 2475 | FLJ32855 | MGC17403 | -20.6175346 | -39.53048929 | 4 |
| 2476 | NCOA1 | NFKB1 | -20.61716231 | -87.7676884 | 8 |
| 2477 | MCM7 | CCDC5 | -20.59894537 | -48.94936099 | 4 |
| 2478 | PTK2B | CBLB | -20.59608747 | -56.97236745 | 6 |
| 2479 | KHDRBS1 | LAT | -20.59465295 | -53.4570806 | 5 |
| 2480 | SHC1 | CAV1 | -20.58653358 | -91.23509758 | 9 |
| 2481 | GAB3 | PTK2 | -20.56935305 | -43.29673878 | 5 |
| 2482 | ZBTB16 | CBFA2T2 | -20.54839511 | -56.59642427 | 5 |
| 2483 | CSF1R | IRS1 | -20.5436269 | -53.25735912 | 5 |
| 2484 | VAV3 | IRS1 | -20.5436269 | -47.11083147 | 5 |
| 2485 | ZAP70 | CD5 | -20.53912663 | -51.42036953 | 5 |
| 2486 | KLKB1 | F11 | -20.53806726 | -40.31690483 | 3 |
| 2487 | DLL1 | MAML1 | -20.53806726 | -38.70966231 | 3 |
| 2488 | TGIF | TCF8 | -20.53806726 | -32.87450975 | 3 |
| 2489 | U2AF1L2 | SFRS2IP | -20.53806726 | -36.24973164 | 3 |
| 2490 | DUSP22 | MAP3K2 | -20.53806726 | -36.23765906 | 3 |
| 2491 | TNFRSF18 | TNFRSF17 | -20.53806726 | -30.56167018 | 3 |
| 2492 | TNFRSF12A | TNFRSF17 | -20.53806726 | -30.56167018 | 3 |
| 2493 | HUS1B | RAD1 | -20.53806726 | -41.70984893 | 3 |
| 2494 | RAD9B | RAD1 | -20.53806726 | -42.21337525 | 3 |
| 2495 | ARPC5 | ARPC1B | -20.53806726 | -43.22052181 | 3 |
| 2496 | ARPC5 | ARPC2 | -20.53806726 | -43.22052181 | 3 |
| 2497 | ARPC5 | ARPC4 | -20.53806726 | -43.22052181 | 3 |
| 2498 | DCTD | CUTC | -20.53806726 | -32.26350042 | 3 |
| 2499 | CITED2 | CITED4 | -20.53725193 | -39.19417062 | 3 |
| 2500 | CCL8 | CCL16 | -20.53725193 | -38.28892973 | 3 |
| 2501 | IL10RB | IL28RA | -20.53725193 | -51.34467242 | 3 |
| 2502 | NRG1 | BTC | -20.53725193 | -31.76459477 | 3 |
| 2503 | NGB | RGS18 | -20.53725193 | -33.37936603 | 3 |
| 2504 | NGB | RGS5 | -20.53725193 | -34.36197941 | 3 |
| 2505 | SSTR2 | SSTR3 | -20.53725193 | -39.66784398 | 3 |
| 2506 | IL1R1 | IL1R2 | -20.53725193 | -43.24299467 | 3 |
| 2507 | PPFIBP2 | PTPRS | -20.53725193 | -39.52367419 | 3 |
| 2508 | PPFIBP2 | PTPRD | -20.53725193 | -39.52367419 | 3 |
| 2509 | PPFIBP2 | PPFIBP1 | -20.53725193 | -39.52367419 | 3 |
| 2510 | CCL2 | CCL11 | -20.53725193 | -37.44163187 | 3 |
| 2511 | CCL2 | CCL13 | -20.53725193 | -37.44163187 | 3 |
| 2512 | RASIP1 | RAPGEF5 | -20.53725193 | -39.64145722 | 3 |
| 2513 | SPAG9 | DUSP22 | -20.53725193 | -33.58569833 | 3 |
| 2514 | ARPC3 | ARPC5 | -20.53725193 | -43.22052181 | 3 |
| 2515 | POLR2C | POLR2G | -20.53725193 | -41.96206082 | 3 |
| 2516 | JAK2 | GAB3 | -20.53662417 | -44.25593 | 5 |
| 2517 | RHOA | RHOG | -20.53610678 | -68.27978555 | 5 |
| 2518 | SYK | FYN | -20.53390922 | -98.75708694 | 9 |
| 2519 | CCR2 | CCR3 | -20.52217259 | -59.12347164 | 4 |
| 2520 | TCF4 | MYOG | -20.5205406 | -43.68391302 | 4 |
| 2521 | ARNT | ARNTL | -20.5205406 | -55.87365102 | 4 |
| 2522 | TRIP4 | TDG | -20.5205406 | -36.99632035 | 4 |
| 2523 | TNFAIP3 | TANK | -20.5205406 | -42.65251179 | 4 |
| 2524 | CBLB | SOCS1 | -20.51843788 | -51.70701055 | 5 |
| 2525 | SOCS1 | SHB | -20.51571494 | -52.16993765 | 5 |
| 2526 | ASCL2 | NEUROD1 | -20.48927693 | -32.95758362 | 3 |
| 2527 | PPEF2 | NEUROD1 | -20.48927693 | -32.95758362 | 3 |
| 2528 | ST13 | STUB1 | -20.48927693 | -32.46631354 | 3 |
| 2529 | IGSF8 | CD63 | -20.48927693 | -37.60885463 | 3 |
| 2530 | TGFBR3 | SLITL2 | -20.48683061 | -37.19786872 | 3 |
| 2531 | TGFBR3 | FMOD | -20.48683061 | -37.19786872 | 3 |
| 2532 | SPRY2 | EGFL9 | -20.48683061 | -31.73230771 | 3 |
| 2533 | NEUROD1 | PPEF1 | -20.48683061 | -32.95758362 | 3 |
| 2534 | RPL5 | RPL23A | -20.48683061 | -39.50532505 | 3 |
| 2535 | RPL5 | RPS7 | -20.48683061 | -39.50532505 | 3 |
| 2536 | BIRC5 | BIRC7 | -20.48683061 | -35.24822133 | 3 |
| 2537 | OSM | ANTXR2 | -20.48683061 | -37.71356558 | 3 |
| 2538 | UPF3A | MAGOH | -20.48683061 | -41.08124027 | 3 |
| 2539 | SP3 | SP4 | -20.48683061 | -36.17318548 | 3 |
| 2540 | PER3 | TIMELESS | -20.48683061 | -41.70254963 | 3 |
| 2541 | DGKZ | SCN4A | -20.48683061 | -39.93610797 | 3 |
| 2542 | DGKZ | SCN5A | -20.48683061 | -39.93610797 | 3 |
| 2543 | DTNA | SCN4A | -20.48683061 | -39.93610797 | 3 |
| 2544 | DTNA | SCN5A | -20.48683061 | -39.93610797 | 3 |
| 2545 | ARHGEF7 | ARHGEF6 | -20.48683061 | -37.98578343 | 3 |
| 2546 | TP53 | HNRPU | -20.47551627 | -70.50581362 | 6 |
| 2547 | PTPRC | CBL | -20.47488427 | -69.18240697 | 7 |
| 2548 | PDGFRB | CBL | -20.47488427 | -64.01996578 | 7 |
| 2549 | KRT15 | EFCBP2 | -20.46907623 | -78.43480312 | 6 |
| 2550 | RAPGEF1 | PTK2B | -20.45560266 | -49.51350002 | 5 |
| 2551 | MCM10 | CDC45L | -20.4529298 | -46.6777174 | 4 |
| 2552 | BLNK | EPOR | -20.44852018 | -48.70398185 | 5 |
| 2553 | PAG1 | NEDD9 | -20.44852018 | -47.68578834 | 5 |
| 2554 | LSM4 | LSM8 | -20.44852018 | -63.85593742 | 5 |
| 2555 | KIT | PAG1 | -20.44035024 | -46.35592982 | 5 |
| 2556 | KIT | BCR | -20.44035024 | -48.62070965 | 5 |
| 2557 | EPOR | IL4R | -20.44035024 | -50.48223222 | 5 |
| 2558 | SYK | EGFR | -20.43865402 | -88.84120948 | 9 |
| 2559 | TLN1 | ILK | -20.4376404 | -44.10562009 | 4 |
| 2560 | CD22 | VAV3 | -20.4376404 | -34.99545444 | 4 |
| 2561 | PPP5C | PDCD6 | -20.4376404 | -50.75853217 | 4 |
| 2562 | PPP5C | RDX | -20.4376404 | -50.32181452 | 4 |
| 2563 | BRAF | CDC25B | -20.4376404 | -43.6177529 | 4 |
| 2564 | RUNX2 | RUNX1 | -20.4376404 | -47.31583994 | 4 |
| 2565 | CDC27 | SKIL | -20.4370964 | -45.7615246 | 4 |
| 2566 | SNRPE | LSM6 | -20.4370964 | -50.7483798 | 4 |
| 2567 | APP | OSM | -20.42833978 | -63.8657166 | 5 |
| 2568 | MCM5 | ORC2L | -20.41902817 | -48.85451047 | 4 |
| 2569 | PSMC2 | GTF2E2 | -20.41902817 | -46.11247791 | 4 |
| 2570 | DLG2 | LIN7B | -20.41413154 | -47.76509694 | 4 |
| 2571 | CDC25A | RIN1 | -20.41413154 | -41.84773342 | 4 |
| 2572 | ERBB2 | VAV1 | -20.40612445 | -66.14981127 | 7 |
| 2573 | ZFYVE9 | STRAP | -20.39425509 | -37.55189878 | 4 |
| 2574 | RIBC2 | MGC17403 | -20.39425509 | -39.53048929 | 4 |
| 2575 | ADAM15 | PAG1 | -20.38705144 | -36.3632432 | 4 |
| 2576 | CSF3R | BCR | -20.38705144 | -35.79009364 | 4 |
| 2577 | CDC7 | MCM6 | -20.38705144 | -50.27157649 | 4 |
| 2578 | DGKZ | KCNJ12 | -20.38705144 | -53.24433628 | 4 |
| 2579 | DTNA | KCNJ12 | -20.38705144 | -53.24433628 | 4 |
| 2580 | GAB2 | INPP5D | -20.38378725 | -37.39558847 | 4 |
| 2581 | PAG1 | INPP5D | -20.38378725 | -35.68479806 | 4 |
| 2582 | RBBP4 | SP3 | -20.38378725 | -37.85205054 | 4 |
| 2583 | RRAS2 | RRAS | -20.38378725 | -52.12770549 | 4 |
| 2584 | KCNJ12 | LIN7A | -20.38378725 | -44.17987877 | 4 |
| 2585 | BIN1 | AMPH | -20.38378725 | -51.68986139 | 4 |
| 2586 | TU3A | C1orf65 | -20.38378725 | -37.47055141 | 4 |
| 2587 | EPHA2 | PDGFRB | -20.38257602 | -44.58746245 | 5 |
| 2588 | ERBB2 | VAV3 | -20.37476753 | -43.70632075 | 5 |
| 2589 | TNFRSF1A | BIRC2 | -20.37476753 | -55.97464796 | 5 |
| 2590 | SRC | SYK | -20.37084082 | -88.61368147 | 9 |
| 2591 | KRT18 | TNFAIP3 | -20.36963764 | -54.6410957 | 5 |
| 2592 | GTF2B | GTF2E2 | -20.36199348 | -60.41902195 | 5 |
| 2593 | HSPCA | DAP3 | -20.35411863 | -53.69258236 | 5 |
| 2594 | BLNK | PLCG1 | -20.35352969 | -68.45966571 | 6 |
| 2595 | CSF3R | CBL | -20.35078926 | -48.36747249 | 5 |
| 2596 | INPP5D | CBL | -20.35078926 | -50.10710289 | 5 |
| 2597 | EVL | CBL | -20.35078926 | -47.78643322 | 5 |
| 2598 | MAPK3 | MAPK8 | -20.34679183 | -88.76477824 | 7 |
| 2599 | PCTK1 | KRT18 | -20.34114746 | -43.8559258 | 4 |
| 2600 | REM1 | KRT18 | -20.34114746 | -42.31369664 | 4 |
| 2601 | XPO1 | RANBP5 | -20.33722945 | -61.76738004 | 5 |
| 2602 | PIK3R1 | STAT5A | -20.33416727 | -84.16454666 | 8 |
| 2603 | KRT18 | KIF1C | -20.32644557 | -43.6682423 | 4 |
| 2604 | ABL1 | RAPGEF1 | -20.3228429 | -52.24625783 | 5 |
| 2605 | KRT15 | KCTD17 | -20.3228429 | -57.19493078 | 5 |
| 2606 | GRB2 | CBLB | -20.31491399 | -87.82148635 | 8 |
| 2607 | CALD1 | KCNQ5 | -20.31465199 | -32.95758362 | 3 |
| 2608 | CALD1 | GRM5 | -20.31465199 | -32.95758362 | 3 |
| 2609 | KCNJ6 | KCNJ9 | -20.31465199 | -41.37849179 | 3 |
| 2610 | PCTK1 | REM1 | -20.31465199 | -31.52852674 | 3 |
| 2611 | PCTK1 | KIF1C | -20.31465199 | -32.8830724 | 3 |
| 2612 | REM1 | KIF1C | -20.31465199 | -31.34084324 | 3 |
| 2613 | IQGAP2 | KTN1 | -20.31465199 | -33.0846776 | 3 |
| 2614 | KCNQ5 | GRM5 | -20.31465199 | -32.95758362 | 3 |
| 2615 | RPS6KA4 | DUSP1 | -20.31465199 | -30.5592851 | 3 |
| 2616 | DSC1 | DSG2 | -20.31465199 | -39.60421305 | 3 |
| 2617 | DSC2 | DSG2 | -20.31465199 | -39.60421305 | 3 |
| 2618 | DSG1 | DSG2 | -20.31465199 | -39.09338743 | 3 |
| 2619 | RFX2 | RFX3 | -20.31465199 | -44.49348749 | 3 |
| 2620 | PMAIP1 | BID | -20.31465199 | -34.15394096 | 3 |
| 2621 | PBP | KSR2 | -20.31465199 | -31.81971726 | 3 |
| 2622 | KCNA4 | ATP2B4 | -20.31465199 | -31.63938458 | 3 |
| 2623 | KCNA4 | GUCY1A2 | -20.31465199 | -31.63938458 | 3 |
| 2624 | AKAP10 | AKAP11 | -20.31465199 | -37.79782592 | 3 |
| 2625 | DUSP1 | DUSP10 | -20.31465199 | -31.34195373 | 3 |
| 2626 | DUSP1 | SNCG | -20.31465199 | -30.7728592 | 3 |
| 2627 | TNFRSF7 | TNFRSF19 | -20.31465199 | -31.98570487 | 3 |
| 2628 | PFDN4 | PIGT | -20.31465199 | -35.43373748 | 3 |
| 2629 | NLGN3 | ATP2B4 | -20.31465199 | -33.20702537 | 3 |
| 2630 | NLGN3 | GUCY1A2 | -20.31465199 | -33.20702537 | 3 |
| 2631 | PLCB2 | PLCB1 | -20.31465199 | -35.88699813 | 3 |
| 2632 | SEMA4C | ATP2B4 | -20.31465199 | -31.41037591 | 3 |
| 2633 | SEMA4C | GUCY1A2 | -20.31465199 | -31.41037591 | 3 |
| 2634 | HNRPH1 | HNRPF | -20.31465199 | -43.86933318 | 3 |
| 2635 | TNFRSF9 | TNFRSF19 | -20.31465199 | -30.56167018 | 3 |
| 2636 | AFTIPHILIN | M6PR | -20.31465199 | -41.23539095 | 3 |
| 2637 | GHR | PIK3R1 | -20.28521582 | -68.02739851 | 7 |
| 2638 | GADD45G | NRIP1 | -20.27943916 | -52.95444188 | 5 |
| 2639 | BRCA1 | PELP1 | -20.27805667 | -45.28409163 | 5 |
| 2640 | DNM1 | SOS1 | -20.27508811 | -55.94089312 | 5 |
| 2641 | SHB | WAS | -20.27508811 | -46.72116742 | 5 |
| 2642 | TUBA1 | TUBB2 | -20.27508811 | -68.55011798 | 5 |
| 2643 | SOS1 | RICS | -20.2716842 | -49.80778574 | 5 |
| 2644 | PTK2 | FYN | -20.26922573 | -102.7906664 | 10 |
| 2645 | CSF1R | SHC1 | -20.2505606 | -60.34284751 | 6 |
| 2646 | JUN | HDAC1 | -20.2472183 | -111.0092971 | 11 |
| 2647 | RHOA | RHOC | -20.23489686 | -59.7520803 | 4 |
| 2648 | KHDRBS1 | SYK | -20.22528818 | -54.65270038 | 6 |
| 2649 | PTK2B | LCP2 | -20.22247017 | -58.96550749 | 6 |
| 2650 | TYK2 | SOCS1 | -20.21469314 | -52.22280887 | 5 |
| 2651 | PTPN1 | SOCS1 | -20.21469314 | -48.01287027 | 5 |
| 2652 | NIF3L1 | PSMF1 | -20.20574469 | -54.04944719 | 5 |
| 2653 | SYK | CD22 | -20.20383518 | -49.21833759 | 5 |
| 2654 | ZBTB16 | TIF1 | -20.19938076 | -61.60824212 | 6 |
| 2655 | FOSL1 | ATF4 | -20.19042856 | -44.36769865 | 4 |
| 2656 | IFNAR1 | IRS4 | -20.18434099 | -38.52550728 | 4 |
| 2657 | RIPK1 | TRPC4AP | -20.18281003 | -42.1078728 | 4 |
| 2658 | RIPK1 | TNFRSF8 | -20.18281003 | -43.16570148 | 4 |
| 2659 | ATF4 | FOSL2 | -20.18281003 | -43.51612424 | 4 |
| 2660 | GAB3 | SHB | -20.16825848 | -36.62127963 | 4 |
| 2661 | ERCC2 | GTF2H1 | -20.16825848 | -48.87963051 | 4 |
| 2662 | APEX2 | KIAA0408 | -20.16825848 | -39.80526795 | 4 |
| 2663 | JAK2 | SRC | -20.16434932 | -101.0770695 | 10 |
| 2664 | STX4A | VAMP8 | -20.16281741 | -49.08399593 | 4 |
| 2665 | LDOC1 | ZBTB8 | -20.13980544 | -57.52665237 | 5 |
| 2666 | PML | AR | -20.13930751 | -86.66164082 | 9 |
| 2667 | NR3C1 | FOS | -20.1374789 | -85.23477807 | 8 |
| 2668 | NR3C1 | CEBPB | -20.1374789 | -90.4135042 | 8 |
| 2669 | RELA | CEBPB | -20.1374789 | -87.66340189 | 8 |
| 2670 | ASCL2 | MYOG | -20.13260199 | -32.95758362 | 3 |
| 2671 | PPEF2 | MYOG | -20.13260199 | -32.95758362 | 3 |
| 2672 | PPEF1 | MYOG | -20.13260199 | -32.95758362 | 3 |
| 2673 | ENG | SLITL2 | -20.12974776 | -37.19786872 | 3 |
| 2674 | ENG | FMOD | -20.12974776 | -37.19786872 | 3 |
| 2675 | RBPSUH | JAG2 | -20.12974776 | -38.70966231 | 3 |
| 2676 | GPS2 | CORO2A | -20.12974776 | -38.2072234 | 3 |
| 2677 | TYK2 | CBL | -20.12096767 | -57.74601884 | 6 |
| 2678 | JAK2 | IGF1R | -20.11950996 | -75.16772546 | 7 |
| 2679 | COL1A1 | COL3A1 | -20.11097756 | -53.24231812 | 4 |
| 2680 | NRIP1 | THRAP4 | -20.11097756 | -42.16927198 | 4 |
| 2681 | EPOR | PLCG1 | -20.10648336 | -71.71931343 | 7 |
| 2682 | MYC | HDAC1 | -20.10497649 | -97.00327359 | 9 |
| 2683 | BIRC5 | BIRC4 | -20.10005015 | -49.65506193 | 4 |
| 2684 | CDC7 | MCM3 | -20.10005015 | -50.47410075 | 4 |
| 2685 | TOB1 | LEF1 | -20.10005015 | -36.88999119 | 4 |
| 2686 | USP7 | RIPK2 | -20.10005015 | -42.22076515 | 4 |
| 2687 | MXD1 | SIN3A | -20.09762996 | -55.08759231 | 5 |
| 2688 | MUC1 | EGF | -20.09624166 | -39.34136781 | 4 |
| 2689 | GADD45G | ERBP | -20.09624166 | -40.02920582 | 4 |
| 2690 | SUV39H1 | SP3 | -20.09624166 | -40.64292346 | 4 |
| 2691 | RIPK2 | TRAF3IP2 | -20.09624166 | -42.86739232 | 4 |
| 2692 | SIN3A | SAP30 | -20.08060113 | -61.58800725 | 5 |
| 2693 | EP300 | TP53 | -20.07150901 | -161.2561469 | 13 |
| 2694 | CASP8 | CASP4 | -20.0712524 | -55.17222793 | 4 |
| 2695 | E2F4 | SKIIP | -20.07086845 | -41.127605 | 4 |
| 2696 | E2F4 | PHB | -20.07086845 | -41.22363764 | 4 |
| 2697 | EDF1 | NR0B2 | -20.06978039 | -53.57041636 | 4 |
| 2698 | NUP214 | RANBP2 | -20.06978039 | -50.75415258 | 4 |
| 2699 | TRIP4 | EGR1 | -20.06978039 | -36.59672259 | 4 |
| 2700 | FBF1 | KIAA1267 | -20.06978039 | -37.19880782 | 4 |
| 2701 | NUP98 | RANBP2 | -20.06978039 | -48.48282003 | 4 |
| 2702 | POU2F1 | NCOA6 | -20.06865571 | -55.26157556 | 5 |
| 2703 | HNRPK | KHDRBS3 | -20.06865571 | -65.59375043 | 5 |
| 2704 | TRIM37 | HOOK2 | -20.06865571 | -61.69042631 | 5 |
| 2705 | IKBKG | TRAF6 | -20.06765001 | -79.41051111 | 6 |
| 2706 | GAB2 | STAT5A | -20.06529659 | -51.31963128 | 5 |
| 2707 | NCOA1 | DDX54 | -20.06526387 | -43.33769985 | 4 |
| 2708 | NCOA1 | ONECUT1 | -20.06526387 | -44.05918723 | 4 |
| 2709 | EPHA2 | IRS1 | -20.06139243 | -44.58746245 | 5 |
| 2710 | TNFAIP3 | IRS1 | -20.06139243 | -54.26742092 | 5 |
| 2711 | SMAD3 | SKIL | -20.04789297 | -67.80701028 | 6 |
| 2712 | CASP8 | CASP2 | -20.04709016 | -70.74667369 | 5 |
| 2713 | JUN | DDIT3 | -20.04517722 | -87.78957742 | 7 |
| 2714 | MCM10 | KIAA0408 | -20.03916122 | -55.46457994 | 5 |
| 2715 | SNRPE | LSM5 | -20.03272017 | -52.22998434 | 4 |
| 2716 | FOXO1A | CDC25B | -20.03272017 | -37.03150805 | 4 |
| 2717 | HDAC4 | HDAC3 | -20.02410743 | -76.33694938 | 6 |
| 2718 | PML | RXRA | -20.02036796 | -88.11092915 | 8 |
| 2719 | APP | FN1 | -19.99613474 | -101.9622744 | 8 |
| 2720 | PELP1 | TIF1 | -19.99016661 | -39.06103373 | 4 |
| 2721 | RAF1 | GRB10 | -19.98667311 | -62.5694626 | 6 |
| 2722 | IKBKB | TRPC4AP | -19.98200334 | -41.78047607 | 4 |
| 2723 | IKBKG | TRPC4AP | -19.98200334 | -45.42069035 | 4 |
| 2724 | MYF6 | RAB3B | -19.97858742 | -32.95758362 | 3 |
| 2725 | MYF6 | MYF5 | -19.97858742 | -32.95758362 | 3 |
| 2726 | MYF6 | KCNQ3 | -19.97858742 | -32.95758362 | 3 |
| 2727 | MYF6 | RALB | -19.97858742 | -32.95758362 | 3 |
| 2728 | EMP3 | RALB | -19.97858742 | -32.95758362 | 3 |
| 2729 | CCNC | MED25 | -19.97858742 | -40.14968207 | 3 |
| 2730 | CCNC | CDK8 | -19.97858742 | -36.8644836 | 3 |
| 2731 | CCNC | SURB7 | -19.97858742 | -36.8644836 | 3 |
| 2732 | LTBP3 | CTGF | -19.97858742 | -37.19786872 | 3 |
| 2733 | TNFRSF4 | TNFRSF8 | -19.97858742 | -31.98570487 | 3 |
| 2734 | TNFRSF18 | TNFRSF8 | -19.97858742 | -30.56167018 | 3 |
| 2735 | TNFRSF12A | TNFRSF8 | -19.97858742 | -30.56167018 | 3 |
| 2736 | OTX1 | CATSPER1 | -19.97858742 | -31.69257754 | 3 |
| 2737 | CALCRL | ADM2 | -19.97736435 | -46.15171557 | 3 |
| 2738 | RAB3B | EMP3 | -19.97736435 | -32.95758362 | 3 |
| 2739 | RAB3B | KCNQ2 | -19.97736435 | -32.95758362 | 3 |
| 2740 | MYF5 | EMP3 | -19.97736435 | -32.95758362 | 3 |
| 2741 | MYF5 | KCNQ2 | -19.97736435 | -32.95758362 | 3 |
| 2742 | KCNQ3 | EMP3 | -19.97736435 | -32.95758362 | 3 |
| 2743 | KCNQ3 | KCNQ2 | -19.97736435 | -32.95758362 | 3 |
| 2744 | RGS14 | RGS18 | -19.97736435 | -33.37936603 | 3 |
| 2745 | RGS14 | RGS5 | -19.97736435 | -34.36197941 | 3 |
| 2746 | CCL7 | CCL16 | -19.97736435 | -38.28892973 | 3 |
| 2747 | PPP4C | NKRF | -19.97736435 | -32.25486699 | 3 |
| 2748 | RAB8A | RAB3D | -19.97736435 | -41.6519059 | 3 |
| 2749 | RALB | KCNQ2 | -19.97736435 | -32.95758362 | 3 |
| 2750 | HBP1 | PHF12 | -19.97736435 | -32.42661977 | 3 |
| 2751 | MYCBP | UBE2M | -19.97736435 | -38.99074889 | 3 |
| 2752 | KCNJ2 | KCNJ10 | -19.97736435 | -31.52160155 | 3 |
| 2753 | TRPC4AP | RIPK3 | -19.97736435 | -30.62168167 | 3 |
| 2754 | KCNJ4 | KCNJ10 | -19.97736435 | -31.52160155 | 3 |
| 2755 | KHDRBS1 | LCP2 | -19.96875654 | -56.06431268 | 5 |
| 2756 | NIF3L1 | FXR2 | -19.95630983 | -77.41989579 | 7 |
| 2757 | CHUK | MAP3K8 | -19.95036247 | -41.19139186 | 4 |
| 2758 | CREBBP | NCOA1 | -19.94534009 | -101.5126221 | 9 |
| 2759 | CREBBP | NFKB1 | -19.94534009 | -100.4211191 | 9 |
| 2760 | PTPN6 | IRS2 | -19.93905839 | -62.8011684 | 6 |
| 2761 | PDGFRB | SNX6 | -19.93148562 | -60.79312073 | 5 |
| 2762 | THBS1 | SPARC | -19.93148562 | -59.04902592 | 5 |
| 2763 | PPARG | ESR2 | -19.92864819 | -60.94375048 | 5 |
| 2764 | DDIT3 | ATF2 | -19.92728653 | -51.27014715 | 5 |
| 2765 | KNG1 | SERPINB13 | -19.92642893 | -50.50609704 | 4 |
| 2766 | PECAM1 | NPHS1 | -19.92642893 | -39.44046662 | 4 |
| 2767 | CDC42 | RHOG | -19.92176227 | -69.39366531 | 5 |
| 2768 | EGFR | FYN | -19.91876669 | -131.4881304 | 12 |
| 2769 | PDGFRB | IGF1R | -19.89584778 | -63.43290923 | 6 |
| 2770 | IL2RB | PTK2B | -19.89540322 | -57.81005484 | 6 |
| 2771 | ERBB2 | PTPN12 | -19.8933497 | -46.25603965 | 5 |
| 2772 | TNFRSF1A | TNFAIP3 | -19.8933497 | -53.90661637 | 5 |
| 2773 | GAB2 | BCAR1 | -19.88433966 | -48.92298638 | 5 |
| 2774 | PAG1 | BCAR1 | -19.88433966 | -46.03330073 | 5 |
| 2775 | MCM6 | MCM7 | -19.88433966 | -67.84141246 | 5 |
| 2776 | AP1B1 | AP1G1 | -19.87717908 | -51.33628723 | 4 |
| 2777 | GLI3 | SNIP1 | -19.87717908 | -37.28205959 | 4 |
| 2778 | SNRPB | DDX20 | -19.87717908 | -51.67572036 | 4 |
| 2779 | BCL6 | CBFA2T2 | -19.87445873 | -44.82864099 | 4 |
| 2780 | TRAF1 | RIPK2 | -19.87276477 | -57.14134773 | 5 |
| 2781 | APP | MMP9 | -19.87166836 | -76.19311567 | 6 |
| 2782 | AR | ESR2 | -19.86542818 | -81.83261046 | 7 |
| 2783 | JAK3 | PIK3R1 | -19.86498547 | -77.46924466 | 7 |
| 2784 | PIK3R1 | GNB2L1 | -19.85597883 | -97.19691546 | 9 |
| 2785 | LCK | LCP2 | -19.84974908 | -63.07134948 | 6 |
| 2786 | PML | CHD3 | -19.84882472 | -50.97507996 | 5 |
| 2787 | ABLIM1 | LNX | -19.84563222 | -50.92622558 | 5 |
| 2788 | TCEB3B | LNX | -19.84563222 | -52.82220336 | 5 |
| 2789 | GTF2H5 | GTF2E2 | -19.83246704 | -50.37190223 | 4 |
| 2790 | FBN1 | ELN | -19.83246704 | -54.40318865 | 4 |
| 2791 | ENAH | VASP | -19.83246704 | -58.06259968 | 4 |
| 2792 | KIT | CRKL | -19.83166819 | -63.17424364 | 6 |
| 2793 | NEDD9 | CRKL | -19.83166819 | -64.57575839 | 6 |
| 2794 | IRS2 | CSF3R | -19.82811418 | -36.57427444 | 4 |
| 2795 | IRS2 | INPP5D | -19.82811418 | -38.47567098 | 4 |
| 2796 | NCF1 | NCF2 | -19.82811418 | -55.16311209 | 4 |
| 2797 | BCL2 | MCL1 | -19.82631564 | -68.25446774 | 5 |
| 2798 | LNX | KIAA1267 | -19.82382788 | -48.44166086 | 5 |
| 2799 | ASCL2 | GRM7 | -19.81414825 | -32.95758362 | 3 |
| 2800 | PPEF2 | GRM7 | -19.81414825 | -32.95758362 | 3 |
| 2801 | PPEF1 | GRM7 | -19.81414825 | -32.95758362 | 3 |
| 2802 | ANTXR2 | MATN2 | -19.81414825 | -37.71356558 | 3 |
| 2803 | IGFBP1 | IGFBP5 | -19.81414825 | -38.99164636 | 3 |
| 2804 | IGFBP2 | IGFBP5 | -19.81414825 | -38.99164636 | 3 |
| 2805 | E2F4 | RBBP9 | -19.81088605 | -32.33585507 | 3 |
| 2806 | NPDC1 | DMTF1 | -19.81088605 | -36.58867815 | 3 |
| 2807 | IGFBP5 | IGFBP6 | -19.81088605 | -38.99164636 | 3 |
| 2808 | MAP1A | ATP2B2 | -19.81088605 | -31.63938458 | 3 |
| 2809 | BRAF | UCP2 | -19.81088605 | -32.6448995 | 3 |
| 2810 | BRAF | UCP3 | -19.81088605 | -32.6448995 | 3 |
| 2811 | PPARD | HDAC10 | -19.81088605 | -31.25765249 | 3 |
| 2812 | RAB11A | RAB25 | -19.81088605 | -44.42894897 | 3 |
| 2813 | DTX2 | NEU4 | -19.81088605 | -30.75502354 | 3 |
| 2814 | AR | TRIP4 | -19.80786788 | -56.52915041 | 6 |
| 2815 | MST1R | WAS | -19.79952018 | -34.74794017 | 4 |
| 2816 | SCAP1 | WAS | -19.79952018 | -39.21531456 | 4 |
| 2817 | ASK | MCM2 | -19.79952018 | -49.10055162 | 4 |
| 2818 | ERBB2 | CBL | -19.79759522 | -66.5095194 | 7 |
| 2819 | KHDRBS1 | TUB | -19.7908121 | -35.87553999 | 4 |
| 2820 | KHDRBS1 | SCAP1 | -19.7908121 | -35.06991483 | 4 |
| 2821 | PSMA1 | NIF3L1 | -19.7806694 | -53.55503744 | 5 |
| 2822 | RET | CBL | -19.76948608 | -56.81387837 | 6 |
| 2823 | IRS4 | PTK2B | -19.76777764 | -46.11780638 | 5 |
| 2824 | GRB2 | PACSIN1 | -19.76657934 | -74.20669463 | 6 |
| 2825 | RAPGEF1 | PTK2 | -19.76470232 | -49.51350002 | 5 |
| 2826 | PTK2 | RICS | -19.75947706 | -57.37722639 | 6 |
| 2827 | PTK2 | PDGFRA | -19.75947706 | -64.4301441 | 6 |
| 2828 | TBP | GTF2H1 | -19.75947706 | -67.13393866 | 6 |
| 2829 | PTPN11 | GAB1 | -19.74892979 | -59.09697471 | 6 |
| 2830 | GTF2B | GTF2F2 | -19.73637007 | -56.42168361 | 5 |
| 2831 | GTF2B | AHR | -19.73637007 | -54.1857689 | 5 |
| 2832 | MCM10 | FLJ32855 | -19.73637007 | -53.60087381 | 5 |
| 2833 | CD22 | CD28 | -19.73534918 | -36.94538874 | 4 |
| 2834 | ABLIM1 | ZNF250 | -19.73534918 | -48.53161019 | 4 |
| 2835 | MYST2 | ZNF408 | -19.73534918 | -46.99465128 | 4 |
| 2836 | RAB4A | RAB5A | -19.73534918 | -50.38938295 | 4 |
| 2837 | CD28 | CTLA4 | -19.73371697 | -52.95211905 | 4 |
| 2838 | PEX19 | PEX5 | -19.73371697 | -61.4748469 | 4 |
| 2839 | PSMA1 | ABLIM1 | -19.73371697 | -42.05671924 | 4 |
| 2840 | PSMA1 | KIAA1267 | -19.73371697 | -40.31409187 | 4 |
| 2841 | ZNF250 | MYST2 | -19.73371697 | -48.30066557 | 4 |
| 2842 | PTK2 | CSF3R | -19.73265311 | -46.24569052 | 5 |
| 2843 | JUN | PELP1 | -19.72606393 | -45.50200414 | 5 |
| 2844 | MBD3 | HDAC2 | -19.72582435 | -59.79031305 | 5 |
| 2845 | KIT | IRS2 | -19.72334833 | -46.64560222 | 5 |
| 2846 | DIPA | Cep70 | -19.72001152 | -87.99245911 | 7 |
| 2847 | HIF1A | NPAS2 | -19.70328567 | -41.20217707 | 4 |
| 2848 | ATF2 | SPIB | -19.70328567 | -39.39621562 | 4 |
| 2849 | MMP9 | COL7A1 | -19.70328567 | -44.51203171 | 4 |
| 2850 | CCND1 | CCNA1 | -19.69891759 | -56.15964965 | 5 |
| 2851 | CASK | KCNJ12 | -19.69891759 | -59.35523812 | 5 |
| 2852 | SRC | BCAR1 | -19.6852835 | -85.10048822 | 8 |
| 2853 | CCND1 | CDK2 | -19.68510691 | -66.01759975 | 6 |
| 2854 | CDKN1A | MCM3 | -19.68434223 | -64.93017945 | 5 |
| 2855 | GRB2 | NEDD9 | -19.6825172 | -97.73679177 | 9 |
| 2856 | SRC | NCK1 | -19.68245272 | -99.92758892 | 9 |
| 2857 | JAK2 | CBL | -19.67218627 | -73.78218129 | 8 |
| 2858 | PML | RUNX1T1 | -19.67045728 | -61.69897878 | 6 |
| 2859 | BCL2 | SHOC2 | -19.66986769 | -47.73424889 | 4 |
| 2860 | RASA1 | VAV3 | -19.66961131 | -46.8633172 | 5 |
| 2861 | HDAC3 | SIN3A | -19.66775301 | -80.91675126 | 7 |
| 2862 | HMGB2 | MNAT1 | -19.66608442 | -48.91599815 | 4 |
| 2863 | SNRPE | LSM2 | -19.66608442 | -50.45112827 | 4 |
| 2864 | ACVR1B | ACVR2A | -19.66554035 | -55.27028914 | 4 |
| 2865 | SPEN | MBD2 | -19.66554035 | -44.71891888 | 4 |
| 2866 | SPEN | SAP30 | -19.66554035 | -43.10282243 | 4 |
| 2867 | CCNT1 | CDK9 | -19.66554035 | -54.08775667 | 4 |
| 2868 | TNFAIP3 | CDC25B | -19.66554035 | -42.31369664 | 4 |
| 2869 | RELA | RBBP4 | -19.65973009 | -57.28592189 | 6 |
| 2870 | PTPN12 | SHC1 | -19.64137435 | -59.49276152 | 6 |
| 2871 | NCOA1 | TDG | -19.63583448 | -49.60035165 | 5 |
| 2872 | GIT2 | PAK3 | -19.62177669 | -35.70251412 | 3 |
| 2873 | CNR1 | NGB | -19.62177669 | -33.37936603 | 3 |
| 2874 | RUVBL1 | RUVBL2 | -19.62177669 | -42.41404595 | 3 |
| 2875 | CREG1 | JARID1A | -19.62177669 | -30.13798093 | 3 |
| 2876 | GSPT1 | DIABLO | -19.62177669 | -39.32300349 | 3 |
| 2877 | KCNA4 | CRIPT | -19.62177669 | -31.63938458 | 3 |
| 2878 | NLGN3 | CRIPT | -19.62177669 | -33.20702537 | 3 |
| 2879 | TNFRSF9 | TNFRSF17 | -19.62177669 | -30.56167018 | 3 |
| 2880 | TNFRSF19 | TNFRSF17 | -19.62177669 | -30.56167018 | 3 |
| 2881 | KHDRBS2 | RBM7 | -19.62177669 | -36.50673971 | 3 |
| 2882 | TEF | HLF | -19.62177669 | -36.79994508 | 3 |
| 2883 | CITED2 | CITED1 | -19.621369 | -34.20568356 | 3 |
| 2884 | ORC5L | ORC3L | -19.621369 | -37.02446699 | 3 |
| 2885 | ARHGDIG | KTN1 | -19.621369 | -33.46682327 | 3 |
| 2886 | NGB | GPSM2 | -19.621369 | -34.36197941 | 3 |
| 2887 | TRPC4 | TRPC5 | -19.621369 | -38.28691156 | 3 |
| 2888 | BCL2L11 | PMAIP1 | -19.621369 | -33.90262653 | 3 |
| 2889 | MOAP1 | PMAIP1 | -19.621369 | -33.29649073 | 3 |
| 2890 | RASIP1 | RAPGEF6 | -19.621369 | -39.64145722 | 3 |
| 2891 | RAB8B | PFDN4 | -19.621369 | -35.43373748 | 3 |
| 2892 | RAB8B | PIGT | -19.621369 | -35.43373748 | 3 |
| 2893 | TCF8 | FOXH1 | -19.621369 | -31.13154044 | 3 |
| 2894 | MAML1 | JAG1 | -19.621369 | -38.70966231 | 3 |
| 2895 | EIF2B2 | EIF2B4 | -19.621369 | -46.66254119 | 3 |
| 2896 | CRIPT | SEMA4C | -19.621369 | -31.41037591 | 3 |
| 2897 | SF3B1 | SF3B2 | -19.621369 | -40.75054081 | 3 |
| 2898 | AP1GBP1 | M6PR | -19.621369 | -41.23539095 | 3 |
| 2899 | ALS2CR11 | MGC10854 | -19.621369 | -30.34686762 | 3 |
| 2900 | SUMO1 | ETS1 | -19.60921133 | -58.31800022 | 5 |
| 2901 | E2F4 | MYC | -19.60886155 | -47.48280281 | 5 |
| 2902 | GRM7 | MYC | -19.60886155 | -67.33226917 | 5 |
| 2903 | MCM10 | ASK | -19.60835452 | -47.15068107 | 4 |
| 2904 | MCM10 | ZNF417 | -19.60835452 | -47.20335456 | 4 |
| 2905 | FOS | TEF | -19.60483526 | -50.87031345 | 4 |
| 2906 | NRIP1 | NCOA2 | -19.60462776 | -50.97307168 | 5 |
| 2907 | RELA | NRIP1 | -19.60283573 | -70.62485979 | 7 |
| 2908 | TRAF2 | KIAA0980 | -19.59968848 | -121.6915857 | 9 |
| 2909 | PML | SP1 | -19.59147742 | -76.86477598 | 8 |
| 2910 | MTA1 | SUV39H1 | -19.590314 | -46.42366698 | 4 |
| 2911 | GLI3 | LEF1 | -19.590314 | -36.88999119 | 4 |
| 2912 | APEX1 | WRN | -19.58704936 | -42.46525142 | 4 |
| 2913 | ADRBK1 | GRK6 | -19.58704936 | -56.2902583 | 4 |
| 2914 | MUC1 | PTPRJ | -19.58704936 | -41.17621236 | 4 |
| 2915 | ERBB2IP | GLI3 | -19.58704936 | -36.88999119 | 4 |
| 2916 | CRKL | CBL | -19.58630319 | -70.64888636 | 7 |
| 2917 | SHC1 | PIK3R2 | -19.58315331 | -63.95716951 | 6 |
| 2918 | MMP14 | MMP2 | -19.58183358 | -60.50976601 | 4 |
| 2919 | SP1 | HDAC3 | -19.57944405 | -82.21283961 | 8 |
| 2920 | KIAA0408 | C1orf65 | -19.57693628 | -36.4055248 | 4 |
| 2921 | ABL1 | CRKL | -19.5729207 | -78.02468206 | 7 |
| 2922 | PTPN6 | INPP5D | -19.55042147 | -57.82242993 | 5 |
| 2923 | CCNA1 | CDK2 | -19.54127977 | -55.75785427 | 5 |
| 2924 | PTPN6 | CBLB | -19.54069811 | -60.47491733 | 6 |
| 2925 | PTPN6 | GRB10 | -19.54069811 | -64.78808284 | 6 |
| 2926 | ZBTB16 | LNX | -19.54058349 | -75.10693305 | 7 |
| 2927 | ITGB1 | ITGA6 | -19.53983202 | -60.40185415 | 5 |
| 2928 | BCL3 | TAF1 | -19.53508458 | -54.21948659 | 5 |
| 2929 | NCOR2 | HDAC9 | -19.52970175 | -58.08051118 | 5 |
| 2930 | PDGFRB | BCR | -19.52970175 | -49.13873819 | 5 |
| 2931 | TAF1 | GTF2E2 | -19.52759394 | -58.83074328 | 5 |
| 2932 | BIRC7 | BIRC2 | -19.52646618 | -35.24822133 | 3 |
| 2933 | UCP2 | CDC25B | -19.52646618 | -32.6448995 | 3 |
| 2934 | UCP3 | CDC25B | -19.52646618 | -32.6448995 | 3 |
| 2935 | ACVR2A | INHA | -19.52279596 | -39.9879798 | 3 |
| 2936 | RPA2 | RPA4 | -19.52279596 | -41.30805355 | 3 |
| 2937 | ELK1 | PTPN7 | -19.52279596 | -30.5592851 | 3 |
| 2938 | ELK1 | DUSP4 | -19.52279596 | -30.5592851 | 3 |
| 2939 | ELK1 | GMFB | -19.52279596 | -30.5592851 | 3 |
| 2940 | CCL5 | CCL3L1 | -19.52279596 | -37.73888339 | 3 |
| 2941 | GRIK2 | GDA | -19.52279596 | -31.41037591 | 3 |
| 2942 | CDK6 | DMTF1 | -19.52279596 | -36.58867815 | 3 |
| 2943 | SYT1 | SYT2 | -19.52279596 | -39.28420374 | 3 |
| 2944 | BAX | BIK | -19.52279596 | -34.15394096 | 3 |
| 2945 | RABEP1 | AP1S2 | -19.52279596 | -40.93813943 | 3 |
| 2946 | EXOC7 | SEC6L1 | -19.52279596 | -39.21677755 | 3 |
| 2947 | PTK2B | INSR | -19.51675587 | -72.58385764 | 7 |
| 2948 | XPOT | XPO1 | -19.51473456 | -49.89951331 | 4 |
| 2949 | BCAR1 | IRS1 | -19.5092168 | -60.73841899 | 6 |
| 2950 | GDF9 | BMP4 | -19.50871977 | -35.58087953 | 3 |
| 2951 | APH1A | NCSTN | -19.50871977 | -38.56491203 | 3 |
| 2952 | PSENEN | NCSTN | -19.50871977 | -38.56491203 | 3 |
| 2953 | HLA-F | HLA-B | -19.50871977 | -40.67944489 | 3 |
| 2954 | RPA3 | XPA | -19.50871977 | -41.1437505 | 3 |
| 2955 | DOCK8 | PARD6A | -19.50871977 | -32.15733703 | 3 |
| 2956 | NKRF | MEN1 | -19.50871977 | -32.43718854 | 3 |
| 2957 | SH3BP5 | DUSP16 | -19.50871977 | -37.06693842 | 3 |
| 2958 | TNFRSF4 | TNFRSF11A | -19.50871977 | -31.98570487 | 3 |
| 2959 | TNFRSF18 | TNFRSF11A | -19.50871977 | -30.56167018 | 3 |
| 2960 | TNFRSF12A | TNFRSF11A | -19.50871977 | -30.56167018 | 3 |
| 2961 | ACTR3 | ARPC5 | -19.50708889 | -43.22052181 | 3 |
| 2962 | RGS16 | EDG3 | -19.50708889 | -33.0245504 | 3 |
| 2963 | RGS16 | MTNR1A | -19.50708889 | -33.92526176 | 3 |
| 2964 | RIC8 | EDG3 | -19.50708889 | -33.0245504 | 3 |
| 2965 | RIC8 | MTNR1A | -19.50708889 | -33.92526176 | 3 |
| 2966 | LTBR | TNFRSF4 | -19.50708889 | -31.98570487 | 3 |
| 2967 | HUS1 | HUS1B | -19.50708889 | -42.33400324 | 3 |
| 2968 | HUS1 | RAD9B | -19.50708889 | -42.83752956 | 3 |
| 2969 | ID2 | HAND1 | -19.50708889 | -36.04148488 | 3 |
| 2970 | RAB11FIP2 | MYO5B | -19.50708889 | -43.93614185 | 3 |
| 2971 | EIF3S12 | EIF3S7 | -19.50708889 | -43.66680892 | 3 |
| 2972 | CUL1 | SKP1A | -19.49905171 | -57.31987772 | 4 |
| 2973 | MAPK1 | RAF1 | -19.49290289 | -97.47737657 | 8 |
| 2974 | MAP3K5 | TNFRSF11A | -19.49197688 | -42.62226986 | 4 |
| 2975 | RIPK1 | TNFRSF11A | -19.49197688 | -43.16570148 | 4 |
| 2976 | CTBP1 | ZNFN1A3 | -19.49197688 | -48.80813938 | 4 |
| 2977 | LYN | ZAP70 | -19.49134181 | -73.17176543 | 7 |
| 2978 | MCM10 | LNX | -19.48616068 | -68.28492957 | 6 |
| 2979 | PTPRC | CD4 | -19.48020232 | -76.02832037 | 6 |
| 2980 | JAK1 | KIT | -19.47962844 | -61.36008887 | 6 |
| 2981 | PTPN12 | CRK | -19.4758195 | -48.86309992 | 5 |
| 2982 | PIK3R1 | PDGFRB | -19.47199168 | -75.27020186 | 8 |
| 2983 | RELA | ESR1 | -19.46517333 | -103.7465458 | 10 |
| 2984 | FOS | TRIP4 | -19.45878835 | -48.19106002 | 5 |
| 2985 | CEBPB | CEBPA | -19.45878835 | -59.33765147 | 5 |
| 2986 | PTPN11 | SNX4 | -19.45392238 | -55.24247127 | 5 |
| 2987 | MCM10 | RIBC2 | -19.45127885 | -52.52768817 | 5 |
| 2988 | AR | RELA | -19.4500883 | -103.2518676 | 10 |
| 2989 | JAK3 | GRB2 | -19.44913738 | -90.125064 | 8 |
| 2990 | SCAP1 | FASLG | -19.44366301 | -35.06991483 | 4 |
| 2991 | CDK4 | AKAP8 | -19.43386508 | -48.17017947 | 4 |
| 2992 | EGFR | LEPR | -19.43160242 | -74.28365567 | 6 |
| 2993 | CD22 | GAB2 | -19.42614734 | -35.87390622 | 4 |
| 2994 | MYST2 | TU3A | -19.42614734 | -43.97161356 | 4 |
| 2995 | GAB1 | GRB7 | -19.42397092 | -38.89427035 | 4 |
| 2996 | GAB1 | FRS2 | -19.42397092 | -39.53782782 | 4 |
| 2997 | PAG1 | CTLA4 | -19.42397092 | -37.68159365 | 4 |
| 2998 | RBBP4 | PPARD | -19.42397092 | -41.99530216 | 4 |
| 2999 | TU3A | TCEB3B | -19.42397092 | -38.75183499 | 4 |
| 3000 | TU3A | C16orf48 | -19.42397092 | -38.22088003 | 4 |
| 3001 | PTK2B | LYN | -19.41913335 | -80.22328052 | 8 |
| 3002 | GHR | PTK2 | -19.41711383 | -59.39976816 | 6 |
| 3003 | CD82 | PTK2 | -19.41711383 | -64.36444131 | 6 |
| 3004 | PTPN1 | PTK2 | -19.41711383 | -59.68534336 | 6 |
| 3005 | JUN | ETS1 | -19.41288143 | -69.01861274 | 6 |
| 3006 | EPOR | CBLB | -19.40092566 | -47.95781872 | 5 |
| 3007 | LRP2 | SEMA4C | -19.3904422 | -43.08284901 | 4 |
| 3008 | LRP2 | ATP2B4 | -19.3904422 | -43.61261183 | 4 |
| 3009 | LRP2 | GUCY1A2 | -19.3904422 | -43.61261183 | 4 |
| 3010 | JAK2 | PECAM1 | -19.386819 | -58.5859366 | 6 |
| 3011 | RXRA | AHR | -19.386819 | -63.21366481 | 6 |
| 3012 | RXRA | PPARBP | -19.386819 | -71.44004205 | 6 |
| 3013 | NCOR1 | BCL6 | -19.36608199 | -57.50143054 | 5 |
| 3014 | RABAC1 | CUTC | -19.36545046 | -45.37105804 | 4 |
| 3015 | JUN | GTF2B | -19.35714797 | -77.21862579 | 7 |
| 3016 | ADAM15 | PECAM1 | -19.34612586 | -38.94947997 | 4 |
| 3017 | COPS2 | PPARBP | -19.34612586 | -44.31744899 | 4 |
| 3018 | CD82 | CD63 | -19.34068405 | -55.67575425 | 4 |
| 3019 | PPARBP | ERBP | -19.34068405 | -42.34725513 | 4 |
| 3020 | PPARBP | TADA3L | -19.34068405 | -37.52400684 | 4 |
| 3021 | CDKN1A | ORC2L | -19.33930797 | -64.93017945 | 5 |
| 3022 | CSF1R | ITK | -19.33070136 | -38.11074766 | 4 |
| 3023 | CD19 | CSF1R | -19.32961315 | -37.20376473 | 4 |
| 3024 | HNRPC | LOC138046 | -19.32961315 | -45.67169435 | 4 |
| 3025 | ERCC3 | CCNH | -19.32961315 | -45.7499823 | 4 |
| 3026 | VAMP2 | NAPA | -19.32961315 | -48.06224163 | 4 |
| 3027 | CFLAR | BIRC2 | -19.32961315 | -42.04055113 | 4 |
| 3028 | VAV1 | SOS1 | -19.32829243 | -56.16245865 | 6 |
| 3029 | BRCA1 | AR | -19.3217813 | -96.07577171 | 10 |
| 3030 | MET | CRKL | -19.32160906 | -48.47986567 | 5 |
| 3031 | RPA1 | WRN | -19.31905811 | -41.92563935 | 4 |
| 3032 | ITGB3 | CD47 | -19.31905811 | -47.87466606 | 4 |
| 3033 | CD2 | UNC119 | -19.31905811 | -40.89649285 | 4 |
| 3034 | HDAC1 | SUMO1 | -19.31355311 | -100.4462965 | 8 |
| 3035 | CD3E | CD5 | -19.29904081 | -41.02625647 | 4 |
| 3036 | NUP214 | NUP98 | -19.29904081 | -55.15387879 | 4 |
| 3037 | DDIT3 | ATF3 | -19.29130648 | -45.37929956 | 4 |
| 3038 | DCN | COL7A1 | -19.29130648 | -44.51203171 | 4 |
| 3039 | NRIP1 | EVI1 | -19.26667494 | -44.21029441 | 4 |
| 3040 | PLCG1 | BCAR1 | -19.26541806 | -69.47041336 | 7 |
| 3041 | DNASE1 | CFL1 | -19.26410192 | -38.47117048 | 3 |
| 3042 | TCF7L2 | CTNNA1 | -19.26410192 | -31.04396037 | 3 |
| 3043 | ASCL2 | TCF4 | -19.26410192 | -32.95758362 | 3 |
| 3044 | PPEF2 | TCF4 | -19.26410192 | -32.95758362 | 3 |
| 3045 | RBBP9 | PHB | -19.26410192 | -32.33585507 | 3 |
| 3046 | CD3E | TRAT1 | -19.26002361 | -33.0536705 | 3 |
| 3047 | EDF1 | ASCL2 | -19.26002361 | -32.95758362 | 3 |
| 3048 | EDF1 | PPEF2 | -19.26002361 | -32.95758362 | 3 |
| 3049 | EDF1 | PPEF1 | -19.26002361 | -32.95758362 | 3 |
| 3050 | TCF4 | PPEF1 | -19.26002361 | -32.95758362 | 3 |
| 3051 | C1QR1 | ANTXR2 | -19.26002361 | -37.71356558 | 3 |
| 3052 | CD5 | SLA2 | -19.26002361 | -32.30811371 | 3 |
| 3053 | TUBG1 | TUBG2 | -19.26002361 | -39.81666131 | 3 |
| 3054 | AR | VDR | -19.25851 | -67.01846514 | 6 |
| 3055 | EP300 | FOS | -19.25195496 | -98.00892389 | 8 |
| 3056 | SP1 | SRF | -19.25160017 | -76.88879189 | 7 |
| 3057 | NFKB1 | SP1 | -19.25068493 | -87.27333271 | 8 |
| 3058 | PML | PIAS3 | -19.24855484 | -46.08154761 | 5 |
| 3059 | PML | TOP2A | -19.24855484 | -46.94940364 | 5 |
| 3060 | PML | RUNX2 | -19.24855484 | -47.55141672 | 5 |
| 3061 | TCF1 | RELA | -19.24272305 | -61.91592695 | 6 |
| 3062 | FAS | FASLG | -19.23290523 | -54.23538504 | 5 |
| 3063 | NCOR2 | HDAC7A | -19.23164644 | -46.01197375 | 4 |
| 3064 | PTPN12 | RASA1 | -19.20727292 | -50.02874468 | 5 |
| 3065 | JAK1 | CBL | -19.18284789 | -70.95056773 | 7 |
| 3066 | RB1 | SIN3A | -19.1712992 | -94.93073111 | 8 |
| 3067 | FXR2 | PLEKHF2 | -19.16917111 | -70.73738897 | 6 |
| 3068 | PTPN6 | PTPN1 | -19.16831428 | -59.03763222 | 6 |
| 3069 | PTPN6 | LCP2 | -19.16831428 | -63.67182853 | 6 |
| 3070 | STX1A | VAMP2 | -19.16198765 | -69.36997189 | 5 |
| 3071 | DIPA | DPPA2 | -19.16139221 | -94.58204604 | 7 |
| 3072 | SOCS1 | WAS | -19.15935659 | -52.45483706 | 5 |
| 3073 | ADAM15 | PLCG1 | -19.15871698 | -46.65141615 | 5 |
| 3074 | KRT18 | BAD | -19.15869004 | -54.51593256 | 5 |
| 3075 | THBS1 | COL1A2 | -19.15869004 | -58.56012318 | 5 |
| 3076 | SYK | EPOR | -19.15416074 | -59.02698014 | 6 |
| 3077 | BCAR1 | CRKL | -19.14540442 | -63.373429 | 6 |
| 3078 | APP | MATN2 | -19.14106147 | -63.8657166 | 5 |
| 3079 | SERPINE1 | SERPINA5 | -19.1394185 | -51.4232986 | 4 |
| 3080 | E2F4 | SUV39H1 | -19.1394185 | -41.22363764 | 4 |
| 3081 | ABLIM1 | 76P | -19.1394185 | -44.44439625 | 4 |
| 3082 | ADRBK1 | GRK5 | -19.13669778 | -51.2297752 | 4 |
| 3083 | MUC1 | GRB7 | -19.13669778 | -39.34136781 | 4 |
| 3084 | BAD | BRAF | -19.13669778 | -43.6177529 | 4 |
| 3085 | GADD45G | NR0B2 | -19.13669778 | -41.45995194 | 4 |
| 3086 | SUV39H1 | MBD3 | -19.13669778 | -48.91920072 | 4 |
| 3087 | SOCS1 | CBL | -19.12494895 | -65.66745321 | 6 |
| 3088 | TNFRSF10A | FAS | -19.12366346 | -45.71595581 | 4 |
| 3089 | SRC | PDGFRB | -19.12298358 | -73.78603277 | 8 |
| 3090 | SRC | KRT18 | -19.12298358 | -87.90030942 | 8 |
| 3091 | PLCG1 | VAV2 | -19.12187687 | -50.28637284 | 5 |
| 3092 | KPNA2 | KPNA1 | -19.11767706 | -54.99275944 | 4 |
| 3093 | GAB3 | WAS | -19.10841592 | -33.94231501 | 4 |
| 3094 | MCM5 | MCM2 | -19.10841592 | -49.82355177 | 4 |
| 3095 | KIT | TUB | -19.1061962 | -36.94002679 | 4 |
| 3096 | CHUK | TRPC4AP | -19.1061962 | -42.19884458 | 4 |
| 3097 | TRIM37 | ZNF297B | -19.1061962 | -46.79998492 | 4 |
| 3098 | TRIM37 | TNFRSF8 | -19.1061962 | -43.16570148 | 4 |
| 3099 | MYF6 | NEUROD1 | -19.10339065 | -32.95758362 | 3 |
| 3100 | PTPRZ1 | PTPRB | -19.10339065 | -36.02716663 | 3 |
| 3101 | RGS18 | RGS12 | -19.10339065 | -33.37936603 | 3 |
| 3102 | SH3GL3 | SH3GL2 | -19.10339065 | -34.89392431 | 3 |
| 3103 | TNFRSF4 | USP7 | -19.10339065 | -31.98570487 | 3 |
| 3104 | PTPRS | PPFIA2 | -19.10339065 | -41.31543366 | 3 |
| 3105 | PTPRS | PPFIA3 | -19.10339065 | -41.31543366 | 3 |
| 3106 | PTPRD | PPFIA2 | -19.10339065 | -41.31543366 | 3 |
| 3107 | PTPRD | PPFIA3 | -19.10339065 | -41.31543366 | 3 |
| 3108 | TNFRSF18 | USP7 | -19.10339065 | -30.56167018 | 3 |
| 3109 | TNFRSF12A | USP7 | -19.10339065 | -30.56167018 | 3 |
| 3110 | RPP14 | RPP38 | -19.10339065 | -42.2111051 | 3 |
| 3111 | RPP21 | RPP38 | -19.10339065 | -42.2111051 | 3 |
| 3112 | DCTD | KCTD17 | -19.10339065 | -32.26350042 | 3 |
| 3113 | NCOR1 | NR2C1 | -19.10216204 | -40.0176805 | 4 |
| 3114 | NCOR1 | HDAC7A | -19.10216204 | -43.51801726 | 4 |
| 3115 | TGFBR3 | LTBP3 | -19.10135191 | -37.19786872 | 3 |
| 3116 | CCT5 | CCT4 | -19.10135191 | -39.17536749 | 3 |
| 3117 | BCOR | UBE2M | -19.10135191 | -38.99074889 | 3 |
| 3118 | NEUROD1 | EMP3 | -19.10135191 | -32.95758362 | 3 |
| 3119 | NEUROD1 | KCNQ2 | -19.10135191 | -32.95758362 | 3 |
| 3120 | PAK2 | NCKAP1 | -19.10135191 | -32.93894802 | 3 |
| 3121 | NCF2 | CYBA | -19.10135191 | -39.02167043 | 3 |
| 3122 | U2AF1 | U2AF1L2 | -19.10135191 | -36.24973164 | 3 |
| 3123 | RGS7 | RGS18 | -19.10135191 | -33.56135151 | 3 |
| 3124 | RGS7 | RGS5 | -19.10135191 | -34.54396489 | 3 |
| 3125 | RGS7 | RGS10 | -19.10135191 | -35.96126495 | 3 |
| 3126 | RGS19 | RGS18 | -19.10135191 | -33.37936603 | 3 |
| 3127 | RGS19 | RGS5 | -19.10135191 | -34.36197941 | 3 |
| 3128 | RGS19 | RGS10 | -19.10135191 | -35.96126495 | 3 |
| 3129 | RGS4 | RGS18 | -19.10135191 | -32.41981418 | 3 |
| 3130 | RGS4 | RGS5 | -19.10135191 | -33.40242755 | 3 |
| 3131 | RAP2A | RAP2B | -19.10135191 | -42.48815392 | 3 |
| 3132 | SH3GL2 | SH3GL1 | -19.10135191 | -36.97418252 | 3 |
| 3133 | RAP1GDS1 | SHOC2 | -19.10135191 | -37.88548686 | 3 |
| 3134 | TIFA | DCTD | -19.10135191 | -32.26350042 | 3 |
| 3135 | C1orf65 | MGC5356 | -19.10135191 | -30.30989719 | 3 |
| 3136 | KHDRBS1 | GAB3 | -19.10025154 | -36.55605453 | 4 |
| 3137 | SOS1 | SYNJ1 | -19.10025154 | -46.17368141 | 4 |
| 3138 | CALM3 | ID2 | -19.10025154 | -48.46319463 | 4 |
| 3139 | CALM3 | ID3 | -19.10025154 | -48.46319463 | 4 |
| 3140 | MAP3K7 | MAP3K7IP1 | -19.10025154 | -48.03580591 | 4 |
| 3141 | SNAP23 | VAMP8 | -19.10025154 | -48.22732138 | 4 |
| 3142 | EPOR | LCP2 | -19.09854415 | -48.9613128 | 5 |
| 3143 | POU2F1 | PPARBP | -19.09854415 | -50.70810029 | 5 |
| 3144 | TRAF2 | PCBD1 | -19.09699573 | -65.56025217 | 6 |
| 3145 | PDGFRB | IRS1 | -19.09248285 | -58.37014874 | 6 |
| 3146 | PIK3R1 | PTPN12 | -19.08952365 | -57.29558442 | 6 |
| 3147 | XPO1 | KPNB1 | -19.07797394 | -80.93639458 | 6 |
| 3148 | CCNB1 | GADD45A | -19.06801646 | -45.11394908 | 4 |
| 3149 | TUBA1 | K-ALPHA-1 | -19.06801646 | -43.63838851 | 4 |
| 3150 | TUBA1 | TUBA2 | -19.06801646 | -43.63838851 | 4 |
| 3151 | RET | PTK2 | -19.06645461 | -56.81387837 | 6 |
| 3152 | CBLB | PLCG1 | -19.06366233 | -60.21622279 | 6 |
| 3153 | CALD1 | RAB3B | -19.06243283 | -32.95758362 | 3 |
| 3154 | CALD1 | MYF5 | -19.06243283 | -32.95758362 | 3 |
| 3155 | CALD1 | KCNQ3 | -19.06243283 | -32.95758362 | 3 |
| 3156 | CALD1 | RALB | -19.06243283 | -32.95758362 | 3 |
| 3157 | KCNQ5 | MYF5 | -19.06243283 | -32.95758362 | 3 |
| 3158 | KCNQ5 | KCNQ3 | -19.06243283 | -32.95758362 | 3 |
| 3159 | KCNQ5 | RALB | -19.06243283 | -32.95758362 | 3 |
| 3160 | GRM5 | KCNQ3 | -19.06243283 | -32.95758362 | 3 |
| 3161 | GRM5 | RALB | -19.06243283 | -32.95758362 | 3 |
| 3162 | XPOT | XPO5 | -19.06243283 | -36.79195569 | 3 |
| 3163 | CNR1 | RGS14 | -19.06243283 | -33.37936603 | 3 |
| 3164 | ICAM2 | ICAM3 | -19.06243283 | -39.8883173 | 3 |
| 3165 | KCNA4 | KCNJ4 | -19.06243283 | -31.63938458 | 3 |
| 3166 | TNFRSF7 | TNFRSF8 | -19.06243283 | -31.98570487 | 3 |
| 3167 | TNFRSF9 | TNFRSF8 | -19.06243283 | -30.56167018 | 3 |
| 3168 | FLJ22746 | CATSPER1 | -19.06243283 | -31.02897204 | 3 |
| 3169 | LRPAP1 | LPL | -19.06161739 | -34.78861349 | 3 |
| 3170 | SCAP1 | SCAP2 | -19.06161739 | -32.47256961 | 3 |
| 3171 | RAB3B | KCNQ5 | -19.06161739 | -32.95758362 | 3 |
| 3172 | RAB3B | GRM5 | -19.06161739 | -32.95758362 | 3 |
| 3173 | MYF5 | GRM5 | -19.06161739 | -32.95758362 | 3 |
| 3174 | ARHGDIB | DOCK2 | -19.06161739 | -32.98483543 | 3 |
| 3175 | CDK3 | TFDP2 | -19.06161739 | -39.03305127 | 3 |
| 3176 | RGS14 | GPSM2 | -19.06161739 | -34.36197941 | 3 |
| 3177 | MAP2K1IP1 | KSR2 | -19.06161739 | -32.1881411 | 3 |
| 3178 | SYT4 | SYT7 | -19.06161739 | -34.56592075 | 3 |
| 3179 | KCNJ2 | ATP2B4 | -19.06161739 | -30.41000206 | 3 |
| 3180 | KCNJ4 | GUCY1A2 | -19.06161739 | -31.63938458 | 3 |
| 3181 | TNFRSF13B | TNFRSF19 | -19.06161739 | -31.04076854 | 3 |
| 3182 | NFIL3 | TEF | -19.06161739 | -36.79994508 | 3 |
| 3183 | NFIL3 | CREB3L1 | -19.06161739 | -39.0418361 | 3 |
| 3184 | RPP25 | POP1 | -19.06161739 | -42.00858084 | 3 |
| 3185 | POP5 | POP1 | -19.06161739 | -42.00858084 | 3 |
| 3186 | PHF5A | SF3B2 | -19.06161739 | -42.75051818 | 3 |
| 3187 | JM11 | FLJ38984 | -19.06161739 | -34.15821752 | 3 |
| 3188 | PLCG1 | PXN | -19.03940267 | -84.10310754 | 8 |
| 3189 | JAK2 | IL2RB | -19.03697655 | -58.28371298 | 6 |
| 3190 | NCOA1 | NR2F1 | -19.03583826 | -54.47686842 | 5 |
| 3191 | CCND3 | CDKN1A | -19.02465011 | -57.26971099 | 5 |
| 3192 | SHB | STAT5A | -19.02465011 | -55.20836961 | 5 |
| 3193 | PSMA6 | PSMA1 | -19.02293986 | -37.65472508 | 3 |
| 3194 | ACVR2A | BMPR1A | -19.02163576 | -57.44504086 | 4 |
| 3195 | GRIK2 | KCNJ12 | -19.02163576 | -42.38322931 | 4 |
| 3196 | SNRPE | LSM7 | -19.02163576 | -50.7483798 | 4 |
| 3197 | LSM5 | DDX20 | -19.02163576 | -51.34436323 | 4 |
| 3198 | JAK1 | IFNAR1 | -19.02145611 | -50.43268475 | 5 |
| 3199 | NTRK1 | NTRK2 | -19.02000333 | -52.86484666 | 4 |
| 3200 | GAB2 | CSF1R | -19.02000333 | -37.58047084 | 4 |
| 3201 | PAG1 | CSF1R | -19.02000333 | -36.5597343 | 4 |
| 3202 | IFNAR1 | IFNAR2 | -19.01845342 | -41.44790741 | 3 |
| 3203 | CD19 | IGSF8 | -19.01845342 | -37.60885463 | 3 |
| 3204 | ADRB2 | OPRK1 | -19.01845342 | -36.82057449 | 3 |
| 3205 | PPARGC1A | BRD8 | -19.01845342 | -33.31569294 | 3 |
| 3206 | CFTR | CLCN3 | -19.01845342 | -36.75878701 | 3 |
| 3207 | VAMP2 | STXBP6 | -19.01845342 | -34.67128127 | 3 |
| 3208 | GTF2E1 | TBPL1 | -19.01845342 | -38.87308662 | 3 |
| 3209 | PTPN11 | GNB2L1 | -19.01839891 | -89.98064943 | 8 |
| 3210 | PLCG1 | ZAP70 | -19.01715176 | -76.28268162 | 7 |
| 3211 | JAK1 | STAT5A | -19.01540975 | -64.81584424 | 6 |
| 3212 | CREBBP | UBE2I | -19.01219246 | -107.7846294 | 10 |
| 3213 | PIK3R1 | TUB | -18.99675354 | -46.89829413 | 5 |
| 3214 | ZAP70 | CD2 | -18.9830082 | -53.65467483 | 5 |
| 3215 | CASP8 | TNFRSF10B | -18.9767262 | -47.53481721 | 4 |
| 3216 | BGN | SPARC | -18.96379398 | -46.0612695 | 4 |
| 3217 | CD40 | MAP3K14 | -18.96379398 | -40.87083844 | 4 |
| 3218 | PKD1 | PTPRF | -18.96379398 | -42.22395698 | 4 |
| 3219 | CASP10 | CFLAR | -18.96379398 | -50.96166322 | 4 |
| 3220 | GTF2F1 | GTF2E1 | -18.96379398 | -48.97818142 | 4 |
| 3221 | CD28 | CD5 | -18.96324984 | -40.96171795 | 4 |
| 3222 | PPARGC1A | TRIP4 | -18.96324984 | -40.48358308 | 4 |
| 3223 | JAK2 | STAT1 | -18.95817478 | -78.83520841 | 7 |
| 3224 | IL6ST | PTK2B | -18.9533696 | -61.69756151 | 6 |
| 3225 | SOS1 | PTK2B | -18.9533696 | -57.40366291 | 6 |
| 3226 | CDH1 | CDH15 | -18.95190942 | -47.30077418 | 4 |
| 3227 | TRAF1 | TRPC4AP | -18.95190942 | -42.20318298 | 4 |
| 3228 | E2F1 | E2F3 | -18.95190942 | -52.33541791 | 4 |
| 3229 | TAF1 | GTF2A2 | -18.95190942 | -49.16289512 | 4 |
| 3230 | AR | CEBPB | -18.94492629 | -76.78631845 | 8 |
| 3231 | VAV1 | CSF1R | -18.93735577 | -46.90811647 | 5 |
| 3232 | GDF5 | IGSF1 | -18.92862968 | -35.454236 | 3 |
| 3233 | GDF5 | INHBC | -18.92862968 | -35.454236 | 3 |
| 3234 | ZFP36 | EPB41L3 | -18.92862968 | -34.08542236 | 3 |
| 3235 | ORC5L | CDC45L | -18.92862968 | -35.56917976 | 3 |
| 3236 | RNPS1 | SFRS4 | -18.92862968 | -42.16273152 | 3 |
| 3237 | FGF7 | PRELP | -18.92862968 | -35.28648602 | 3 |
| 3238 | COL13A1 | PRELP | -18.92862968 | -34.73358166 | 3 |
| 3239 | SNRPG | SNRPF | -18.92862968 | -38.49372414 | 3 |
| 3240 | EDNRB | TBXA2R | -18.92862968 | -37.94752225 | 3 |
| 3241 | HLA-DMB | HLA-DMA | -18.92862968 | -40.31690483 | 3 |
| 3242 | GGA1 | AP1G2 | -18.92862968 | -42.14438238 | 3 |
| 3243 | MKNK1 | DYRK1B | -18.92862968 | -32.01457233 | 3 |
| 3244 | RNF8 | UBOX5 | -18.92862968 | -44.8989526 | 3 |
| 3245 | DDEF2 | PIP5K1A | -18.92862968 | -39.82986872 | 3 |
| 3246 | STX6 | SYBL1 | -18.92862968 | -37.8149657 | 3 |
| 3247 | ORC4L | MCM10 | -18.92663954 | -47.15068107 | 4 |
| 3248 | MCM5 | MCM10 | -18.92663954 | -48.25667347 | 4 |
| 3249 | SMAD2 | SMURF2 | -18.92335433 | -104.0994164 | 8 |
| 3250 | PTPN6 | IRS1 | -18.92045349 | -73.63537531 | 7 |
| 3251 | GAB1 | CRKL | -18.91969148 | -48.49771556 | 5 |
| 3252 | GAB2 | CRKL | -18.91969148 | -49.19228622 | 5 |
| 3253 | PAG1 | CRKL | -18.91969148 | -46.4745321 | 5 |
| 3254 | BCR | CRKL | -18.91969148 | -50.42577582 | 5 |
| 3255 | MCM10 | ZNF426 | -18.91793028 | -41.53216928 | 4 |
| 3256 | KHDRBS1 | CSK | -18.91600849 | -48.2345928 | 5 |
| 3257 | SOS1 | WAS | -18.91600849 | -46.950188 | 5 |
| 3258 | TGFBR3 | TGFBR2 | -18.91303561 | -45.1586721 | 4 |
| 3259 | TGFB3 | TGFBR2 | -18.91303561 | -47.55858554 | 4 |
| 3260 | RIPK1 | USP7 | -18.90650455 | -43.16570148 | 4 |
| 3261 | GNAI2 | GNAQ | -18.90552997 | -85.52569343 | 6 |
| 3262 | HGS | TU3A | -18.90538532 | -61.5411066 | 5 |
| 3263 | CREBBP | TBP | -18.89655713 | -95.25776881 | 9 |
| 3264 | JUN | RXRA | -18.89655713 | -98.57887126 | 9 |
| 3265 | TEK | EGFR | -18.89203793 | -69.4381309 | 6 |
| 3266 | CDC2 | CDC25C | -18.88480213 | -52.1469799 | 5 |
| 3267 | CD22 | IRS2 | -18.87210786 | -34.99545444 | 4 |
| 3268 | E2F4 | RBBP8 | -18.87210786 | -41.63271794 | 4 |
| 3269 | DLG2 | DLGAP1 | -18.86884278 | -46.71038483 | 4 |
| 3270 | CDC25A | BRAF | -18.86884278 | -41.02732446 | 4 |
| 3271 | CD22 | LCK | -18.85978658 | -49.40359316 | 5 |
| 3272 | TNFRSF1A | TNFRSF17 | -18.8550926 | -46.87293819 | 4 |
| 3273 | TCF3 | TCF12 | -18.8550926 | -61.03301415 | 4 |
| 3274 | ZBTB16 | POU2F1 | -18.84589249 | -59.49589386 | 6 |
| 3275 | GTF2A1 | GTF2F2 | -18.83679845 | -49.05893914 | 4 |
| 3276 | TDG | PPARBP | -18.83679845 | -43.49539949 | 4 |
| 3277 | GHR | IRS4 | -18.83190049 | -35.55827247 | 4 |
| 3278 | PECAM1 | UNC119 | -18.83190049 | -39.90867119 | 4 |
| 3279 | FXR2 | KIAA1267 | -18.83183259 | -52.22015248 | 5 |
| 3280 | FXR2 | C16orf48 | -18.83183259 | -47.86976612 | 5 |
| 3281 | PSMF1 | LNX | -18.83013417 | -51.11255542 | 5 |
| 3282 | FYN | SLA | -18.82971485 | -52.93644469 | 5 |
| 3283 | SRC | PTPN12 | -18.82474597 | -58.14887723 | 6 |
| 3284 | IRS2 | PDGFRB | -18.82428288 | -44.34987821 | 5 |
| 3285 | IL2RB | KIT | -18.81863004 | -48.82307611 | 5 |
| 3286 | PTPRC | LAT | -18.81406559 | -54.06444072 | 5 |
| 3287 | PTPRC | CD2 | -18.81406559 | -54.00405047 | 5 |
| 3288 | CD4 | CD2 | -18.81406559 | -61.10586372 | 5 |
| 3289 | RBBP4 | RB1 | -18.81206089 | -63.80016327 | 6 |
| 3290 | HOXC4 | XRCC5 | -18.79979631 | -33.69370785 | 3 |
| 3291 | GRIN2A | GDA | -18.79490168 | -31.41037591 | 3 |
| 3292 | GRIN2A | ATP2B2 | -18.79490168 | -31.63938458 | 3 |
| 3293 | GRIN2A | GRIN2D | -18.79490168 | -34.2235727 | 3 |
| 3294 | YAP1 | VGLL1 | -18.79490168 | -45.05310328 | 3 |
| 3295 | IL4R | LILRB4 | -18.79490168 | -32.59033935 | 3 |
| 3296 | KCNJ12 | SCN4A | -18.79490168 | -39.93610797 | 3 |
| 3297 | KCNJ12 | SCN5A | -18.79490168 | -39.93610797 | 3 |
| 3298 | KCNJ12 | GDA | -18.79490168 | -31.41037591 | 3 |
| 3299 | KCNJ12 | ATP2B2 | -18.79490168 | -31.63938458 | 3 |
| 3300 | PML | MDM2 | -18.77925612 | -65.76027243 | 6 |
| 3301 | BCR | INSR | -18.77587541 | -50.27453505 | 5 |
| 3302 | RBBP4 | SIN3A | -18.77587541 | -55.08759231 | 5 |
| 3303 | NR3C1 | BCL3 | -18.77190799 | -61.05506271 | 6 |
| 3304 | NR3C1 | HNF4A | -18.77190799 | -65.1817642 | 6 |
| 3305 | INSR | IL4R | -18.76088698 | -51.00475388 | 5 |
| 3306 | JAK2 | CRKL | -18.74866173 | -68.42885615 | 7 |
| 3307 | SOSTDC1 | BMPR2 | -18.74685171 | -40.01615067 | 3 |
| 3308 | PDGFA | PDGFB | -18.74685171 | -40.56166186 | 3 |
| 3309 | IL6ST | PIK3R1 | -18.74528899 | -74.69302807 | 7 |
| 3310 | MARS | RARS | -18.74440506 | -43.66680892 | 3 |
| 3311 | MYOG | EMP3 | -18.74440506 | -32.95758362 | 3 |
| 3312 | MYOG | KCNQ2 | -18.74440506 | -32.95758362 | 3 |
| 3313 | PCBD1 | DCTD | -18.74440506 | -32.26350042 | 3 |
| 3314 | PCBD1 | PAICS | -18.74440506 | -31.34267453 | 3 |
| 3315 | PKP2 | PKP3 | -18.74440506 | -44.43691714 | 3 |
| 3316 | FANCC | FANCE | -18.74440506 | -41.73891727 | 3 |
| 3317 | TUBA3 | TUBA8 | -18.74440506 | -34.9062448 | 3 |
| 3318 | ENG | LTBP3 | -18.74440506 | -37.19786872 | 3 |
| 3319 | TAL1 | TAL2 | -18.74440506 | -37.74087103 | 3 |
| 3320 | TAL1 | LYL1 | -18.74440506 | -37.74087103 | 3 |
| 3321 | K-ALPHA-1 | TUBA8 | -18.74440506 | -34.9062448 | 3 |
| 3322 | TUBA2 | TUBA8 | -18.74440506 | -34.9062448 | 3 |
| 3323 | HOXA1 | OTX1 | -18.74440506 | -31.69257754 | 3 |
| 3324 | PRKD1 | SH3BP5 | -18.74440506 | -34.9697973 | 3 |
| 3325 | RBPSUH | DLL1 | -18.74440506 | -38.70966231 | 3 |
| 3326 | TNFRSF14 | TNFRSF18 | -18.74440506 | -30.56167018 | 3 |
| 3327 | TNFRSF14 | TNFRSF12A | -18.74440506 | -30.56167018 | 3 |
| 3328 | SIP1 | U2AF1L2 | -18.74440506 | -36.24973164 | 3 |
| 3329 | STX7 | VPS33A | -18.74440506 | -45.45856838 | 3 |
| 3330 | TANK | TNFRSF18 | -18.74440506 | -30.56167018 | 3 |
| 3331 | TANK | TNFRSF12A | -18.74440506 | -30.56167018 | 3 |
| 3332 | BMPR2 | NOG | -18.74440506 | -40.01615067 | 3 |
| 3333 | JAK2 | PTK2 | -18.73872905 | -78.70123752 | 8 |
| 3334 | SUV39H1 | MBD2 | -18.73286643 | -44.36954324 | 4 |
| 3335 | VDP | NAPA | -18.73286643 | -58.39395682 | 4 |
| 3336 | PDGFRB | CRKL | -18.72921823 | -57.45609614 | 6 |
| 3337 | NDP52 | MDFI | -18.72135461 | -130.9222376 | 9 |
| 3338 | PML | SAP30 | -18.71542506 | -54.18072917 | 5 |
| 3339 | HSF1 | STUB1 | -18.70597046 | -48.99156448 | 4 |
| 3340 | PTPN6 | ERBB2 | -18.70429388 | -66.87469063 | 7 |
| 3341 | PIK3R1 | ERBB2 | -18.70075323 | -76.20070408 | 8 |
| 3342 | TBP | RB1 | -18.70073496 | -110.2683697 | 9 |
| 3343 | MAP3K1 | TNFRSF1A | -18.69657536 | -47.94256762 | 5 |
| 3344 | TYK2 | PLCG1 | -18.69114776 | -61.04401418 | 6 |
| 3345 | PAK1 | WAS | -18.68416824 | -54.7233742 | 5 |
| 3346 | PIK3R1 | SOS1 | -18.68016333 | -72.12020769 | 7 |
| 3347 | PIK3R1 | WAS | -18.68016333 | -69.15010921 | 7 |
| 3348 | SCAP1 | BCAR1 | -18.67264015 | -36.02910605 | 4 |
| 3349 | ASK | MCM7 | -18.67264015 | -49.64250817 | 4 |
| 3350 | AR | TBP | -18.66990667 | -93.83693648 | 9 |
| 3351 | CASK | SDCBP | -18.6611319 | -68.38514606 | 5 |
| 3352 | BCAR1 | TRPV4 | -18.66011632 | -39.90867119 | 4 |
| 3353 | CD3E | PAG1 | -18.65486463 | -40.3117995 | 4 |
| 3354 | FBF1 | TU3A | -18.65486463 | -37.19880782 | 4 |
| 3355 | GGA2 | AP1G1 | -18.65486463 | -56.95668809 | 4 |
| 3356 | GAB2 | CD5 | -18.65377627 | -40.80583154 | 4 |
| 3357 | PAG1 | CD5 | -18.65377627 | -41.05796263 | 4 |
| 3358 | PTPN11 | RAPGEF1 | -18.651733 | -50.6680452 | 5 |
| 3359 | PTPN11 | GRB14 | -18.651733 | -55.07541719 | 5 |
| 3360 | NIF3L1 | FLJ10204 | -18.65071909 | -66.20839959 | 5 |
| 3361 | DIPA | ZBTB8 | -18.64751109 | -81.09421489 | 7 |
| 3362 | NDP52 | LDOC1 | -18.64230985 | -71.93802813 | 6 |
| 3363 | CBFA2T3 | SIN3A | -18.63924048 | -44.05048353 | 4 |
| 3364 | CDC42 | RAC2 | -18.6358527 | -71.63319226 | 5 |
| 3365 | PML | ARID4A | -18.63145678 | -38.26868238 | 4 |
| 3366 | GRB2 | BCAR1 | -18.62767231 | -100.3120673 | 9 |
| 3367 | DLG4 | LIN7B | -18.62654386 | -63.77675651 | 5 |
| 3368 | GRB7 | GRB10 | -18.62174692 | -42.44643876 | 4 |
| 3369 | NR2F1 | NCOA2 | -18.62174692 | -41.01334591 | 4 |
| 3370 | CDKN1B | NPDC1 | -18.61793739 | -47.49699303 | 4 |
| 3371 | NCOA2 | NR0B2 | -18.61793739 | -39.70634424 | 4 |
| 3372 | KIAA0408 | C16orf48 | -18.61793739 | -36.76060986 | 4 |
| 3373 | BRCA1 | HDAC1 | -18.61621249 | -107.5869685 | 10 |
| 3374 | GNAI3 | GNAZ | -18.60902979 | -55.14405705 | 4 |
| 3375 | ATF2 | ATF1 | -18.60902979 | -44.65590034 | 4 |
| 3376 | PTK2B | SYN1 | -18.60806853 | -46.22530209 | 5 |
| 3377 | EGFR | IRS1 | -18.60096956 | -82.94318966 | 8 |
| 3378 | REM1 | RIN1 | -18.59270116 | -31.34084324 | 3 |
| 3379 | CNR1 | RGS16 | -18.59270116 | -33.37936603 | 3 |
| 3380 | CNR1 | RIC8 | -18.59270116 | -33.37936603 | 3 |
| 3381 | AIP | DAP3 | -18.59270116 | -33.1722609 | 3 |
| 3382 | GEMIN4 | SNRPD3 | -18.59270116 | -39.09985994 | 3 |
| 3383 | GEMIN4 | SNRPD1 | -18.59270116 | -39.09985994 | 3 |
| 3384 | DUSP1 | DUSP16 | -18.59270116 | -31.01127023 | 3 |
| 3385 | TNFRSF7 | TNFRSF11A | -18.59270116 | -31.98570487 | 3 |
| 3386 | TFDP2 | RYBP | -18.59270116 | -42.25989527 | 3 |
| 3387 | MSC | ID2 | -18.59270116 | -37.89135198 | 3 |
| 3388 | ARCN1 | TAPBP | -18.59270116 | -38.9746968 | 3 |
| 3389 | TNFRSF9 | TNFRSF11A | -18.59270116 | -30.56167018 | 3 |
| 3390 | UTP14A | ZNF426 | -18.59270116 | -31.40292029 | 3 |
| 3391 | CKS1B | CCNE1 | -18.59215694 | -33.49965794 | 3 |
| 3392 | RBBP9 | SUV39H1 | -18.59215694 | -32.33585507 | 3 |
| 3393 | PARC | UBE3A | -18.59215694 | -35.71598342 | 3 |
| 3394 | BIRC7 | BIRC4 | -18.59215694 | -35.24822133 | 3 |
| 3395 | AFAP | PDLIM5 | -18.59147792 | -37.0717117 | 3 |
| 3396 | HLA-B | HLA-E | -18.59147792 | -39.91730484 | 3 |
| 3397 | OPRM1 | CNR1 | -18.59147792 | -34.1155306 | 3 |
| 3398 | SERPINB13 | SERPINA1 | -18.59147792 | -40.94770888 | 3 |
| 3399 | ORC4L | ORC3L | -18.59147792 | -37.6486213 | 3 |
| 3400 | BAK1 | BID | -18.59147792 | -34.15394096 | 3 |
| 3401 | ICAM1 | ICAM2 | -18.59147792 | -36.47607008 | 3 |
| 3402 | RGS16 | GPSM2 | -18.59147792 | -34.36197941 | 3 |
| 3403 | RIC8 | GPSM2 | -18.59147792 | -34.36197941 | 3 |
| 3404 | LTBR | TNFRSF7 | -18.59147792 | -31.98570487 | 3 |
| 3405 | LTBR | TNFRSF19 | -18.59147792 | -31.98570487 | 3 |
| 3406 | TAF11 | TAF7 | -18.59147792 | -38.85039908 | 3 |
| 3407 | APEX2 | UTP14A | -18.59147792 | -31.2693889 | 3 |
| 3408 | APEX2 | FLJ22746 | -18.59147792 | -30.53029651 | 3 |
| 3409 | APEX2 | RHPN1 | -18.59147792 | -32.56335018 | 3 |
| 3410 | RIN1 | KIF1C | -18.59147792 | -31.34084324 | 3 |
| 3411 | CCL3 | CCL14 | -18.59147792 | -37.44163187 | 3 |
| 3412 | SCNN1B | SCNN1A | -18.59147792 | -38.94570926 | 3 |
| 3413 | DEDD | DEDD2 | -18.59147792 | -36.14738318 | 3 |
| 3414 | TRIM39 | RNF126 | -18.59147792 | -42.95304245 | 3 |
| 3415 | PAG1 | SHC1 | -18.59064291 | -61.25399945 | 6 |
| 3416 | BAD | BIK | -18.58685407 | -34.15394096 | 3 |
| 3417 | BAD | UCP2 | -18.58685407 | -32.6448995 | 3 |
| 3418 | BAD | UCP3 | -18.58685407 | -32.6448995 | 3 |
| 3419 | SUV39H1 | HR | -18.58685407 | -31.17670786 | 3 |
| 3420 | ERBB4 | GDA | -18.58685407 | -31.41037591 | 3 |
| 3421 | ERBB4 | ATP2B2 | -18.58685407 | -31.63938458 | 3 |
| 3422 | PTK2B | CBLC | -18.54588896 | -36.64239393 | 4 |
| 3423 | PIK3R1 | CSF2RB | -18.54154147 | -65.06308427 | 6 |
| 3424 | TYK2 | BCAR1 | -18.54145063 | -49.84196829 | 5 |
| 3425 | VIM | HOOK2 | -18.53492021 | -62.62836531 | 5 |
| 3426 | BCAR1 | PECAM1 | -18.53395795 | -48.70604 | 5 |
| 3427 | VIM | USHBP1 | -18.52937516 | -76.60335405 | 6 |
| 3428 | CSF1R | CBL | -18.52888899 | -47.6596882 | 5 |
| 3429 | TNFRSF10A | FADD | -18.52267245 | -47.91714079 | 4 |
| 3430 | TNFRSF10A | TRADD | -18.52267245 | -47.24201211 | 4 |
| 3431 | CCND1 | PELP1 | -18.52182982 | -36.20221033 | 4 |
| 3432 | YWHAH | PELP1 | -18.52182982 | -39.11135284 | 4 |
| 3433 | TNFRSF1A | CASP8 | -18.51863218 | -71.36144331 | 6 |
| 3434 | STAT3 | SRC | -18.51729715 | -107.3878024 | 9 |
| 3435 | PTK2B | EGFR | -18.51729715 | -86.34434485 | 9 |
| 3436 | KHDRBS1 | ADAM15 | -18.51505184 | -35.06991483 | 4 |
| 3437 | SOS1 | EGF | -18.51505184 | -39.34136781 | 4 |
| 3438 | FOS | HLF | -18.51085514 | -48.56772835 | 4 |
| 3439 | FOS | NFE2L1 | -18.51085514 | -45.78109494 | 4 |
| 3440 | ERBB2 | FYN | -18.50338891 | -81.30210607 | 8 |
| 3441 | NCOA1 | CDC25B | -18.50298219 | -49.69936042 | 5 |
| 3442 | CDKN1B | CDK2 | -18.50186272 | -64.63667852 | 5 |
| 3443 | SKP2 | CDK2 | -18.50186272 | -63.47599381 | 5 |
| 3444 | TP53 | SREBF2 | -18.49739463 | -59.5582422 | 6 |
| 3445 | RARA | HNF4A | -18.49261108 | -63.27450645 | 5 |
| 3446 | PTPRC | SHB | -18.49232593 | -52.68861766 | 5 |
| 3447 | BCAR1 | RASA1 | -18.48017866 | -61.9663631 | 6 |
| 3448 | TLN1 | PTK2 | -18.47798089 | -56.86380207 | 5 |
| 3449 | CD22 | PTK2 | -18.47798089 | -45.45105132 | 5 |
| 3450 | BCL3 | REL | -18.4651477 | -44.38159535 | 4 |
| 3451 | CDC25A | CDC25B | -18.4651477 | -45.74768384 | 4 |
| 3452 | FASLG | WAS | -18.46279954 | -44.25148326 | 5 |
| 3453 | JAK2 | CD22 | -18.4472913 | -44.8322868 | 5 |
| 3454 | JAK2 | CTLA4 | -18.4472913 | -50.6243622 | 5 |
| 3455 | JAK2 | PRLR | -18.4472913 | -59.03248293 | 5 |
| 3456 | TBP | NR2F1 | -18.4472913 | -52.27882718 | 5 |
| 3457 | CSK | EGFR | -18.44278634 | -71.21070847 | 7 |
| 3458 | HRAS | RAF1 | -18.43479856 | -83.31543375 | 7 |
| 3459 | MYF6 | GRM7 | -18.42853401 | -32.95758362 | 3 |
| 3460 | GRK1 | GRK5 | -18.42853401 | -39.14532232 | 3 |
| 3461 | IGFBP4 | IGFBP5 | -18.42853401 | -38.99164636 | 3 |
| 3462 | UBE2M | SKIL | -18.42853401 | -38.99074889 | 3 |
| 3463 | RAB11FIP4 | RAB11A | -18.42853401 | -45.12209615 | 3 |
| 3464 | MGC2560 | DTX2 | -18.42853401 | -30.75502354 | 3 |
| 3465 | LTBR | TRIM37 | -18.42652541 | -44.74388685 | 4 |
| 3466 | FANCA | FANCD2 | -18.42567939 | -37.92822252 | 3 |
| 3467 | CAST | PTPRS | -18.42567939 | -39.52367419 | 3 |
| 3468 | CAST | PTPRD | -18.42567939 | -39.52367419 | 3 |
| 3469 | CAST | PPFIBP1 | -18.42567939 | -39.52367419 | 3 |
| 3470 | AKAP5 | AKAP12 | -18.42567939 | -34.18787373 | 3 |
| 3471 | GRM7 | EMP3 | -18.42567939 | -32.95758362 | 3 |
| 3472 | GRM7 | KCNQ2 | -18.42567939 | -32.95758362 | 3 |
| 3473 | PPP5C | UBE2M | -18.42567939 | -38.99074889 | 3 |
| 3474 | MAP3K4 | GADD45GIP1 | -18.42567939 | -38.56086344 | 3 |
| 3475 | NFATC2 | NFATC4 | -18.42567939 | -30.94161091 | 3 |
| 3476 | DLGAP1 | GRIN3A | -18.42567939 | -31.70967851 | 3 |
| 3477 | TAF15 | POLR2F | -18.42567939 | -45.74625046 | 3 |
| 3478 | CRY1 | CRY2 | -18.42567939 | -42.12636387 | 3 |
| 3479 | C16orf48 | MGC5356 | -18.42567939 | -30.30989719 | 3 |
| 3480 | NXF2 | XPO1 | -18.42034745 | -52.36359049 | 4 |
| 3481 | EPOR | GAB3 | -18.41618105 | -35.52378629 | 4 |
| 3482 | ITK | FYN | -18.40792966 | -65.63154387 | 6 |
| 3483 | GNAZ | GNAO1 | -18.4039808 | -54.15365834 | 4 |
| 3484 | SIT1 | PTPRC | -18.40177697 | -37.33190192 | 4 |
| 3485 | RBBP9 | RBBP8 | -18.39800093 | -32.33585507 | 3 |
| 3486 | MFAP5 | ELN | -18.39800093 | -42.16273152 | 3 |
| 3487 | TGFBR2 | ENG | -18.39799359 | -45.1586721 | 4 |
| 3488 | RUNX1T1 | CBFA2T2 | -18.39799359 | -46.77455114 | 4 |
| 3489 | RIPK1 | TANK | -18.39799359 | -42.23414328 | 4 |
| 3490 | JAK2 | SOCS1 | -18.39571837 | -62.94170343 | 6 |
| 3491 | RXRA | ESR2 | -18.39571837 | -72.26205176 | 6 |
| 3492 | ITK | NCK1 | -18.39280547 | -59.26963343 | 5 |
| 3493 | ZNF250 | LNX | -18.39280547 | -53.87568806 | 5 |
| 3494 | GRIN2B | GRIN2D | -18.39228975 | -34.2235727 | 3 |
| 3495 | CDK2 | CDK3 | -18.38816226 | -49.78210301 | 4 |
| 3496 | NCOR2 | CEBPB | -18.38754838 | -59.70348188 | 6 |
| 3497 | TRAF2 | KRT15 | -18.38678278 | -125.3149849 | 10 |
| 3498 | CD22 | PECAM1 | -18.38631175 | -37.70973892 | 4 |
| 3499 | SERPINE1 | KNG1 | -18.38631175 | -47.57539724 | 4 |
| 3500 | NR2F1 | PPARBP | -18.38631175 | -43.09360411 | 4 |
| 3501 | MYST2 | FLJ32855 | -18.38631175 | -45.50467849 | 4 |
| 3502 | GHR | CD22 | -18.38195772 | -35.16222927 | 4 |
| 3503 | BTK | TEC | -18.38195772 | -44.28699658 | 4 |
| 3504 | FLJ32855 | TCEB3B | -18.38195772 | -40.1245237 | 4 |
| 3505 | FLJ32855 | ZNF638 | -18.38195772 | -39.93668103 | 4 |
| 3506 | EP300 | ESR1 | -18.37817988 | -111.5670162 | 10 |
| 3507 | RELA | YY1 | -18.37519447 | -60.67517036 | 6 |
| 3508 | MYOZ2 | PDLIM1 | -18.36942181 | -36.07694493 | 3 |
| 3509 | MYOZ2 | MYOZ1 | -18.36942181 | -36.07694493 | 3 |
| 3510 | ARHGDIG | ARHGDIB | -18.36942181 | -35.95172992 | 3 |
| 3511 | MAFF | MAFK | -18.36942181 | -41.9773283 | 3 |
| 3512 | SIGIRR | TRIAD3 | -18.36942181 | -47.76115348 | 3 |
| 3513 | BNIP3 | BNIP3L | -18.36942181 | -37.48614547 | 3 |
| 3514 | E2F6 | E2F3 | -18.36942181 | -42.3776783 | 3 |
| 3515 | SF3B1 | PHF5A | -18.36942181 | -45.64088994 | 3 |
| 3516 | TNFRSF17 | TNFRSF8 | -18.36942181 | -30.56167018 | 3 |
| 3517 | ASK | CDC45L | -18.36901406 | -35.56917976 | 3 |
| 3518 | ARHGDIB | GDI1 | -18.36901406 | -38.97259816 | 3 |
| 3519 | EVI1 | TCF8 | -18.36901406 | -32.43082343 | 3 |
| 3520 | MED25 | MED6 | -18.36901406 | -37.51062474 | 3 |
| 3521 | KCNJ4 | CRIPT | -18.36901406 | -31.63938458 | 3 |
| 3522 | NFIL3 | HLF | -18.36901406 | -36.79994508 | 3 |
| 3523 | RPP25 | POP4 | -18.36901406 | -42.41404595 | 3 |
| 3524 | POP5 | POP4 | -18.36901406 | -42.41404595 | 3 |
| 3525 | PNKP | ZNF655 | -18.36901406 | -30.13117299 | 3 |
| 3526 | FOSL2 | HLF | -18.36901406 | -38.68701473 | 3 |
| 3527 | FOSL2 | NFE2L1 | -18.36901406 | -34.01331167 | 3 |
| 3528 | CD40 | RIPK2 | -18.36840829 | -42.22076515 | 4 |
| 3529 | FGA | SERPINA5 | -18.36840829 | -51.76195504 | 4 |
| 3530 | TNFAIP3 | RIPK2 | -18.36840829 | -41.28920695 | 4 |
| 3531 | MAP3K1 | MAP3K11 | -18.36677564 | -48.6381666 | 4 |
| 3532 | BAD | TNFAIP3 | -18.36677564 | -42.31369664 | 4 |
| 3533 | SUV39H1 | PHB | -18.36677564 | -41.22363764 | 4 |
| 3534 | TRAF1 | RIPK1 | -18.34972185 | -55.64346231 | 5 |
| 3535 | TBP | FOS | -18.34876511 | -76.09437107 | 7 |
| 3536 | CDC7 | MCM10 | -18.34103242 | -50.27157649 | 4 |
| 3537 | CRKL | IRS1 | -18.33932087 | -59.54351857 | 6 |
| 3538 | NIF3L1 | KIAA0408 | -18.32911609 | -46.53328596 | 5 |
| 3539 | IFNAR1 | GAB2 | -18.31934617 | -38.52550728 | 4 |
| 3540 | PFN1 | GSN | -18.31934617 | -43.8046033 | 4 |
| 3541 | CD19 | PAG1 | -18.31934617 | -39.03053223 | 4 |
| 3542 | LSM3 | DDX20 | -18.31934617 | -50.72020892 | 4 |
| 3543 | CSF2RB | IL4R | -18.31934617 | -38.2799963 | 4 |
| 3544 | PSMA1 | TU3A | -18.31934617 | -38.89880997 | 4 |
| 3545 | GRB2 | GAB2 | -18.31893378 | -70.69271409 | 7 |
| 3546 | GRB2 | PAG1 | -18.31893378 | -69.10547062 | 7 |
| 3547 | GRB2 | IL4R | -18.31893378 | -75.09565966 | 7 |
| 3548 | GAB1 | CD28 | -18.31880195 | -37.44818219 | 4 |
| 3549 | GAB2 | CD28 | -18.31880195 | -39.24147894 | 4 |
| 3550 | GAB2 | CSF2RB | -18.31880195 | -39.9831296 | 4 |
| 3551 | PAG1 | CSF2RB | -18.31880195 | -37.68740559 | 4 |
| 3552 | SNRP70 | SFRS2 | -18.31880195 | -56.93421524 | 4 |
| 3553 | HDAC1 | ZNFN1A1 | -18.31831125 | -74.20062836 | 6 |
| 3554 | JAK2 | LYN | -18.31314384 | -90.45911091 | 8 |
| 3555 | TBP | SP1 | -18.31314384 | -88.96371937 | 8 |
| 3556 | JUN | ZBTB16 | -18.30609865 | -85.17674831 | 8 |
| 3557 | CD22 | PTPN6 | -18.29766551 | -47.53327428 | 5 |
| 3558 | LRP2 | CRIPT | -18.29673522 | -43.61261183 | 4 |
| 3559 | PTPN11 | PXN | -18.29618066 | -87.10868006 | 8 |
| 3560 | EPOR | VAV1 | -18.28991492 | -56.32130104 | 6 |
| 3561 | SRC | ITK | -18.2773146 | -61.66100695 | 6 |
| 3562 | SRC | CBL | -18.27392439 | -92.43978259 | 9 |
| 3563 | TRAF2 | TSC22D4 | -18.26720499 | -92.93477076 | 8 |
| 3564 | HSPCA | TRA1 | -18.26560756 | -60.55303836 | 5 |
| 3565 | MLLT4 | RASSF5 | -18.26203066 | -53.30636048 | 4 |
| 3566 | RELA | CCND1 | -18.26029533 | -71.67288752 | 7 |
| 3567 | PHB | HDAC3 | -18.25964465 | -50.96024174 | 5 |
| 3568 | SPEN | HDAC3 | -18.25964465 | -56.21038005 | 5 |
| 3569 | IL2RB | IGF1R | -18.25649828 | -50.25040463 | 5 |
| 3570 | JUN | RB1 | -18.25126181 | -106.4702598 | 10 |
| 3571 | PML | SKIIP | -18.23578476 | -52.30250356 | 5 |
| 3572 | PML | PHB | -18.23578476 | -47.31066205 | 5 |
| 3573 | PML | SPEN | -18.23578476 | -51.68971011 | 5 |
| 3574 | ELK1 | MAP2K1 | -18.21764413 | -42.64373799 | 4 |
| 3575 | SSSCA1 | EFCBP2 | -18.21764413 | -48.18774973 | 4 |
| 3576 | ANTXR2 | FBLN2 | -18.21567937 | -37.71356558 | 3 |
| 3577 | TRIP3 | NCOA2 | -18.21567937 | -33.65006212 | 3 |
| 3578 | CDKN1B | CDK6 | -18.21437861 | -47.6972158 | 4 |
| 3579 | SHB | SYN1 | -18.21437861 | -35.76970521 | 4 |
| 3580 | SHB | MAP4K1 | -18.21437861 | -41.64580567 | 4 |
| 3581 | PDGFRA | LEPR | -18.21437861 | -48.84869899 | 4 |
| 3582 | CD22 | LYN | -18.20993392 | -51.26032135 | 5 |
| 3583 | CDKN1B | DMTF1 | -18.20955984 | -36.58867815 | 3 |
| 3584 | GTF2H1 | GTF2H2 | -18.20955984 | -39.91808946 | 3 |
| 3585 | GTF2H1 | GTF2H4 | -18.20955984 | -38.73943446 | 3 |
| 3586 | MAP2K1 | PTPN7 | -18.20955984 | -30.5592851 | 3 |
| 3587 | MAP2K1 | DUSP4 | -18.20955984 | -30.5592851 | 3 |
| 3588 | MAP2K1 | GMFB | -18.20955984 | -30.5592851 | 3 |
| 3589 | PDGFRA | PDAP1 | -18.20955984 | -39.53967453 | 3 |
| 3590 | NCOA2 | BRD8 | -18.20955984 | -33.31569294 | 3 |
| 3591 | TNK2 | SOCS1 | -18.20412776 | -35.45411445 | 4 |
| 3592 | NR4A1 | THRB | -18.20412776 | -47.69804252 | 4 |
| 3593 | GFAP | VIM | -18.20378786 | -64.13277334 | 5 |
| 3594 | EXOC8 | HGS | -18.20378786 | -62.09568248 | 5 |
| 3595 | GHR | PDGFRB | -18.19948372 | -46.89656222 | 5 |
| 3596 | TIF1 | TDG | -18.19759582 | -41.49763598 | 4 |
| 3597 | CASP3 | DIABLO | -18.1938081 | -52.85437536 | 4 |
| 3598 | ITGB1 | TSPAN4 | -18.1938081 | -52.87239387 | 4 |
| 3599 | ESR1 | SP1 | -18.19363474 | -93.72184364 | 9 |
| 3600 | CRKL | PLCG2 | -18.19084243 | -58.42562483 | 5 |
| 3601 | EPRS | QARS | -18.18750805 | -38.33590336 | 3 |
| 3602 | CALD1 | NEUROD1 | -18.18750805 | -32.95758362 | 3 |
| 3603 | GIT2 | ARHGEF7 | -18.18750805 | -36.57798286 | 3 |
| 3604 | HLA-E | HLA-G | -18.18750805 | -39.91730484 | 3 |
| 3605 | KCNQ5 | NEUROD1 | -18.18750805 | -32.95758362 | 3 |
| 3606 | CNR1 | RGS19 | -18.18750805 | -33.37936603 | 3 |
| 3607 | CNR1 | RGS12 | -18.18750805 | -33.37936603 | 3 |
| 3608 | DEF6 | NCF2 | -18.18750805 | -32.66086335 | 3 |
| 3609 | SEC8L1 | LIN7A | -18.18750805 | -33.20702537 | 3 |
| 3610 | KIF13A | AP1M2 | -18.18750805 | -37.85361165 | 3 |
| 3611 | NRBF2 | COPS2 | -18.18750805 | -32.2329961 | 3 |
| 3612 | CGI-63 | COPS2 | -18.18750805 | -32.2329961 | 3 |
| 3613 | TNFRSF7 | USP7 | -18.18750805 | -31.98570487 | 3 |
| 3614 | NLGN3 | LIN7A | -18.18750805 | -33.20702537 | 3 |
| 3615 | TNFRSF9 | USP7 | -18.18750805 | -30.56167018 | 3 |
| 3616 | TES | ZYX | -18.18750805 | -37.40608088 | 3 |
| 3617 | C10orf10 | MGC3162 | -18.18750805 | -30.23382687 | 3 |
| 3618 | MMP14 | MMP3 | -18.18587695 | -46.43939764 | 3 |
| 3619 | TTRAP | TRIP | -18.18587695 | -35.50616568 | 3 |
| 3620 | TTRAP | TNFRSF19 | -18.18587695 | -31.04076854 | 3 |
| 3621 | NEUROD1 | GRM5 | -18.18587695 | -32.95758362 | 3 |
| 3622 | BIRC5 | GORASP1 | -18.18587695 | -33.67065862 | 3 |
| 3623 | NCF2 | ARHGDIA | -18.18587695 | -32.66086335 | 3 |
| 3624 | SERPINF2 | SERPINA1 | -18.18587695 | -42.48815392 | 3 |
| 3625 | U2AF1 | SFRS11 | -18.18587695 | -36.24973164 | 3 |
| 3626 | RGS19 | GPSM2 | -18.18587695 | -34.36197941 | 3 |
| 3627 | CD63 | PTGFRN | -18.18587695 | -37.60885463 | 3 |
| 3628 | CTNND2 | PKP4 | -18.18587695 | -39.38322235 | 3 |
| 3629 | TNFSF11 | DUSP1 | -18.18587695 | -30.7728592 | 3 |
| 3630 | TNFSF11 | SNCG | -18.18587695 | -30.7728592 | 3 |
| 3631 | RPH3AL | RAB3IP | -18.18587695 | -41.85443016 | 3 |
| 3632 | PER3 | PER2 | -18.18587695 | -42.21337525 | 3 |
| 3633 | TAF5 | TAF7 | -18.18587695 | -38.3579226 | 3 |
| 3634 | RENT1 | DCP1A | -18.18587695 | -39.8744144 | 3 |
| 3635 | LIN7A | GUCY1A2 | -18.18587695 | -33.20702537 | 3 |
| 3636 | PTPN6 | SOCS1 | -18.17803659 | -59.58822153 | 6 |
| 3637 | DIPA | EFCBP2 | -18.17178404 | -85.8030983 | 7 |
| 3638 | PCTK1 | RAF1 | -18.17002435 | -43.8559258 | 4 |
| 3639 | REM1 | RAF1 | -18.17002435 | -42.31369664 | 4 |
| 3640 | ADAM15 | FASLG | -18.16736065 | -35.06991483 | 4 |
| 3641 | RET | CD22 | -18.15922332 | -34.99545444 | 4 |
| 3642 | HIF1A | EGR1 | -18.15922332 | -35.20320798 | 4 |
| 3643 | ATF2 | RUNX2 | -18.15922332 | -38.89076204 | 4 |
| 3644 | PPARG | PPARD | -18.15922332 | -50.15163021 | 4 |
| 3645 | RIBC2 | ZNF638 | -18.15922332 | -41.60778567 | 4 |
| 3646 | BRCA1 | CCND1 | -18.15883575 | -70.5835714 | 7 |
| 3647 | CLTC | HIP1 | -18.1586502 | -61.06938179 | 4 |
| 3648 | CD3E | PTK2B | -18.15310837 | -49.10916831 | 5 |
| 3649 | SUMO2 | SUMO1 | -18.14822088 | -46.14094521 | 4 |
| 3650 | STAT3 | PTPN11 | -18.1482029 | -91.94788289 | 8 |
| 3651 | RAF1 | KIF1C | -18.14330305 | -43.6682423 | 4 |
| 3652 | DIPA | TSC22D4 | -18.14169373 | -95.95574565 | 8 |
| 3653 | FIBP | FGF1 | -18.14098798 | -37.3478408 | 3 |
| 3654 | NDP52 | TSC22D4 | -18.1398395 | -71.02651067 | 6 |
| 3655 | BMP2 | GDF9 | -18.13772534 | -35.58087953 | 3 |
| 3656 | CCL5 | CCL21 | -18.13772534 | -37.97139469 | 3 |
| 3657 | CCL5 | CCL11 | -18.13772534 | -36.89158553 | 3 |
| 3658 | CCL5 | CCL13 | -18.13772534 | -36.89158553 | 3 |
| 3659 | HLA-A | HLA-F | -18.13772534 | -40.67944489 | 3 |
| 3660 | GRIK2 | GRIA4 | -18.13772534 | -37.76787093 | 3 |
| 3661 | PDCD6 | UBE2M | -18.13772534 | -38.99074889 | 3 |
| 3662 | CHEK2 | CLSPN | -18.13772534 | -36.30773477 | 3 |
| 3663 | REL | NKRF | -18.13772534 | -32.43718854 | 3 |
| 3664 | RDX | UBE2M | -18.13772534 | -38.99074889 | 3 |
| 3665 | CDC25B | NFATC4 | -18.13772534 | -30.94161091 | 3 |
| 3666 | CASP8 | CASP1 | -18.13351498 | -53.96825512 | 4 |
| 3667 | CASP8 | TRPC4AP | -18.13351498 | -46.27586227 | 4 |
| 3668 | MUC1 | SHC1 | -18.13049629 | -59.23223073 | 6 |
| 3669 | STAT3 | TRIP4 | -18.1285651 | -47.52323065 | 5 |
| 3670 | PTK2B | PIK3R2 | -18.1285651 | -46.12298455 | 5 |
| 3671 | PTPN11 | PDGFRB | -18.12495208 | -64.76050158 | 7 |
| 3672 | DLG1 | CASK | -18.10590739 | -80.80013942 | 6 |
| 3673 | RAF1 | CDC25B | -18.10354428 | -51.34525884 | 5 |
| 3674 | GTF2F1 | GTF2E2 | -18.10137022 | -50.30731737 | 4 |
| 3675 | TNNT1 | MBIP | -18.10137022 | -41.32788893 | 4 |
| 3676 | PTK2 | WAS | -18.1002728 | -56.5876415 | 6 |
| 3677 | IRS2 | EPHA2 | -18.0991932 | -34.81210934 | 4 |
| 3678 | EXOC8 | ABI2 | -18.0991932 | -47.97043926 | 4 |
| 3679 | SAA1 | FN1 | -18.09465364 | -50.17449604 | 4 |
| 3680 | ZBTB16 | HEY2 | -18.09345164 | -39.73101589 | 4 |
| 3681 | JAK3 | ZAP70 | -18.08265554 | -54.2938187 | 5 |
| 3682 | TP53 | GTF2E2 | -18.06606325 | -86.04858588 | 7 |
| 3683 | NEDD9 | SHC1 | -18.06169381 | -75.80357593 | 7 |
| 3684 | CD3E | CBL | -18.0488424 | -49.10916831 | 5 |
| 3685 | PTPN12 | ABL1 | -18.0488424 | -54.15264327 | 5 |
| 3686 | PIK3R2 | CBL | -18.0488424 | -48.98686751 | 5 |
| 3687 | KIT | WAS | -18.04798144 | -49.81380246 | 5 |
| 3688 | CHUK | TRADD | -18.04798144 | -56.81699428 | 5 |
| 3689 | TRIP3 | PPARBP | -18.04382912 | -33.65006212 | 3 |
| 3690 | NDP52 | KRTAP4-12 | -18.04090078 | -104.9195819 | 8 |
| 3691 | PTPN1 | PIK3C2B | -18.03730117 | -31.73593382 | 3 |
| 3692 | KNG1 | TFPI2 | -18.03730117 | -40.63828682 | 3 |
| 3693 | PECAM1 | LILRB4 | -18.03730117 | -32.59033935 | 3 |
| 3694 | SNAP25 | CPLX1 | -18.03730117 | -36.1428685 | 3 |
| 3695 | PPARBP | BRD8 | -18.03730117 | -33.31569294 | 3 |
| 3696 | PSMA1 | 76P | -18.03302612 | -42.30093285 | 4 |
| 3697 | THAP1 | DPPA2 | -18.03302612 | -47.5341742 | 4 |
| 3698 | STAT3 | RELA | -18.03247974 | -79.04170802 | 8 |
| 3699 | GADD45G | PPARGC1A | -18.03193761 | -42.16927198 | 4 |
| 3700 | PIK3R1 | WASF1 | -18.02768591 | -48.59141692 | 5 |
| 3701 | MYF5 | TCF3 | -18.02726949 | -49.19189058 | 4 |
| 3702 | JUN | BCL3 | -18.02669127 | -64.02301057 | 6 |
| 3703 | ERBB2 | RICS | -18.01808759 | -45.10912839 | 5 |
| 3704 | TNK2 | SOS1 | -18.0138903 | -35.45411445 | 4 |
| 3705 | TNK2 | WAS | -18.0138903 | -36.59201016 | 4 |
| 3706 | PROC | F2 | -18.0138903 | -54.92376657 | 4 |
| 3707 | CDC6 | MCM2 | -18.0138903 | -48.49441582 | 4 |
| 3708 | ERBB2 | TUB | -18.01201789 | -35.72592447 | 4 |
| 3709 | ERBB2 | AXL | -18.01201789 | -38.12936504 | 4 |
| 3710 | GRIN2A | KCNJ12 | -18.01014515 | -43.61261183 | 4 |
| 3711 | BLNK | GAB2 | -18.01014515 | -40.27788679 | 4 |
| 3712 | BLNK | PAG1 | -18.01014515 | -38.03481087 | 4 |
| 3713 | BCR | IL4R | -18.01014515 | -41.54871993 | 4 |
| 3714 | BCL6 | HDAC9 | -18.01014515 | -43.68405869 | 4 |
| 3715 | HDAC9 | ZNFN1A1 | -18.01014515 | -44.19440304 | 4 |
| 3716 | FN1 | COL4A4 | -18.00850846 | -62.51503663 | 5 |
| 3717 | SH3KBP1 | PDCD6IP | -18.00681354 | -57.1028706 | 4 |
| 3718 | TRADD | TNFRSF1B | -18.00681354 | -40.47852872 | 4 |
| 3719 | SNAP23 | STX7 | -18.00681354 | -55.52160357 | 4 |
| 3720 | RELA | GTF2F2 | -18.00445764 | -61.91604772 | 6 |
| 3721 | AR | RB1 | -18.00281118 | -111.8143928 | 10 |
| 3722 | GRB2 | SH3KBP1 | -18.00105721 | -92.22348062 | 8 |
| 3723 | GRB2 | HCK | -18.00105721 | -91.6354155 | 8 |
| 3724 | TRAF6 | MAP3K7IP2 | -17.99554014 | -49.89382259 | 4 |
| 3725 | JAK2 | SOCS2 | -17.99286677 | -46.11024256 | 4 |
| 3726 | TBP | TAF6 | -17.99286677 | -46.64083602 | 4 |
| 3727 | TBP | TAF9 | -17.99286677 | -46.44399491 | 4 |
| 3728 | NRIP1 | SMARCD1 | -17.99173279 | -37.91462119 | 4 |
| 3729 | RB1 | E2F1 | -17.98855219 | -83.73527681 | 7 |
| 3730 | SYK | PTK2 | -17.98729876 | -68.78527694 | 7 |
| 3731 | GAB3 | BCAR1 | -17.98249054 | -34.90150622 | 4 |
| 3732 | MCM5 | MCM7 | -17.98249054 | -50.36550831 | 4 |
| 3733 | CSF1R | PECAM1 | -17.98234527 | -39.52696911 | 4 |
| 3734 | TERT | AHR | -17.98234527 | -41.99780058 | 4 |
| 3735 | NAPA | SNAP25 | -17.98234527 | -50.26342661 | 4 |
| 3736 | EXOC7 | FLJ32855 | -17.98234527 | -40.86526541 | 4 |
| 3737 | GHR | CSF1R | -17.97853523 | -40.49917714 | 4 |
| 3738 | GHR | LEPR | -17.97853523 | -41.50219263 | 4 |
| 3739 | TYK2 | CSF1R | -17.97853523 | -37.20376473 | 4 |
| 3740 | PTPN1 | VAV3 | -17.97853523 | -38.06594839 | 4 |
| 3741 | PTK2 | RASA1 | -17.97199402 | -66.49754318 | 7 |
| 3742 | PSEN1 | NCSTN | -17.97051041 | -56.70755787 | 4 |
| 3743 | PIK3R1 | RAF1 | -17.95365049 | -91.75173529 | 9 |
| 3744 | FN1 | COL2A1 | -17.93111001 | -76.45373292 | 6 |
| 3745 | BRCA1 | PIN1 | -17.92074405 | -73.20615296 | 7 |
| 3746 | PTK2 | VAV3 | -17.91525681 | -43.70632075 | 5 |
| 3747 | PTK2 | SYN1 | -17.91525681 | -48.23063566 | 5 |
| 3748 | GNAI3 | GNAQ | -17.9148047 | -71.74300713 | 5 |
| 3749 | GAB2 | FYN | -17.90833616 | -64.74240041 | 6 |
| 3750 | ACTB | VASP | -17.9064961 | -66.88813937 | 5 |
| 3751 | HSPA1A | BAG1 | -17.9064961 | -62.21617924 | 5 |
| 3752 | GDF5 | BMP4 | -17.89982615 | -35.58087953 | 3 |
| 3753 | CCL8 | CCL3 | -17.89982615 | -37.44163187 | 3 |
| 3754 | ITGA2 | ITGA1 | -17.89982615 | -42.00858084 | 3 |
| 3755 | SNRPF | SNRPD3 | -17.89982615 | -39.35117437 | 3 |
| 3756 | E2F6 | E2F2 | -17.89982615 | -42.3776783 | 3 |
| 3757 | VAMP1 | VAMP8 | -17.89982615 | -37.51823379 | 3 |
| 3758 | MAP3K7IP2 | MAP3K7IP1 | -17.89982615 | -34.21806863 | 3 |
| 3759 | ARPC3 | ACTR2 | -17.89982615 | -43.22052181 | 3 |
| 3760 | TNFRSF17 | TNFRSF11A | -17.89982615 | -30.56167018 | 3 |
| 3761 | ARL2 | ARL3 | -17.8990106 | -43.02715042 | 3 |
| 3762 | BMP4 | BMP6 | -17.8990106 | -35.58087953 | 3 |
| 3763 | OPRM1 | NGB | -17.8990106 | -33.27928257 | 3 |
| 3764 | F5 | SERPINB6 | -17.8990106 | -37.99728725 | 3 |
| 3765 | MCM5 | ORC5L | -17.8990106 | -34.94844515 | 3 |
| 3766 | ACTR3 | ARPC1B | -17.8990106 | -43.22052181 | 3 |
| 3767 | BAK1 | BCL2L11 | -17.8990106 | -33.90262653 | 3 |
| 3768 | BAK1 | MOAP1 | -17.8990106 | -33.29649073 | 3 |
| 3769 | TGFBI | PRELP | -17.8990106 | -32.63727632 | 3 |
| 3770 | MEP50 | LSM11 | -17.8990106 | -40.19847223 | 3 |
| 3771 | STX12 | SYBL1 | -17.8990106 | -36.47996463 | 3 |
| 3772 | VAMP8 | STX11 | -17.8990106 | -36.15875986 | 3 |
| 3773 | MAP3K7IP1 | MAP2K6 | -17.8990106 | -33.90496494 | 3 |
| 3774 | ARFIP2 | DDEF2 | -17.8990106 | -39.82986872 | 3 |
| 3775 | ARFIP2 | PIP5K1A | -17.8990106 | -39.82986872 | 3 |
| 3776 | DEDD | TNFRSF10B | -17.8990106 | -34.97070252 | 3 |
| 3777 | ACTR2 | ARPC1B | -17.8990106 | -43.22052181 | 3 |
| 3778 | ACTR2 | ARPC2 | -17.8990106 | -43.22052181 | 3 |
| 3779 | ACTR2 | ARPC4 | -17.8990106 | -43.22052181 | 3 |
| 3780 | TAPBP | COPE | -17.8990106 | -38.9746968 | 3 |
| 3781 | ATF3 | HLF | -17.8990106 | -35.64249229 | 3 |
| 3782 | NR3C1 | PGR | -17.89322035 | -57.57933993 | 5 |
| 3783 | RICS | CRKL | -17.88191444 | -55.31830841 | 5 |
| 3784 | ANTXR2 | MMP9 | -17.88131019 | -37.71356558 | 3 |
| 3785 | PTPN1 | IRS1 | -17.8799398 | -51.78390622 | 5 |
| 3786 | CTRB1 | ELA2 | -17.87875978 | -40.94770888 | 3 |
| 3787 | MYF6 | EDF1 | -17.87875978 | -32.95758362 | 3 |
| 3788 | MYF6 | TCF4 | -17.87875978 | -32.95758362 | 3 |
| 3789 | CASP8AP2 | CASP10 | -17.87875978 | -34.23453795 | 3 |
| 3790 | CFL1 | TMSB4X | -17.87508906 | -38.47117048 | 3 |
| 3791 | CD40 | TNFRSF4 | -17.87508906 | -31.98570487 | 3 |
| 3792 | CD40 | TNFRSF18 | -17.87508906 | -30.56167018 | 3 |
| 3793 | CD40 | TNFRSF12A | -17.87508906 | -30.56167018 | 3 |
| 3794 | EDF1 | EMP3 | -17.87508906 | -32.95758362 | 3 |
| 3795 | EDF1 | KCNQ2 | -17.87508906 | -32.95758362 | 3 |
| 3796 | TCF4 | EMP3 | -17.87508906 | -32.95758362 | 3 |
| 3797 | TCF4 | KCNQ2 | -17.87508906 | -32.95758362 | 3 |
| 3798 | PKD1 | PTPN14 | -17.87508906 | -31.18441221 | 3 |
| 3799 | CBX1 | MBD1 | -17.87508906 | -37.35326087 | 3 |
| 3800 | CCNA2 | CKS1B | -17.87437377 | -33.49965794 | 3 |
| 3801 | FYN | SH3BP2 | -17.86106001 | -53.34118918 | 5 |
| 3802 | HDAC2 | ZNFN1A1 | -17.85281737 | -58.10876716 | 5 |
| 3803 | ITGB1 | CD9 | -17.85197689 | -63.59939194 | 5 |
| 3804 | CD3E | CBLB | -17.85128186 | -41.42637564 | 4 |
| 3805 | CD2AP | RICS | -17.85128186 | -37.27992361 | 4 |
| 3806 | TRIP4 | NCOA2 | -17.85128186 | -40.48358308 | 4 |
| 3807 | RICS | PKD1 | -17.8485604 | -39.96196989 | 4 |
| 3808 | GTF2H1 | GTF2F1 | -17.8485604 | -48.54785628 | 4 |
| 3809 | KIAA0408 | PSMF1 | -17.8485604 | -38.18731935 | 4 |
| 3810 | BLNK | EGFR | -17.84433471 | -62.89807118 | 6 |
| 3811 | PAG1 | EGFR | -17.84433471 | -56.34761522 | 6 |
| 3812 | NR3C1 | NFKB1 | -17.84364888 | -86.54845825 | 8 |
| 3813 | CASK | LIN7B | -17.83236037 | -48.16689232 | 4 |
| 3814 | RABAC1 | RTN3 | -17.83236037 | -53.03216176 | 4 |
| 3815 | KIT | CSF3R | -17.83152683 | -36.50177059 | 4 |
| 3816 | KIT | INPP5D | -17.83152683 | -37.39558847 | 4 |
| 3817 | CHUK | TRAF3IP2 | -17.83152683 | -43.0540165 | 4 |
| 3818 | NEDD9 | EVL | -17.83152683 | -39.07556691 | 4 |
| 3819 | CALD1 | MYOG | -17.83110514 | -32.95758362 | 3 |
| 3820 | KCNQ5 | MYOG | -17.83110514 | -32.95758362 | 3 |
| 3821 | GRM5 | MYOG | -17.83110514 | -32.95758362 | 3 |
| 3822 | GEMIN4 | SIP1 | -17.83110514 | -39.09985994 | 3 |
| 3823 | GEMIN4 | SNRPB | -17.83110514 | -39.09985994 | 3 |
| 3824 | RIMS2 | RAB3IP | -17.82906613 | -41.85443016 | 3 |
| 3825 | PKP2 | DSC1 | -17.82906613 | -38.89271673 | 3 |
| 3826 | PKP2 | DSC2 | -17.82906613 | -40.59746482 | 3 |
| 3827 | PKP2 | DSG2 | -17.82906613 | -40.59746482 | 3 |
| 3828 | HOXA1 | FLJ22746 | -17.82906613 | -31.02897204 | 3 |
| 3829 | RBPSUH | JAG1 | -17.82906613 | -38.70966231 | 3 |
| 3830 | TNFRSF14 | TNFRSF9 | -17.82906613 | -30.56167018 | 3 |
| 3831 | TNFRSF14 | TNFRSF19 | -17.82906613 | -30.56167018 | 3 |
| 3832 | CDC6 | CCDC5 | -17.82906613 | -37.45487105 | 3 |
| 3833 | TANK | TNFRSF9 | -17.82906613 | -30.56167018 | 3 |
| 3834 | TANK | TNFRSF19 | -17.82906613 | -30.56167018 | 3 |
| 3835 | DLG4 | LIN7A | -17.82600193 | -59.81148294 | 5 |
| 3836 | AR | SMARCE1 | -17.82600193 | -52.41887325 | 5 |
| 3837 | MDM2 | CHD3 | -17.82476499 | -40.71243172 | 4 |
| 3838 | PPARA | NR4A1 | -17.82476499 | -41.99326055 | 4 |
| 3839 | BRCA1 | BARD1 | -17.82019569 | -62.62626226 | 5 |
| 3840 | RAB3B | MYF5 | -17.80994219 | -32.95758362 | 3 |
| 3841 | RAB3B | KCNQ3 | -17.80994219 | -32.95758362 | 3 |
| 3842 | RAB3B | RALB | -17.80994219 | -32.95758362 | 3 |
| 3843 | MYF5 | KCNQ3 | -17.80994219 | -32.95758362 | 3 |
| 3844 | MYF5 | RALB | -17.80994219 | -32.95758362 | 3 |
| 3845 | KCNQ3 | RALB | -17.80994219 | -32.95758362 | 3 |
| 3846 | EPB41L1 | EPB41L2 | -17.80994219 | -39.81547998 | 3 |
| 3847 | VIPR1 | CRHR1 | -17.80994219 | -33.56135151 | 3 |
| 3848 | MED25 | CDK8 | -17.80994219 | -37.84709697 | 3 |
| 3849 | MED25 | SURB7 | -17.80994219 | -37.84709697 | 3 |
| 3850 | PDLIM1 | MYOZ1 | -17.80994219 | -36.07694493 | 3 |
| 3851 | TXNL2 | CATSPER1 | -17.80994219 | -33.52846666 | 3 |
| 3852 | UBE2D3 | UBE2D1 | -17.80994219 | -40.92239107 | 3 |
| 3853 | KIAA1217 | FLJ12529 | -17.80994219 | -37.28316152 | 3 |
| 3854 | PXN | CD36 | -17.79982033 | -54.55651885 | 5 |
| 3855 | JAK2 | PLCG1 | -17.78189195 | -82.10302697 | 8 |
| 3856 | SP1 | ARID4A | -17.77721263 | -39.36198961 | 4 |
| 3857 | PML | TAF1 | -17.77294352 | -57.42482523 | 6 |
| 3858 | NEDD9 | CBL | -17.772855 | -59.04555222 | 6 |
| 3859 | TRIM37 | KRT15 | -17.772855 | -73.82858337 | 6 |
| 3860 | CD28 | CD2 | -17.76612435 | -43.07266402 | 4 |
| 3861 | ERCC3 | GTF2E2 | -17.76612435 | -50.37190223 | 4 |
| 3862 | KRT19 | EXOC8 | -17.76612435 | -44.27734217 | 4 |
| 3863 | VDR | HNF4A | -17.76612435 | -45.73434791 | 4 |
| 3864 | IRS2 | CSF2RB | -17.76449147 | -39.45341129 | 4 |
| 3865 | GRB2 | SOCS3 | -17.76419743 | -75.8795541 | 7 |
| 3866 | RET | VAV3 | -17.75593716 | -34.99545444 | 4 |
| 3867 | HRMT1L2 | RBMX | -17.75593716 | -54.41008787 | 4 |
| 3868 | NUP62 | NUP153 | -17.75593716 | -49.20128501 | 4 |
| 3869 | GRB7 | SOCS1 | -17.75405003 | -40.42508905 | 4 |
| 3870 | NFKB1 | POU2F1 | -17.75402053 | -59.94760398 | 6 |
| 3871 | SOCS1 | TEC | -17.74806201 | -41.76172571 | 4 |
| 3872 | PTPN11 | RET | -17.74504972 | -61.07119031 | 6 |
| 3873 | PTPN6 | VAV3 | -17.73384708 | -51.84005095 | 5 |
| 3874 | JUN | MYC | -17.73307573 | -81.83268046 | 8 |
| 3875 | BRCA1 | NCOA1 | -17.72878362 | -76.3564755 | 8 |
| 3876 | GHR | TNFRSF1A | -17.72770143 | -47.09579068 | 5 |
| 3877 | PTPN1 | ERBB2 | -17.72770143 | -45.48236603 | 5 |
| 3878 | PIK3R1 | CD3Z | -17.72770107 | -84.28812852 | 7 |
| 3879 | TP53 | TP73 | -17.72733175 | -72.08460038 | 6 |
| 3880 | RAB33A | PLEKHF2 | -17.72715951 | -37.51372167 | 3 |
| 3881 | GRIN2A | ERBB4 | -17.72396141 | -43.61261183 | 4 |
| 3882 | RBBP4 | SUV39H1 | -17.72396141 | -38.26868238 | 4 |
| 3883 | CCNA1 | CCNE1 | -17.72396141 | -44.9485066 | 4 |
| 3884 | TIAM1 | YWHAG | -17.72396141 | -43.59496889 | 4 |
| 3885 | 76P | TU3A | -17.72341712 | -39.61535551 | 4 |
| 3886 | TGFBR2 | SLITL2 | -17.71981456 | -37.19786872 | 3 |
| 3887 | TGFBR2 | FMOD | -17.71981456 | -37.19786872 | 3 |
| 3888 | ERBB2 | PECAM1 | -17.71679927 | -47.05041959 | 5 |
| 3889 | MET | PTK2B | -17.71660069 | -44.72980152 | 5 |
| 3890 | VAV1 | SOS2 | -17.71534842 | -36.45746154 | 4 |
| 3891 | MAGEA11 | RP11-301I17.1 | -17.70780913 | -48.62664988 | 4 |
| 3892 | MYOD1 | NPAS2 | -17.69882927 | -37.66930186 | 4 |
| 3893 | THBS1 | COL7A1 | -17.69882927 | -48.03954497 | 4 |
| 3894 | PTK2B | CD36 | -17.69273755 | -53.09798977 | 5 |
| 3895 | PTK2B | CSF2RB | -17.69273755 | -47.4627587 | 5 |
| 3896 | INPP5D | SHC1 | -17.69041691 | -49.85651892 | 5 |
| 3897 | EGF | SHC1 | -17.69041691 | -52.09954978 | 5 |
| 3898 | EP300 | HDAC1 | -17.67843308 | -110.4474788 | 10 |
| 3899 | MXD1 | SP1 | -17.67763053 | -51.65654196 | 5 |
| 3900 | TRAF1 | TTRAP | -17.67751287 | -48.26434765 | 4 |
| 3901 | PTPN11 | IRS1 | -17.6730073 | -69.34711504 | 7 |
| 3902 | FOS | FOSL1 | -17.66819007 | -45.56062162 | 4 |
| 3903 | CEBPB | PELP1 | -17.66819007 | -36.49608437 | 4 |
| 3904 | PLCG1 | SOCS1 | -17.66688486 | -59.17581618 | 6 |
| 3905 | NR3C1 | HIF1A | -17.65653669 | -59.58710893 | 6 |
| 3906 | RELA | HMGB1 | -17.65653669 | -66.26581394 | 6 |
| 3907 | BLNK | VAV1 | -17.64246696 | -51.9197569 | 5 |
| 3908 | GAB1 | VAV1 | -17.64246696 | -45.3615413 | 5 |
| 3909 | HOMER2 | HOMER1 | -17.63773381 | -41.05673912 | 3 |
| 3910 | CASP8AP2 | CFLAR | -17.63773381 | -30.49424021 | 3 |
| 3911 | HLA-C | HLA-F | -17.63365495 | -40.67944489 | 3 |
| 3912 | PTPRF | PTPRS | -17.63365495 | -39.52367419 | 3 |
| 3913 | PTPRF | PTPRD | -17.63365495 | -39.52367419 | 3 |
| 3914 | PTPRF | PPFIBP1 | -17.63365495 | -39.52367419 | 3 |
| 3915 | PTPRF | PTPN14 | -17.63365495 | -31.18441221 | 3 |
| 3916 | ITGAM | ITGAX | -17.63365495 | -37.89588714 | 3 |
| 3917 | VEGF | PGF | -17.63365495 | -43.176186 | 3 |
| 3918 | MAPK8IP3 | SH3BP5 | -17.63365495 | -37.06693842 | 3 |
| 3919 | MAPK8IP3 | DUSP22 | -17.63365495 | -36.23765906 | 3 |
| 3920 | RAD51 | RAD52 | -17.63365495 | -32.1738906 | 3 |
| 3921 | MAP3K14 | TNFRSF18 | -17.63365495 | -30.56167018 | 3 |
| 3922 | MAP3K14 | TNFRSF12A | -17.63365495 | -30.56167018 | 3 |
| 3923 | RAF1 | TNFAIP3 | -17.62472545 | -54.6410957 | 5 |
| 3924 | TP53 | CSNK2A1 | -17.62317573 | -102.1196744 | 9 |
| 3925 | LMO3 | LNX | -17.62165686 | -58.54814025 | 5 |
| 3926 | CD2AP | PECAM1 | -17.61611933 | -38.56777789 | 4 |
| 3927 | ARNT | AHR | -17.61611933 | -49.2506581 | 4 |
| 3928 | TRIP4 | AHR | -17.61611933 | -38.13654623 | 4 |
| 3929 | TRIP4 | PPARBP | -17.61611933 | -41.2645583 | 4 |
| 3930 | FBF1 | FLJ32855 | -17.61611933 | -37.19880782 | 4 |
| 3931 | GHR | TEK | -17.61285335 | -37.52620185 | 4 |
| 3932 | GHR | EPHA2 | -17.61285335 | -34.81210934 | 4 |
| 3933 | TYK2 | PIK3R2 | -17.61285335 | -40.19067928 | 4 |
| 3934 | PTPN1 | PTPN12 | -17.61285335 | -39.13043519 | 4 |
| 3935 | GTF2F2 | GTF2F1 | -17.61285335 | -45.83584568 | 4 |
| 3936 | CD28 | CBL | -17.61247162 | -48.56607379 | 5 |
| 3937 | KIT | FASLG | -17.59722782 | -53.15851328 | 5 |
| 3938 | IL2RB | IRS1 | -17.5955342 | -48.99802539 | 5 |
| 3939 | DAXX | SUMO1 | -17.5955342 | -54.6366021 | 5 |
| 3940 | NCOA1 | ETS1 | -17.587925 | -48.07856988 | 5 |
| 3941 | KRT15 | KRT20 | -17.587925 | -69.83119962 | 5 |
| 3942 | CASP8 | FAS | -17.58599481 | -63.01209922 | 5 |
| 3943 | ACTB | ACTA1 | -17.58557668 | -61.05905931 | 5 |
| 3944 | ASCL2 | ESR2 | -17.58055603 | -32.95758362 | 3 |
| 3945 | PPEF2 | ESR2 | -17.58055603 | -32.95758362 | 3 |
| 3946 | PPEF1 | ESR2 | -17.58055603 | -32.95758362 | 3 |
| 3947 | LCP2 | CRKL | -17.58008227 | -53.92490953 | 5 |
| 3948 | DCN | ANTXR2 | -17.57280251 | -37.71356558 | 3 |
| 3949 | THRB | HDAC10 | -17.57280251 | -31.25765249 | 3 |
| 3950 | FGR | CSK | -17.56394893 | -43.77941995 | 4 |
| 3951 | GRB7 | SOS1 | -17.56394893 | -39.34136781 | 4 |
| 3952 | F10 | F2 | -17.56394893 | -55.32923167 | 4 |
| 3953 | HRAS | NRAS | -17.55984018 | -51.97445227 | 4 |
| 3954 | TGFBR1 | SMAD3 | -17.54638826 | -165.0279177 | 13 |
| 3955 | RUNX1T1 | SAP30 | -17.54517362 | -41.25549311 | 4 |
| 3956 | CSF3R | STAT5A | -17.54012062 | -40.14490769 | 4 |
| 3957 | PML | HDAC7A | -17.53884315 | -41.96540169 | 4 |
| 3958 | E2F1 | HDAC1 | -17.53755754 | -79.10453684 | 7 |
| 3959 | GTF2B | SP1 | -17.52536768 | -68.45239779 | 6 |
| 3960 | CASP3 | CASP8 | -17.51780031 | -72.05855089 | 6 |
| 3961 | CD28 | SHB | -17.51617232 | -37.44818219 | 4 |
| 3962 | ITK | SHB | -17.51617232 | -41.41516822 | 4 |
| 3963 | CDKN1B | MYBL2 | -17.513995 | -47.38610347 | 4 |
| 3964 | CALD1 | GRM7 | -17.51292348 | -32.95758362 | 3 |
| 3965 | KCNQ5 | GRM7 | -17.51292348 | -32.95758362 | 3 |
| 3966 | GEMIN4 | SNRPD2 | -17.51292348 | -39.09985994 | 3 |
| 3967 | DNAJC7 | RAD17 | -17.51292348 | -40.7935582 | 3 |
| 3968 | NUP50 | RANBP2 | -17.51292348 | -32.79542444 | 3 |
| 3969 | PER2 | CRY1 | -17.51292348 | -43.33033668 | 3 |
| 3970 | RAB11B | RAB11A | -17.51292348 | -44.42894897 | 3 |
| 3971 | UTP14A | KIAA1267 | -17.51292348 | -31.40292029 | 3 |
| 3972 | E2F4 | CREG1 | -17.5104765 | -32.33585507 | 3 |
| 3973 | E2F4 | E2F5 | -17.5104765 | -37.08697917 | 3 |
| 3974 | RYR2 | HOMER3 | -17.5104765 | -38.33763909 | 3 |
| 3975 | ITSN1 | PACSIN3 | -17.5104765 | -38.47117048 | 3 |
| 3976 | GRK5 | AVP | -17.5104765 | -47.94347503 | 3 |
| 3977 | DSP | DSG1 | -17.5104765 | -39.09338743 | 3 |
| 3978 | DSP | DSG2 | -17.5104765 | -39.09338743 | 3 |
| 3979 | MAP1A | KCNA4 | -17.5104765 | -31.63938458 | 3 |
| 3980 | MAP1A | PFDN4 | -17.5104765 | -35.43373748 | 3 |
| 3981 | MAP1A | PIGT | -17.5104765 | -35.43373748 | 3 |
| 3982 | MAP1A | ATP2B4 | -17.5104765 | -31.63938458 | 3 |
| 3983 | MAP1A | GUCY1A2 | -17.5104765 | -31.63938458 | 3 |
| 3984 | CSNK1E | PER2 | -17.5104765 | -40.42161578 | 3 |
| 3985 | MYST2 | UTP14A | -17.5104765 | -35.63412404 | 3 |
| 3986 | ZNF638 | RHPN1 | -17.5104765 | -31.31344028 | 3 |
| 3987 | ITGB1 | ITGB3 | -17.50858287 | -59.32049297 | 5 |
| 3988 | GDF5 | INHBB | -17.49476906 | -35.454236 | 3 |
| 3989 | CSPG3 | CNTN1 | -17.49476906 | -40.5269763 | 3 |
| 3990 | TIMP3 | BCAN | -17.49476906 | -42.21988993 | 3 |
| 3991 | NSD1 | COPS2 | -17.49476906 | -32.64497589 | 3 |
| 3992 | NGB | RGS12 | -17.49476906 | -33.37936603 | 3 |
| 3993 | HLA-DMB | HLA-DRA | -17.49476906 | -39.91143972 | 3 |
| 3994 | HLA-DMA | HLA-DRA | -17.49476906 | -42.39634637 | 3 |
| 3995 | HLA-DMA | CD63 | -17.49476906 | -39.91143972 | 3 |
| 3996 | THRAP4 | ERBP | -17.49476906 | -30.67478204 | 3 |
| 3997 | PAK3 | ARHGEF7 | -17.49476906 | -36.98344797 | 3 |
| 3998 | CRIPT | LIN7A | -17.49476906 | -33.20702537 | 3 |
| 3999 | TAB3 | TRAF3IP2 | -17.49476906 | -30.10921034 | 3 |
| 4000 | CUTC | KCTD17 | -17.49476906 | -32.26350042 | 3 |
| 4001 | EGF | NRG1 | -17.49354565 | -31.63943163 | 3 |
| 4002 | CDC7 | ORC5L | -17.49354565 | -34.94844515 | 3 |
| 4003 | GEMIN5 | SNRPF | -17.49354565 | -38.49372414 | 3 |
| 4004 | U2AF1 | SFRS2IP | -17.49354565 | -36.24973164 | 3 |
| 4005 | RGS7 | NGB | -17.49354565 | -34.42081991 | 3 |
| 4006 | RGS4 | NGB | -17.49354565 | -33.27928257 | 3 |
| 4007 | CD63 | TSPAN4 | -17.49354565 | -38.56086344 | 3 |
| 4008 | TIFA | CUTC | -17.49354565 | -32.26350042 | 3 |
| 4009 | USP7 | TNFRSF17 | -17.49354565 | -30.56167018 | 3 |
| 4010 | KCTD17 | RPIA | -17.49354565 | -35.56180338 | 3 |
| 4011 | SKI | RBL1 | -17.49261526 | -39.03457203 | 4 |
| 4012 | BCAR1 | WAS | -17.48503504 | -46.20978793 | 5 |
| 4013 | PRKDC | WRN | -17.48390362 | -40.85581351 | 4 |
| 4014 | ADAM12 | RASA1 | -17.47232457 | -37.53757036 | 4 |
| 4015 | CD2AP | PTK2 | -17.46604155 | -44.98185978 | 5 |
| 4016 | NR5A1 | RXRA | -17.46604155 | -59.77977833 | 5 |
| 4017 | STAT3 | PTK2B | -17.46432492 | -70.64860937 | 7 |
| 4018 | HDAC1 | ZNFN1A4 | -17.45905372 | -58.03019916 | 5 |
| 4019 | GAB1 | LAT | -17.45719596 | -40.8530493 | 4 |
| 4020 | PAG1 | LAT | -17.45719596 | -40.30349441 | 4 |
| 4021 | BCL6 | HDAC5 | -17.45719596 | -45.64227757 | 4 |
| 4022 | IRS2 | GAB2 | -17.4561073 | -37.10783334 | 4 |
| 4023 | IRS2 | BCR | -17.4561073 | -35.90124288 | 4 |
| 4024 | IRS2 | IL4R | -17.4561073 | -40.740205 | 4 |
| 4025 | CSPG2 | CCBP2 | -17.4561073 | -57.06204861 | 4 |
| 4026 | SYK | TUB | -17.45434334 | -35.87553999 | 4 |
| 4027 | SYK | TRPV4 | -17.45434334 | -37.03803619 | 4 |
| 4028 | SMURF2 | STRAP | -17.45434334 | -37.65198224 | 4 |
| 4029 | PLSCR1 | TSRC1 | -17.44502102 | -69.97382198 | 5 |
| 4030 | CASP8 | DEDD | -17.444455 | -51.75602031 | 4 |
| 4031 | PIK3R1 | KIT | -17.43911292 | -68.38450097 | 7 |
| 4032 | PIK3R1 | NEDD9 | -17.43911292 | -69.54062167 | 7 |
| 4033 | ERBB2IP | LEF1 | -17.4373697 | -36.88999119 | 4 |
| 4034 | TRIM29 | 76P | -17.4373697 | -49.27782509 | 4 |
| 4035 | GHR | INSR | -17.43681309 | -52.42920001 | 5 |
| 4036 | TYK2 | INSR | -17.43681309 | -54.83791088 | 5 |
| 4037 | JAK2 | EPHA2 | -17.43671195 | -44.58746245 | 5 |
| 4038 | JAK2 | PIK3R2 | -17.43671195 | -47.37289445 | 5 |
| 4039 | PTK2 | PIK3R2 | -17.43671195 | -46.90395976 | 5 |
| 4040 | TBP | TRIP4 | -17.43671195 | -47.21842754 | 5 |
| 4041 | TBP | MNAT1 | -17.43671195 | -54.82666493 | 5 |
| 4042 | TBP | GTF2F1 | -17.43671195 | -60.63263601 | 5 |
| 4043 | RXRA | TRIP4 | -17.43671195 | -49.89434861 | 5 |
| 4044 | SUFU | ZIC1 | -17.43263193 | -43.44366536 | 3 |
| 4045 | SUFU | ZIC2 | -17.43263193 | -43.44366536 | 3 |
| 4046 | NID2 | NID | -17.41472635 | -42.88404958 | 3 |
| 4047 | UBE2G2 | UBE2L3 | -17.41472635 | -38.34787226 | 3 |
| 4048 | UBE2G1 | UBE2L3 | -17.41472635 | -34.65899281 | 3 |
| 4049 | PRC1 | TU3A | -17.41472635 | -30.19609442 | 3 |
| 4050 | GAB2 | SLAMF6 | -17.41023931 | -30.03182088 | 3 |
| 4051 | PAG1 | SLAMF6 | -17.41023931 | -30.03182088 | 3 |
| 4052 | BMPR1A | SOSTDC1 | -17.41023931 | -40.01615067 | 3 |
| 4053 | RBBP4 | PHF12 | -17.41023931 | -32.42661977 | 3 |
| 4054 | MAPT | MAP4 | -17.41023931 | -38.1615903 | 3 |
| 4055 | CDH5 | DSC3 | -17.41023931 | -34.81672131 | 3 |
| 4056 | PPFIA1 | PTPRS | -17.41023931 | -42.38587507 | 3 |
| 4057 | PPFIA1 | PTPRD | -17.41023931 | -42.38587507 | 3 |
| 4058 | XRCC5 | TERF2 | -17.41023931 | -39.7398973 | 3 |
| 4059 | KCNJ12 | HTR2C | -17.41023931 | -37.65187201 | 3 |
| 4060 | GRB2 | IRS1 | -17.4101323 | -98.26809184 | 9 |
| 4061 | KHDRBS1 | PLCG1 | -17.40517574 | -59.87618205 | 6 |
| 4062 | CSK | PLCG1 | -17.40517574 | -61.0636518 | 6 |
| 4063 | HDAC3 | HDAC9 | -17.40022434 | -57.5381763 | 5 |
| 4064 | RAPGEF1 | BCAR1 | -17.39770186 | -41.20671306 | 4 |
| 4065 | NUP214 | NUP62 | -17.39420218 | -50.77947038 | 4 |
| 4066 | POM121 | RIBC2 | -17.39420218 | -48.38144639 | 4 |
| 4067 | IL2RB | TEK | -17.39039162 | -38.09674671 | 4 |
| 4068 | RET | TEK | -17.39039162 | -43.67723097 | 4 |
| 4069 | HIF1A | TRIP4 | -17.39039162 | -37.27548103 | 4 |
| 4070 | CCNA2 | PHB | -17.39039162 | -42.41621111 | 4 |
| 4071 | HSPA5 | CALR | -17.38733806 | -64.99001988 | 5 |
| 4072 | POU2F1 | PRKDC | -17.38665673 | -64.092484 | 5 |
| 4073 | BCAR1 | EVL | -17.3862655 | -39.07556691 | 4 |
| 4074 | MUC1 | EGFR | -17.38570251 | -59.95443132 | 6 |
| 4075 | FXR2 | ZNF408 | -17.3847408 | -59.2718606 | 5 |
| 4076 | PTPRC | SOCS1 | -17.38423538 | -49.25985062 | 5 |
| 4077 | ZBTB16 | BAG1 | -17.38278889 | -55.2775715 | 5 |
| 4078 | ZBTB16 | HDAC5 | -17.38278889 | -61.93010878 | 5 |
| 4079 | JAK2 | FYN | -17.37959695 | -94.8581421 | 9 |
| 4080 | MCM10 | TCEB3B | -17.37550392 | -47.91465239 | 4 |
| 4081 | MCM10 | ZNF638 | -17.37550392 | -40.55703805 | 4 |
| 4082 | PTPN11 | SNX2 | -17.36993528 | -55.24247127 | 5 |
| 4083 | NCOA1 | NSD1 | -17.36975717 | -38.6236474 | 4 |
| 4084 | NCOA1 | THRAP4 | -17.36975717 | -42.16927198 | 4 |
| 4085 | REPS1 | RALBP1 | -17.34048256 | -40.05529058 | 3 |
| 4086 | CDK3 | TFDP1 | -17.34048256 | -39.03305127 | 3 |
| 4087 | VIPR1 | RIC8 | -17.34048256 | -33.56135151 | 3 |
| 4088 | CCL7 | CCL3 | -17.34048256 | -37.44163187 | 3 |
| 4089 | FLT1 | NRP1 | -17.34048256 | -43.94344115 | 3 |
| 4090 | EIF3S8 | EIF3S10 | -17.34048256 | -42.12636387 | 3 |
| 4091 | TNFRSF13B | TNFRSF11A | -17.34048256 | -31.04076854 | 3 |
| 4092 | ZNF297B | Cep72 | -17.34048256 | -35.87402656 | 3 |
| 4093 | OPRM1 | RGS14 | -17.34007476 | -33.27928257 | 3 |
| 4094 | RGS16 | CRHR1 | -17.34007476 | -33.46126805 | 3 |
| 4095 | VCAM1 | ICAM3 | -17.34007476 | -39.38552385 | 3 |
| 4096 | LTBR | TNFRSF8 | -17.34007476 | -31.98570487 | 3 |
| 4097 | PCBD1 | MGC2749 | -17.33309083 | -42.18846905 | 4 |
| 4098 | PCBD1 | TSC22D4 | -17.33309083 | -43.83811339 | 4 |
| 4099 | SMN1 | SNRPB | -17.32383409 | -55.21144864 | 4 |
| 4100 | GAB1 | PTK2B | -17.31646138 | -45.12412898 | 5 |
| 4101 | CCND1 | SMARCA4 | -17.31092067 | -46.47823883 | 5 |
| 4102 | CBLB | SHC1 | -17.30943118 | -65.0693685 | 6 |
| 4103 | NR3C1 | EGR1 | -17.29596499 | -45.9806544 | 5 |
| 4104 | RELA | NFKB2 | -17.29596499 | -63.683889 | 5 |
| 4105 | RELA | NFATC2 | -17.29596499 | -52.64166648 | 5 |
| 4106 | RELA | PPARD | -17.29596499 | -49.2529588 | 5 |
| 4107 | RET | CRKL | -17.29595015 | -51.22684433 | 5 |
| 4108 | NCOR2 | EP300 | -17.29571823 | -79.77962049 | 7 |
| 4109 | PTK2B | IL4R | -17.29327847 | -50.44474871 | 5 |
| 4110 | FOS | AHR | -17.28469862 | -50.10977348 | 5 |
| 4111 | CEBPB | AHR | -17.28469862 | -48.64011948 | 5 |
| 4112 | TRAF2 | TRPC4AP | -17.28317589 | -57.00219167 | 5 |
| 4113 | IFNAR1 | TYK2 | -17.28114613 | -39.43373174 | 4 |
| 4114 | MET | GHR | -17.28114613 | -36.05975721 | 4 |
| 4115 | CD19 | LCP2 | -17.28114613 | -40.3025832 | 4 |
| 4116 | CD28 | LCP2 | -17.28114613 | -44.46122338 | 4 |
| 4117 | ITK | BTK | -17.28114613 | -45.62812777 | 4 |
| 4118 | ITK | LCP2 | -17.28114613 | -43.32570079 | 4 |
| 4119 | VAMP2 | SNAP25 | -17.28114613 | -50.88758092 | 4 |
| 4120 | GHR | CSF2RB | -17.2784243 | -37.79270117 | 4 |
| 4121 | PTK2 | CAV1 | -17.27837216 | -75.70044375 | 7 |
| 4122 | FLJ10204 | LNX | -17.27785606 | -61.17796167 | 5 |
| 4123 | KIT | FYN | -17.27391268 | -73.98002253 | 7 |
| 4124 | CREBBP | ATF1 | -17.27149785 | -53.65551168 | 5 |
| 4125 | YWHAH | ESR1 | -17.26896997 | -81.96387685 | 7 |
| 4126 | JUN | GTF2F2 | -17.26171481 | -64.49658917 | 6 |
| 4127 | PTPN6 | CD5 | -17.25557627 | -54.51169124 | 5 |
| 4128 | GRB2 | LAT | -17.25419981 | -81.09820079 | 7 |
| 4129 | LYN | GRAP2 | -17.22950681 | -71.35456522 | 6 |
| 4130 | EGFR | RASA1 | -17.22909954 | -84.91886425 | 8 |
| 4131 | PIK3R1 | RAPGEF1 | -17.22851892 | -50.82996297 | 5 |
| 4132 | PIK3R1 | CSF3R | -17.22851892 | -46.34962755 | 5 |
| 4133 | PIK3R1 | INPP5D | -17.22851892 | -48.65713876 | 5 |
| 4134 | STATIP1 | MDK | -17.22551352 | -31.06121642 | 3 |
| 4135 | PCTK1 | CDC25B | -17.22551352 | -31.52852674 | 3 |
| 4136 | LPL | DAB1 | -17.22551352 | -34.78861349 | 3 |
| 4137 | HIS1 | CDK9 | -17.22551352 | -38.09989673 | 3 |
| 4138 | ZNF24 | ZNF165 | -17.22551352 | -42.70172802 | 3 |
| 4139 | KIF1C | CDC25B | -17.22551352 | -31.34084324 | 3 |
| 4140 | BRCA1 | TOP2A | -17.22307757 | -49.69560561 | 5 |
| 4141 | BRCA1 | EGR1 | -17.22307757 | -44.52864422 | 5 |
| 4142 | ELK1 | DUSP1 | -17.22265851 | -30.5592851 | 3 |
| 4143 | VAV3 | KTN1 | -17.22265851 | -33.32260495 | 3 |
| 4144 | GRIK2 | GRIK1 | -17.22265851 | -33.87419706 | 3 |
| 4145 | GRIK2 | SEMA4C | -17.22265851 | -31.41037591 | 3 |
| 4146 | GRIK2 | GUCY1A2 | -17.22265851 | -31.41037591 | 3 |
| 4147 | GRIA1 | GRIK1 | -17.22265851 | -37.44666616 | 3 |
| 4148 | GADD45B | DUT | -17.22265851 | -36.46991429 | 3 |
| 4149 | ORC1L | ORC3L | -17.22265851 | -37.02446699 | 3 |
| 4150 | SNRPE | GEMIN4 | -17.22265851 | -39.09985994 | 3 |
| 4151 | BAX | BID | -17.22265851 | -34.15394096 | 3 |
| 4152 | SNAP29 | SYT7 | -17.22265851 | -38.83211557 | 3 |
| 4153 | BATF | TEF | -17.22265851 | -38.28154962 | 3 |
| 4154 | RABEP1 | M6PR | -17.22265851 | -41.23539095 | 3 |
| 4155 | NUP153 | NXT1 | -17.22265851 | -37.51776208 | 3 |
| 4156 | DLG4 | CASK | -17.22109016 | -95.65326713 | 7 |
| 4157 | AR | CCND1 | -17.22109016 | -68.41398807 | 7 |
| 4158 | INPPL1 | CRKL | -17.22056538 | -40.00631472 | 4 |
| 4159 | GAB1 | CBL | -17.21246923 | -49.31027614 | 5 |
| 4160 | GAB1 | CBLB | -17.20738027 | -39.28420939 | 4 |
| 4161 | GAB1 | RICS | -17.20738027 | -39.56384258 | 4 |
| 4162 | GAB1 | PDGFRA | -17.20738027 | -40.12089755 | 4 |
| 4163 | GAB2 | CBLB | -17.20738027 | -41.54619648 | 4 |
| 4164 | PAG1 | SHB | -17.20738027 | -40.82075092 | 4 |
| 4165 | RBBP4 | YY1 | -17.20738027 | -42.9446312 | 4 |
| 4166 | CBLB | BCR | -17.20574717 | -37.87259563 | 4 |
| 4167 | ADRBK1 | GRK1 | -17.20232781 | -39.14532232 | 3 |
| 4168 | MUC1 | BTC | -17.20232781 | -31.76459477 | 3 |
| 4169 | RAP1A | RAP2B | -17.20232781 | -40.68534461 | 3 |
| 4170 | LMO3 | FLJ11016 | -17.20232781 | -33.62546268 | 3 |
| 4171 | EPOR | EGFR | -17.20012818 | -69.32795504 | 7 |
| 4172 | PTPN12 | LYN | -17.19881673 | -51.07239972 | 5 |
| 4173 | TRIM37 | MGC2749 | -17.18486573 | -54.66262733 | 5 |
| 4174 | CD40 | RIPK1 | -17.18411967 | -43.16570148 | 4 |
| 4175 | VAV1 | PDGFRB | -17.18285093 | -53.32884591 | 6 |
| 4176 | THAP7 | LNX | -17.17317808 | -37.92560719 | 4 |
| 4177 | MGC17403 | LNX | -17.17317808 | -38.32085075 | 4 |
| 4178 | BAHD1 | LNX | -17.17317808 | -41.8981164 | 4 |
| 4179 | HOOK2 | ZBTB8 | -17.17074059 | -49.17930611 | 4 |
| 4180 | ITGB2 | ITGAL | -17.17044973 | -43.176186 | 3 |
| 4181 | CDK4 | DMTF1 | -17.17044973 | -36.58867815 | 3 |
| 4182 | EXOC8 | 76P | -17.17019623 | -39.8544994 | 4 |
| 4183 | CDH1 | PTPRJ | -17.16995656 | -41.17621236 | 4 |
| 4184 | VAV3 | WAS | -17.16052786 | -35.74161756 | 4 |
| 4185 | SNAP29 | SNAP23 | -17.16052786 | -53.20996864 | 4 |
| 4186 | PIK3R1 | LAT | -17.16030453 | -67.39873686 | 6 |
| 4187 | PIK3R1 | CD2 | -17.16030453 | -66.6632925 | 6 |
| 4188 | KHDRBS1 | CSF1R | -17.15453902 | -38.3007851 | 4 |
| 4189 | KHDRBS1 | SYN1 | -17.15453902 | -39.07063529 | 4 |
| 4190 | SOS1 | MAP4K1 | -17.15453902 | -37.64150739 | 4 |
| 4191 | LSM1 | SNRPE | -17.15453902 | -50.7483798 | 4 |
| 4192 | SNAP23 | NAPA | -17.15453902 | -50.26342661 | 4 |
| 4193 | LNX | ZNF417 | -17.15355818 | -45.19016241 | 4 |
| 4194 | HDAC4 | SIN3A | -17.15281774 | -56.21114111 | 5 |
| 4195 | CSK | PDGFRB | -17.14893106 | -46.6323406 | 5 |
| 4196 | CD22 | GRB2 | -17.14577676 | -58.36197374 | 6 |
| 4197 | FGR | GRB2 | -17.14577676 | -67.67541692 | 6 |
| 4198 | ESR1 | NR4A1 | -17.14324445 | -53.9664878 | 5 |
| 4199 | SMAD1 | SMAD7 | -17.14123183 | -59.82873216 | 5 |
| 4200 | INHBC | ENG | -17.1385022 | -35.77544076 | 3 |
| 4201 | ORC5L | CDC6 | -17.1385022 | -35.56917976 | 3 |
| 4202 | COL13A1 | FBLN1 | -17.1385022 | -34.73358166 | 3 |
| 4203 | SNRPF | SNRPB | -17.1385022 | -39.35117437 | 3 |
| 4204 | NR2C1 | TNFRSF14 | -17.1385022 | -33.28562173 | 3 |
| 4205 | NSD1 | TDG | -17.1385022 | -30.89136819 | 3 |
| 4206 | TRPC4 | ITPR3 | -17.1385022 | -41.08491 | 3 |
| 4207 | TRPC6 | ITPR3 | -17.1385022 | -50.24606013 | 3 |
| 4208 | BMP7 | IGSF1 | -17.13687088 | -35.454236 | 3 |
| 4209 | BMP7 | INHBC | -17.13687088 | -35.454236 | 3 |
| 4210 | AP1B1 | DDEF2 | -17.13687088 | -39.82986872 | 3 |
| 4211 | AP1B1 | PIP5K1A | -17.13687088 | -39.82986872 | 3 |
| 4212 | PCBD1 | CUTC | -17.13687088 | -32.26350042 | 3 |
| 4213 | PROC | THBD | -17.13687088 | -37.97926875 | 3 |
| 4214 | FBLN1 | PRELP | -17.13687088 | -34.73358166 | 3 |
| 4215 | CD8A | KLRD1 | -17.13687088 | -42.12636387 | 3 |
| 4216 | RBPSUH | MAML1 | -17.13687088 | -38.70966231 | 3 |
| 4217 | CDC6 | CDC45L | -17.13687088 | -35.56917976 | 3 |
| 4218 | SIP1 | ZNF265 | -17.13687088 | -36.31906634 | 3 |
| 4219 | TANK | TNFRSF17 | -17.13687088 | -30.56167018 | 3 |
| 4220 | SRC | EPOR | -17.13496686 | -69.74539879 | 7 |
| 4221 | YWHAZ | CHEK1 | -17.1287447 | -45.66342491 | 4 |
| 4222 | RAPGEF1 | PDGFRB | -17.12738457 | -39.30430355 | 4 |
| 4223 | TSRC1 | KRTAP4-12 | -17.12276044 | -67.07222478 | 5 |
| 4224 | PXN | ZAP70 | -17.12011897 | -59.7214545 | 6 |
| 4225 | KCNE2 | KCNE1 | -17.11489081 | -30.13543705 | 2 |
| 4226 | IHH | DHH | -17.11489081 | -30.13543705 | 2 |
| 4227 | IHH | SMO | -17.11489081 | -30.13543705 | 2 |
| 4228 | UCN3 | CRH | -17.11489081 | -30.08664688 | 2 |
| 4229 | PRKAB1 | PRKAB2 | -17.11489081 | -32.03255703 | 2 |
| 4230 | DHH | SMO | -17.11489081 | -30.13543705 | 2 |
| 4231 | TRIM54 | USP13 | -17.11489081 | -30.42311912 | 2 |
| 4232 | ERO1L | ERO1LB | -17.11489081 | -31.18525917 | 2 |
| 4233 | COG2 | COG1 | -17.11489081 | -32.43802214 | 2 |
