# Supplementary material for: Local Network Topology in Human Protein Interaction Data Predicts Functional Association
Source: PLoS One. 2009 Jul 29;4(7):e6410. doi: 10.1371/journal.pone.0006410 (PMC2713831; doi:10.1371/journal.pone.0006410)
Supplement: Table S2 — Predictions of 466 KEGG pathways for 274 proteins and 123 GO annotations for 114 proteins. The 2nd column is the predicted KEGG and GO IDs for proteins in the 1st column, with 3rd column as corresponding KEGG pathway names and GO terms. Ratio is the number of significant partners with the assigned annotation(s) divided by the total number of significant partners. (0.40 MB DOC) [file pone.0006410.s006.doc]

| **Protein** | **KEGG ID** | **KEGG Pathway Name** | **Ratio** |
| --- | --- | --- | --- |
| **DNASE1** | hsa04810 | Regulation of actin cytoskeleton | 2/2 |
| **DLG4** | hsa04080; hsa04530; hsa04720 | Neuroactive ligand-receptor interaction;  Tight junction;  Long-term potentiation | 2/11 |
| **GRIN2B** | hsa04020 | Calcium signaling pathway | 3/6 |
| **PML** | hsa05220 | Chronic myeloid leukemia | 4/30 |
| **CREBBP** | hsa04010; hsa04620; hsa04660; hsa04662 | MAPK signaling pathway;  Toll-like receptor signaling pathway;  T cell receptor signaling pathway;  B cell receptor signaling pathway | 3/12 |
| **NCOR1** | hsa04330 | Notch signaling pathway | 2/9 |
| **NCOR2** | hsa04110; hsa05220 | Cell cycle;  Chronic myeloid leukemia | 3/14 |
| **RARA** | hsa03320; hsa04080 | PPAR signaling pathway;  Neuroactive ligand-receptor interaction | 3/9 |
| **BRCA1** | hsa04110; hsa05212; hsa05220 | Cell cycle;  Pancreatic cancer;  Chronic myeloid leukemia | 4/22 |
| **TGFBR3** | hsa04010; hsa04060; hsa04350; hsa05210; hsa05212; hsa05220 | MAPK signaling pathway;  Cytokine-cytokine receptor interaction;  TGF-beta signaling pathway;  Colorectal cancer;  Pancreatic cancer;  Chronic myeloid leukemia | 2/6 |
| **GDF9** | hsa04350 | TGF-beta signaling pathway | 5/5 |
| **IGSF1** | hsa04060; hsa04350 | Cytokine-cytokine receptor interaction;  TGF-beta signaling pathway | 6/6 |
| **NCOA1** | hsa04920 | Adipocytokine signaling pathway | 5/38 |
| **NR3C1** | hsa04620; hsa04920 | Toll-like receptor signaling pathway;  Adipocytokine signaling pathway | 4/22 |
| **STATIP1** | hsa04060; hsa04630 | Cytokine-cytokine receptor interaction;  Jak-STAT signaling pathway | 2/3 |
| **MET** | hsa04810 | Regulation of actin cytoskeleton | 3/6 |
| **KHDRBS1** | hsa04650; hsa04660 | Natural killer cell mediated cytotoxicity;  T cell receptor signaling pathway | 5/18 |
| **AR** | hsa04110; hsa05212; hsa05220 | Cell cycle;  Pancreatic cancer;  Chronic myeloid leukemia | 3/19 |
| **PTPN11** | hsa04510 | Focal adhesion | 16/50 |
| **RET** | hsa04510 | Focal adhesion | 7/15 |
| **JUN** | hsa05220 | Chronic myeloid leukemia | 7/35 |
| **TRAF3** | hsa04010 | MAPK signaling pathway | 2/4 |
| **LRPAP1** | hsa05010 | Alzheimer's disease | 2/4 |
| **APH1B** | hsa04330; hsa05010 | Notch signaling pathway;  Alzheimer's disease | 2/2 |
| **CD19** | hsa04650 | Natural killer cell mediated cytotoxicity | 5/10 |
| **CD22** | hsa04650; hsa04660 | Natural killer cell mediated cytotoxicity;  T cell receptor signaling pathway | 8/23 |
| **ELA2** | hsa04080 | Neuroactive ligand-receptor interaction | 2/4 |
| **CD3E** | hsa04630; hsa04910; hsa05220 | Jak-STAT signaling pathway;  Insulin signaling pathway;  Chronic myeloid leukemia | 2/6 |
| **GNB2L1** | hsa04630; hsa04650; hsa04670; hsa04910; hsa04920; hsa04930; hsa05220 | Jak-STAT signaling pathway;  Natural killer cell mediated cytotoxicity;  Leukocyte transendothelial migration;  Insulin signaling pathway;  Adipocytokine signaling pathway;  Type II diabetes mellitus;  Chronic myeloid leukemia | 2/3 |
| **TRAF2** | hsa04620 | Toll-like receptor signaling pathway | 4/17 |
| **TRAF1** | hsa04010; hsa04210 | MAPK signaling pathway;  Apoptosis | 4/10 |
| **CSK** | hsa04650 | Natural killer cell mediated cytotoxicity | 10/15 |
| **TNK2** | hsa04630; hsa04810; hsa04910 | Jak-STAT signaling pathway;  Regulation of actin cytoskeleton;  Insulin signaling pathway | 2/3 |
| **KIT** | hsa04630 | Jak-STAT signaling pathway | 11/38 |
| **SIT1** | hsa04670 | Leukocyte transendothelial migration | 5/8 |
| **BLNK** | hsa04650; hsa04660; hsa04664 | Natural killer cell mediated cytotoxicity;  T cell receptor signaling pathway;  Fc epsilon RI signaling pathway | 7/15 |
| **GAB1** | hsa04510 | Focal adhesion | 11/27 |
| **IRS2** | hsa04630 | Jak-STAT signaling pathway | 9/27 |
| **GAB2** | hsa04650 | Natural killer cell mediated cytotoxicity | 11/34 |
| **IRS4** | hsa04060 | Cytokine-cytokine receptor interaction | 4/7 |
| **PTPN12** | hsa04510 | Focal adhesion | 9/15 |
| **ADAM15** | hsa04670 | Leukocyte transendothelial migration | 3/10 |
| **MUC1** | hsa04510 | Focal adhesion | 5/9 |
| **PTPRC** | hsa04650 | Natural killer cell mediated cytotoxicity | 7/15 |
| **TEK** | hsa04060 | Cytokine-cytokine receptor interaction | 4/5 |
| **CD2AP** | hsa04670 | Leukocyte transendothelial migration | 3/10 |
| **DNM1** | hsa04810 | Regulation of actin cytoskeleton | 2/3 |
| **ABL1** | hsa04510 | Focal adhesion | 5/9 |
| **PAG1** | hsa04660 | T cell receptor signaling pathway | 13/45 |
| **CD28** | hsa04640 | Hematopoietic cell lineage | 4/12 |
| **MAPK14** | hsa04510; hsa04910; hsa04930; hsa05210; hsa05212 | Focal adhesion;  Insulin signaling pathway;  Type II diabetes mellitus;  Colorectal cancer;  Pancreatic cancer | 3/3 |
| **RAPGEF1** | hsa04670; hsa04810 | Leukocyte transendothelial migration;  Regulation of actin cytoskeleton | 5/10 |
| **BCL2A1** | hsa01510; hsa04210; hsa05030 | Neurodegenerative Diseases;  Apoptosis;  Amyotrophic lateral sclerosis (ALS) | 2/3 |
| **GAB3** | hsa04060 | Cytokine-cytokine receptor interaction | 5/21 |
| **CSF1R** | hsa04630; hsa04670 | Jak-STAT signaling pathway;  Leukocyte transendothelial migration | 4/17 |
| **GRB7** | hsa04650; hsa05214 | Natural killer cell mediated cytotoxicity;  Glioma | 6/11 |
| **TUB** | hsa04510 | Focal adhesion | 3/8 |
| **FRS2** | hsa04650; hsa05214 | Natural killer cell mediated cytotoxicity;  Glioma | 2/4 |
| **ITK** | hsa04664 | Fc epsilon RI signaling pathway | 8/13 |
| **MST1R** | hsa04510; hsa04810 | Focal adhesion;  Regulation of actin cytoskeleton | 4/10 |
| **PTK2B** | hsa04630 | Jak-STAT signaling pathway | 21/68 |
| **EPHA2** | hsa04920 | Adipocytokine signaling pathway | 3/6 |
| **SCAP1** | hsa04650; hsa04670 | Natural killer cell mediated cytotoxicity;  Leukocyte transendothelial migration | 4/14 |
| **CALD1** | hsa04080 | Neuroactive ligand-receptor interaction | 2/15 |
| **CBLB** | hsa04650 | Natural killer cell mediated cytotoxicity | 8/19 |
| **SYNJ1** | hsa04810 | Regulation of actin cytoskeleton | 2/3 |
| **SH3KBP1** | hsa04010; hsa04510; hsa04910; hsa05220 | MAPK signaling pathway;  Focal adhesion;  Insulin signaling pathway;  Chronic myeloid leukemia | 2/4 |
| **INPP5D** | hsa04630 | Jak-STAT signaling pathway | 6/11 |
| **RASA1** | hsa04510 | Focal adhesion | 11/22 |
| **SHB** | hsa04650; hsa04660 | Natural killer cell mediated cytotoxicity;  T cell receptor signaling pathway | 8/25 |
| **BCR** | hsa04630 | Jak-STAT signaling pathway | 9/17 |
| **SYN1** | hsa04020; hsa04510; hsa04670; hsa04810 | Calcium signaling pathway;  Focal adhesion;  Leukocyte transendothelial migration;  Regulation of actin cytoskeleton | 2/6 |
| **BCL3** | hsa04620; hsa05120 | Toll-like receptor signaling pathway;  Epithelial cell signaling in Helicobacter pylori infection | 2/8 |
| **GIT2** | hsa04810 | Regulation of actin cytoskeleton | 3/3 |
| **HLA-F** | hsa04650 | Natural killer cell mediated cytotoxicity | 4/4 |
| **CTLA4** | hsa04670 | Leukocyte transendothelial migration | 2/7 |
| **SOSTDC1** | hsa04350 | TGF-beta signaling pathway | 3/3 |
| **PCTK1** | hsa04010 | MAPK signaling pathway | 2/6 |
| **REM1** | hsa04010; hsa04510; hsa04910; hsa05210; hsa05212; hsa05218; hsa05220 | MAPK signaling pathway;  Focal adhesion;  Insulin signaling pathway;  Colorectal cancer;  Pancreatic cancer;  Melanoma;  Chronic myeloid leukemia | 3/11 |
| **E2F4** | hsa05220 | Chronic myeloid leukemia | 3/10 |
| **RBBP4** | hsa05220 | Chronic myeloid leukemia | 5/17 |
| **ERBB2IP** | hsa05217 | Basal cell carcinoma | 2/4 |
| **KRT18** | hsa04370; hsa04510 | VEGF signaling pathway;  Focal adhesion | 2/7 |
| **CTNNB1** | hsa04514 | Cell adhesion molecules (CAMs) | 2/4 |
| **RICS** | hsa04510 | Focal adhesion | 8/15 |
| **CTNNA1** | hsa05216 | Thyroid cancer | 2/2 |
| **GRIK2** | hsa00230 | Purine metabolism | 2/7 |
| **BAD** | hsa04010 | MAPK signaling pathway | 2/12 |
| **MYF6** | hsa04080 | Neuroactive ligand-receptor interaction | 2/18 |
| **ASCL2** | hsa04080 | Neuroactive ligand-receptor interaction | 2/18 |
| **PPEF2** | hsa04080 | Neuroactive ligand-receptor interaction | 2/18 |
| **KCNQ5** | hsa04080 | Neuroactive ligand-receptor interaction | 2/15 |
| **PPEF1** | hsa04080 | Neuroactive ligand-receptor interaction | 2/18 |
| **ESR1** | hsa04110 | Cell cycle | 4/21 |
| **GRM7** | hsa04310 | Wnt signaling pathway | 2/11 |
| **EMP3** | hsa04080 | Neuroactive ligand-receptor interaction | 2/17 |
| **CALM3** | hsa04350 | TGF-beta signaling pathway | 2/4 |
| **STX1A** | hsa04130 | SNARE interactions in vesicular transport | 5/6 |
| **ITGB1** | hsa04640 | Hematopoietic cell lineage | 4/6 |
| **SPIB** | hsa04010 | MAPK signaling pathway | 2/2 |
| **FOS** | hsa04310 | Wnt signaling pathway | 4/19 |
| **PKP2** | hsa01430 | Cell Communication | 3/5 |
| **PECAM1** | hsa04510; hsa04640 | Focal adhesion;  Hematopoietic cell lineage | 4/26 |
| **SERPINB13** | hsa04610 | Complement and coagulation cascades | 2/2 |
| **PCAF** | hsa01510; hsa04110; hsa04310; hsa04350; hsa04520; hsa04630; hsa04720; hsa04916; hsa05040 | Neurodegenerative Diseases;  Cell cycle;  Wnt signaling pathway;  TGF-beta signaling pathway;  Adherens junction;  Jak-STAT signaling pathway;  Long-term potentiation;  Melanogenesis;  Huntington's disease | 2/2 |
| **MYC** | hsa04110 | Cell cycle | 2/5 |
| **CEBPB** | hsa04620 | Toll-like receptor signaling pathway | 3/10 |
| **CEBPG** | hsa04010 | MAPK signaling pathway | 4/9 |
| **CKS1B** | hsa04110 | Cell cycle | 2/2 |
| **BIRC5** | hsa04210; hsa04510 | Apoptosis;  Focal adhesion | 3/5 |
| **LYN** | hsa04650 | Natural killer cell mediated cytotoxicity | 12/22 |
| **MCM10** | hsa04110 | Cell cycle | 12/26 |
| **AKAP8** | hsa04110; hsa05212; hsa05214; hsa05218; hsa05220 | Cell cycle;  Pancreatic cancer;  Glioma;  Melanoma;  Chronic myeloid leukemia | 2/3 |
| **CDC25A** | hsa04010 | MAPK signaling pathway | 2/3 |
| **CDC42** | hsa04310 | Wnt signaling pathway | 3/5 |
| **KTN1** | hsa04810 | Regulation of actin cytoskeleton | 2/4 |
| **ACTR3** | hsa04810 | Regulation of actin cytoskeleton | 2/3 |
| **RAC2** | hsa05120 | Epithelial cell signaling in Helicobacter pylori infection | 2/2 |
| **SUV39H1** | hsa00271; hsa04110 | Methionine metabolism;  Cell cycle | 2/13 |
| **PHB** | hsa04110 | Cell cycle | 2/7 |
| **RBBP8** | hsa04110 | Cell cycle | 2/3 |
| **BAK1** | hsa04210 | Apoptosis | 3/7 |
| **PXN** | hsa04650 | Natural killer cell mediated cytotoxicity | 6/16 |
| **COL4A3** | hsa01430; hsa04510; hsa04512 | Cell Communication;  Focal adhesion;  ECM-receptor interaction | 6/7 |
| **COL7A1** | hsa04350 | TGF-beta signaling pathway | 2/3 |
| **SPARC** | hsa01430; hsa04350; hsa04510; hsa04512; hsa04810 | Cell Communication;  TGF-beta signaling pathway;  Focal adhesion;  ECM-receptor interaction;  Regulation of actin cytoskeleton | 2/5 |
| **CD36** | hsa04670 | Leukocyte transendothelial migration | 3/5 |
| **COL4A5** | hsa01430; hsa04510; hsa04512 | Cell Communication;  Focal adhesion;  ECM-receptor interaction | 6/7 |
| **CDK3** | hsa04110 | Cell cycle | 2/3 |
| **DSP** | hsa01430 | Cell Communication | 2/2 |
| **JUND** | hsa04912 | GnRH signaling pathway | 2/4 |
| **ERCC3** | hsa03022 | Basal transcription factors | 2/5 |
| **SUMO1** | hsa04010; hsa04110; hsa05220 | MAPK signaling pathway;  Cell cycle;  Chronic myeloid leukemia | 2/6 |
| **GNAZ** | hsa04916 | Melanogenesis | 3/3 |
| **ENG** | hsa04060; hsa04350 | Cytokine-cytokine receptor interaction;  TGF-beta signaling pathway | 3/7 |
| **GRB10** | hsa04650; hsa04910 | Natural killer cell mediated cytotoxicity;  Insulin signaling pathway | 7/11 |
| **RGS16** | hsa04080 | Neuroactive ligand-receptor interaction | 5/14 |
| **TRIP4** | hsa04920 | Adipocytokine signaling pathway | 4/22 |
| **POU2F1** | hsa04620; hsa05120 | Toll-like receptor signaling pathway; Epithelial cell signaling in Helicobacter pylori infection | 3/14 |
| **PELP1** | hsa04110; hsa04310; hsa04510; hsa05210 | Cell cycle;  Wnt signaling pathway;  Focal adhesion;  Colorectal cancer | 2/8 |
| **MNAT1** | hsa03022 | Basal transcription factors | 2/4 |
| **NRIP1** | hsa04010; hsa04920; hsa05220 | MAPK signaling pathway;  Adipocytokine signaling pathway;  Chronic myeloid leukemia | 2/11 |
| **BTK** | hsa04660 | T cell receptor signaling pathway | 3/3 |
| **CASP8AP2** | hsa04210 | Apoptosis | 4/4 |
| **GRAP2** | hsa04664 | Fc epsilon RI signaling pathway | 5/7 |
| **FOSL1** | hsa04010 | MAPK signaling pathway | 2/5 |
| **TRPV4** | hsa04670 | Leukocyte transendothelial migration | 3/10 |
| **NEDD9** | hsa04510 | Focal adhesion | 7/13 |
| **CD2** | hsa04660 | T cell receptor signaling pathway | 5/6 |
| **CASP7** | hsa05210 | Colorectal cancer | 2/3 |
| **INSR** | hsa04630 | Jak-STAT signaling pathway | 8/17 |
| **HCK** | hsa04650; hsa04660; hsa04664 | Natural killer cell mediated cytotoxicity;  T cell receptor signaling pathway;  Fc epsilon RI signaling pathway | 3/4 |
| **RAP1A** | hsa04530; hsa04810 | Tight junction;  Regulation of actin cytoskeleton | 3/5 |
| **YES1** | hsa04510; hsa04664 | Focal adhesion;  Fc epsilon RI signaling pathway | 3/4 |
| **NGB** | hsa04080 | Neuroactive ligand-receptor interaction | 2/12 |
| **RGS14** | hsa04080 | Neuroactive ligand-receptor interaction | 2/9 |
| **RIC8** | hsa04080 | Neuroactive ligand-receptor interaction | 6/15 |
| **UNC119** | hsa04514 | Cell adhesion molecules (CAMs) | 2/3 |
| **LILRB2** | hsa04612 | Antigen processing and presentation | 2/3 |
| **RHOA** | hsa04010; hsa04370; hsa05120; hsa05212 | MAPK signaling pathway;  VEGF signaling pathway;  Epithelial cell signaling in Helicobacter pylori infection;  Pancreatic cancer | 2/4 |
| **IGF1R** | hsa04630 | Jak-STAT signaling pathway | 7/12 |
| **SHC1** | hsa04630 | Jak-STAT signaling pathway | 15/50 |
| **SOCS2** | hsa04920 | Adipocytokine signaling pathway | 3/3 |
| **SOCS3** | hsa04650 | Natural killer cell mediated cytotoxicity | 6/10 |
| **PRKCD** | hsa04010; hsa04020; hsa04070; hsa04310; hsa04370; hsa04510; hsa04540; hsa04650; hsa04670; hsa04720; hsa04730; hsa04916; hsa05214 | MAPK signaling pathway;  Calcium signaling pathway;  Phosphatidylinositol signaling system;  Wnt signaling pathway;  VEGF signaling pathway;  Focal adhesion;  Gap junction;  Natural killer cell mediated cytotoxicity;  Leukocyte transendothelial migration;  Long-term potentiation;  Long-term depression;  Melanogenesis;  Glioma | 3/3 |
| **SERPINB6** | hsa04610 | Complement and coagulation cascades | 2/2 |
| **BIK** | hsa04210 | Apoptosis | 3/5 |
| **BCL2L11** | hsa01510; hsa04210; hsa05030; hsa05210 | Neurodegenerative Diseases;  Apoptosis;  Amyotrophic lateral sclerosis (ALS);  Colorectal cancer | 2/4 |
| **PMAIP1** | hsa04210 | Apoptosis | 3/7 |
| **TRAF5** | hsa04010 | MAPK signaling pathway | 2/4 |
| **CD5** | hsa04660 | T cell receptor signaling pathway | 6/10 |
| **ASIP** | hsa04080; hsa04920 | Neuroactive ligand-receptor interaction; Adipocytokine signaling pathway | 2/2 |
| **MAP1A** | hsa04020 | Calcium signaling pathway | 2/7 |
| **CCBP2** | hsa04060 | Cytokine-cytokine receptor interaction | 4/6 |
| **JUNB** | hsa04010 | MAPK signaling pathway | 3/4 |
| **SP1** | hsa05220 | Chronic myeloid leukemia | 4/15 |
| **SKI** | hsa04110; hsa04350 | Cell cycle;  TGF-beta signaling pathway | 3/3 |
| **MAP3K8** | hsa04210; hsa04620; hsa04662; hsa04920; hsa05120; hsa05212; hsa05220 | Apoptosis;  Toll-like receptor signaling pathway;  B cell receptor signaling pathway;  Adipocytokine signaling pathway;  Epithelial cell signaling in Helicobacter pylori infection;  Pancreatic cancer;  Chronic myeloid leukemia | 2/2 |
| **BRAF** | hsa04110; hsa04370 | Cell cycle;  VEGF signaling pathway | 2/7 |
| **RAP2A** | hsa04010 | MAPK signaling pathway | 4/5 |
| **BATF** | hsa04010 | MAPK signaling pathway | 3/10 |
| **TOB1** | hsa05217 | Basal cell carcinoma | 2/5 |
| **RASIP1** | hsa04670; hsa05210; hsa05212 | Leukocyte transendothelial migration;  Colorectal cancer;  Pancreatic cancer | 2/6 |
| **CRKL** | hsa04650 | Natural killer cell mediated cytotoxicity | 14/43 |
| **SLA** | hsa04650 | Natural killer cell mediated cytotoxicity | 4/5 |
| **UBE2L3** | hsa04120 | Ubiquitin mediated proteolysis | 2/5 |
| **HDAC3** | hsa04330 | Notch signaling pathway | 3/16 |
| **KCNA4** | hsa04020 | Calcium signaling pathway | 3/8 |
| **KSR2** | hsa04010 | MAPK signaling pathway | 4/5 |
| **PRKCG** | hsa04664; hsa04912 | Fc epsilon RI signaling pathway;  GnRH signaling pathway | 3/3 |
| **RHOG** | hsa04360; hsa04510; hsa04520; hsa04670; hsa04810 | Axon guidance;  Focal adhesion;  Adherens junction;  Leukocyte transendothelial migration;  Regulation of actin cytoskeleton | 3/3 |
| **NAPA** | hsa04130 | SNARE interactions in vesicular transport | 4/6 |
| **ERBB3** | hsa04320; hsa04510 | Dorso-ventral axis formation;  Focal adhesion | 3/3 |
| **STRAP** | hsa04350 | TGF-beta signaling pathway | 2/5 |
| **TNFAIP3** | hsa04010 | MAPK signaling pathway | 5/15 |
| **ZNF8** | hsa05217 | Basal cell carcinoma | 2/5 |
| **ALS2CR2** | hsa04010; hsa04620 | MAPK signaling pathway;  Toll-like receptor signaling pathway | 2/2 |
| **CASP9** | hsa01510; hsa05010; hsa05050 | Neurodegenerative Diseases;  Alzheimer's disease;  Dentatorubropallidoluysian atrophy (DRPLA) | 2/3 |
| **DIABLO** | hsa04210 | Apoptosis | 3/4 |
| **KCNJ12** | hsa04020 | Calcium signaling pathway | 5/17 |
| **MAP3K14** | hsa04060 | Cytokine-cytokine receptor interaction | 6/17 |
| **TRPC4AP** | hsa04210 | Apoptosis | 8/13 |
| **TANK** | hsa04060 | Cytokine-cytokine receptor interaction | 5/9 |
| **SH3BP5** | hsa04010 | MAPK signaling pathway | 2/3 |
| **SIN3A** | hsa04110; hsa04330; hsa05220 | Cell cycle;  Notch signaling pathway;  Chronic myeloid leukemia | 3/16 |
| **E2F2** | hsa04110; hsa05212; hsa05214; hsa05218; hsa05220 | Cell cycle;  Pancreatic cancer;  Glioma;  Melanoma;  Chronic myeloid leukemia | 2/3 |
| **PLCG2** | hsa04660 | T cell receptor signaling pathway | 6/8 |
| **MCL1** | hsa01510; hsa04210; hsa05030 | Neurodegenerative Diseases;  Apoptosis;  Amyotrophic lateral sclerosis (ALS) | 2/3 |
| **AHR** | hsa03320; hsa04010; hsa04620; hsa04660; hsa04662; hsa04920; hsa05210 | PPAR signaling pathway;  MAPK signaling pathway;  Toll-like receptor signaling pathway;  T cell receptor signaling pathway;  B cell receptor signaling pathway;  Adipocytokine signaling pathway;  Colorectal cancer | 2/13 |
| **HNF4A** | hsa04080 | Neuroactive ligand-receptor interaction | 2/7 |
| **PPARBP** | hsa04920 | Adipocytokine signaling pathway | 3/23 |
| **DUSP22** | hsa04010 | MAPK signaling pathway | 2/3 |
| **ID2** | hsa04020; hsa04070; hsa04720; hsa04740; hsa04910; hsa04912; hsa04916; hsa05040; hsa05214 | Calcium signaling pathway;  Phosphatidylinositol signaling system;  Long-term potentiation;  Olfactory transduction;  Insulin signaling pathway;  GnRH signaling pathway;  Melanogenesis;  Huntington's disease;  Glioma | 2/6 |
| **PPARD** | hsa04920 | Adipocytokine signaling pathway | 2/5 |
| **SH3BP2** | hsa04660 | T cell receptor signaling pathway | 7/12 |
| **KCNJ4** | hsa04020 | Calcium signaling pathway | 3/10 |
| **BIRC7** | hsa04210; hsa04510 | Apoptosis;  Focal adhesion | 3/4 |
| **SNIP1** | hsa05217 | Basal cell carcinoma | 2/3 |
| **CRIPT** | hsa04020 | Calcium signaling pathway | 4/14 |
| **SEMA4C** | hsa00230 | Purine metabolism | 2/7 |
| **GDA** | hsa04020 | Calcium signaling pathway | 3/8 |
| **KCNJ10** | hsa04020; hsa04080; hsa04720 | Calcium signaling pathway;  Neuroactive ligand-receptor interaction;  Long-term potentiation | 3/5 |
| **PGF** | hsa04060 | Cytokine-cytokine receptor interaction | 2/3 |
| **SIN3B** | hsa04330; hsa05220 | Notch signaling pathway;  Chronic myeloid leukemia | 3/4 |
| **ZNFN1A1** | hsa04330; hsa05220 | Notch signaling pathway;  Chronic myeloid leukemia | 3/8 |
| **THRB** | hsa03320 | PPAR signaling pathway | 3/8 |
| **USP7** | hsa04060 | Cytokine-cytokine receptor interaction | 11/15 |
| **TRIM37** | hsa04060 | Cytokine-cytokine receptor interaction | 5/13 |
| **MAP4K1** | hsa04910 | Insulin signaling pathway | 3/5 |
| **DEDD** | hsa04210 | Apoptosis | 4/5 |
| **CDC5L** | hsa04110 | Cell cycle | 4/5 |
| **KIF1C** | hsa04010 | MAPK signaling pathway | 2/7 |
| **DAB1** | hsa05010 | Alzheimer's disease | 2/4 |
| **RIPK2** | hsa04210 | Apoptosis | 4/12 |
| **ACTR2** | hsa04810 | Regulation of actin cytoskeleton | 5/6 |
| **TP73** | hsa04110; hsa05212; hsa05214; hsa05218; hsa05220 | Cell cycle;  Pancreatic cancer;  Glioma;  Melanoma;  Chronic myeloid leukemia | 2/2 |
| **DMTF1** | hsa04110; hsa05220 | Cell cycle;  Chronic myeloid leukemia | 3/5 |
| **DIPA** | hsa01430 | Cell Communication | 2/19 |
| **USHBP1** | hsa01430 | Cell Communication | 3/4 |
| **CCNH** | hsa00500; hsa00790 | Starch and sucrose metabolism;  Folate biosynthesis | 2/4 |
| **HOOK2** | hsa01430 | Cell Communication | 2/9 |
| **ESR2** | hsa03320; hsa05216 | PPAR signaling pathway;  Thyroid cancer | 2/8 |
| **IGF2** | hsa04150 | mTOR signaling pathway | 2/3 |
| **ATF3** | hsa04010 | MAPK signaling pathway | 3/8 |
| **FOSL2** | hsa04010 | MAPK signaling pathway | 3/8 |
| **UCP2** | hsa04010; hsa04510; hsa04910; hsa05210; hsa05212; hsa05218; hsa05220 | MAPK signaling pathway;  Focal adhesion;  Insulin signaling pathway;  Colorectal cancer;  Pancreatic cancer;  Melanoma;  Chronic myeloid leukemia | 2/5 |
| **UCP3** | hsa04010; hsa04510; hsa04910; hsa05210; hsa05212; hsa05218; hsa05220 | MAPK signaling pathway;  Focal adhesion;  Insulin signaling pathway;  Colorectal cancer;  Pancreatic cancer;  Melanoma;  Chronic myeloid leukemia | 2/5 |
| **TEF** | hsa04010 | MAPK signaling pathway | 3/7 |
| **HLF** | hsa04010 | MAPK signaling pathway | 2/8 |
| **KCNQ2** | hsa04080 | Neuroactive ligand-receptor interaction | 2/17 |
| **NID** | hsa04512 | ECM-receptor interaction | 2/3 |
| **IRS1** | hsa04510 | Focal adhesion | 8/21 |
| **CBL** | hsa04650 | Natural killer cell mediated cytotoxicity | 17/62 |
| **RALGDS** | hsa04670 | Leukocyte transendothelial migration | 2/5 |
| **TRAF3IP2** | hsa04010; hsa04210; hsa04660; hsa05120 | MAPK signaling pathway;  Apoptosis;  T cell receptor signaling pathway;  Epithelial cell signaling in Helicobacter pylori infection | 2/8 |
| **LILRB1** | hsa04612 | Antigen processing and presentation | 2/3 |
| **NOG** | hsa04060 | Cytokine-cytokine receptor interaction | 2/3 |
| **CDC25B** | hsa04910 | Insulin signaling pathway | 4/13 |
| **CDH2** | hsa04520; hsa05130; hsa05131; hsa05216 | Adherens junction;  Pathogenic Escherichia coli infection - EHEC;  Pathogenic Escherichia coli infection - EPEC;  Thyroid cancer | 2/3 |
| **SKIL** | hsa04110 | Cell cycle | 3/8 |
| **GUCY1A2** | hsa04020 | Calcium signaling pathway | 4/15 |
| **NFE2L1** | hsa04010 | MAPK signaling pathway | 2/6 |
| **ZIC1** | hsa04340 | Hedgehog signaling pathway | 3/3 |
| **ZIC2** | hsa05217 | Basal cell carcinoma | 2/3 |
| **GMFB** | hsa04010 | MAPK signaling pathway | 6/6 |
| **STXBP6** | hsa04130 | SNARE interactions in vesicular transport | 2/2 |
| **CCL3L1** | hsa04060 | Cytokine-cytokine receptor interaction | 4/4 |
| **Rasd2*** | mmu05212 | Pancreatic cancer | 4/24 |
| **Rps27a*** | mmu04620 | Toll-like receptor signaling pathway | 2/15 |
| **Sqstm1*** | mmu04010; mmu04810 | MAPK signaling pathway;  Regulation of actin cytoskeleton | 2/13 |
| **Map2k3*** | mmu04810; mmu05212 | Regulation of actin cytoskeleton;  Pancreatic cancer | 2/13 |
| **Uhmk1*** | mmu04620 | Toll-like receptor signaling pathway | 2/15 |
| **Rhod*** | mmu04010; mmu04620 | MAPK signaling pathway;  Toll-like receptor signaling pathway | 2/15 |
| **CCDC5** | hsa04110 | Cell cycle | 5/6 |
| **Protein** | **GO ID** | **GO Term** | **Ratio** |
| **APP** | GO:0005509 | calcium ion binding | 4/9 |
| **DLG4** | GO:0004385 | guanylate kinase activity | 4/11 |
| **NCOA1** | GO:0008270 | zinc ion binding | 13/38 |
| **KHDRBS1** | GO:0005524 | ATP binding | 5/18 |
| **RELA** | GO:0008270 | zinc ion binding | 11/31 |
| **PTPN11** | GO:0005524 | ATP binding | 16/50 |
| **GHR** | GO:0005524 | ATP binding | 14/33 |
| **IL2RB** | GO:0005524 | ATP binding | 8/17 |
| **JUN** | GO:0008270 | zinc ion binding | 12/35 |
| **SAA1** | GO:0005509 | calcium ion binding | 4/11 |
| **CD19** | GO:0005524 | ATP binding | 4/10 |
| **CD22** | GO:0005524 | ATP binding | 9/23 |
| **PIK3R1** | GO:0005524 | ATP binding | 21/66 |
| **PTPN6** | GO:0005524 | ATP binding | 11/35 |
| **TRAF1** | GO:0005524; GO:0043123 | ATP binding;  positive regulation of I-kappaB kinase/NF-kappaB cascade | 4/10 |
| **PTPN1** | GO:0005159 | insulin-like growth factor receptor binding | 4/14 |
| **GRB2** | GO:0005524 | ATP binding | 23/61 |
| **BLNK** | GO:0005070 | SH3/SH2 adaptor activity | 4/15 |
| **IRS2** | GO:0005524 | ATP binding | 7/27 |
| **EPOR** | GO:0005524 | ATP binding | 12/38 |
| **GAB2** | GO:0005070; GO:0005524 | SH3/SH2 adaptor activity;  ATP binding | 6/34 |
| **PTPN12** | GO:0005524 | ATP binding | 7/15 |
| **PTPRC** | GO:0005524 | ATP binding | 4/15 |
| **CD2AP** | GO:0005524 | ATP binding | 4/10 |
| **VAV1** | GO:0005524 | ATP binding | 14/43 |
| **PAG1** | GO:0005524 | ATP binding | 10/45 |
| **CD28** | GO:0005070 | SH3/SH2 adaptor activity | 4/12 |
| **RAPGEF1** | GO:0005524 | ATP binding | 5/10 |
| **GAB3** | GO:0005524 | ATP binding | 6/21 |
| **TUB** | GO:0005524 | ATP binding | 4/8 |
| **SOS1** | GO:0005524 | ATP binding | 6/19 |
| **PLCG1** | GO:0005524 | ATP binding | 13/49 |
| **BCAR1** | GO:0005524 | ATP binding | 6/28 |
| **RASA1** | GO:0005524 | ATP binding | 6/22 |
| **SOCS1** | GO:0005524 | ATP binding | 6/23 |
| **VAV3** | GO:0005524 | ATP binding | 5/17 |
| **SHB** | GO:0005524 | ATP binding | 7/25 |
| **CRK** | GO:0005524 | ATP binding | 4/13 |
| **BCR** | GO:0005524 | ATP binding | 6/17 |
| **TBP** | GO:0008270 | zinc ion binding | 7/20 |
| **REM1** | GO:0005524 | ATP binding | 4/11 |
| **RBBP4** | GO:0008270 | zinc ion binding | 7/17 |
| **RICS** | GO:0005524 | ATP binding | 5/15 |
| **GNAI1** | GO:0003924 | GTPase activity | 4/4 |
| **FOS** | GO:0008270 | zinc ion binding | 6/19 |
| **PECAM1** | GO:0005524 | ATP binding | 9/26 |
| **FN1** | GO:0005509 | calcium ion binding | 6/18 |
| **RB1** | GO:0008270 | zinc ion binding | 6/16 |
| **GADD45G** | GO:0030521 | androgen receptor signaling pathway | 4/8 |
| **MCM10** | GO:0008270 | zinc ion binding | 10/26 |
| **ORC2L** | GO:0005524 | ATP binding | 8/11 |
| **ASK** | GO:0005524 | ATP binding | 6/8 |
| **GTF2H1** | GO:0005524 | ATP binding | 4/9 |
| **HDAC1** | GO:0008270 | zinc ion binding | 8/24 |
| **PXN** | GO:0005524 | ATP binding | 5/16 |
| **DCN** | GO:0005509 | calcium ion binding | 5/15 |
| **TGFBI** | GO:0005509 | calcium ion binding | 4/13 |
| **OSM** | GO:0005509 | calcium ion binding | 4/12 |
| **ANTXR2** | GO:0005509 | calcium ion binding | 4/10 |
| **HABP2** | GO:0005509 | calcium ion binding | 4/12 |
| **COL1A1** | GO:0005587 | collagen type IV | 6/12 |
| **COL1A2** | GO:0005587 | collagen type IV | 6/9 |
| **POU2F1** | GO:0008270 | zinc ion binding | 5/14 |
| **FAS** | GO:0043123 | positive regulation of I-kappaB kinase/NF-kappaB cascade | 5/6 |
| **CASP10** | GO:0043123 | positive regulation of I-kappaB kinase/NF-kappaB cascade | 4/6 |
| **TIF1** | GO:0003714 | transcription corepressor activity | 4/12 |
| **SHC1** | GO:0005524 | ATP binding | 16/50 |
| **SOCS3** | GO:0005159 | insulin-like growth factor receptor binding | 4/10 |
| **IL4R** | GO:0005524; GO:0043560 | ATP binding;  insulin receptor substrate binding | 4/12 |
| **PIK3R2** | GO:0005524 | ATP binding | 4/10 |
| **LCP2** | GO:0005524 | ATP binding | 7/25 |
| **GTF2E2** | GO:0005524 | ATP binding | 6/9 |
| **CRKL** | GO:0005524 | ATP binding | 16/43 |
| **HDAC3** | GO:0008270 | zinc ion binding | 5/16 |
| **SERPINE2** | GO:0005509 | calcium ion binding | 4/12 |
| **ARID4A** | GO:0008270 | zinc ion binding | 4/5 |
| **TNFAIP3** | GO:0005524 | ATP binding | 7/15 |
| **KCNJ12** | GO:0005509; GO:0005524 | calcium ion binding;  ATP binding | 5/17 |
| **MAP3K14** | GO:0043123 | positive regulation of I-kappaB kinase/NF-kappaB cascade | 7/17 |
| **SIN3A** | GO:0003714; GO:0008270 | transcription corepressor activity;  zinc ion binding | 4/16 |
| **NCOA2** | GO:0008270; GO:0030521 | zinc ion binding;  androgen receptor signaling pathway | 5/12 |
| **AHR** | GO:0008270 | zinc ion binding | 7/13 |
| **PPARBP** | GO:0008270 | zinc ion binding | 9/23 |
| **KCNJ4** | GO:0000287; GO:0005509 | magnesium ion binding;  calcium ion binding | 4/10 |
| **CRIPT** | GO:0005509 | calcium ion binding | 4/14 |
| **CDC45L** | GO:0005524 | ATP binding | 4/8 |
| **ORC3L** | GO:0005524 | ATP binding | 4/5 |
| **HDAC9** | GO:0008270 | zinc ion binding | 5/12 |
| **NR0B2** | GO:0030521; GO:0050681 | androgen receptor signaling pathway;  androgen receptor binding | 4/10 |
| **USP7** | GO:0043123 | positive regulation of I-kappaB kinase/NF-kappaB cascade | 4/15 |
| **TNFRSF19** | GO:0043123 | positive regulation of I-kappaB kinase/NF-kappaB cascade | 4/19 |
| **ACTR2** | GO:0005885 | Arp2/3 protein complex | 6/6 |
| **RIBC2** | GO:0008270 | zinc ion binding | 4/14 |
| **FLJ32855** | GO:0008270 | zinc ion binding | 6/19 |
| **TU3A** | GO:0008270 | zinc ion binding | 9/27 |
| **UTP14A** | GO:0008270 | zinc ion binding | 4/7 |
| **C1orf65** | GO:0008270 | zinc ion binding | 4/8 |
| **WAS** | GO:0005524 | ATP binding | 7/23 |
| **IRS1** | GO:0005524 | ATP binding | 8/21 |
| **CBL** | GO:0005524 | ATP binding | 18/62 |
| **TNFRSF17** | GO:0005031 | tumor necrosis factor receptor activity | 4/11 |
| **ATP2B4** | GO:0030955 | potassium ion binding | 4/16 |
| **TRAF3IP2** | GO:0005524 | ATP binding | 4/8 |
| **CDC25B** | GO:0005524 | ATP binding | 4/13 |
| **GUCY1A2** | GO:0005509 | calcium ion binding | 4/15 |
| **TNFRSF8** | GO:0005031 | tumor necrosis factor receptor activity | 4/14 |
| **TNFRSF11A** | GO:0005524; GO:0043123 | ATP binding;  positive regulation of I-kappaB kinase/NF-kappaB cascade | 4/17 |
| **Rps27a*** | GO:0005525 | GTP binding | 6/15 |
| **Fbxo3*** | GO:0005525 | GTP binding | 6/13 |
| **Sqstm1*** | GO:0005525 | GTP binding | 6/13 |
| **Map2k3*** | GO:0005525 | GTP binding | 5/13 |
| **Uhmk1*** | GO:0005525 | GTP binding | 6/15 |
| **KIAA1267** | GO:0008270 | zinc ion binding | 4/15 |
| **CCDC5** | GO:0005524 | ATP binding | 4/6 |

* indicates mouse proteins.
