# Supplementary material for: Local Network Topology in Human Protein Interaction Data Predicts Functional Association
Source: PLoS One. 2009 Jul 29;4(7):e6410. doi: 10.1371/journal.pone.0006410 (PMC2713831; doi:10.1371/journal.pone.0006410)
Supplement: Figure S2 — A cluster that consists of 1729 human proteins. Indices above protein names are their coordinates in this cluster. (0.08 MB PDF) [file pone.0006410.s003.pdf]

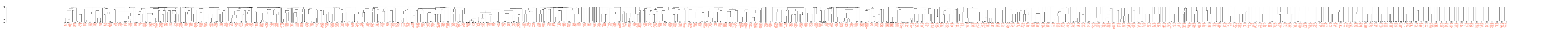

10
8
6
4
2
0
